# Natural history of Ebola virus disease in rhesus monkeys shows viral variant emergence dynamics and tissue-specific host responses

**DOI:** 10.1016/j.xgen.2023.100440

**Published:** 2023-11-21

**Authors:** Erica Normandin, Sergio Triana, Siddharth S. Raju, Tammy C.T. Lan, Kim Lagerborg, Melissa Rudy, Gordon C. Adams, Katherine C. DeRuff, James Logue, David Liu, Daniel Strebinger, Arya Rao, Katelyn S. Messer, Molly Sacks, Ricky D. Adams, Krisztina Janosko, Dylan Kotliar, Rickey Shah, Ian Crozier, John L. Rinn, Marta Melé, Anna N. Honko, Feng Zhang, Mehrtash Babadi, Jeremy Luban, Richard S. Bennett, Alex K. Shalek, Nikolaos Barkas, Aaron E. Lin, Lisa E. Hensley, Pardis C. Sabeti, Katherine J. Siddle

**Affiliations:** 1 Broad Institute of Harvard and MIT, Cambridge, MA 02142, USA; 2 Department of Systems Biology, Harvard Medical School, Boston, MA 02115, USA; 3 Harvard-MIT Division of Health Sciences and Technology, Massachusetts Institute of Technology, Cambridge, MA 02142, USA; 4 Department of Chemistry, Institute for Medical Engineering and Sciences (IMES), and Koch Institute for Integrative Cancer Research, MIT, Cambridge, MA 02142, USA; 5 Ragon Institute of MGH, Harvard, and MIT, Cambridge, MA 02139, USA; 6 Department of Molecular and Cellular Biology, Harvard University, Boston, MA, USA; 7 Harvard Program in Biological and Biomedical Sciences, Harvard Medical School, Boston, MA, USA; 8 Division of Infectious Diseases, Massachusetts General Hospital, Boston, MA 02114, USA; 9 Integrated Research Facility, Division of Clinical Research, National Institute of Allergy and Infectious Diseases, National Institutes of Health, Frederick, MD 21702, USA; 10 Howard Hughes Medical Institute, Chevy Chase, MD 20815-6789, USA; 11 McGovern Institute for Brain Research, Massachusetts Institute of Technology, Cambridge, MA 02139, USA; 12 Department of Brain and Cognitive Sciences, Massachusetts Institute of Technology, Cambridge, MA 02139, USA; 13 Department of Biological Engineering, Massachusetts Institute of Technology, Cambridge, MA 02139, USA; 14 Columbia University, New York, NY, USA; 15 Harvard/MIT MD-PhD Program, Harvard Medical School, Boston, MA 02115, USA; 16 Clinical Monitoring Research Program Directorate, Frederick National Laboratory for Cancer Research, Frederick, MD 21702, USA; 17 Department of Biochemistry, University of Colorado Boulder, Boulder, CO 80303, USA; 18 Life Sciences Department, Barcelona Supercomputing Center, 08034 Barcelona, Catalonia, Spain; 19 National Emerging Infectious Diseases Laboratories, Boston University, Boston, MA 02118, USA; 20 Program in Molecular Medicine, University of Massachusetts Chan Medical School, Worcester, MA 01655, USA; 21 Harvard Program in Virology, Harvard Medical School, Boston, MA 02115, USA; 22 Department of Molecular Biology, Princeton University, Princeton, NJ 08544, USA; 23 Lewis-Sigler Institute for Integrative Genomics, Princeton University, Princeton, NJ 08544, USA; 24 Department of Organismic and Evolutionary Biology, Harvard University, Cambridge, MA 02138, USA; 25 Department of Immunology and Infectious Diseases, Harvard T.H. Chan School of Public Health, Boston, MA 02115, USA; 26 Department of Molecular Microbiology and Immunology, Brown University, Providence, RI 02912, USA

**Keywords:** Ebola virus, transcriptomics, host-virus interactions, viral variants, Ebola virus disease, minigenome, hemorrhagic fevers, non-human primates, deconvolution

## Abstract

Ebola virus (EBOV) causes Ebola virus disease (EVD), marked by severe hemorrhagic fever; however, the mechanisms underlying the disease remain unclear. To assess the molecular basis of EVD across time, we performed RNA sequencing on 17 tissues from a natural history study of 21 rhesus monkeys, developing new methods to characterize host-pathogen dynamics. We identified alterations in host gene expression with previously unknown tissue-specific changes, including downregulation of genes related to tissue connectivity. EBOV was widely disseminated throughout the body; using a new, broadly applicable deconvolution method, we found that viral load correlated with increased monocyte presence. Patterns of viral variation between tissues differentiated primary infections from compartmentalized infections, and several variants impacted viral fitness in a EBOV/Kikwit minigenome system, suggesting that functionally significant variants can emerge during early infection. This comprehensive portrait of host-pathogen dynamics in EVD illuminates new features of pathogenesis and establishes resources to study other emerging pathogens.

## Highlights

• Ebola virus disseminates quickly and widely, likely mediated by infected monocytes
• Emerging viral variation impacts fitness and identifies compartmentalized infections
• Host-transcriptional signatures identify changes in vasculature-related genes
• Temporal deconvolution of bulk transcriptomes improves cell abundance estimates

## Graphical abstract

**Figure:**
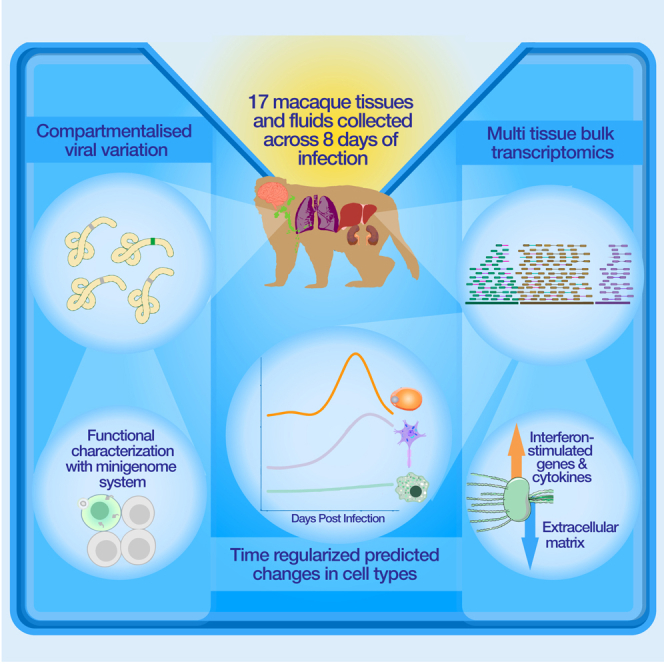

## Teaser

Normandin et al. explored temporal and spatial host-pathogen dynamics in Ebola virus disease through a natural history study in rhesus monkeys. Bulk transcriptomics of over 400 samples highlighted clinically relevant changes in host gene expression and the emergence of viral mutations that were specific to individual tissues.

## Introduction

Ebola virus disease (EVD), caused by infection with Ebola virus (EBOV), is among the most severe infectious diseases, with case fatality rates (CFRs) ranging from 40% to 50% in patients.^1^ Since 1976, over 30 outbreaks of EVD have been recorded, claiming tens of thousands of lives.^2^^,^^3^ While new vaccines^4^ and treatments^5^ are available, CFRs remain high, especially among patients who present late in the disease course.^6^ Recent outbreaks of EVD in the Democratic Republic of the Congo and Uganda and of other filovirus diseases, such as Marburg virus disease, underscore the importance of addressing filovirus threats. EVD is a prototypical viral hemorrhagic fever (VHF) with clinical manifestations including fever, severe gastrointestinal involvement, hemodynamic dysfunction, and multiorgan failure leading to death.^7^ Notably, the host-pathogen determinants of this severity remain relatively obscure, and we lack comprehensive insight into the molecular pathobiology underlying severe EVD.

Genomic technologies let us better understand the molecular basis of infection, but their application has been centered on a few well-studied pathogens. Transcriptomic approaches in particular enable quantification of host transcripts and pathogen sequences, shedding light on relevant host factors, tissue pathologies, cellular targets of infection, and emerging genetic variation.^8^^,^^9^^,^^10^^,^^11^ Comparative analyses of these signals between pathogens and populations can identify pathogen-agnostic and pathogen-specific responses, thereby indicating pathways of potential evolutionary and therapeutic significance.^12^ Despite the important roles genomics and transcriptomics have played in our understanding of diseases, including coronavirus disease 2019 (COVID-19),^8^^,^^9^^,^^10^^,^^11^ many severe viral threats have not been studied as extensively, in particular high-containment pathogens. Thus, there is a need for improved datasets and analytical methods integrating transcriptomics data to build a comprehensive understanding of molecular factors involved in diverse pathologies.

Previous studies of EBOV infection in non-human primate (NHP) models have largely focused on immune-related organs, with limited temporal or spatial resolution and overlooking pathogen dynamics. These studies have found that EVD is characterized by lymphocyte depletion and reduction in platelet counts,^7^ while interferon-stimulated genes (ISGs), pro-inflammatory cytokines, and apoptosis-related genes have been identified as blood biomarkers that predict EVD severity and fatality.^13^^,^^14^^,^^15^ An extended time course further identified early and conserved blood transcriptional responses,^16^ with tissue-specific and temporal-specific gene expression changes observed in some solid tissues.^17^ Single-cell RNA sequencing (scRNA-seq) and protein quantification by mass cytometry (CyTOF) of peripheral immune cells revealed emergency myelopoiesis and suppression of antiviral responses in infected cells.^18^ RNA viruses, including EBOV, have a high mutation rate, allowing better resolution of inter-tissue viral spread and evolution. Emerging variations may allow the virus to better infect and replicate in a host;^19^ biologically meaningful EBOV variants have emerged during animal studies^20^ and recent outbreaks,^21^^,^^22^ and varying levels of evolutionary constraint and adaptive potential have been described across the viral genome.^23^ In patients, these variants are generally identified from blood, which likely reflects only a subset of viral diversity as tissues present different selective pressures.^24^^,^^25^^,^^26^^,^^27^ Determining the shared and specific host dynamics across tissues and associating them with the corresponding viral dynamics promises to yield a more holistic view of disease progression.

Here, we present the first comprehensive spatiotemporal characterization of host and viral dynamics in a key NHP model of severe EVD. This dataset—the largest of its kind for any maximum-containment pathogen—provides novel insights into the establishment and progression of EVD and a rich resource for understanding host-pathogen interactions. To explore this dataset, we developed and applied ternaDecov, a computational tool to infer cell type proportions from bulk RNA-seq datasets with continuous covariates, and demonstrated its broader applicability. This study elucidates global and tissue-specific changes that may contribute to pathogenesis and illuminates potential routes of viral adaptation, circulation, and compartmentalization in peripheral tissues.

## Results

### Multiorgan RNA-seq of rhesus monkeys with EVD shows widespread viral distribution and transcriptional changes

**Figure 1:**
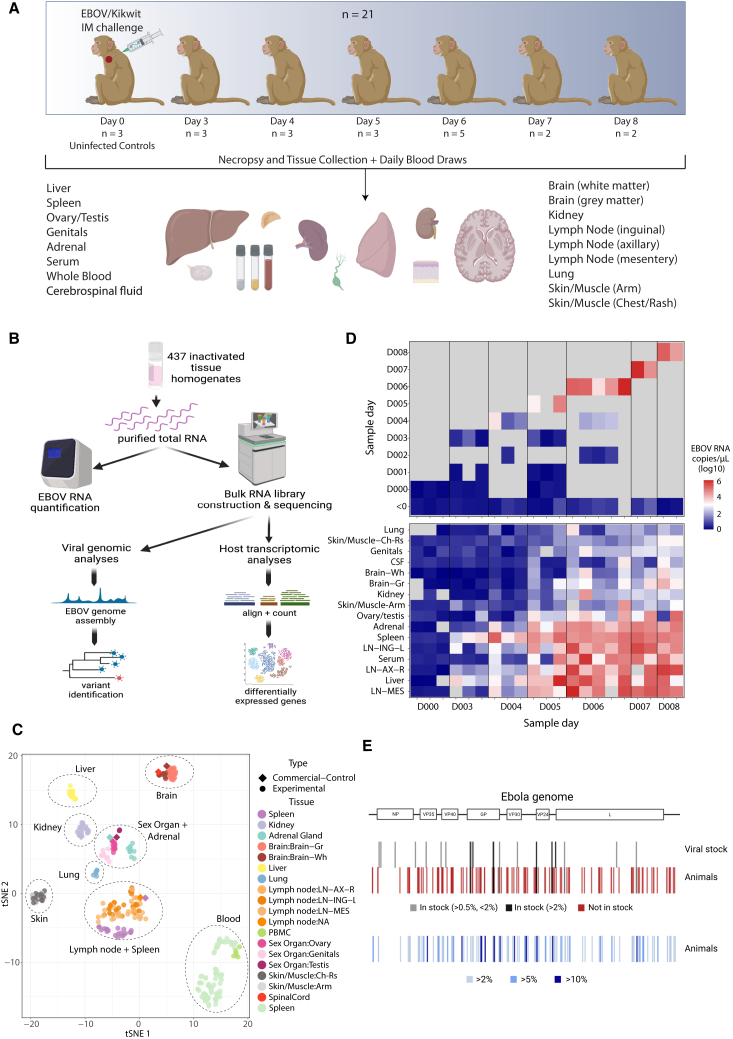
Study overview (A) Description of the animal study and dataset, including the number of animals, time points, and samples collected. (B) Schematization of study design and experimental and analytical workflow. (C) t-distributed stochastic neighbor embedding (tSNE) plot of transcriptional signatures, demonstrating that unique tissues cluster together and with commercial controls of the same type. (D) Viral load across time in whole blood (top) and across tissues and other fluids at necropsy (bottom) for each animal, ordered by time between infection and necropsy. Colors represent viral RNA as log10(copies/μL), as assessed by qRT-PCR; gray represents no data. (E) Viral variants across the EBOV genome identified in infecting viral stock and infected animals. Variants, designated by lines, are colored by their presence in stock (top) and frequency in infected animals (bottom). Images were created with BioRender.

We established an extensive viral genomic and host transcriptomic dataset from a natural history study in 21 NHPs exposed to a lethal dose of EBOV. In this study, described in depth previously,^18^^,^^28^ rhesus monkeys were sacrificed at baseline or 3–8 days post infection (DPI). Over 400 bulk RNA samples were collected at necropsy from 14 solid tissues and 3 tissue fluids (Figure 1A). Additionally, blood draws on alternate days were collected for a subset of animals. We quantified viral load by qRT-PCR and attempted bulk RNA-seq on all samples (Figures 1B and 1C).

We observed high EBOV viral loads across fluids and tissues, indicating widespread viral dissemination (Figure 1D and Table S1). Viral loads were under a detectable threshold across tissues in uninfected animals but ranged from undetected to greater than 10^6^ copies/μL in EBOV-exposed animals and were detectable in all tissues by 6 DPI. Viral loads were generally highest in the blood, serum, liver, lymph nodes, spleen, and adrenal gland. Viral loads in some tissues, such as kidney, skin, ovary/testis, and brain, were high in select animals after 6 DPI by qRT-PCR and sequencing-based viral read counts, which were highly correlated (Figure S1).

We obtained high-quality sequencing data from over 300 samples despite variable RNA quality, likely arising from challenges intrinsic to biosafety level 4 (BSL-4) containment conditions. We employed rigorous filtering and quality control methods to ensure the accuracy of this large dataset (Table S2). Briefly, we removed 13 samples that had insufficient total reads (<0.5 million reads), and eight additional samples that did not match the expected animal or tissue from NHP genotype fingerprinting, chromosome X:Y read ratios, or dimensionality reduction clustering (Figure S2). Host gene expression patterns across the sample set were driven primarily by the tissue identity (Figure 1C), and within each tissue group, host expression clustering patterns were driven by DPI (Figures S3 and S4). We assembled complete EBOV genomes from many tissues and identified variants in samples with high coverage depth (Figure 1E).

### Host-virus analysis, using time-regularized deconvolution, reveals the contribution of direct infection and monocyte infiltration to tissue-specific viral loads and host responses

**Figure 2:**
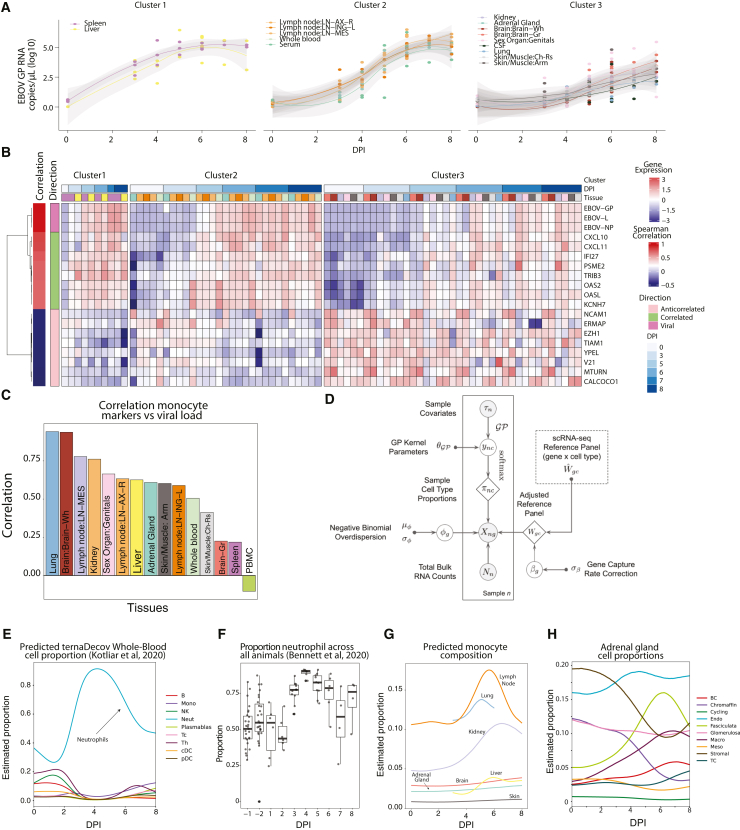
Correlating viral dynamics and host response to infection (A) Viral loads, as determined by qRT-PCR, plotted versus time. The trajectories for different tissues were separated into three distinct patterns using K-means longitudinal data clustering, yielding groups of tissues with similar viral load dynamics. (B) Gene expression across tissues (separated by the clusters in A) for the top 8 correlated and anti-correlated DEGs and 3 representative viral genes. Samples are ordered along the x axis by tissue and DPI. On the y axis, DEGs are clustered and labeled by direction. (C) Correlation between viral load and canonical monocyte marker expression across each tissue. (D) Overview of modular deconvolution framework used in ternaDecov. The output proportions from the models are then used to draw observed sample counts from a negative binomial distribution based on the provided single-cell profiles. (E) Deconvolution of whole blood using scRNA-seq data^18^ confirms the detected increase in neutrophils at 4 DPI. (F) Proportion of neutrophils across samples using Sysmex XT-2000iV automated hematology by flow cytometry^28^. (G) Deconvolution of monocyte composition across time for each tissue based on an scRNA-seq reference of *Macaca fascicularis*. (H) Deconvolution of predicted cell type proportion across time for adrenal glands.

The host and virus data from this study provide a spatiotemporal picture of how EBOV establishes infection and spreads to multiple organ systems. Viral loads increased over time across all tissues, but the rate of increase differed (Figure 2A). Spleen and liver had the sharpest rise in viral load; these tissues were likely the primary sites of infection and replication after intramuscular exposure, putatively seeding infections throughout the body.^14^^,^^29^^,^^30^ Lymph nodes, whole blood, and serum had high terminal viral loads (∼10^5^ copies/μL) but peaked later in infection (Figure 2A); these tissues likely accumulated infected cells. Other tissues (including brain, ovary/testis, skin, lung, kidney, and adrenal) had generally lower peak viral loads (<10^3^ copies/μL) and slower rates of increase in viral RNA burden. In most tissues, we found that several host genes were correlated with viral RNA load. The top genes that correlated with viral load were interferon gamma and alpha ISGs (such as *CXCL10/11*, *IF16*, and *IFI27*) and those thought to be involved in viral defense (*KCNH*, *OASL*, and *OAS2*) (Figure 2B). The top genes anticorrelated with viral load included epigenetic and cell division-related genes, such as a H3K27 methyltransferase (*EZH1*) and a Yippee-like protein (*YPEL*) as well as a cell adhesion protein (*NCAM1*) involved in cell-matrix interactions and expansion of lymphocytes.^31^

We sought to further determine the factors driving differences in viral load across tissues. The viral load of a given tissue is determined by the efficiency with which EBOV infects and spreads within that tissue, the propensity of infected monocytes—the main infected immune cell population *in vivo**^18^*—to infiltrate the tissue during infection, and/or the virus load present in circulating blood. We noted that the expression of canonical monocyte genes demonstrated a trend toward positive correlation with viral load in most tissues (Figure 2C) but not in tissues in which monocytes/monocyte-derived-macrophages are either normally abundant (blood and spleen) or a low viral load is detected (brain). We observed no consistent correlation (correlation < 0.45) between non-monocyte blood cell marker genes and viral load (Figure S5), suggesting that recruitment of infected monocytes is a significant driver of the viral load. This finding led us to investigate the role that intra-tissue changes in cell type proportion may play during pathogenesis.

Despite the availability of several deconvolution methods, which allow inference of cell type composition in bulk RNA-seq samples based on an scRNA-seq reference set,^32^^,^^33^^,^^34^^,^^35^ most approaches are computationally inefficient. Furthermore, existing approaches provide only single-point estimates and do not use continuous covariates (such as time, age, developmental stage, or location) that are common features of large sequencing datasets. To address these limitations, we developed and applied a novel computational method to characterize tissue-specific changes in cell type proportions over the course of disease. We reasoned that continuous processes result in smooth trajectories that can simultaneously improve deconvolution (by sharing information between samples in close temporal proximity) and provide more information about the underlying biological process by inferring a specific parametric form of the cellular change trajectory. In our generalizable model for trajectory-based deconvolution, ternaDecov (temporal RNA deconvolution), the cellular proportions at each data point for every sample are drawn from a continuous function (Figure 2D). The form of the continuous function is not fixed and can be derived from alternative parametric and non-parametric trajectory models (STAR Methods).

We confirmed the accuracy and biological relevance of ternaDecov’s cellular proportion estimates and showed that trajectory models have advantages over individual point estimates made by existing methods. We benchmarked ternaDecov using a published bulk RNA-seq dataset from human pancreatic islets^36^ and an scRNA-seq reference dataset.^37^ We used expression of HbA1C as the covariate for trajectory regularization because levels of this gene are known to be related to changes in cell proportions.^32^ Estimated cell proportions from ternaDecov showed a high correlation with results from an established deconvolution method, MuSiC,^32^ including a negative correlation of β cell abundance with HbA1C levels (Figure S6). To further assess the biological relevance of ternaDecov’s outputs, we used the whole-blood samples in our study. Deconvolution of bulk whole-blood RNA sequencing with ternaDecov identified an increase in the proportion of neutrophils that peaked at 4 DPI (Figure 2E). This peak mirrored the observed increase in neutrophils as measured by fluorescence flow cytometry^28^ (Figure 2F), scRNA-seq (0.2%–65.1% of cells between baseline and late EVD),^18^ and CyTOF (9.3%–49.8%).^18^ Results were again consistent between ternaDecov and MuSiC (Figure S6), but ternaDecov showed faster runtimes. In addition, the trajectory models used by ternaDecov allow inference of unmeasured time points and reduce L1 error of estimates for measured time points (STAR Methods).

We next applied ternaDecov to estimate monocyte infiltration across tissues. For each tissue, we created a joint atlas of tissue-specific cell types and blood cell types (STAR Methods), and deconvolved their blood monocyte, blood non-monocyte, and tissue-specific cell type fractions. The proportion of monocytes/monocyte-derived macrophages varied across tissues, with the highest peak occurring in the lymph nodes following infection. Several tissues—most notably the lymph node, lung, kidney and liver—showed a sharp increase in the proportion of monocytes beginning around 4 DPI (Figure 2G). In contrast, the proportions of other blood cell types remained stable, and this change was not observed in tissues that are large reservoirs of monocytes at baseline (Figures 2E and S6), indicating a specific increase in monocytes in certain tissues and not an increase in circulating blood. This finding suggests that infiltrating monocytes influence the transcriptional signatures observed at this stage of infection. Deconvolution further illuminated changes in tissue-specific cell types during infection (Figure S6), such as the decrease of chromaffin cells in the adrenal gland (Figure 2H), a cell type that is infected during EVD.^38^ Chromaffin cells produce epinephrine, an essential hormone for the host response to infection, whose depletion could be associated with severe disease.

### A tissue atlas illuminates the spatiotemporal dynamics of interferon and cytokines during EVD

**Figure 3:**
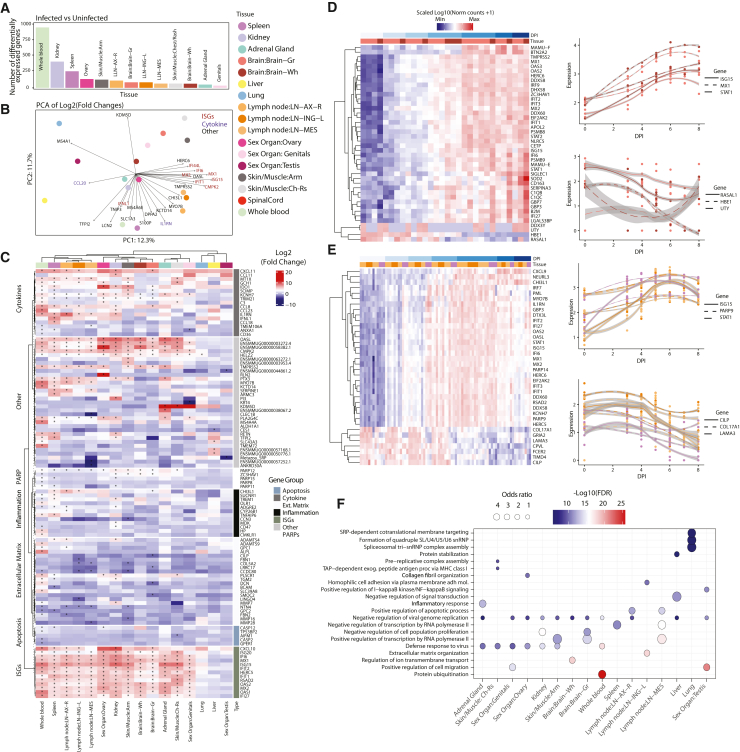
Host transcriptomics across tissues and time (A) Number of DEGs between non-infected and infected samples; tissues with more than 5 DEGs are shown in the plot. (B) PCA of log2 fold changes of significantly DEGs between infected and uninfected samples. Top contributing genes for PC1 and PC2 are highlighted. (C) Heatmap of fold-changes of top DEGs across tissues, stratified by meaningful gene categories; stars marks significant differential expression (FDR < 0.05). (D) Left: heatmap of genes changing significantly across time for brain. Right: gene expression changes across time for selected genes. Colors atop plots designate gray (light red) and white matter (dark red). (E) Same as (D) but for lymph nodes (shades of orange) and spleen (purple); colors atop plots designate tissues. (F) Gene Ontology (GO) term analysis of genes differentially expressed (top 100 FDR < 0.01) across time as determined by ImpulseDE2. Enriched terms were determined per tissue, and the top 3 GO terms, as determined by Kolmogorov-Smirnov (KS) test, per tissue were selected for display. Colors of circles correspond to −log10(KS pval) of the enriched term within tissue, and sizes of circles correspond to odds ratio.

To further discover molecular signatures of infection, we identified genes whose expression changed upon infection in at least one tissue or fluid. We identified differentially expressed genes (DEGs) between infected and non-infected samples (DPI ≤ 0) independently for every tissue (false discovery rate [FDR] < 0.05 and log2 fold change (FC) > 2), resulting in the identification of between 35 and 974 DEGs per tissue (Figure 3A; Table S3). To avoid tissue sampling effects, we excluded tissue marker genes when interpreting genes across tissues (Figure S7; STAR Methods). Principal component analysis (PCA) using the log2 FCs of DEGs showed separation of tissues, indicating tissue-specific differences in response to infection (Figure 3B). Interestingly, the primary axis of variation (PC1; 12.3% variance explained) across tissues is driven by several genes related to the interferon response (Figure 3B).

We confirmed the key role of interferons and cytokines in the host response during EVD across tissues. Past studies have shown that expression of genes associated with the type I interferon response generally increases in blood and several tissues during EVD.^14^^,^^17^^,^^39^^,^^40^^,^^41^ Similarly, we found that interferon and related genes were upregulated in EVD and demonstrate that this trend is recapitulated in our extensive set of 15 distinct tissues (Figures 3C and S4). We observe a similar increase in some cytokine genes, especially in the whole blood, spleen, and skin (Figure 3C). These responses are common to viral infections in general, and their increased expression across multiple tissues is present in the well-established clinical manifestation of “cytokine storm/cytokine release syndrome,” which occurs during EVD.^42^^,^^43^

While these genes were upregulated across distinct tissues, the degree and temporal dynamics of this upregulation differed. Indeed, although many of these genes were globally upregulated across tissues, they were also represented as the top genes driving the separation of tissues, underscoring the distinct dynamic profiles (Figure 3B). To further explore differences in the interferon and cytokine response across tissues, we examined DEGs changing over time in each tissue. Among these genes globally upregulated in response to infection, ISGs and cytokines had different dynamics between tissues across time, with an early increase in spleen, lymph nodes, liver, and whole blood and a delayed increase in secondary organs such as the brain (Figures 3D and S8). This indicates a broadly conserved interferon and cytokine response across tissues, albeit with distinct dynamics likely associated with the circulation of the virus and recruited immune cells during pathogenesis.

### Tissue-specific transcription profiles reveal novel genes and pathways dysregulated in EVD

We uncovered novel transcriptional signatures of disease, identifying differences in the host responses across tissues and inter-tissue heterogeneity (Figures 3D, 3E, and S8). Among the DEGs with the greatest fold change in each tissue, several genes were differentially expressed in only a subset of tissues. For example, we observed changes in apoptosis- and inflammation-related genes particularly in the whole blood and kidneys. We also noted increased expression of PARP-family genes (*PARP12*, *ZC3HAV1*, *PARP15*, *PARP6*, and *PARP11*) in kidney and skin (Figure 3C). Members of the PARP family are responsible for functions including DNA repair and chaperoning^44^^,^^45^ and can have pro-viral effects. For instance, PARP11 acts as a pro-viral factor in vesicular stomatitis virus infection by inhibiting the strength of interferon (IFN)-I-activated signaling.^46^ It is possible, therefore, that the PARP family may contribute to pathogenesis during EVD.

To nominate underlying pathogenic processes of EVD that might be indicated by DEGs, we used Gene Ontology enrichment analysis to interpret tissue-conserved and tissue-specific signals. We identified common pathways enriched across tissues during infection, including “negative regulation of viral genome replication” and “defense response to virus” (Figure 3F). These pathways likely represent an enrichment of general antiviral defense genes common to all tissues, including genes related to the conserved IFN and cytokine responses we identified previously. Additionally, we identified enriched tissue-specific pathways, including cell migration, matrix formation, and organization (Figure 3F). These pathways suggest differential remodeling of tissues as a driver or consequence of EVD progression.

We observed significant changes in expression of genes encoding tissue connectivity- and extracellular matrix (ECM)-related proteins. Specifically, we saw a significant decrease in expression over time for tissue connectivity-related genes such as laminin, cartilage, and collagen (*CILP*, *LAMA3*, and *COL17A1*) in lymph nodes and spleen (Figures 3E and S9). These genes have not been reported as molecular signatures of disease but are consistent with the histological changes in vascular structure and function observed during EVD.^42^ We observed similar changes in ECM-related genes in other organs, specifically in skin/muscle samples, as well as an increase in the expression of genes encoding metallopeptidases proteins (*MMP2*, *MMP3*, and *MMP8*) in the skin, brain, and whole blood (Figure S9). These results suggest that onset of multiorgan failure, increase in vascular permeability, and internal bleeding associated with EVD may be related to weakening of tissue connectivity associated with a downregulation of ECM genes, in addition to the known increase of tissue factor (F3) in the blood^30^ (Figure S9).

### Viral variants reveal patterns of compartmentalization and circulation among tissues

Given the high viral loads in several tissues in this study and the promiscuous tropism of EBOV,^47^ we sought to elucidate how the virus spreads *in vivo* using viral variants that emerge over infection. We attempted viral genome assembly on all sequenced samples and obtained complete (>95% unambiguous nucleotides) viral genomes from 95 samples for further comparisons. Among all complete genomes, there was a single consensus-level (>50% variant frequency) mutation. The variant, which fell at position 10,343 (in the viral protein 24 [VP24] 5′ UTR), was detected in the sex organ of an animal sacrificed 6 DPI. The lack of consensus-level variants was expected, given the short duration of infection and absence of specific selective pressure. We also profiled minor variants in 45 samples that had sufficient viral coverage (>400x mean depth) (Figure S10; Table S4). Across the sample set, minor variants ranged from 2%–22% frequency and fell at a total of 111 unique nucleotide positions. Of these 111 variants, 5 variants were present in the infecting stock at more than 2% frequency, and an additional 3 variants were present at a more conservative threshold of 0.5% frequency (Figure 1E). To focus our analysis only on variants that arose within animals, we filtered out these 8 variants, leaving variants at 103 nucleotide positions for further study.

**Figure 4:**
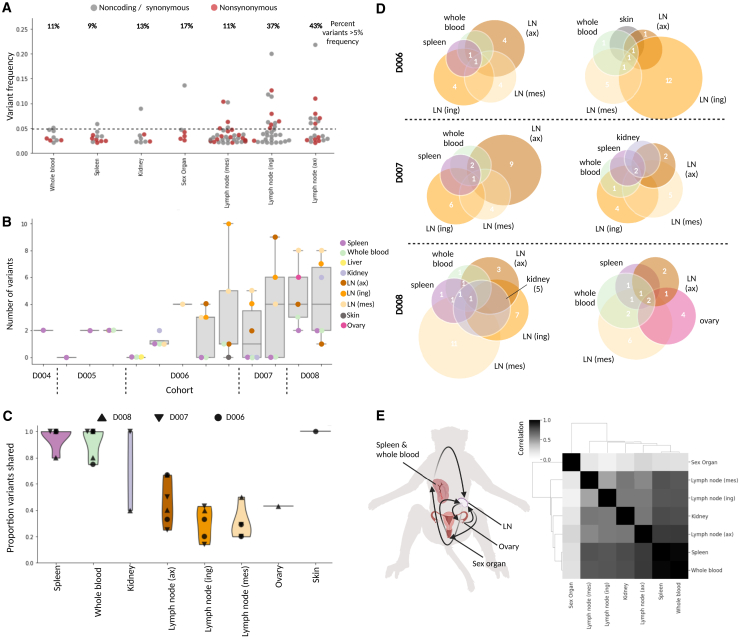
Minor viral variants show compartmentalization and circulation (A) Frequencies of all nonsynonymous (red) and synonymous/noncoding (gray) variants that emerged during infection, plotted and separated by tissue; the percentage of variants above 5% frequency (dotted line) is given above each tissue. (B) For each animal (ordered by DPI), the number of variants that emerged in every tissue (samples with >400× mean viral coverage). (C) Violin plot showing the proportion of shared viral variants, separated by tissue; each point represents a unique animal, and symbols demonstrate DPI. (D) Schematic representing variants that are shared (numbers displayed in overlapping circles) and not shared (numbers displayed in non-overlapping circles) in all tissues available for 6 animals (2 of each the D6, D7, and D8 cohorts). (E) Left: schematic of viral circulation among tissues, based on the variant profiles (image created with BioRender). Right: a Spearman correlation of different tissues’ variant profiles, concatenated across animals.

We first assessed global patterns in the number and frequency of variants in different tissues. We analyzed all samples available but specifically focused on whole blood, spleen, and the three distinct lymph nodes because high-coverage viral genomes were available for many animals in each of these tissues. The lymph nodes had a large number of variants that emerged within animals with high frequency; 37% of variants in the inguinal lymph node and 43% of variants in the axial lymph node had more than 5% frequency (Figure 4A). The number of variants was also consistently high in the lymph node samples across animals but with variable DPI (Figure 4B). Conversely, spleen and whole blood consistently had the fewest variants detected across animals (Figure 4B). We observe that, compared with spleen and whole blood, lymph nodes harbor more variants, and these variants also tend to be observed at higher frequencies. We find an apparent skew in the ratio of nonsynonymous to synonymous mutations in high-frequency (>5%) vs. low-frequency (<5%) variants in the inguinal lymph nodes by permutation test (5 vs. 0.11 in inguinal, p = 0.006; 1 vs. 1.36 in mesenteric, p = 0.58; 1.3 vs. 1.7 in axial, p = 0.43), suggesting that selective pressure may contribute to differences in variant frequencies between tissues.

We probed further to investigate the cause of the higher viral population diversity observed in the lymph nodes compared with that of the whole blood and spleen. For the 6 animals (2 animals from each of the 6-, 7-, and 8-DPI cohorts), we assessed the overlap of all variants observed across tissues. Globally, we found that samples from each of the three lymph nodes had several variants that were unique to that tissue, while spleen and whole blood variants were almost always shared with at least one other tissue (Figure 4C). In fact, many of the variants identified in the whole blood and spleen samples were identified in every other tissue profiled (Figure 4D). Generally, we observed a high degree of similarity between variant profiles in the whole blood and spleen and more similarity between these two tissues and each lymph node than among the lymph nodes (Figure 4D).

To investigate the source of viral diversity in the lymph nodes, we considered all tissues, noting that the sex organ samples have variant profiles that are most distinct from other tissues. For example, in the animal with a consensus-level (>50% frequency) variant, we found that there were multiple high-frequency variants in the sex organ and ovary samples, which were at an elevated frequency in the mesenteric lymph node sample, but were not detected or at low frequency (<5%) in any other sample from that individual. Previous studies have suggested that infection can be compartmentalized to the sex organs and ovaries.^48^^,^^49^ Our data more directly confirm the occurrence of compartmentalized infections in these tissues. The variants rising to high frequency in these sites were likely spread to the more proximal mesenteric lymph node (Figure 4E). This hypothesis may be generalized to explain why lymph nodes harbor many high-frequency, unshared variants; they likely traffic between a subset of peripheral tissues with high-frequency variants that have emerged in compartmentalized infections.

### Viral variants and functional analysis suggest adaptation during EBOV infection

The viral variants that emerged over the course of infection can also help us understand viral evolution and dynamics. Emergent variants may positively or negatively impact virus biology, including altering tropism, infectivity, and escape potential.^20^^,^^50^

**Figure 5:**
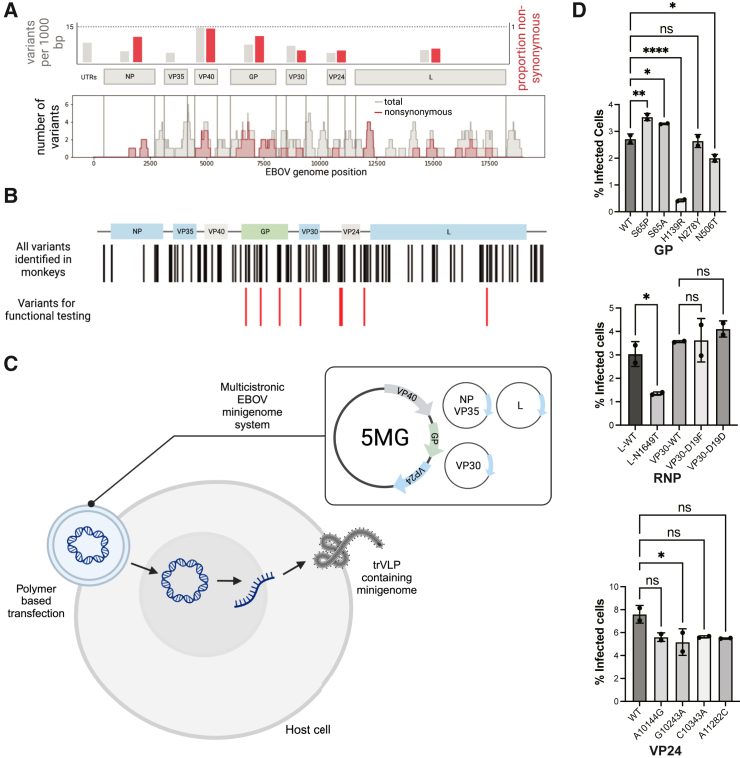
Viral adaptation and fitness effects (A) Top: number of emergent variants per 1,000 bp (gray) were quantified for each gene-coding region as well as proportion of nonsynonymous variants (red). Bottom: accumulation of total (gray) and nonsynonymous (red) variants in specific gene regions was quantified using a sliding window of 200 bp. (B) Genomic locations of variants selected for further functional testing (red) among all variants identified across the EBOV genome (black). (C) Schematic of the EBOV/Kikwit transcription- and replication-competent virus-like particle (trVLP) minigenome system that recapitulates the wild-type and variant viral life cycle in a host cell (image created with BioRender). (D) Flow cytometry analysis of the percentage of GFP+ cells 48 h post minigenome transfection as a percentage of infected host cells by seed stock (wild type [WT]) or viral variants in GP, RNP, and VP24. Error bars represent standard deviation.

We examined the distribution and types of emerging mutations across the viral genome. UTRs showed a higher number of variants per 1,000 bp than coding regions (8.1 versus 5.9), consistent with findings of intra-host diversity in human cases.^23^ Among genes, we observed the highest number of mutations per 1,000 bp in VP40 (14.3), which is involved in virion assembly and immune evasion,^51^ and glycoprotein (GP) (6.9), which is immunogenic and critical for infectivity^52^ (Figure 5A). VP40 and GP also had the highest proportions of nonsynonymous variants. We observed narrower regions of other genes that, with high proportions of nonsynonymous variants, including the C-terminal end of the nucleoprotein (NP) and N-terminal end of the viral polymerase (L), which are each part of the ribonucleoprotein (RNP) complex that performs viral replication and transcription (Figure 5A). We find evidence of negative selection in the L gene by binomial test (p = 2.6 × 10^−5^) but no evidence of ratio skew in VP40, GP, or NP (respective p values of 0.24, 0.53, and 0.13). Across the genome, A-to-G and T-to-C mutations were more frequent than G-to-A or C-to-T mutations, with a particularly high proportion of these mutations in two specific animals (Figure S11). We did not observe clear tissue-specific trends in variant location or type (Figure S12).

We adapted a well-established transcription- and replication-competent virus-like particle (trVLP) minigenome system^53^ to assess the functional effects of eight coding mutations (in GP, L, and VP30) and four non coding mutation (in the UTR of VP24) across the complete viral life cycle (Figure 5B). This system allows the study of EBOV genes outside of BSL-4 laboratories by separating the RNP complex into three separate plasmids (L, NP-P2A-VP35, and VP30) that drive replication of a T7-driven minigenome composed of reporter genes and the remaining three EBOV genes (VP40, GP, and VP24) (Figure 5C). We recloned the entire system to encode the EBOV/Kikwit backbone, as established previously trVLP systems encoded EBOV genes from variants that diverge in sequence from Kikwit by hundreds of nucleotides. Co-transfection of all four plasmids into mammalian host cells results in transcription and replication of the multicistronic minigenome, including a fluorescent marker, which we detected by flow cytometry. These cells also produce GP-coated trVLPs, which can infect any target cell that expresses the viral RNP complex. For testing, we prioritized variants that emerged in multiple animals or rose to high frequency or changed in frequency relative to the infecting stock and were in genes or regions likely to be important for viral fitness (Figure 5B).

Because mutations in viral glycoproteins are often under selection, we prioritized these variants for functional effects. Of the five GP variants we tested, four had a significant effect on viral fitness (Figures 5D and S13). Consistent with the role GP plays during viral entry, additional testing with a GP-pseudotyping assay that specifically models this step suggests that this fitness difference is likely due to a difference in productive host-receptor interactions (Figure S13). The convergent mutations at amino acid position 65 (S65A and S65P) resulted in an increase in infectivity. Notably, a mutation at this position was present in viral sequences from a human case (GenBank: MH121168.1) and has been shown previously to be important for establishing mouse-adapted variants of EBOV/Mayinga and EBOV/Makona,^54^^,^^55^^,^^56^ further supporting a key role played by this position. On the other hand, the variants H139R and N506T resulted in a significant loss of infectivity. Interestingly, a published crystal structure of GP bound to the human receptor NPC1 showed that H139R is proximal to this interaction,^57^ and the region surrounding N506T is the binding site of the neutralizing antibody KZ52, derived from a human survivor of the 1995 Kikwit outbreak.^58^

Next, we leveraged our ability to simulate the full viral life cycle with the trVLP minigenome system to study mutations in genes that impact transcription and replication. Functionally relevant mutations have emerged during human outbreaks of EBOV in genes involved in viral replication and transcription as well as in regulatory regions.^22^^,^^23^^,^^59^^,^^60^ Of the four VP24 UTR variants we tested, only G10243A showed a slight impact on viral fitness, potentially because of the more subtle ways in which UTR variants could affect viral fitness, which are outside the limit of detection for this system. Among the three variants we tested in the RNP complex, we found that mutations in VP30 showed no significant effect on viral fitness; however, a mutation (N1649T) on the viral polymerase (L) has a significant effect on viral fitness (Figures 5D and S13). N1649T is located in the predicted MTase domain of the viral RNA dependent RNA polymerase (RdRp)^61^ and decreased viral fitness. Despite recent elucidation of the complete RdRp structure,^61^ the MTase domain has yet to be experimentally resolved. Our results suggest that it might play a role in maintaining viral fitness, warranting further studies of its structure and function.

## Discussion

Here, we apply high-depth, unbiased sequencing, complemented by newly established experimental and computational approaches, to a large natural history study in rhesus monkeys to provide insights into the molecular basis of disease. We describe detectable levels of EBOV RNA in most tissues, with the earliest infection in the liver and spleen and particularly high viral loads in the blood, lymph nodes, and adrenals, consistent with previous reports of tropism and pathology.^47^^,^^49^^,^^62^^,^^63^^,^^64^^,^^65^^,^^66^ By following these dynamics over time, we can further observe how infection drives disease progression and virus adaptation. Together, these perspectives show widespread, systemic changes during acute disease.

Emerging variants at over 100 positions across the viral genome illuminated potential sites of adaptation and compartmentalization during acute infection. Shared patterns of minor variants suggest a model where the spleen and blood spread virus systemically, likely mediated by recruitment of infected monocytes, while the lymph nodes traffic virus among locally compartmentalized infections. Compartmentalized infections in EVD, particularly in immune-privileged sites like the reproductive tract, could promote persistent infection and sustained evolution and pose a risk for reactivation and onward transmission.^67^ Using genomic data, we show that, after viral dissemination in EBOV-exposed NHPs,^48^^,^^68^^,^^69^ viral populations are actively maintained and compartmentalized in these tissues, distinct from infection in other organs. Several features of this emerging viral variation, including a higher frequency of T-to-C mutations, have been observed in human outbreaks^23^^,^^70^^,^^71^ and in response to therapeutic agents.^20^ The higher frequency of T-to-C and A-to-G mutations relative to G-to-A mutations may suggest host RNA editing activity, and past studies indicate that T-to-C mutations are clustered in specific regions.^70^ In contrast, VP40, which here had the highest frequency of nonsynonymous mutations (Figure 5A), has been suggested previously to be strongly conserved in human outbreaks.^23^ The differences in the distribution of mutations across some viral genes may reflect rapid initial adaptation of the virus, similar to that seen immediately after zoonotic spillover. The number of unique viral variants we detect in tissues highlights the importance of animal models for providing insights into selective pressures in different compartments.

Of the 12 variants we tested in our minigenome system, six were found to significantly alter viral fitness, with the majority of these (4 of 6) falling in the GP gene, indicating viral entry as a mechanism. Half of the variants we tested did not have any observed impact on viral fitness. This is unsurprising because variants could have increased in frequency by chance because of genetic drift, further highlighting the importance of experimental assays that can rapidly and easily screen for functional effects of mutations. The filovirus GP, RdRp, and RNP complexes have long been considered promising targets for broad antiviral therapy.^72^^,^^73^^,^^74^^,^^75^^,^^76^ Although further mechanistic and structural studies are needed to determine the impact the emerging mutations detected in this study have on viral fitness, our results support the potential of trVLPs to uncover novel mutations that affect viral entry, replication, and infection, which could guide future rational design approaches in drug discovery.

Our analysis of host transcriptional responses across tissues adds further dynamic and tissue-specific context to known features of pathogenesis and identifies intriguing novel responses related to tissue connectivity. Beyond expected changes in ISG and cytokine expression,^14^ the comprehensive nature of our dataset enabled us to identify differential dynamics across tissues. This study also revealed previously unknown features of disease. We observed changes in ECM genes in most tissues, with widespread dysregulation of collagen-, laminin-, and cartilage-related gene families in several tissues as well as an increase in collagen cleaving enzymes such as metallopeptidase (*MMP8*, *MMP3*, and *MMP2*) in the blood, skin, and brain. These findings provide new molecular insight into the etiology of vascular endothelial and connective tissue disruption (i.e., vascular leak syndromes, characteristic of severe EVD) and may suggest molecular pathobiology common to other hemorrhagic fevers; for example, similar dysregulations in ECM have been reported in other hemorrhagic fevers, such as dengue virus infection,^77^ and ECM cleaving enzymes play a key role in venom-induced hemorrhage.^78^ Interestingly, these enzymes have also been reported to play a role in cell-to-cell viral transmission in West Nile virus^79^ and influenza virus,^80^ warranting further investigation into the roles of these genes in EVD.

Characterizing host and pathogen dynamics in this large serial sacrifice study required establishing new computational and experimental tools that we believe will be of broad use in future studies. ternaDecov fills a key gap among available deconvolution tools^32^^,^^33^^,^^34^^,^^35^ when time-series bulk RNA-seq data are available. By incorporating time as a variable in its deconvolution model of bulk data from a single-cell reference, ternaDecov better models gene expression dynamics. While studying changes over the course of infection was our primary motivation in developing ternaDecov, any continuous covariates can be used, demonstrating the broader applicability of this method. Similarly, existing trVLP minigenome systems were not adapted to the EBOV variant used in this and many other animal studies of EVD. TrVLP minigenomes are powerful systems because they allow the full viral life cycle to be modeled at lower levels of biosafety containment and have been used previously to functionally characterize mutations in other EBOV variants.^22^^,^^53^ Because the EBOV Kikwit variant is recognized as the standard challenge virus for testing clinical countermeasures in animal studies, we believe that the EBOV/Kikwit trVLP system we adapted will be a valuable community resource for future assessment of emerging mutations.

Through this study, we add further spatial and temporal granularity to known signatures of EVD while also suggesting new molecular drivers of pathogenesis. We illustrate relationships between host and viral signatures during EVD and propose potential mechanisms that may generate these signatures. Finally, we provide computational and experimental tools to not only facilitate further investigations of EBOV infections but also provide a model for future studies seeking to nominate and validate molecular bases of disease progression.

### Limitations of the study

The major limitations of this study arise from the constraints inherent to working in maximum containment, and there are several areas where the study could be expanded to increase the breadth and depth of characterization. In particular, many liver samples had low RNA quality, restricting the insights we could obtain for this tissue. The liver harbors many enzymes that degrade RNA, and degradation was likely exacerbated by the constraints of working in maximum containment. Improved preservation methods as well as even broader sampling of clinically relevant tissues, such as the gastrointestinal tract,^81^^,^^82^ would be of interest for future investigations. Additionally, the timing of host transcriptional changes suggests that the recruitment of infected circulating monocytes is a major contributing factor to the spread of the virus to secondary organs. Future studies using scRNA-seq on tissue samples would allow changes in cell type proportions and the impact of infection on specific cell types to be measured more directly, as shown previously in peripheral blood mononuclear cells from this study.^18^ Finally, uniformly lethal animal models like the one used here restrict the study of persistence, acute recovery, and long-term effects of the infection. New experimental challenge models with different routes of inoculation and heterogeneity in outcomes could enable a better understanding of these features in surviving NHPs.

## STAR★Methods

### Key resources table

**Table:** 

REAGENT or RESOURCE	SOURCE	IDENTIFIER
**Bacterial and virus strains**		

Ebola virus/H. sapiens-tc/COD/1995/Kikwit-9510621 (EBOV/Kikwit; GenBank accession MG572235.1; *Filoviridae*: *Zaire ebolavirus*)	BEI Resources	Cat#NR-50306

**Biological samples**		

Monkey Adrenal Total RNA, Rhesus	Zyagen	UR-501
Monkey Brain Total RNA, Rhesus	Zyagen	UR-201
Monkey Kidney Total RNA, Rhesus	Zyagen	UR-901
Monkey Liver Total RNA, Rhesus	Zyagen	UR-314
Monkey Lymph nodes Total RNA, Rhesus	Zyagen	UR-703
Monkey Skin Total RNA Total RNA, Rhesus	Zyagen	UR-101
Monkey Spinal cord Total RNA, Rhesus	Zyagen	UR-230
Monkey Spleen Total RNA, Rhesus	Zyagen	UR-701

**Chemicals, peptides, and recombinant proteins**		

X-tremeGENE 9 DNA Transfection Reagent	Sigma-Aldrich	6365787001
Actinomycin D	Millipore Sigma	A1410-2MG
2′-Deoxyuridine 5′-triphosphate sodium salt (dUTP)	Millipore Sigma	D0184-25UMO
NEBNext Ultra II End Repair/dA-Tailing Module	NEB	E7546L
Instant Sticky-end Ligase Master Mix	NEB	M0370L
Thermolabile USER II Enzyme	NEB	M5508L

**Critical commercial assays**		

Q5 Site-Directed Mutagenesis Kit	New England Biolabs	E0554S

**Deposited data**		

EBOV NHP infection RNA-Seq reads	This study	GSE226106
*Macaca fascicularis* single-cell reference data	^83^	https://db.cngb.org/nhpca/download
RNA-seq data for healthy and diseased pancreatic islet samples	^36^	GSE50244
pancreatic islets scRNA-seq RNA-seq data	^37^	E-MTAB-5061
Peripheral blood data from the same EBOV-infected rhesus monkeys	^18^	GSE158390

**Experimental models: Cell lines**		

HEK293T	ATCC	CRL-3216
U2OS	ATCC	HTB-96

**Oligonucleotides**		

See Table S5		N/A

**Recombinant DNA**		

See Table S5		N/A

**Software and algorithms**		

Bulk RNA-seq Processing	This study	https://github.com/broadinstitute/EbolaNaturalHistory/
ternaDecov	This study	https://doi.org/10.5281/zenodo.8411808
STAR	^84^	https://github.com/alexdobin/STAR
python	Python core team	https://www.python.org/
R	R Core Team	https://www.r-project.org/
UMI-tools	^85^	https://github.com/CGATOxford/UMI-tools
BioMart	^86^	https://github.com/grimbough/biomaRt
viral-ngs	https://viral-ngs.readthedocs.io/en/latest/index.html	https://github.com/broadinstitute/viral-ngs
DESeq2	^87^	https://bioconductor.org/packages/release/bioc/html/DESeq2.html
go.db.df	^88^	https://bioconductor.org/packages/release/data/annotation/html/GO.db.html
topGO	^89^	https://bioconductor.org/packages/release/bioc/html/topGO.html
ImpulseDE2	^90^	https://github.com/YosefLab/ImpulseDE2
MuSiC	^32^	https://xuranw.github.io/MuSiC/articles/MuSiC.html

### Resource availability

#### Lead contact

Further information and requests for resources and reagents should be directed to Katherine Siddle (katherine_siddle@brown.edu).

#### Materials availability

Plasmids generated in this study are available upon request.

#### Data and code availability

The RNA-Seq datasets reported in this paper are available in GEO under accession GSE226106. The scripts used in this study are available at https://github.com/broadinstitute/temporal-rna-seq-deconvolution/and https://github.com/broadinstitute/EbolaNaturalHistory/. The version of ternaDecov used in this study is available at https://doi.org/10.5281/zenodo.8411808.

### Experimental model and subject details

This study included a subset (21 of 27) outbred rhesus monkeys (*Macaca mulatta*) of Chinese origin described recently,^18^^,^^28^ balancing age, weight, and sex (8 males and 13 females). All work was approved and performed in accordance with the Guide for the Care and Use of Laboratory Animals of the National Institute of Health, the Office of Animal Welfare, and the US Department of Agriculture.

HEK293 (human [Homo sapiens] fetal kidney) and U2OS (human [Homo sapiens] osteosarcoma) were obtained from the ATCC (https://www.atcc.org/). Cells were maintained in DMEM containing 10% fetal bovine serum, 1% non-essential amino acids, 1% sodium pyruvate, and 1% penicillin-streptomycin at 37°C with 5% CO2 and seeded onto coated plates for transfection experiments described in details below.

### Method details

#### Natural history study

The details regarding the infecting viral stock and animals used have been published previously.^28^ Briefly, 18 rhesus monkeys were inoculated intramuscularly with 1 mL of 1000 plaque-forming units/mL EBOV/Kikwit (Ebola virus/Homo sapiens-terminal control-COD/1995/Kikwit-9510621 from BEI Resources, Manassas, VA) in the left lateral triceps muscle at study day 0. Animals were humanely euthanized at either a predetermined time point (3 animals on each of days 3, 4, 5 and 6 post-infection) or at terminal endpoint (N = 6). Sequential blood draws under general anesthetic were collected for the 6 animals in the terminal endpoint group. Three uninfected control monkeys (2 female, 1 male) were sham-exposed with 1 mL phosphate-buffered saline at the same anatomic location before sacrifice on day 0. Baseline blood draws at approximately 30 and 14 days prior to infection were collected for all 21 animals. Tissue samples were collected from each animal at necropsy in bead beater tubes and homogenized in TRIzol and inactivated in TRIzol LS.

All monkeys used in this research project were cared for and used humanely according to the following policies: the U.S. Public Health Service Policy on Humane Care and Use of Animals (2000); NIH’s Guide for the Care and Use of Laboratory Animals; and the U.S. Government Principles for Utilization and Care of Vertebrate Animals Used in Testing, Research, and Training (1985). All National Institute of Allergy and Infectious Diseases Integrated Research Facility animal facilities and programs are accredited by the Association for Assessment and Accreditation of Laboratory Animal Care International. This study was performed in the Biosafety Level 4 Laboratory at the NIH/National Institute of Allergy and Infectious Diseases, Integrated Research Facility at Fort Detrick (Fredrick, MD).

#### Sample extraction and RNA purification

Tissue homogenates inactivated in TRIzol were phase-separated with chloroform at the Broad Institute, and total RNA was extracted from the aqueous phase using the MagMAX MirVana total RNA kit (ThermoFisher) on a KingFisher FLEX instrument. DNA was removed by TURBO DNase treatment following RNA extraction. A TRIzol-inactivated aliquot of the viral seed stock injected into animals from this study was also obtained and extracted with the Direct-zol-96 MagBead RNA (Zymo Research).

#### Quantification of viral RNA

Ebola viral load in all extracted RNA samples was measured by qRT-PCR using an SYBR Green assay with previously published primers targeting the EBOV NP gene.^83^ A standard curve of a DNA gBlock (IDT) encoding the target region was used to calculate viral copy numbers. Curves of temporal change in viral load in each tissue were clustered using iterative K-means longitudinal data clustering with the R package KLM with maximum number of NA tolerates per trajectory of 1.

#### Library construction and sequencing

We depleted ribosomal RNA from purified RNA using an RNase H-based approach,^84^ then performed strand-specific ligation-based library construction.^85^ Briefly, we heat-fragmented RNA, performed reverse transcription, labeled second-strand cDNA with dUTP, then ligated xGen UDI-UMI adapters^86^ at a concentration of 0.04 μM for fluid samples and viral seed stock, and 0.2 μM for tissue samples. We then USER-digested the dUTP-labeled strand, and PCR amplified libraries. Libraries were quantified with TapeStation high-sensitivity DNA assay (Agilent). Samples were pooled at equimolar ratios and sequenced on a NovaSeq SP (Illumina) with 2x146bp cycles for the cDNA and 17 cycles of Index Read 1 to sequence the 9-base UMI.

#### Pentacistronic minigenome assay

We constructed a EBOV/Kikwit pentacistronic (5MG) minigenome system based on a previously published EBOV/Mak-C15 tetracistronic (4MG) minigenome system^22^ but cloned in EBOV/Kikwit sequences either amplified by RT-PCR from viral seed stock or ordered as dsDNA gBlocks (IDT) to replace EBOV/Mak-C15 genes. The EBOV/Kikwit 5MG plasmid includes eGFP and nano luciferase as reporter genes and VP40, GP, and VP24 CDS and UTRs. EBOV/Kikwit L and VP30 were cloned into pcDNA3.4 vectors to facilitate site directed mutagenesis (SDM) experiments as pCAGGs vectors from the published system have GC-rich regions that are difficult to amplify under standard PCR conditions. SDM was performed to create single nucleotide variants following manufacturer’s protocol (NEB) with custom designed primers (Table S5). Full plasmid sequences are in Data S1.

We followed an existing protocol for the multicistronic minigenome assay^53^ with some modifications. We seeded HEK 293T cells into collagen-coated 24-well plates, grew to 60% confluency, and transfected cells following the xtremegene9 transfection protocol with the previously described plasmid ratio (31.25 ng of NP-P2A-VP35, 18.75 ng of VP30, 250 ng of L, 62.5 ng of 5MG plasmid encoding eGFP, 62.5ng of T7pol). We harvested cells 48 h post-transfection with trypsin, washed once with PBS and stained with DAPI for cell viability. We then measured the percentage of eGFP positive live cells for each condition which we considered as infected host cells.

#### GP-pseudotyped lentivirus and infectivity assays

The following mutants were selected for a GP-pseudotyping assay: S65A, S65P, H139R, N278Y, and N506T. A gBlock for the EBOV GP seed stock (GenBank: KU182908.1) was designed and synthesized (IDT) with a deleted mucin like domain from amino acid positions 309–489 and an additional adenosine at nucleotide position 890 to produce the full length glycoprotein.^21^^,^^87^^,^^88^ This gBlock was cloned into the pGL4.23 backbone expression plasmid described in Diehl et al. using restriction enzymes with the GP sequence placed under the control of a cytomegalovirus immediate-early (CMV IE) promoter/enhancer.^21^ Q5 Site directed mutagenesis (NEB) was used to introduce the mutations in the backbone.

GP-pseudotyped lentiviral virions carrying an EFS driven H2B-mCherry reporter gene were produced in triplicate by transfecting HEK293FT cells (Takara, Cat# 632180) using polyethylenimine (PEI, Polysciences Cat# 24765–1) with 800 ng GP envelope, 866 ng psPAX2, and 1,333 ng H2B-mCherry reporter plasmid. Media was exchanged 4 h after transfection and viral supernatants were collected 2 days later. The viral supernatant was filtered through a 0.4μm filter (Pall, Cat# 8129), treated with Benzonase-nuclease (Sigma-Aldrich, Cat# E1014-25KU) for 1 h at 37°C after which viral RNA was extracted using a Zymo RNA extraction kit according to manufacturers protocols (Zymo, Cat# R1041). An qRT-PCR was run to determine the titer of each sample using the Takara Lenti-X Quant RT-qPCR kit (Takara Bio, Cat#: 631235). Viral supernatants were normalized to the same multiplicity of infection for infectivity assays.

U2OS cells were maintained in DMEM containing 10% fetal bovine serum, 1% non-essential amino acids, 1% sodium pyruvate, and 1% penicillin-streptomycin at 37°C with 5% CO_2_. U2OS cells were plated in 96-well plates at 7,500 cells per well and the normalized viral supernatant was added to the plate in duplicate. Media was exchanged 24 h later and then cells were analyzed by flow cytometry after 4 days.

#### Sequencing data preprocessing and quality control

Host transcriptomics data was processed using the umiRNAseq custom pipeline for Bulk RNA-seq Processing with UMI correction on Terra (https://github.com/broadinstitute/EbolaNaturalHistory/blob/main/00-bulk-rna-seq/umiRNASeq.wdl). Briefly, we merged and tagged raw Fastq files with their corresponding UMI barcode, and mapped, using the STAR aligner,^89^ to the rhesus monkey (*Macaca mulatta)* reference genome and annotation (Mmul_10). Resulting BAM files were filtered for multiple mapped reads, sorted and indexed using samtools. Then, PCR duplicates were removed by UMI-tools^90^ using the UMI barcodes of each transcript, and featureCounts were used to quantify expression from the aligned and processed RNA-Seq BAM files. We used the BioMart R package^91^ to annotate the gene type, gene name, and gene function using the ensembl *M*. *mulatta* database “mmulatta_gene_ensembl”. Quality control over the sample was performed removing samples with low sequencing quality and mismatched sex assignment.

#### Viral genomic analyses

Viral genomic analyses were performed using viral-ngs pipelines (https://github.com/broadinstitute/viral-ngs) implemented on the Terra platform (app.terra.bio). We assembled EBOV genomes using the assemble_refbased workflow (viral-ngs version 2.0.21), with the EBOV/Kikwit reference GenBank: KU182908.1. Genomes with >95% unambiguous bases were considered complete. On all genomes with >400x mean depth of coverage, we used LoFreq with -q 20 and -Q 20 to identify minor variants, relative to the EBOV reference GenBank: KU182908.1.^92^ We filtered out variants that were present in <2% or >98% of reads mapping to a given position (relative to reference), as well as those at sites with depth of coverage <100 and variant reads <5.

#### Viral mutation statistics

A one-tailed exact binomial test with p = 0.75 was used to determine whether the ratio of nonsynonymous to synonymous mutations in a given analysis differed from the expected 3:1 ratio for neutral selection.These analyses were done within a tissue across all genes, and also with respect to a particular gene across all tissues. A one-tailed permutation test (with 10,000 trials) was used to determine whether the ratio of nonsynonymous to synonymous mutations differed between high-frequency and low-frequency variants.

#### Differential expression analysis

The raw read counts of all samples were normalized using the DESeq2 R package.^93^ In order to identify tissue markers, we compared counts from samples at time zero and 3 days post infection (DPI) using a model matrix to compare each tissue against all others. Genes with an adjusted p-Value and a log2 fold change higher than one in each comparison were selected as tissue markers for that specific tissue.

To identify differentially expressed genes between not infected (samples at 0 DPI) and infected conditions, samples were further analyzed with the DESeq2 package.^93^ For tissues lacking samples at 0 DPI (lung, liver and testis) samples at 3 DPI were used instead. For each tissue, genes previously identified as tissue markers were excluded from downstream interpretation. We considered differentially expressed genes (DEGs) to be those genes with a p-adj <0.05 and a log2 fold change higher than 2. Genes meeting these criteria were stratified into ISGs, Cytokines, Inflammatory response, PARPs, apoptosis, and extracellular matrix related genes using the go.db.df R package and custom lists.

#### GO term enrichment analysis and correlation analysis

Enrichment analysis was performed on DEGs using the R package topGO^94^ with the “Biological Process” ontology. For each tissue, we selected the top 100 DEGs across time (FDR <0.01) for this analysis. We selected the top 3 enriched terms for each tissue as defined by the p values of the Kolmogorov-Smirnov test. Correlation between host genes and viral counts was performed using the normalized DESeq2 counts and the total viral read counts using Spearman rank correlation analysis as implemented in the stats R package. A similar approach was performed for the correlation between viral load and monocyte markers (mean of *CTSS*, *VCAN*, *FCN1*, *CD14*, *S100A9*, *MS4A1* normalized counts) and whole-blood non-monocyte markers (mean of *CD3D*, *HBA*, *SELL*, *PPBP*, *HBA*, *CD8A*, *GNLY* normalized counts).

#### Genes expression changes across time

To identify genes changing across time, we used the ImpulseDE2 package^95^ to perform a time-series differential expressed gene analysis of each tissue across the 8 days of infection. ImpulseDE2 includes a DEseq2 normalization step, thus, the raw gene read counts from FeatureCounts were used as input data. The function “runImpulseDE2” was applied to each tissue independently, significant genes were selected as those with a p-adj <0.05. Furthermore tissue marker genes corresponding to each tissue were excluded from downstream analysis.

The data analysis mentioned before were performed in R version 4.1.2, using the aforementioned R packages. Visualization was performed using the Packages ggplot2, Pheatmap^96^ and ComplexHeatmap.

#### Time-regularized deconvolution of bulk RNA sequencing (ternaDecov)

We developed ternaDecov as a time-regularized method for deconvolution of bulk sequencing data using scRNA-seq reference data. Briefly, ternaDecov uses stochastic variational inference to simultaneously identify an underlying trajectory of cellular composition change in terms of user-specified covariates (e.g., days post infection) and deconvolve individual sample compositions using annotated single-cell profiles. The code for the ternaDecov software is available from github at https://github.com/broadinstitute/temporal-rna-seq-deconvolution as an installable python package and several introductory tutorials are provided.

TernaDecov offers a modular model structure in which the cell type proportions of each sample are obtained from one of several alternative trajectory modules. The trajectory modules take as input the sampling time covariate and return a draw of sample-specific cell proportions ($\pi_{nc}$) as a result in different ways depending on their internal structure. Trajectory modules currently implemented in ternaDeCov include: (1) simple polynomial trajectories, (2) Legendre polynomial trajectories, (3) Gaussian process with different kernel functions, and (4) a “trivial” trajectory model that does not take into account sample collection time, effectively producing independent deconvolution of samples similar to traditional deconvolution algorithms.

The cell-type proportions ($\pi_{nc}$) are multiplied with the summarized single-cell reference (…) after scaling by learnable gene specific capture rate coefficients ($\beta_{g}$) to produce location parameter for a Negative Binomial distribution from which the observed count matrix is sampled from using gene specific dispersion parameters ($\varphi_{g}$).

The full model is specified as follows:

n:sampleindex

c:celltypeindex

g:geneindex

$$N_{n}:total\;library\;size$$

$$\tau_{n}:sampling\;times$$

$${\hat{W}}_{gc}:summarized\;single-cell\;reference$$

$$\mu_{g}:gene-specific\;dispersion\;mean$$

$$\sigma_{g}:gene-specific\;dispersion\;variance$$

$$\sigma_{\beta }:gene-specific\;capture\;rate\;variance$$

$$W_{gc}=\beta_{g}{\hat{W}}_{gc}$$

$$\varphi_{g}=N\left(\mu_{g},\sigma_{g}\right)$$

$$\beta_{g}=N\left(0,\sigma_{\beta }\right)$$

$$\pi_{nc}=Trajectory\;Module\left(\tau_{n}\right)$$

$$X_{ng}=NB\left(N_{n}\pi_{nc}{\hat{W}}_{cg},\varphi_{g}\right)$$

#### ternaDecov: Trajectory models

TernaDecov offers two trajectory models, described below.

##### Polynomial trajectory model

The polynomial trajectory model is shown in Figure S14A (left). To obtain the prior cell proportions for a given sample n at time $\tau_{n}$, we evaluate a specified polynomial function basis $\varphi_{k}\left(.\right)$ for k=1,…,K on $\tau_{n}$ to obtain a polynomial feature matrix $\varphi_{k}\left(\tau_{n}\right)$. At the same time, we (globally) sample a set of weights $z_{kc}∼N\left(0,{\alpha_{k}}^{-1}\right)$, where $\alpha_{k}$ is the precision of prior Gaussian and controls the usage of basis function $\varphi_{k}$. We matrix multiply the global weights with the sample polynomial feature matrix to obtain the unnormalized cell population $y_{nc}=\sum_{k=1}^{K}z_{kc}\varphi_{k}\left(\tau_{n}\right)$. We normalize the latter by applying the softmax function along the last dimension to obtain ${\hat{\pi }}_{nc}=softmax\left(y_{nc}\right)$. To allow sample-specific deviations from this prior trajectory, we finally sample $\pi_{nc}$ from a Dirichlet distribution $\pi_{nc}∼\;Dirichlet\left(\alpha_{dir}{\hat{\pi }}_{nc}\right)$. Here, $\alpha_{dir}$ is the global Dirichlet concentration parameter which controls how sample trajectories can deviate from the prior trajectory.

##### Gaussian process (GP) trajectory model

In contrast to the polynomial model, the GP model (Figure S14A, right) allows for more flexible trajectories. The function space of trajectories is specified by the kernel function, and the parameters of the kernel function are optimized to obtain the maximum likelihood trajectory fit. To obtain the prior cell proportions for a given sample n at time $\tau_{n}$, we draw unnormalized cell proportions $y_{nc}$ independently for each cell type using a cell-type-specific GP and sample collection time $\tau_{n}$ as the covariate. We specifically used radial basis function (RBF) kernel function with added white noise $k\left(\tau ,\tau^{\prime }\right)=\sigma_{0}\;\exp\left(-|\tau -\tau^{\prime }{|}^{2}/2T^{2}\right)+\sigma_{1}\;\delta \left(\tau ,\tau^{\prime }\right)$, where $\theta_{GP}=\left\{\sigma_{0},\sigma_{1},T\right\}$ constitute the set of GP kernel parameters to be optimized. Intuitively, a larger choice of $\sigma_{1}$ allows for more sample-to-sample trajectory deviation, a larger choice of $\sigma_{0}$ couples adjacent times more strongly together (i.e., stronger time regularization), and T sets the trajectory correlation timescale. Like before, we normalize the unnormalized cell population $y_{nc}$ by applying the softmax function along the last dimension to obtain $\pi_{nc}=softmax\left(y_{nc}\right)$. In contrast to the polynomial model, $y_{nc}$ is already a latent variable which accommodates for sample-to-sample deviation from the trajectory. Therefore, sampling from the Dirichlet distribution is no longer needed in this approach.

#### ternaDecov: Implementation

TernaDecov is implemented in python as a hierarchical model using the pyro^97^ probabilistic programming framework. When available, ternaDecov can utilize underlying CUDA graphics processors for acceleration. Parameter estimation is performed using the Adam with a learning rate of 1e-3 optimizer and an ELBO loss; 20,000 learning iterations are utilized unless noted otherwise. TernaDecov can be run using a CLI interface or via API calls using a jupyter notebook. Inputs for ternaDecov execution encompass two scanpy AnnData objects: one for the single-cell reference (that requires a cell type annotation column) and one for the bulk data that requires a column annotating the time of collection of each sample. The results can be exported in tabular format as well as plotter in raster and vector formats.

The package provides facilities for simulating random sample proportion trajectories using different basis functions that are different in functional form from the bases used to estimate trajectories and include softmax normalized sigmoid, sinusoidal and linear (first degree polynomial) trajectories, using the Simulator module. Furthermore, the package allows for automated scanning of prior parameters and configuration options for assessing stability of results with respect to these values, using the SensitivityAnalyzer module.

#### ternaDecov: Technical benchmarking

##### Run time

We benchmarked runtime performance using simulated samples from a fixed random trajectory (Figure S14B). Furthermore, deconvolution of 10 adrenal samples with ternaDecov required 4.7 min, MuSic accomplished the same task in 57.9 min. Although scaling with the number of samples is exponential, running time for 1000 samples is sufficiently short to be run interactively. Scaling of the polynomial trajectory module is more linear that the full GP shown here. We anticipate that memory limitations will be more important than execution time when utilizing the GP model. We found that executing the model using a GPU processor accelerated execution (data not shown).

##### Accuracy

We assessed the value of i) increasing sample number and ii) trajectory estimation on improving sample composition estimates with ternaDecov. Using the built-in simulator we assessed the ability of ternaDecov to recover underlying trajectories from which bulk samples are derived as function of the number of equidistant temporal samples obtained. We generated a single random fixed periodic type of trajectory (Figure S14C) and increasingly sampled N equidistant samples from it. After learning the underlying trajectory we evaluated composition values as 1000 points and scored trajectory reconstruction quality by means of normalized L1 error. L1 error declined with increasing sample numbers, indicating that larger sample sizes improve trajectory estimation (Figure S14D).

Sample proportion and simultaneous trajectory estimation is expected to reduce the error of individual sample proportion estimation as information between samples is shared. In order to confirm that, we deconvolved fixed trajectory using the 'gp' and the 'nontrajectory' deconvolution models. The 'nontrajectory' model does not impose any trajectory structure between samples and therefore does not share any information between samples. It is therefore expected to reflect the performance of all general methods for deconvolution that do not make use of covariate information. The normalized L1 error for 10 independent deconvolution runs on the same dataset was markedly higher without trajectory estimation (Figure S14E), supporting the value of this approach.

##### Robustness

We extensively evaluated the robustness of ternaDecov to perturbations of the prior parameters and gene selection algorithm. For example, using an increasingly stringent parameter for the overall abundance of genes in the single-cell dataset the results remain stable well beyond the values used for the analysis (Figure S14F).

#### ternaDecov: Biological benchmarking and application to EBOV RNAseq data

To benchmark the method on independent biological datasets, we first used the bulk RNA-seq data from Fadista et al.^36^ which contain RNA-seq data for healthy and diseased pancreatic islet samples simulated based on pancreatic islets scRNA-seq RNA-seq data from Segerstolpe et al.^37^ We ran ternaDecov with HbA1C as the covariate to use for trajectory regularization. We compared cell proportions estimated by ternaDecov to those reported for MuSiC^32^ and established quantitative agreement between the two methods. Moreover, ternaDecov inferred cell type composition trajectories were concordant with the results reported earlier.^98^

In order to assess blood infiltration in peripheral tissues during EBOV infection we applied ternaDecov to bulk RNAseq data with two alternative datasets as a single-cell reference; *Macaca fascicularis* single-cell atlas data,^99^ and peripheral blood data from the same EBOV-infected rhesus monkeys.^18^ We performed summarization of the deconvolved cell type proportions to 'Monocytes', 'non-Monocyte blood' and tissue-specific cell types. In all cases, we ran ternaDecov for 20,000 iterations for each analysis in the 'GP trajectory' mode with default settings for gene selection. Stability analysis with respect to the most salient input parameters was performed using 5,000 iterations. We validated the finding of a decrease in Chromaffin cells in adrenal tissue with MuSiC^32^ run using the default parameters and identical single-cell reference.

## Supplemental information

Document S1. Figures S1–S14 and Data S1

Table S1. qRT-PCR results across samples, related to Figure 1

Table S2. Quality control of samples, related to Figure 1

Table S3. DEGs across samples, related to Figure 3

Table S4. All viral variants identified, related to Figure 4

Table S5. Primers used for minigenome system and GP pseudotype assay plasmid construction, related to Figure 5

Document S2. Article plus supplemental information
