# Supplementary material for: Natural history of Ebola virus disease in rhesus monkeys shows viral variant emergence dynamics and tissue-specific host responses
Source: Cell Genom. 2023 Nov 21;3(12):100440. doi: 10.1016/j.xgen.2023.100440 (PMC10759212; doi:10.1016/j.xgen.2023.100440)
Supplement: Document S1. Figures S1–S14 and Data S1 [file mmc1.pdf]

Cell Genomics, Volume *3*

Supplemental information

Natural history of Ebola virus disease in rhesus monkeys shows viral variant emergence dynamics and tissue-specific host responses

Erica Normandin, Sergio Triana, Siddharth S. Raju, Tammy C.T. Lan, Kim Lagerborg, Melissa Rudy, Gordon C. Adams, Katherine C. DeRuff, James Logue, David Liu, Daniel Strebinger, Arya Rao, Katelyn S. Messer, Molly Sacks, Ricky D. Adams, Krisztina Janosko, Dylan Kotliar, Rickey Shah, Ian Crozier, John L. Rinn, Marta Melé, Anna N. Honko, Feng Zhang, Mehrtash Babadi, Jeremy Luban, Richard S. Bennett, Alex K. Shalek, Nikolaos Barkas, Aaron E. Lin, Lisa E. Hensley, Pardis C. Sabeti, and Katherine J. Siddle

Supplemental Figures

Supplemental figures for “Natural history of Ebola virus disease in rhesus monkeys shows viral variant emergence dynamics and tissue specific host responses” by Normandin et al.

Figures Contents

Figure S1. qPCR quantification compared to viral read counts.

Figure S2. Overview of samples profiled.

Figure S3. Host transcriptome dimensionality reduction.

Figure S4. Expression profiles during infections across tissues

Figure S5. Viral load correlates with monocyte markers.

Figure S6. Cell type deconvolution of Bulk RNA-seq.

Figure S7. Tissue specific marker genes.

Figure S8. Genes change across time.

Figure S9. ECM and Coagulation related genes change across time and tissues.

Figure S10. Reliability of minor variant calling methodology.

Figure S11. Mutation types across the sample set.

Figure S12. Mutations across animals and tissues.

Figure S13. Functional characterization of viral variants

Figure S14. Utilizing Time-series Covariate Information for Enhanced RNA-seq Deconvolution

Data S1: Pentacistronic Minigenome Assay plasmid sequences, related to STAR methods.

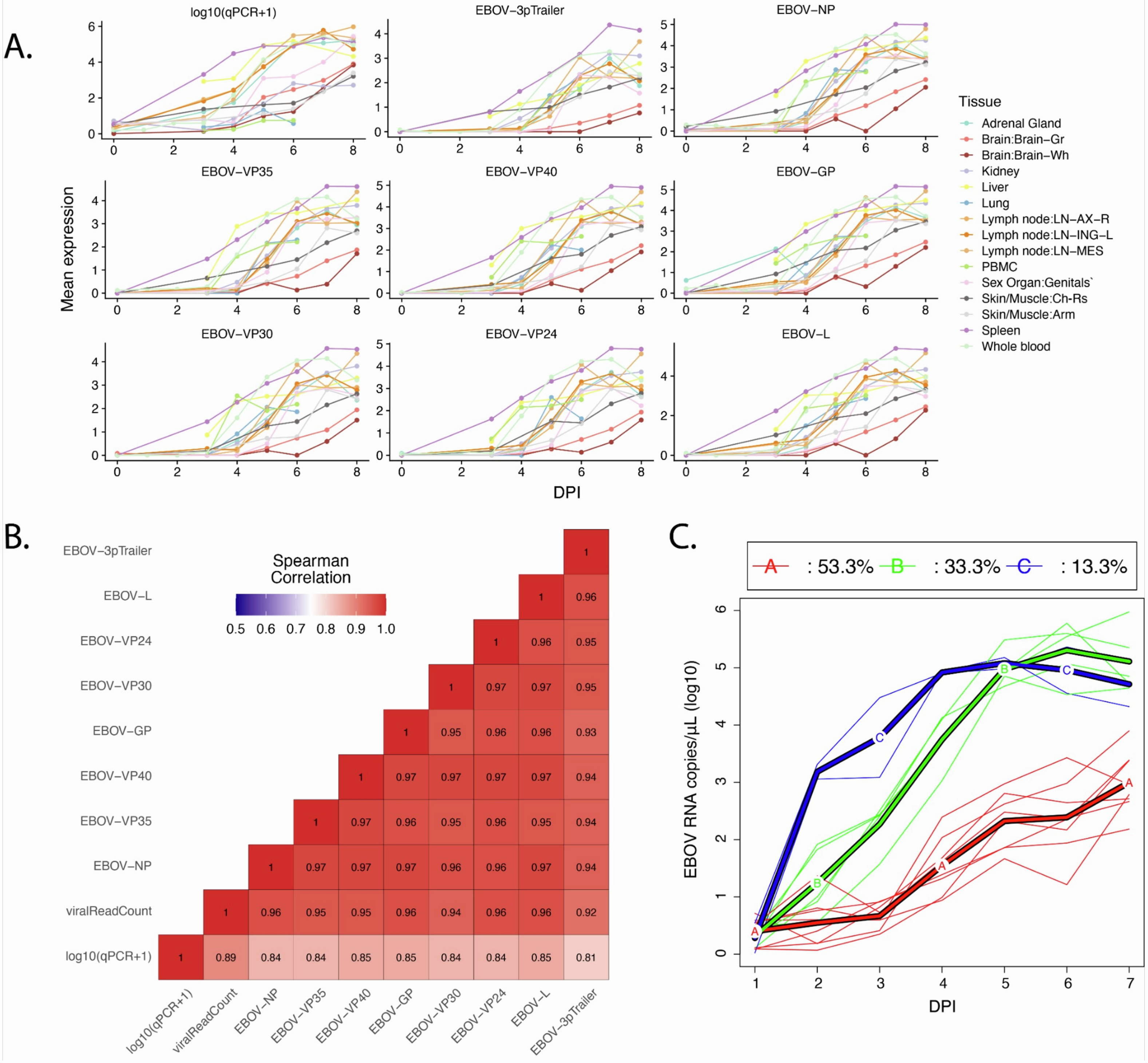

Figure S1. qPCR quantification compared to viral read counts, related to figure 1. (A) Mean expression of viral genes across tissues and DPI (B) Heatmap showing spearman correlations between each viral gene, the total viral read count and the qPCR quantification of GP RNA. (C) Longitudinal K-mean clustering of qPCR data.

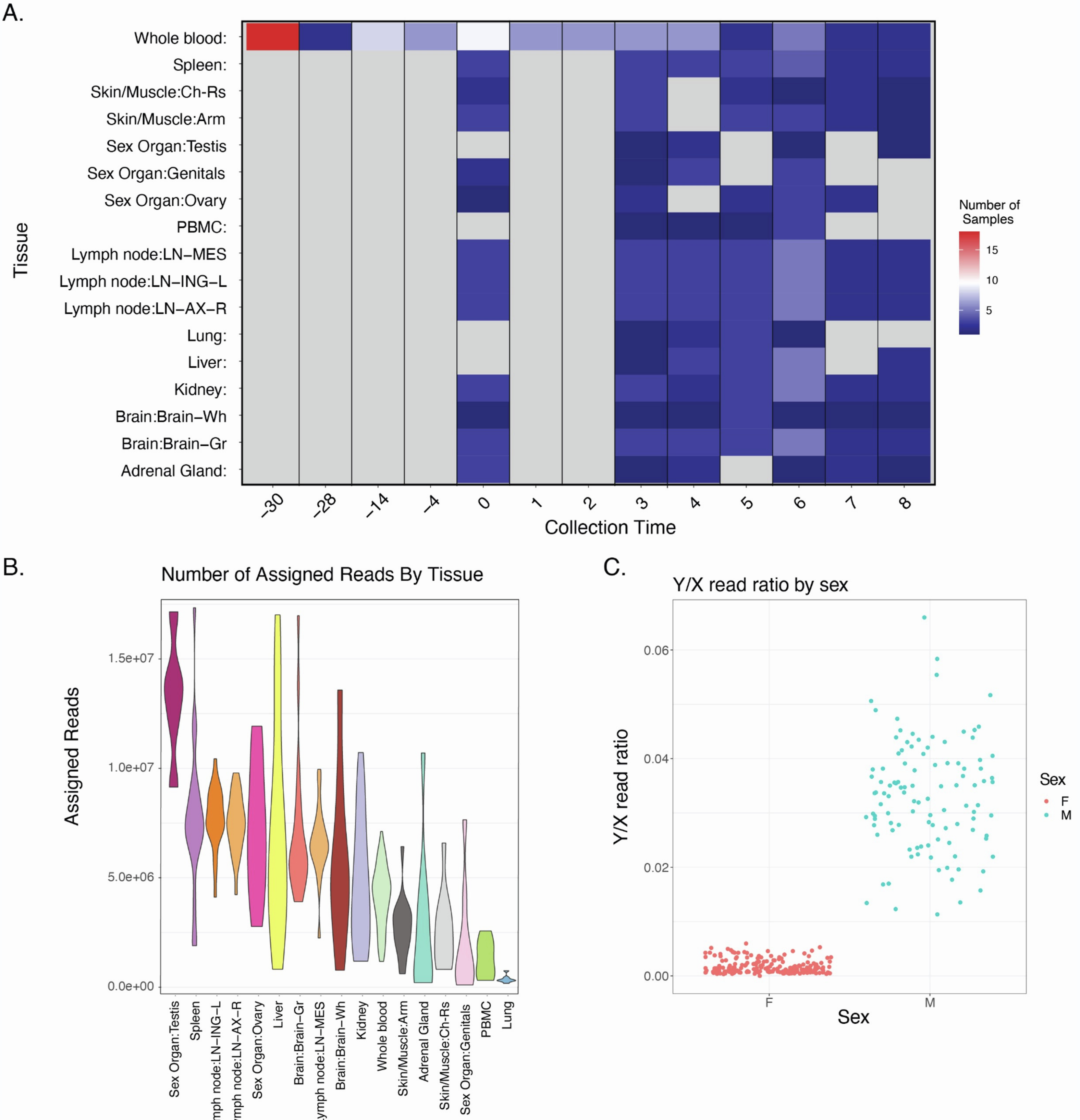

Figure S2. Overview of samples profiled, related to figure 1. (A) Sequenced sample count per tissue and collection time (B) Violin plots of total assigned reads per tissue. (C) Chromosome X and Y ratio per sample.

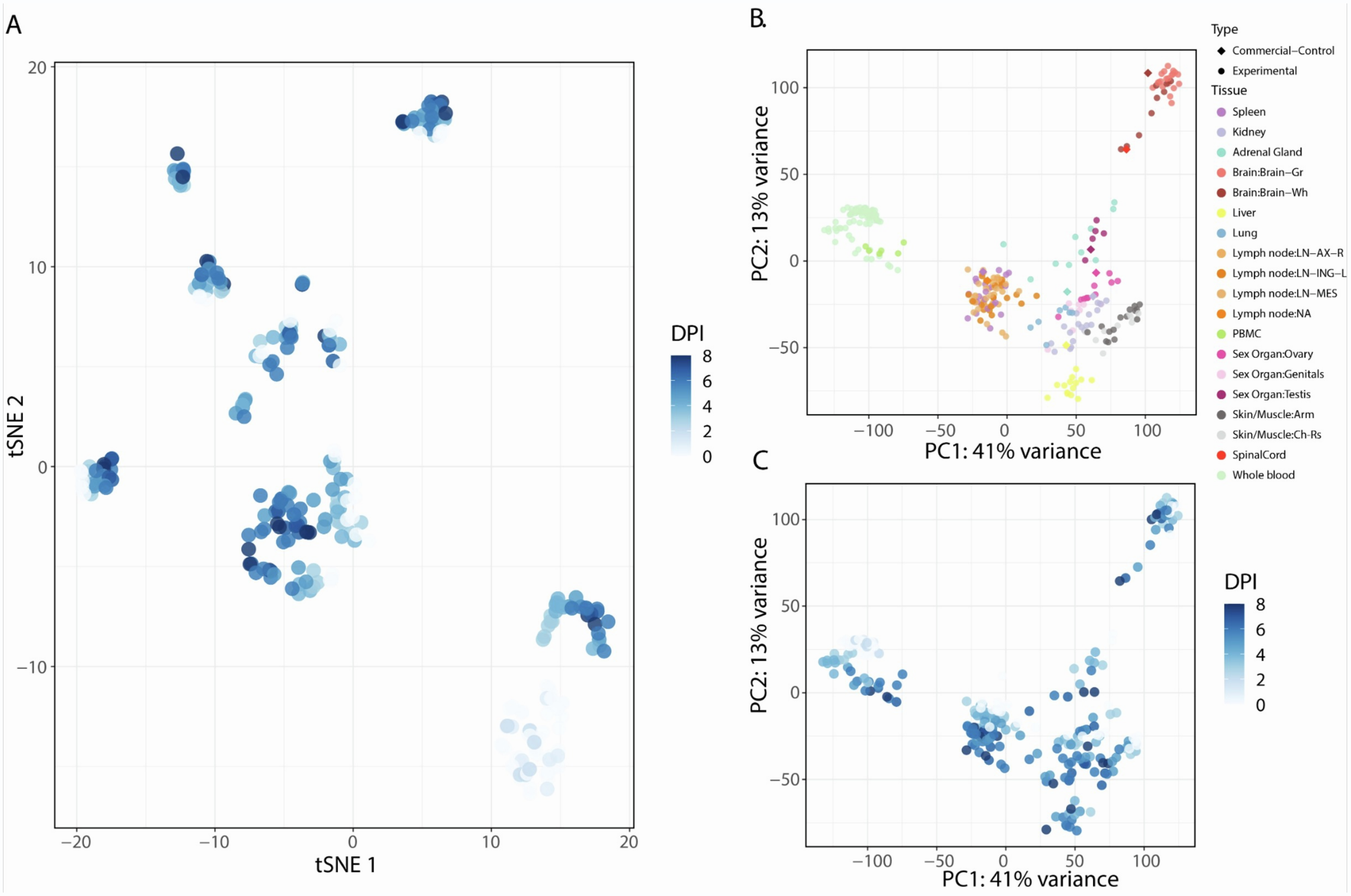

Figure S3. Host transcriptome dimensionality reduction, related to figure 1. (A) tSNE plot of transcriptional signatures colored by DPI. PCA of transcriptional profiles colored by (B) sample type and (C) DPI.

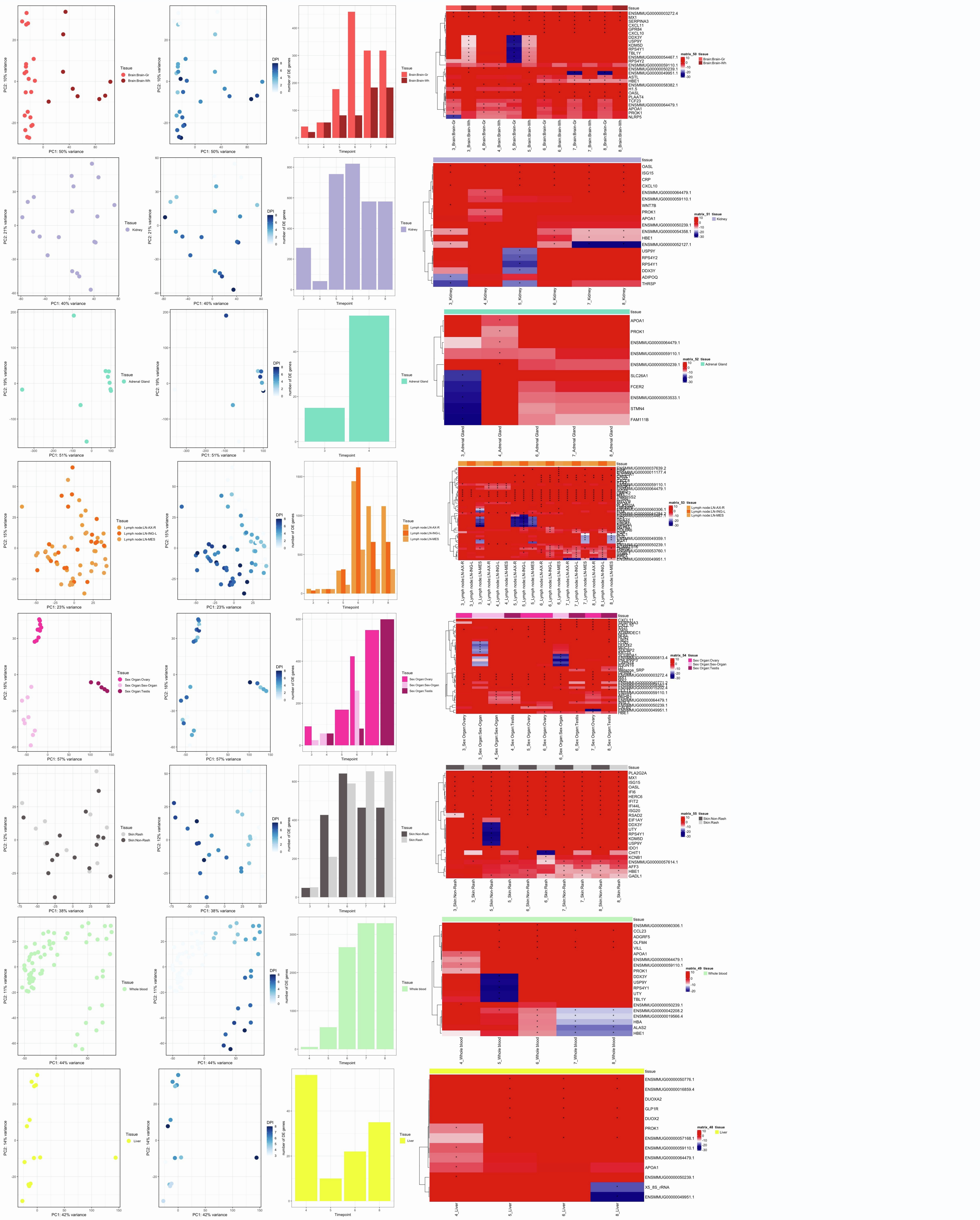

PC1: 50% variance
PC2: 10% variance
Tissue
Brain:Brain-Gr
Brain:Brain-Wh
DPI
number of DE genes
Timepoint
tissue
ENSMMUG00000003272.4
MX1
SERPINA3
CXCL11
GPR84
CXCL10
DDX3Y
USP9Y
KDM5D
RPS4Y1
TBL1Y
ENSMMUG00000054467.1
RPS4Y2
ENSMMUG00000059110.1
ENSMMUG00000050239.1
ENSMMUG00000049951.1
ASTL
HBE1
ENSMMUG00000058382.1
H1.5
OASL
PLAAT4
TCF23
ENSMMUG00000064479.1
APOA1
PROK1
NLRP5
matrix_50
PC1: 40% variance
PC2: 21% variance
Kidney
OASL
ISG15
CRP
CXCL10
ENSMMUG00000064479.1
ENSMMUG00000059110.1
WNT7B
PROK1
APOA1
ENSMMUG00000050239.1
ENSMMUG00000054358.1
HBE1
ENSMMUG00000052127.1
USP9Y
RPS4Y2
RPS4Y1
DDX3Y
ADIPOQ
THRSP
matrix_51
PC1: 51% variance
PC2: 19% variance
Adrenal Gland
APOA1
PROK1
ENSMMUG00000064479.1
ENSMMUG00000059110.1
ENSMMUG00000050239.1
SLC26A1
FCER2
ENSMMUG00000053533.1
STMN4
FAM111B
matrix_52
PC1: 23% variance
PC2: 15% variance
Lymph node:LN-AX-R
Lymph node:LN-ING-L
Lymph node:LN-MES
matrix_53
PC1: 57% variance
PC2: 16% variance
Sex Organ:Ovary
Sex Organ:Sex-Organ
Sex Organ:Testis
matrix_54
PC1: 38% variance
PC2: 12% variance
Skin:Rash
Skin:Non-Rash
PLA2G2A
MX1
ISG15
OASL
IFI6
HERC6
IFIT2
IFI44L
ISG20
RSAD2
EIF1AY
DDX3Y
UTY
RPS4Y1
KDM5D
USP9Y
IDO1
CHIT1
KCNB1
ENSMMUG00000057614.1
AFF3
HBE1
GADL1
matrix_55
PC1: 44% variance
PC2: 11% variance
Whole blood
ENSMMUG00000060306.1
CCL23
ADGRF5
OLFM4
VILL
APOA1
ENSMMUG00000064479.1
ENSMMUG00000059110.1
PROK1
DDX3Y
USP9Y
RPS4Y1
UTY
TBL1Y
ENSMMUG00000050239.1
ENSMMUG00000042208.2
ENSMMUG00000019566.4
HBA
ALAS2
HBE1
matrix_49
PC1: 42% variance
PC2: 14% variance
Liver
ENSMMUG00000050776.1
ENSMMUG00000016859.4
DUOXA2
GLP1R
DUOX2
PROK1
ENSMMUG00000057168.1
ENSMMUG00000059110.1
ENSMMUG00000064479.1
APOA1
ENSMMUG00000050239.1
X5_8S_rRNA
ENSMMUG00000049951.1
matrix_48

Figure S4. Expression profiles during infections across tissues related to figure 3. Right, PCA of transcriptional profiles of each tissue colored by sample type and DPI. Center, Number of differentially expressed genes between non-infected and each time point. Heatmap of Fold-changes of Top DE genes in each time point and tissue

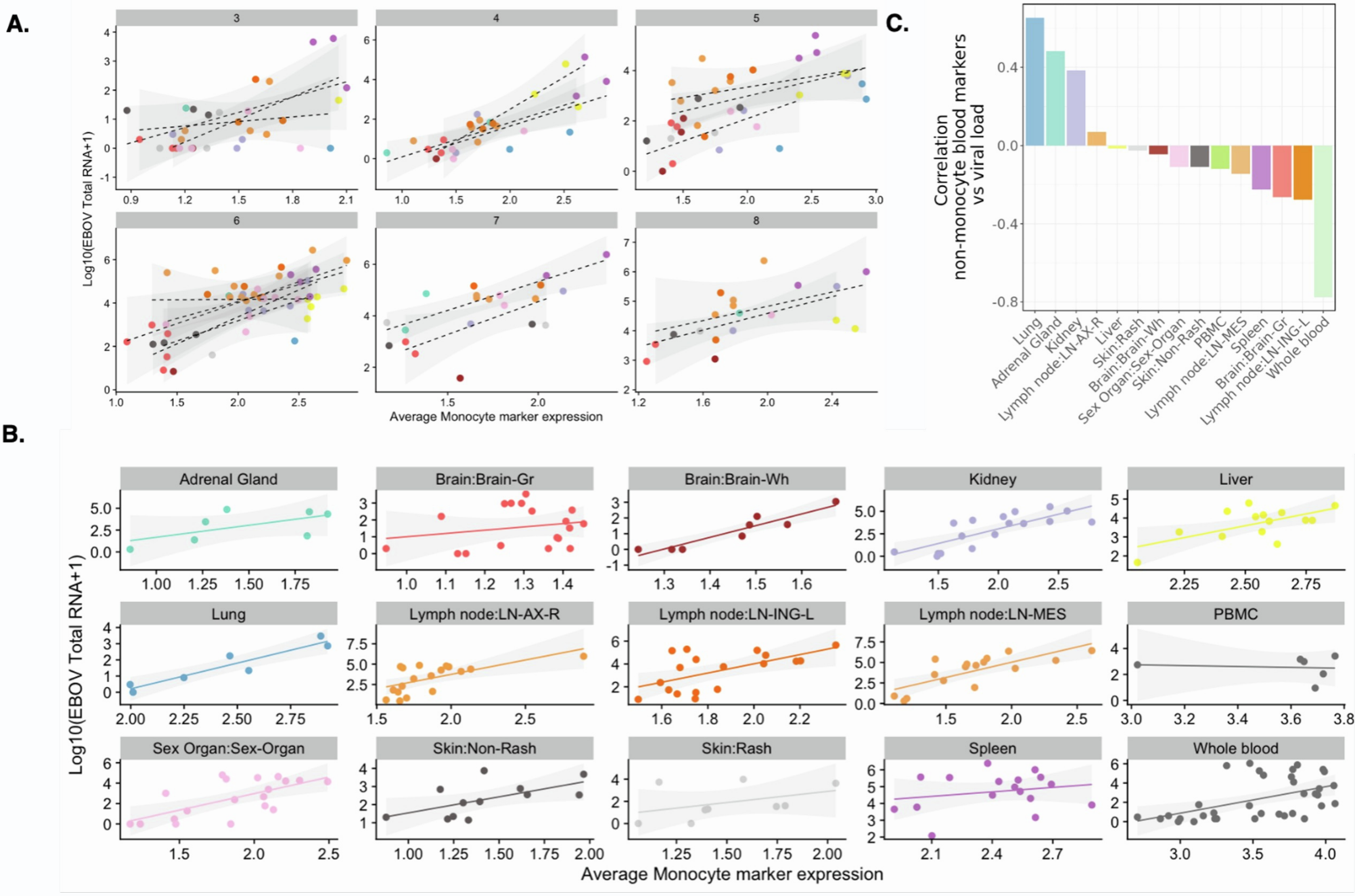

Figure S5. Viral load correlates with monocyte markers, related to figure 2. (A) Total viral counts compare to the average expression of canonical monocyte markers (*CTSS, VCAN, FCN1, CD14, S100A9*) across each time by individual (A) and by tissue (B). (C) Correlation between viral load and non-monocyte blood markers (*CD3D,HBA, SELL, PPBP, HBA,CD8A, GNLY, CD4*) expression across each tissue.

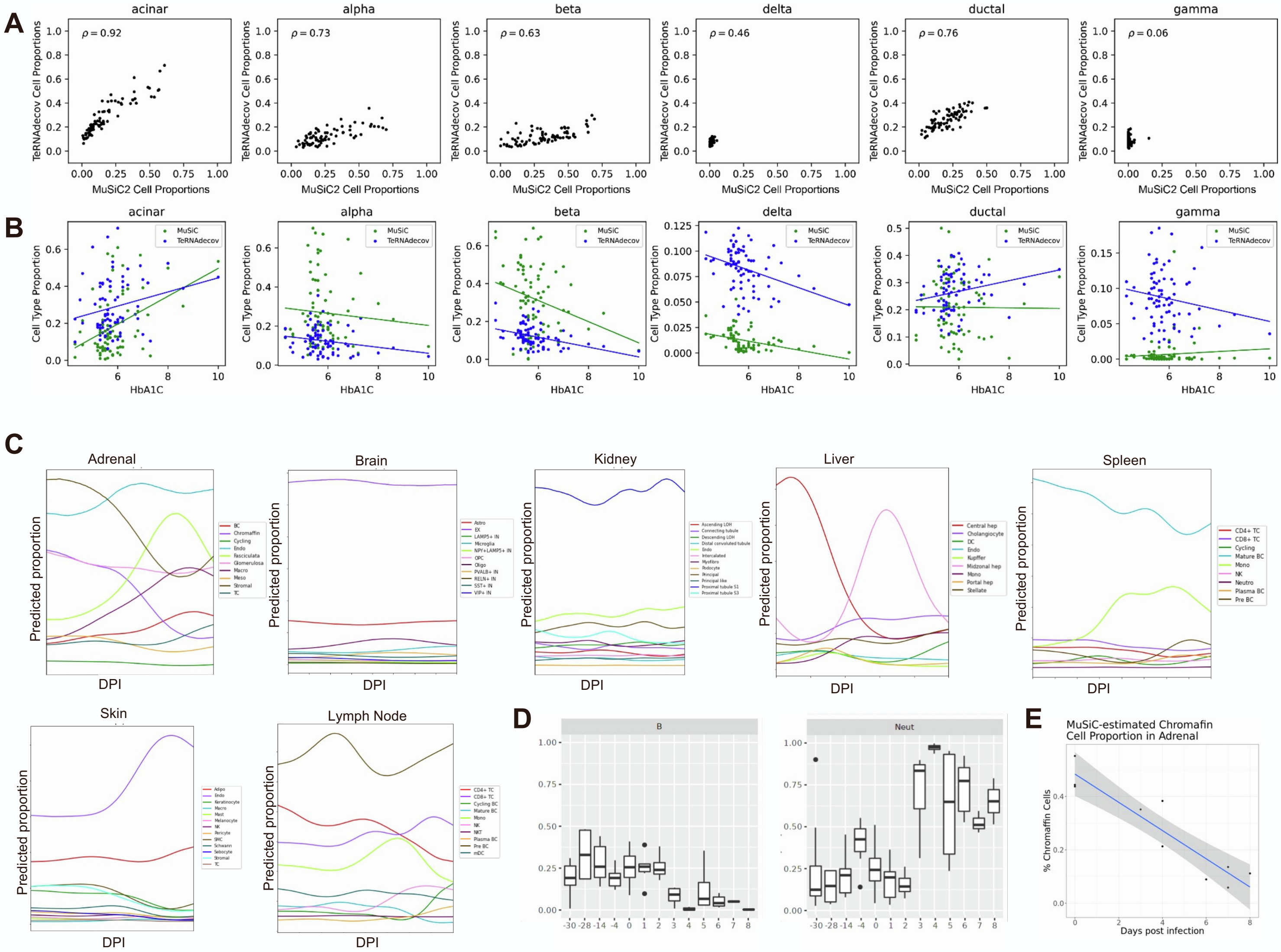

Figure S6. Cell type deconvolution of Bulk RNA-seq, related to figure 2. A) Predicted cell type proportion for pancreatic islet bulk RNA-seq data from Fadista et al using ternaDecov and MuSiC2. B) Correlation of HbA1c level and cell-type composition C) Deconvolution of predicted cell types changes across time for each tissue based on a single-cell RNAseq reference of *Macaca fascicularis*. (D) Deconvolution of Whole Blood with MuSiC confirms Neutrophil peak at 4 DPI. (E) Deconvolution of adrenal tissues using MuSiC confirms the reduction of the relative proportion of Chromaffin cells during infection.

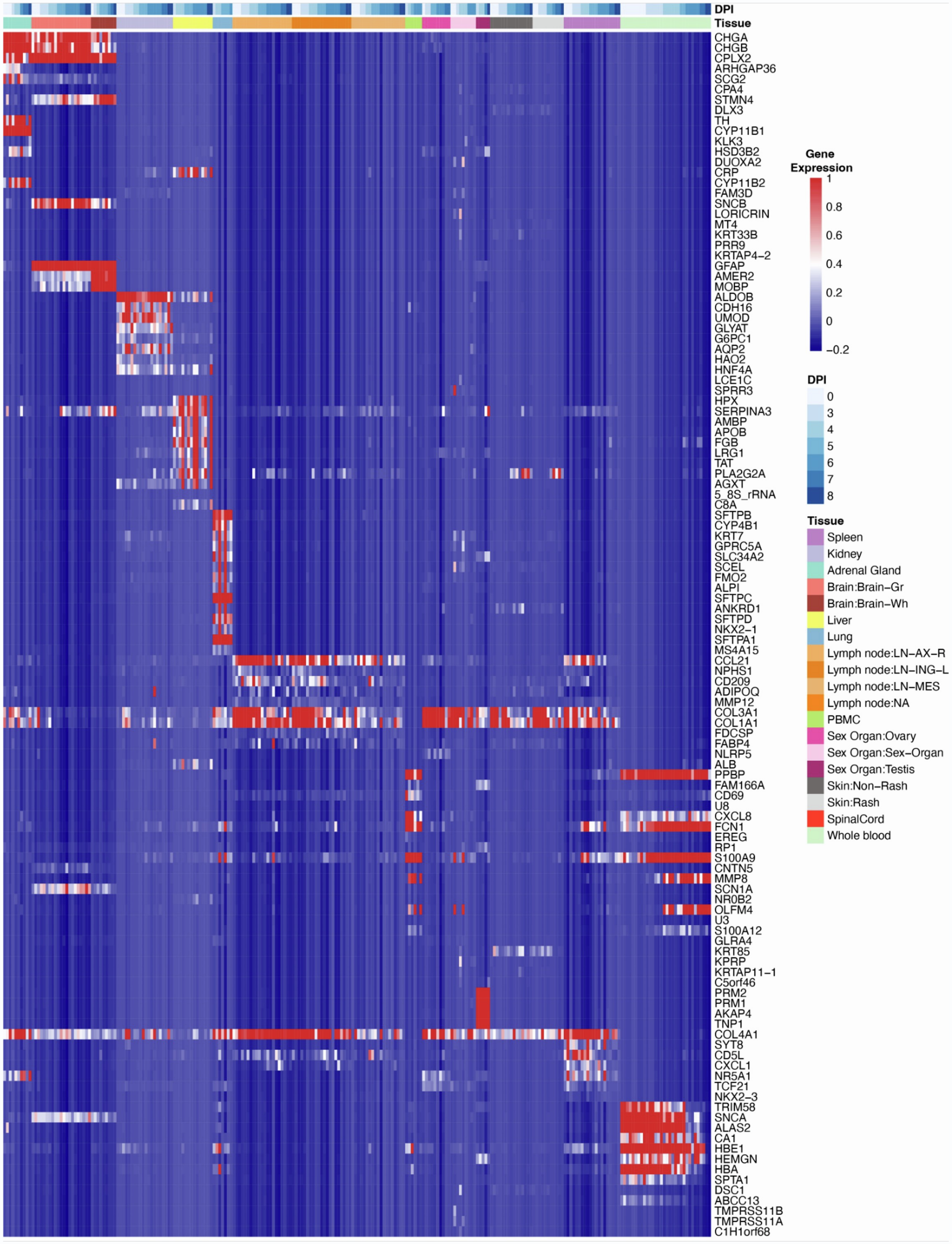

Figure S7. Tissue specific marker genes, related to figure 3. Heatmap of top specific tissue marker genes across time points.

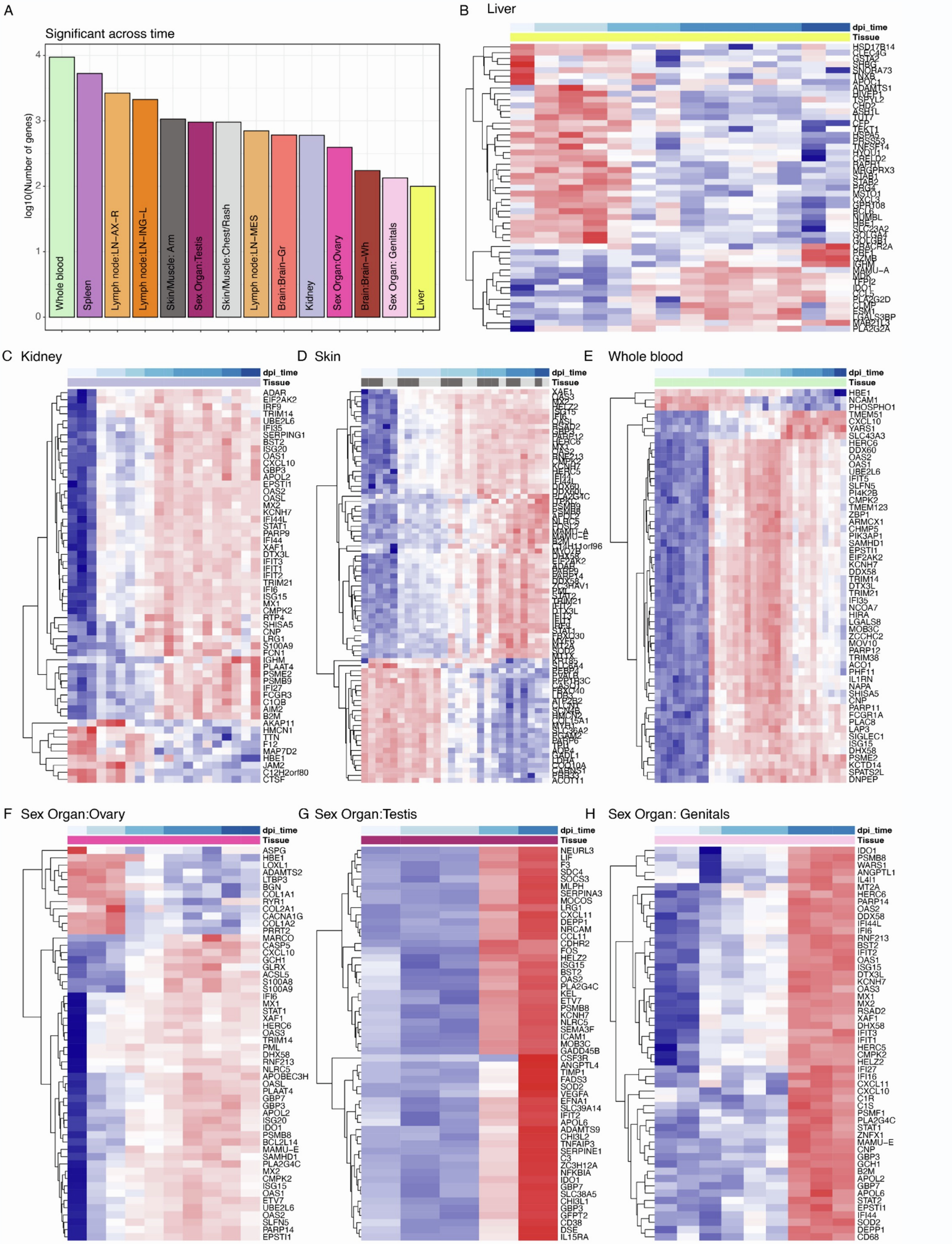

Figure S8. Genes change across time, related to figure 3. (A) Number of differentially expressed genes across time, tissues with more than 5 DE genes are shown in the plot. Heatmap of genes changing significantly across time for (B) Liver (C) Kidney (D) Skin (E) Whole Blood (F) Ovary (G) Testis and (H) Genitals

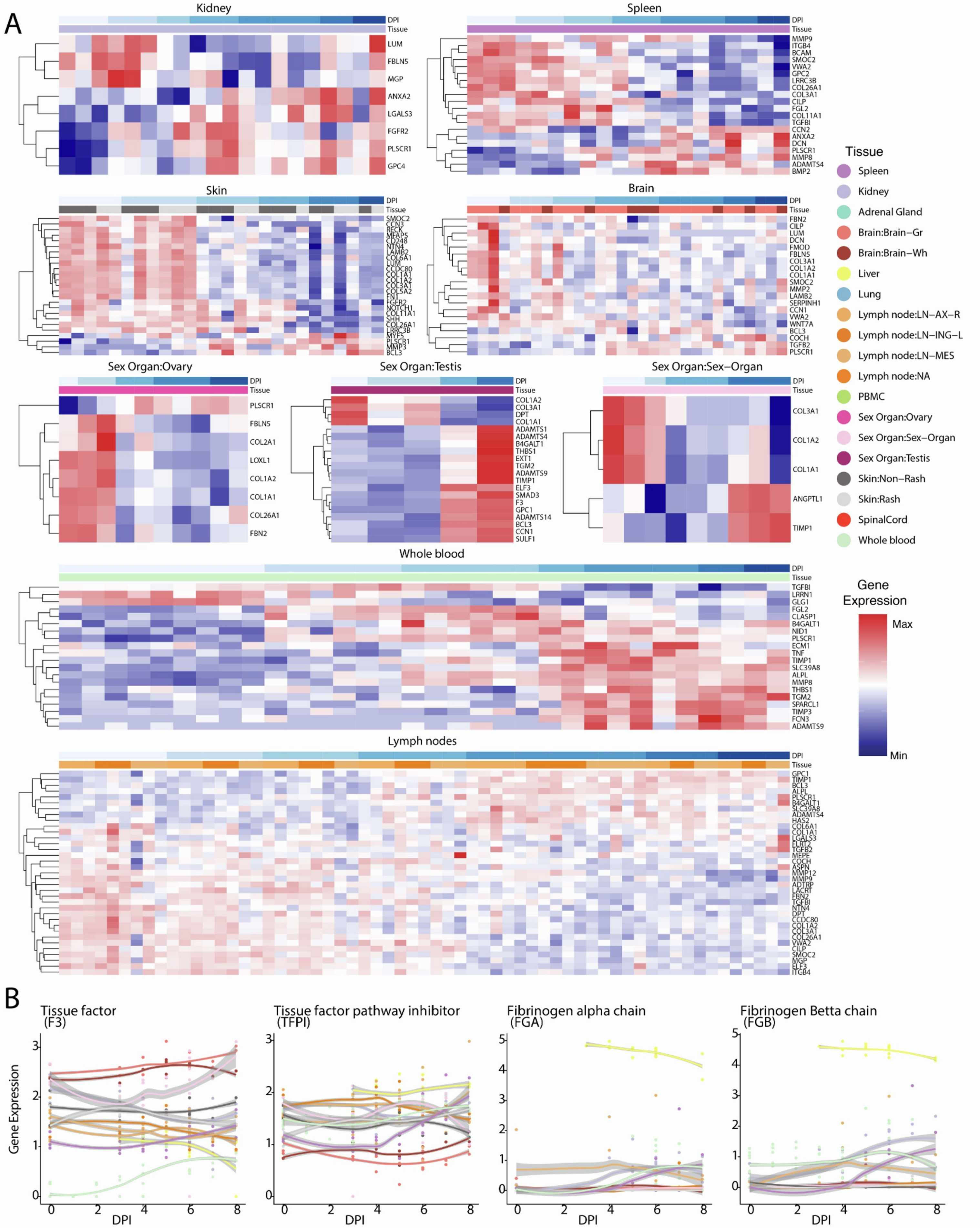

Figure S9. ECM and Coagulation related genes change across time and tissues, related to figure 3. (A) Heatmap of ECM genes that significantly change across time for each tissue (ImpulseDE2 Adjusted P-Value < 0.05). (B) Selected coagulation related genes expression.

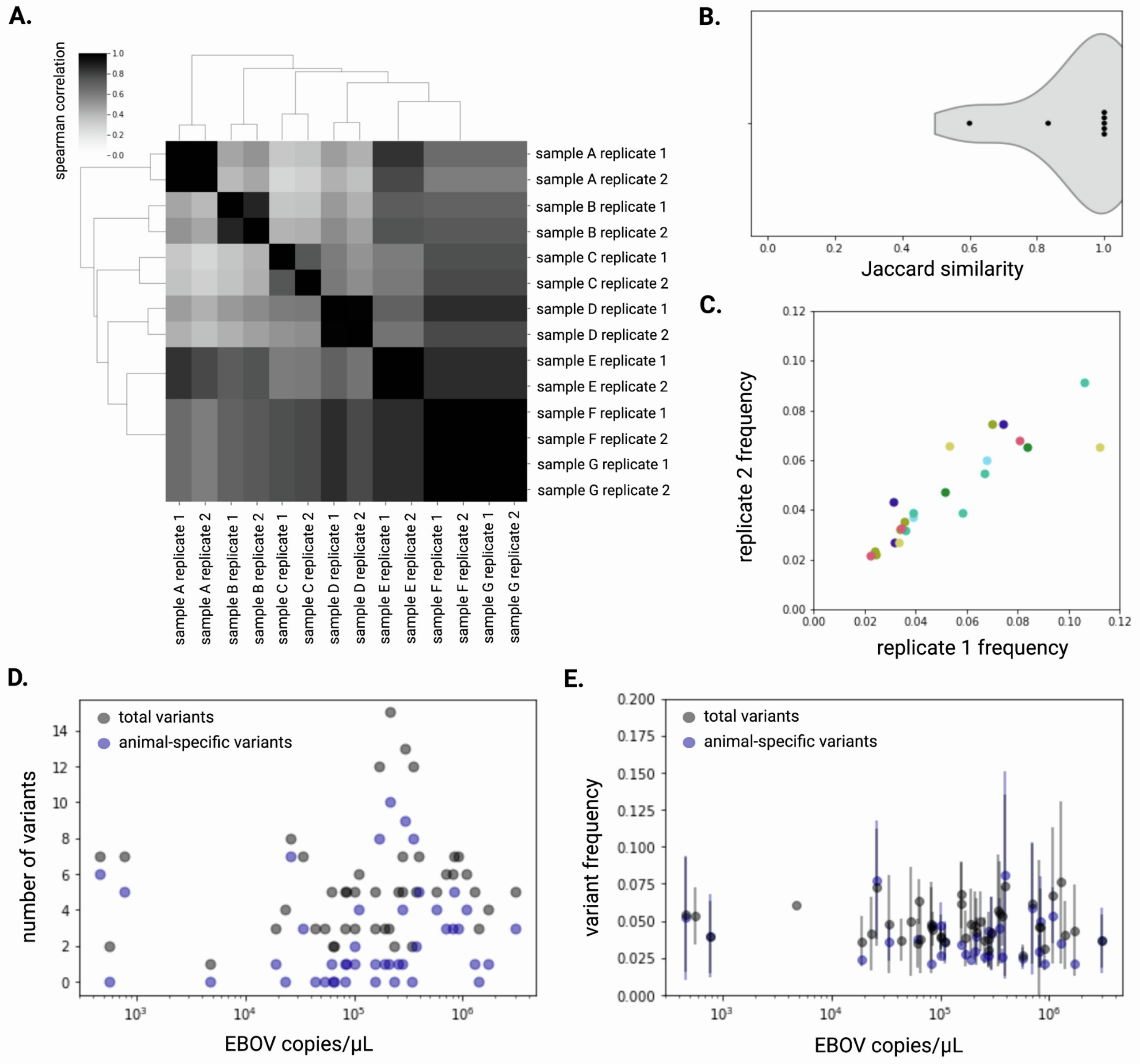

Figure S10. Reliability of minor variant calling methodology, related to figure 4. (A) Spearman correlation of variant profiles of 7 select samples with duplicate sequencing. (B) Violin plot of Jaccard similarities of variants identified in duplicate libraries from 7 samples. (C) For variants identified in two replicates, comparison of the frequency at which the variant was identified in each replicate. Each of the 7 samples is represented by a different color. (D) Total (black) and animal-specific (blue) variants identified versus viral load in each sample profiled. (E) Mean variant frequency versus viral load in each sample profiled. Error bars represent standard deviation of variant frequency.

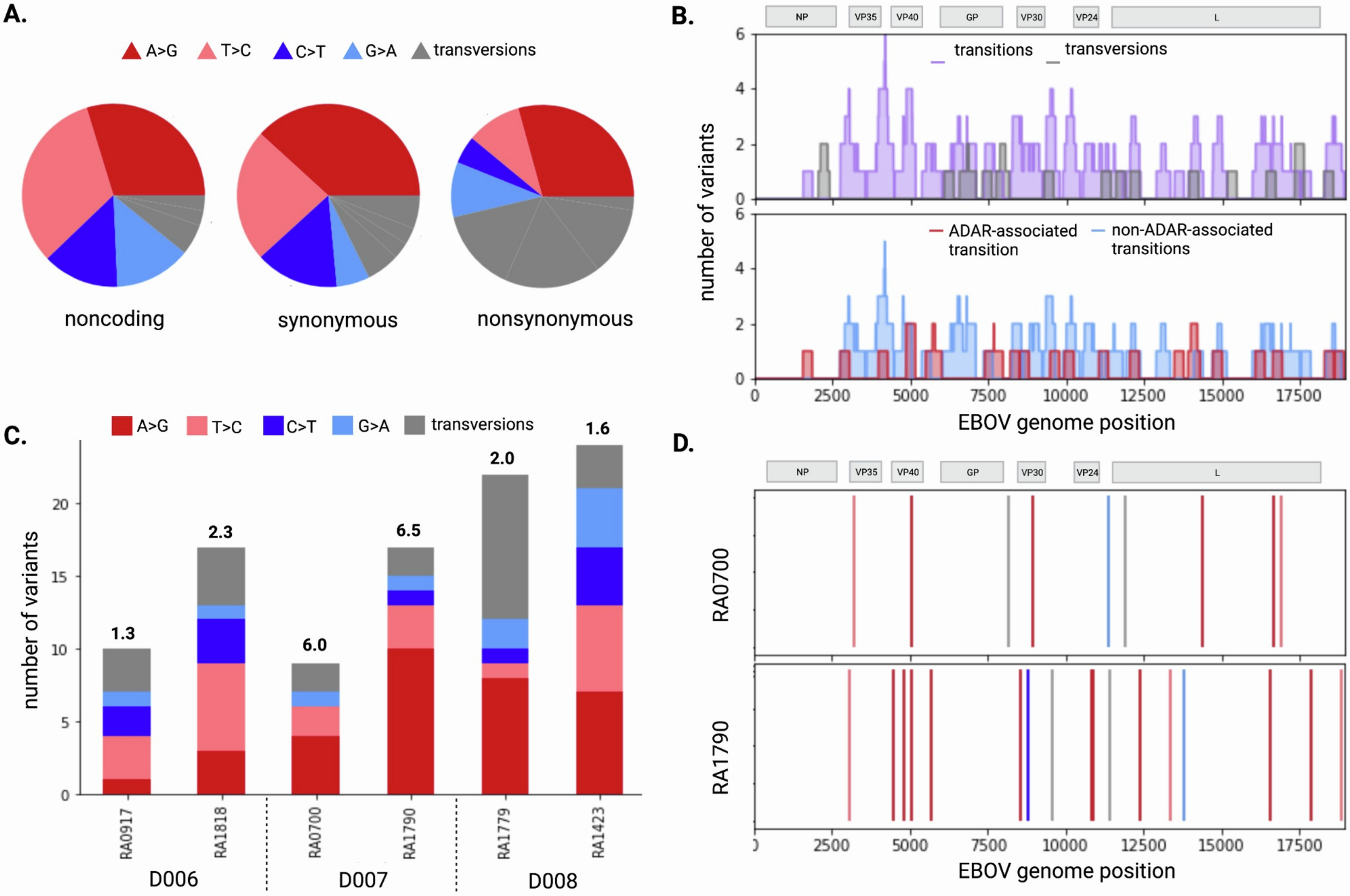

Figure S11. Mutation types across the sample set, related to figure 4. (A) Pie charts show relative amounts of each type of transition (labeled by color) and transversions (gray), separated by the mutation type. (B) Number of mutations that are transitions (purple) or transversions (gray) quantified by a 300 base pair sliding window across the EBOV genome. An EBOV gene map is above. (C) Stacked bar plots show the number of mutations that were each type of transition (labeled by color) and transversions (gray), separated by animal, ordered by DPI cohort. The bold number above shows the ratio of A-to-G and T-to-C mutations to C-to-T and G-to-A mutations, a marker of host RNA editing enzyme activity. (D) For two animals with the highest ratio of host RNA editing-associated mutations (RA0700 and RA1790), each mutation is represented by a vertical line along the EBOV genome on the x-axis. An EBOV gene map is above.

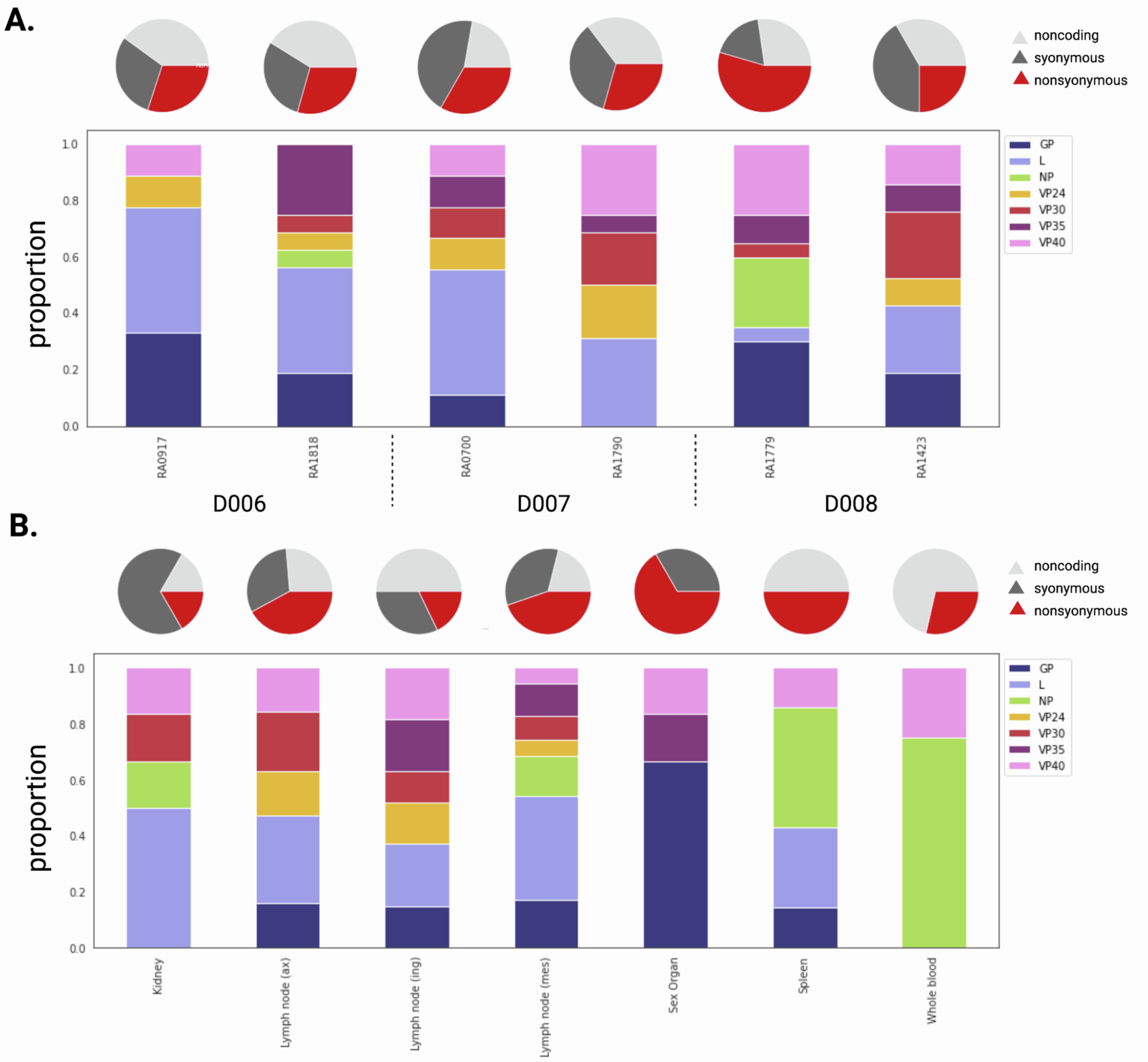

Figure S12. Mutations across animals and tissues, related to figure 4. (A) Proportion of nonsynonymous (red), synonymous (dark gray), and noncoding (light gray) variants across animals, ordered by cohort (top). Proportion of variants falling into each EBOV gene in each animal, ordered by cohort (bottom). (B) Proportion of nonsynonymous (red), synonymous (dark gray), and noncoding (light gray) variants within each tissue (top). Proportion of variants falling into each EBOV gene within each tissue (bottom).

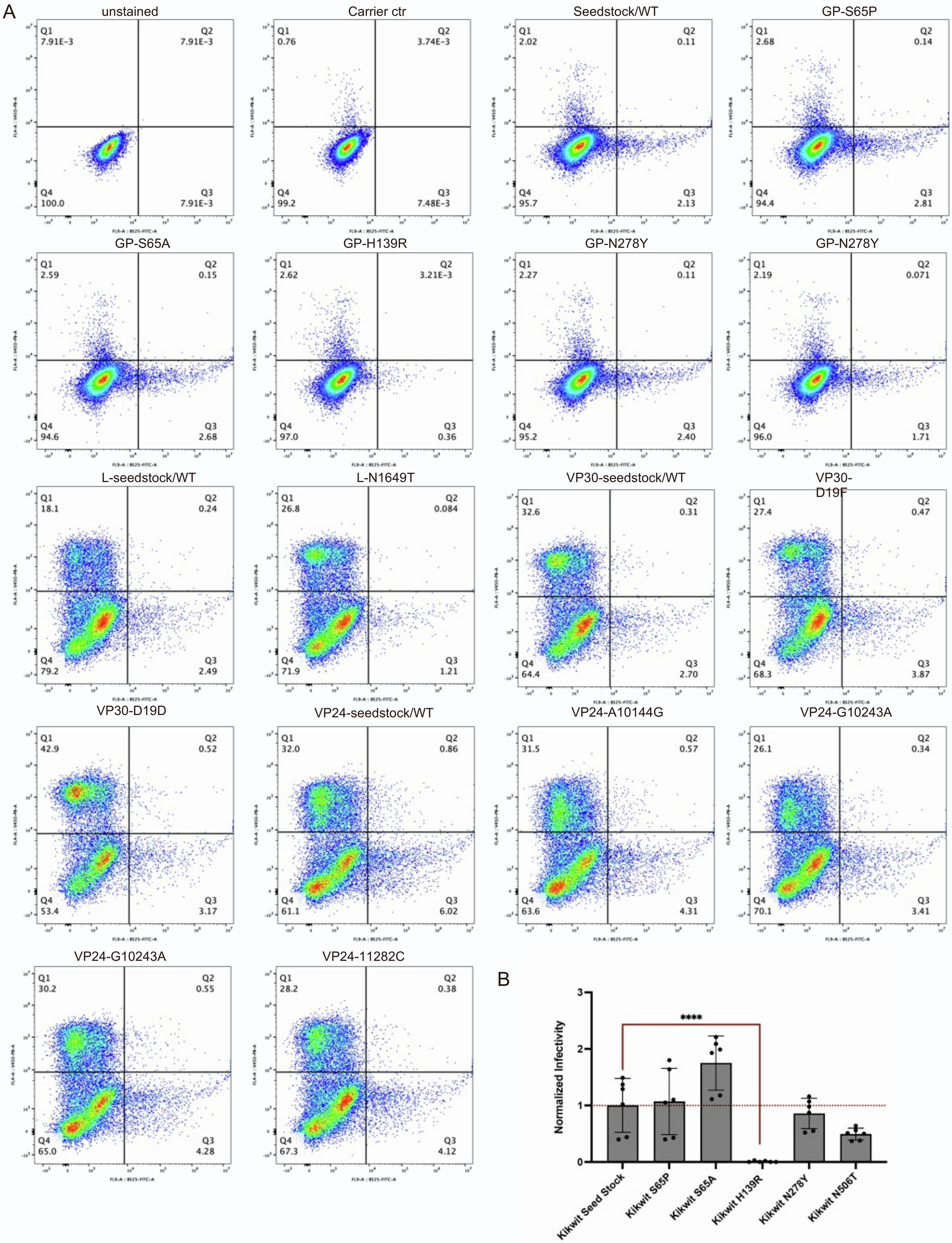

Figure S13. Functional characterization of viral variants, related to figure 5 (A) Gating strategy for GFP+ cells EBOV minigenome expression in HEK293T cells, representative data for n=2 independent biological replicates. (B) Quantification of fold difference in mCherry mRNA expression in HEK293T cells transduced with lentiviral virions pseudotyped with EBOV GP bearing the viral seed stock sequence or variants. Error bars represents standard deviation.

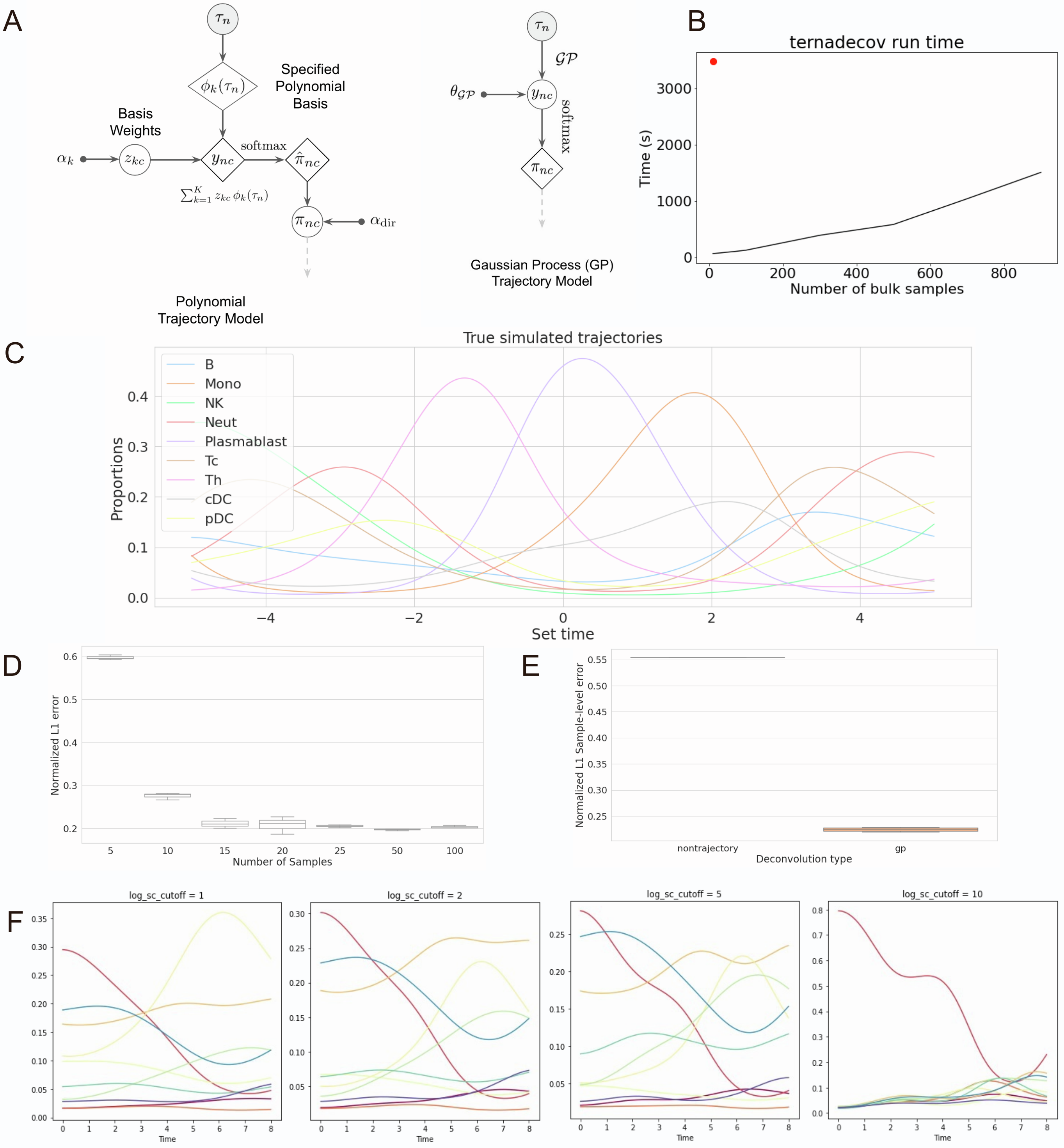

Figure S14. Utilizing Time-series Covariate Information for Enhanced RNA-seq Deconvolution, related to STAR methods (A) The parametric polynomial basis trajectory model (left), and the non-parametric Gaussian process trajectory model (right). The models are drop-in replacements as sub-models inside ternaDecov (see Figure 2D) (B) Execution time of ternaDecov (5,000 iterations, no GPU acceleration) for varying number of simulated samples using the GP trajectory module. The red point indicates the time required for deconvolution of the 10 samples from adrenal glands using MuSiC, shown for validation in the main text (C) Random simulated trajectory used for assessment of improvement in trajectory estimation as the number of sampled points increases (D) Normalized L1 trajectory error for trajectory estimation (at 1000 fixed points) using GP method from variable number of samples (3 replicates per point). Trajectory estimation improves as the number of samples increases (E) Normalized L1 sample-level error for the GP and non-trajectory (naive) model indicates that imposition of trajectory improves our ability to deconvolve the sample composition (F) Deconvolution results for adrenal tissues with increasing values of the log_sc_cutoff parameter (abundance cutoff for gene selection of the single-cell data) indicates robustness to parameter values.

Data S1: Pentacistronic Minigenome Assay plasmid sequences, related to STAR methods.

1. EBOV/Kikwit 5MG

CGGACACACAAAAAGAAAGAAGAATTTTTAGGATCTTTTGTGTGCGAATAACTATGAGGAAGATTAATAATTTTCC
TCTCATTGAAATTTATATCGGAATTTAAATTGAAATTGTTACTGTAATCACACCTGGTTTGTTTCAGAGCCACATCA
CAAAGATAGAGAACAACCTAGGTCTCTGAAGGGAGCAAGGGCATCAGTGTGCTCAGTTGAAAATCCCTTGTCAA
CATCTAGGTCTTATCACATCACAAGTCCCACCTCAGACTCTGCAGGGTGATCCAACAACCTTAATAGAAACATTAT
TGTTAAAGGACAGCATTAGTTCACAGTCAAACAAGCAAGATTGAGAATTAACCTTGGTTTTGAACTTGAATACTTA
GAGGATTGGAGATTCAACAACCCTAAAGCTTGGGGTAAAACATTGGAAATAGTTAAAAGACAAATTGCTCGGAAT
CACAACATTCCGAGTATGGTGAGCAAGGGCGAGGAGCTGTTCACCGGGGTGGTGCCCATCCTGGTCGAGCTGG
ACGGCGACGTAAACGGCCACAAGTTCAGCGTGTCCGGCGAGGGCGAGGGCGATGCCACCTACGGCAAGCTGA
CCCTGAAGTTCATCTGCACCACCGGCAAGCTGCCCGTGCCCTGGCCCACCCTCGTGACCACCTTGACCTACGG
CGTGCAGTGCTTCGCCCGCTACCCCGACCACATGAAGCAGCACGACTTCTTCAAGTCCGCCATGCCCGAAGGC
TACGTCCAGGAGCGCACCATCTTCTTCAAGGACGACGGCAACTACAAGACCCGCGCCGAGGTGAAGTTCGAGG
GCGACACCCTGGTGAACCGCATCGAGCTGAAGGGCATCGACTTCAAGGAGGACGGCAACATCCTGGGGCACAA
GCTGGAGTACAACTACAACAGCCACAAGGTCTATATCACCGCCGACAAGCAGAAGAACGGCATCAAGGTGAACT
TCAAGACCCGCCACAACATCGAGGACGGCAGCGTGCAGCTCGCCGACCACTACCAGCAGAACACCCCCATCGG
CGACGGCCCCGTGCTGCTGCCCGACAACCACTACCTGAGCACCCAGTCCGCCCTGAGCAAAGACCCCAACGAG
AAGCGCGATCACATGGTCCTGCTGGAGTTCGTGACCGCCGCCGGGATCACTCTCGGCATGGACGAGCTGTACA
AGTAAATGAGCATGGAACAATGGGATGATTCAACCGACAAATAGCTAACATTAGGTAGTCAAGGAACGAAAACAG
GAAGAATTTTTGATGTCTAAGGTGTGAATTATTATCACAATAAAAGTGATTCTTATTTTTGAATTTAAAGCTAGCTT
ATTATTACTAGCCGTTTTTCAAAGTCCAATTTGAGTCTTAATGCAAATAGGCGTTAAGCCACAGTTATAGCCATAA
TTGTAACTCAATATCCTAACTAGCGATTTATCTAAATTAAATTACATTATGCTTTTATAACTTACCTACTAGCCTACC
CAACATTTACACGATCATTTTATAATTAAGAAAAAACTAATGATGAAGATTAAAACCTTCATCATCCTTACGTCAAT
TGAATTCTCTAGCACTCGAAGCTTATTGTCCTCAATGTAAAAGAAAAGCTGGTCTAACAAGATGGTCTTCACACTC
GAAGATTTCGTTGGGGACTGGCGACAGACAGCCGGCTACAACCTGGACCAAGTCCTTGAACAGGGAGGTGTGT
CCAGTTTGTTTCAGAATCTCGGGGTGTCCGTAACTCCGATCCAAAGGATTGTCCTGAGCGGTGAAAATGGGCTG
AAGATCGACATCCATGTCATCATCCCGTATGAAGGTCTGAGCGGCGACCAAATGGGCCAGATCGAAAAAATTTTT
AAGGTGGTGTACCCTGTGGATGATCATCACTTTAAGGTGATCCTGCACTATGGCACACTGGTAATCGACGGGGT
TACGCCGAACATGATCGACTATTTCGGACGGCCGTATGAAGGCATCGCCGTGTTCGACGGCAAAAAGATCACTG
TAACAGGGACCCTGTGGAACGGCAACAAAATTATCGACGAGCGCCTGATCAACCCCGACGGCTCCCTGCTGTTC
CGAGTAACCATCAACGGAGTGACCGGCTGGCGGCTGTGCGAACGCATTCTGGCGTAAGCCCATCTTCCTTCCCT
CCGAAAGAGGCGACTAATAGCAGAGGCTTCAACTGCTGAACTATAGGGTACGTTACATTAATGATACACTTGTGA
GTATCAGCCCTGGATAATATAAGTCAATTAAACGACCAAGATAAAATTGTTCTTATCTCGCTAGCAGCTTAAAATA
TGAATGTAATAGGAGCTATATCTCTGACAGTATTATAATCAATCGTTATTAAGTAACCCAAACCAAAAGTGATGAA
GATTAAGAAAAACCTACCTCGACTGAGAGAGTGTTTTTTCATTAACCTTCATCTTGTAAACGTTGAGCAAAATTGT
TAAAAATATGAGGCGGGTTATATTACCTACTGCTCCTCCTGAATATATGGAGGCCATATACCCTGTCAGGTCAAA
TTCAACAATTGCTAGAGGTGGCAACAGCAATACAGGCTTCCTGACACCGGAGTCAGTCAATGGGGACACTCCAT
CGAATCCACTCAGGCCAATTGCCGATGACACCATCGACCATGCCAGCCACACACCAGGCAGTGTGTCATCAGCA
TTCATCCTTGAAGCTATGGTGAATGTCATATCGGGCCCCAAAGTGCTAATGAAGCAAATTCCAATTTGGCTTCCT
CTAGGTGTCGCTGATCAAAAGACCTACAGCTTTGACTCAACAACGGCCGCCATCATGCTTGCTTCATATACTATC
ACCCATTTCGGCAAGGCAACCAATCCACTTGTCAGAGTCAATCGGCTGGGTCCTGGAATCCCGGATCACCCCCT
CAGGCTCCTGCGAATTGGAAACCAGGCCTTCCTCCAGGAGTTCGTTCTTCCGCCAGTCCAACTACCCCAGTATT
TCACCTTTGATTTGACAGCACTCAAACTGATCACCCAACCACTGCCTGCTGCAACATGGACCGATGACACTCCAA
CAGGATCAAATGGAGCGTTGCGCCCAGGGATTTCATTTCATCCAAAACTTCGCCCCATTCTTTTACCCAACAAGA
GTGGGAAGAAGGGGAATAGTGCCGATCTAACATCTCCGGAGAAAATCCAAGCAATAATGACTTCACTCCAGGAC
TTTAAGATCGTTCCAATTGATCCAACCAAAAATATCATGGGAATCGAAGTGCCAGAAACTCTGGTCCACAAGCTG
ACCGGTAAGAAGGTGACTTCTAAAAATGGACAACCAATCATCCCTGTTCTTTTGCCAAAGTACATTGGTTTGGAC
CCGGTGGCTCCAGGAGACCTCACCATGGTAATCACACAGGATTGTGACACGTGTCATTCTCCTGCGAGTCTTCC
AGCTGTGATTGAGAAGTAATTGCAATAATTGACTCAGATCCAGTTTTACAGAATCTTCTCAGGGATAGTGATAACA
TCTATTTAGTAATCCGTCCATTAGAGGAGATACTTTTAATTGATCAATATACTAAAGGTGCTTTACACCATTGTCTT
TTTCCTCTCCTAAATGTAGAACTTAACAAAAGACTCATAATATACTTGTTTTTAAAAGATTGATTGATGAAAGATCA

TAACTAATAACATTACAAATAATCCTACTATAATCAATACGGTGATCCAAATGTTAATCTTTCTCATTGCAAATACT
CTTTGCCCTTATCCTCAAATTGCCTACATGCTTACATCTGAGGATAGCCAGTGTGACTTGGATTGGAGATGTGGA
GAAAAAATCGGGACCCATTTCTAGGTTGTTCACCATCCAAGTACAGACATTGCCCTTCTAATTAAGAAAAAATCG
GCGATGAAGATTAAGCCGACAGTGAGCGTAATCTTCATCTCTCTTAGATTATTTGTCCTCCAGAGTAGGGATCGT
CAGGTCCTTTTCAATCGTATAACCAAAATAAACTTCACTAGAAGGATATTGTGGGGCAACAACACAATGGGTGTT
ACAGGAATATTGCAGTTACCTCGTGATCGATTCAAGAGGACATCATTCTTTCTTTGGGTAATTATCCTTTTCCAAA
GAACATTTTCCATCCCACTTGGAGTCATCCACAATAGCACATTACAGGTTAGTGATGTCGACAAACTGGTTTGCC
GTGACAAACTGTCATCCACAAATCAATTGAGATCAGTTGGACTGAATCTCGAAGGGAATGGAGTGGCAACTGAC
GTGCCATCTGCAACTAAAAGATGGGGCTTCAGGTCCGGTGTCCCACCAAAGGTGGTCAATTATGAAGCTGGTGA
ATGGGCTGAAAACTGCTACAATCTTGAAATCAAAAAACCTGACGGGAGTGAGTGTCTACCAGCAGCGCCAGACG
GGATTCGGGGCTTCCCCCGGTGCCGGTATGTGCACAAAGTATCAGGAACGGGACCGTGTGCCGGAGACTTTGC
CTTCCACAAAGAGGGTGCTTTCTTCCTGTATGACCGACTTGCTTCCACAGTTATCTACCGAGGAACGACTTTCGC
TGAAGGTGTCGTTGCATTTCTGATACTGCCCCAAGCTAAGAAGGACTTCTTCAGCTCACACCCCTTGAGAGAGC
CGGTCAATGCAACGGAGGACCCGTCTAGTGGCTACTATTCTACCACAATTAGATATCAAGCTACCGGTTTTGGAA
CCAATGAGACAGAGTATTTGTTCGAGGTTGACAATTTGACCTACGTCCAACTTGAATCAAGATTCACACCACAGT
TTCTGCTCCAGCTGAATGAGACAATATATACAAGTGGGAAAAGGAGCAATACCACGGGAAAACTAATTTGGAAGG
TCAACCCCGAAATTGATACAACAATCGGGGAGTGGGCCTTCTGGGAAACTAAAAAAAACCTCACTAGAAAAATTC
GCAGTGAAGAGTTGTCTTTCACAGCTGTATCAAACAGAGCCAAAAACATCAGTGGTCAGAGTCCGGCGCGAACT
TCTTCCGACCCAGGGACCAACACAACAACTGAAGACCACAAAATCATGGCTTCAGAAAATTCCTCTGCAATGGTT
CAAGTGCACAGTCAAGGAAGGGAAGCTGCAGTGTCGCATCTGACAACCCTTGCCACAATCTCCACGAGTCCTCA
ACCCCCCACAACCAAACCAGGTCCGGACAACAGCACCCACAATACACCCGTGTATAAACTTGACATCTCTGAGG
CAACTCAAGTTGAACAACATCACCGCAGAACAGACAACGACAGCACAGCCTCCGACACTCCCCCCGCCACGAC
CGCAGCCGGACCCCTAAAAGCAGAGAACACCAACACGAGCAAGGGTACCGACCTCCTGGACCCCGCCACCACA
ACAAGTCCCCAAAACCACAGCGAGACCGCTGGCAACAACAACACTCATCACCAAGATACCGGAGAAGAGAGTG
CCAGCAGCGGGAAGCTAGGCTTAATTACCAATACTATTGCTGGAGTCGCAGGACTGATCACAGGCGGGAGGAG
AGCTCGAAGAGAAGCAATTGTCAATGCTCAACCCAAATGCAACCCTAATTTACATTACTGGACTACTCAGGATGA
AGGTGCTGCAATCGGACTGGCCTGGATACCATATTTCGGGCCAGCAGCCGAGGGAATTTACATAGAGGGGCTG
ATGCACAATCAAGATGGTTTAATCTGTGGGTTGAGACAGCTGGCCAACGAGACGACTCAAGCTCTTCAACTGTTC
CTGAGAGCCACAACCGAGCTACGCACCTTTTCAATCCTCAACCGTAAGGCAATTGATTTCTTGCTGCAGCGATG
GGGCGGCACATGCCACATTTTGGGACCGGACTGCTGTATCGAACCACATGATTGGACCAAGAACATAACAGACA
AAATTGATCAGATTATTCATGATTTTGTTGATAAAACCCTTCCGGACCAGGGGGACAATGACAATTGGTGGACAG
GATGGAGACAATGGATACCGGCAGGTATTGGAGTTACAGGCGTTATAATTGCAGTTATCGCTTTATTCTGTATAT
GCAAATTTGTCTTTTAGTAAGGCTGACTAAAACACTATATAACCTTCTACTTGATCACAATACTCCGTATACCTATT
ATCATATATTTAATCAAGACGATATCCTTTAAGACTTATTCAGTACTATAATCACTCTCGTTTCAAATTAATAAGATG
TGCATGATTGCCCTAATATATGAAGAGGTATGATACAACCCTAACAGTGACCAAAGAAAATCATAATCTCGTATCG
CTCGTAATATAACCTGCCAAGCATACCTCTTGCACAAAGTGATTCTTGTACACAAATAATGTTTTACCCTACAGGA
GGTAGCAACGATCCATCCCATCAAAAAATAAGTATTTTATGACTTACTAATGACCTCTTAAAATATTAAGAAAAACT
GACGGAACATAAATTCTTTCTGCTTCAAGTTGTGGAGGAGGTGTTTGGTATTGGCTATTGTTATATTACAATCAAT
AACAAGCTTGTAAAAATATTGTTCTTGTTTCAAGAGGTAGATTGTGGCTGGAAATGCTAAACTAATGATGAAGATT
AATGCGGAGGTCTGATAAGAATAAACCTTATTATTCAGATTAGGCCCCAAGAGGCATTCTTCATCTCCTTTTAGCA
AAGTACTATTTCAGGGTAGTCCAATTAGTGACACGTCTCTTAGCTGTATATCAGTCGCCCCTGAGATACGCCACA
AAAGTGTCTCTAAGCTAAATTGGTCTGTACACATCTCATACATTGTATTAGGGACAATAATATCTAATTGAACTTAG
CCGTTTAAAATTTAGTGCATAAATCTGGGCTAACTCCACCAGGTCAACTCCATTGGCTGAAAAGAAGCCTACCTA
CAACGAACATCACTTTGAGCGCCCTCACATTTAAAAAATAGGAACGTCGTTCCAACAATCGAGCGCAAGGTTTCA
AGGTTGAACTGAGAGTGTCTAGACAACAAAGTATCGATCCTCCAGACACCAAGCAAGACCTGAGAAAAAACCAT
GGCTAAAGCTACGGGACGATACAATCTAATATCGCCCAAAAAGGACCTGGAGAAAGGGGTTGTCTTAAGCGACC
TCTGTAACTTCTTAGTTAGCCAAACTATTCAGGGGTGGAAGGTTTATTGGGCTGGTATTGAGTTTGATGTGACTCA
CAAAGGAATGGCCCTATTGCAAAGACTGAAAACTAATGACTTTGCCCCTGCATGGTCAATGACAAGGAATCTCTT
TCCTCATTTATTTCAAAATCCGAATTCCACAATTGAATCACCGCTGTGGGCATTGAGAGTCATCCTTGCAGCAGG
GATACAAGACCAGCTGATTGACCAGTCTTTGATTGAACCCTTAGCAGGAGCCCTTGGTCTGATCTCTGATTGGCT
GCTAACAACCAACACTAACCATTTCAACATGCGAACACAACGTGTCAAGGAACAATTGAGCCTAAAAATGCTGTC
GTTGATTCGATCCAATATTCTCAAGTTTATTAACAAATTGGATGCTCTACATGTCGTGAACTACAACGGATTGTTG
AGCAGTATTGAAATTGGAACTCAAAATCATACAATCATCATAACTCGAACTAACATGGGTTTTCTGGTGGAGCTCC
AAGAACCCGACAAATCGGCAATGAACCGCAAGAAGCCTGGGCCGGCGAAATTTTCCCTCCTTCATGAGTCCACA

CTGAAAGCATTTACACAAGGATCCTCAACACGAATGCAAAGTTTGATTCTTGAATTTAATAGCTCTCTTGCTATCT
AACTAAGATGGAATACTTCATATTGAGCTAACTCATATATGCTGACTCAATAGTTATCTTGACATCTCTGCTTTCAT
AATCAGATATATAAGCATAATAAATAAATACGCATATTTCTTGATAATTTGTTTAACCACAGATAAATCCTCACTGT
AAGCCAGCTTCCAAGTTGACACCCTTACAAAAACCAGGACTCAGAATCCCTCAAATAAGAGATTCCAAGACAACA
TCATAGAATTGCTTTATCATATGAATAAGCATTTTATCACCAGAAATCTTATATACTAAATGGTTAATTGTAACTGAA
CCCGCAGGTCACATGTGTTAGGTTTCACAGATTTTATATATTACTAACTCTATACTCGTAATTAACATTAGATAAGT
AGATTAAGAAAAAAATTACCGTGCATAGTATCCTGATACTTGCAAAGGTTGGTTATCAACATACAGATTATAAAAA
ACTCATAAATTGCTCTCATACATCATATTGATCTGATTTCAATAAACAACTATTTAAATAACGAAAGGAGTCCCTAT
ATTATACACTATATTTAGCCTCTCTCCCTGCGTGATAATCAAAAAATTCACAATGCAGCATGTGTGACATATTAATG
CCGCAATGAATTTAACGCAACATAATAAACTCTGCACTCTTTATAATTAAGCTTTAACGAAAGGTCTGGGCTCATA
TTGTTATTGATATAATAATGTTATATCAATGTCCTGTCAGATGGAATAGTGTTTTGGTTGATAACACGACTTCTTAA
AACAAAATTGATCTTTAAGATTAAGTTTTTTATAATTATCATTACTTTAATTTATCGATTTGAAAATGGTAATAGCCTT
AATCTTTGTGTAAAATAAGAGATTAGGTGTAATAACTTTAACATTTTTGTCTAGTAAGTTACTATTTCATACAGAAT
GATAAAATTAAAAGAAAAGGCAGGACTGTAAAATCATAAATACCTTCTTTACAATATAGCAGACTAGATAATAATCT
TTGTGTTAATGATAATTAAGACATTGACCACGCTCATCAGAAGGCTCGCCAAAATAAACGTTGCAAAAAGGATTC
CTGGAAAAATGGTCGCACACAAAAATTTAAAAATAAATCTATTTCTTCTTTTTTGTGTGTCCTATAGTGAGTCGTAT
TAACCCGGGATCGATATCCCCCTGCATTAATGAATCGGCCAACGCGCGGGGAGAGGCGGTTTGCGTATTGGGC
GCTCTTCCGCTTCCTCGCTCACTGACTCGCTGCGCTCGGTCGTTCGGCTGCGGCGAGCGGTATCAGCTCACTC
AAAGGCGGTAATACGGTTATCCACAGAATCAGGGGATAACGCAGGAAAGAACATGTGAGCAAAAGGCCAGCAAA
AGGCCAGGAACCGTAAAAAGGCCGCGTTGCTGGCGTTTTTCCATAGGCTCCGCCCCCCTGACGAGCATCACAA
AAATCGACGCTCAAGTCAGAGGTGGCGAAACCCGACAGGACTATAAAGATACCAGGCGTTTCCCCCTGGAAGCT
CCCTCGTGCGCTCTCCTGTTCCGACCCTGCCGCTTACCGGATACCTGTCCGCCTTTCTCCCTTCGGGAAGCGTG
GCGCTTTCTCATAGCTCACGCTGTAGGTATCTCAGTTCGGTGTAGGTCGTTCGCTCCAAGCTGGGCTGTGTGCA
CGAACCCCCCGTTCAGCCCGACCGCTGCGCCTTATCCGGTAACTATCGTCTTGAGTCCAACCCGGTAAGACACG
ACTTATCGCCACTGGCAGCAGCCACTGGTAACAGGATTAGCAGAGCGAGGTATGTAGGCGGTGCTACAGAGTT
CTTGAAGTGGTGGCCTAACTACGGCTACACTAGAAGAACAGTATTTGGTATCTGCGCTCTGCTGAAGCCAGTTAC
CTTCGGAAAAAGAGTTGGTAGCTCTTGATCCGGCAAACAAACCACCGCTGGTAGCGGTGGTTTTTTTGTTTGCAA
GCAGCAGATTACGCGCAGAAAAAAAGGATCTCAAGAAGATCCTTTGATCTTTTCTACGGGGTCTGACGCTCAGT
GGAACGAAAACTCACGTTAAGGGATTTTGGTCATGAGATTATCAAAAAGGATCTTCACCTAGATCCTTTTAAATTA
AAAATGAAGTTTTAAATCAATCTAAAGTATATATGAGTAAACTTGGTCTGACAGTTACCAATGCTTAATCAGTGAG
GCACCTATCTCAGCGATCTGTCTATTTCGTTCATCCATAGTTGCCTGACTCCCCGTCGTGTAGATAACTACGATA
CGGGAGGGCTTACCATCTGGCCCCAGTGCTGCAATGATACCGCGAGACCCACGCTCACCGGCTCCAGATTTAT
CAGCAATAAACCAGCCAGCCGGAAGGGCCGAGCGCAGAAGTGGTCCTGCAACTTTATCCGCCTCCATCCAGTC
TATTAATTGTTGCCGGGAAGCTAGAGTAAGTAGTTCGCCAGTTAATAGTTTGCGCAACGTTGTTGCCATTGCTAC
AGGCATCGTGGTGTCACGCTCGTCGTTTGGTATGGCTTCATTCAGCTCCGGTTCCCAACGATCAAGGCGAGTTA
CATGATCCCCCATGTTGTGCAAAAAAGCGGTTAGCTCCTTCGGTCCTCCGATCGTTGTCAGAAGTAAGTTGGCC
GCAGTGTTATCACTCATGGTTATGGCAGCACTGCATAATTCTCTTACTGTCATGCCATCCGTAAGATGCTTTTCTG
TGACTGGTGAGTACTCAACCAAGTCATTCTGAGAATAGTGTATGCGGCGACCGAGTTGCTCTTGCCCGGCGTCA
ATACGGGATAATACCGCGCCACATAGCAGAACTTTAAAAGTGCTCATCATTGGAAAACGTTCTTCGGGGCGAAAA
CTCTCAAGGATCTTACCGCTGTTGAGATCCAGTTCGATGTAACCCACTCGTGCACCCAACTGATCTTCAGCATCT
TTTACTTTCACCAGCGTTTCTGGGTGAGCAAAAACAGGAAGGCAAAATGCCGCAAAAAAGGGAATAAGGGCGAC
ACGGAAATGTTGAATACTCATACTCTTCCTTTTTCAATATTATTGAAGCATTTATCAGGGTTATTGTCTCATGAGCG
GATACATATTTGAATGTATTTAGAAAAATAAACAAATAGGGGTTCCGCGCACATTTCCCCGAAAAGTGCCACCTG
ACGTCTAAGAAACCATTATTATCATGACATTAACCTATAAAAATAGGCGTATCACGAGGCCCTTTCGTCTCGCGC
GTTTCGGTGATGACGGTGAAAACCTCTGACACATGCAGCTCCCGGAGACGGTCACAGCTTGTCTGTAAGCGGAT
GCCGGGAGCAGACAAGCCCGTCAGGGCGCGTCAGCGGGTGTTGGCGGGTGTCGGGGCTGGCTTAACTATGCG
GCATCAGAGCAGATTGTACTGAGAGTGCACCATTCGACGCTCTCCCTTATGCGACTCCTGCATTAAGAAGCAGC
CCAGTAGTAGGTTGAGGCCGTTGAGCACCGCCGCCGCAAGGAATGGTGCAAGGAGATGGCGCCCAACAGTCC
CCCGGCCACGGGGCCTGCCACCATACCCACGCCGAAACAAGCGCTCATGAGCCCGAAGTGGCGAGCCCGATC
TTCCCCATCGGTGATGTCGGCGATATAGGCGCCAGCAACCGCACCTGTGGCGCCGGTGATGCCGGCCACGATG
CGTCCGGCGTAGAGGATCTGGCTAGCGATGACCCTGCTGATTGGTTCGCTGACCATTTCCGGGTGCGGGACGG
CGTTACCAGAAACTCAGAAGGTTCGTCCAACCAAACCGACTCTGACGGCAGTTTACGAGAGAGATGATAGGGTC
TGCTTCAGTAAGCCAGATGCTACACAATTAGGCTTGTACATATTGTCGTTAGAACGCGGCTACAATTAATACATAA
CCTTATGTATCATACACATACGATTTAGGTGACACTATAGAATACAAGCTTGCATGCCTGCAGGTCGACTCTAGA

GGATCTCGATCCGGATATAGTTCCTCCTTTCAGCAAAAAACCCCTCAAGACCCGTTTAGAGGCCCCAAGGGGTT
ATGCTAGTTATTGCTCAGCGGTGGCAGCAGCCAACTCAGCTTCCTTTCGGGCTTTGTTAGCAGCCGGATCCTTTT
TTTTGAGCTCTCCCTTAGCCATCCGAGTGGACGACGTCCTCCTTCGGATGCCCAGGTCGGACCGCGAGGAGGT
GGAGATGCCATGCCGACCC

2. EBOV/Kikwit NP-P2A-VP35

GACATTGATTATTGACTAGTTATTAATAGTAATCAATTACGGGGTCATTAGTTCATAGCCCATATATGGAGTTCCG
CGTTACATAACTTACGGTAAATGGCCCGCCTGGCTGACCGCCCAACGACCCCCGCCCATTGACGTCAATAATGA
CGTATGTTCCCATAGTAACGCCAATAGGGACTTTCCATTGACGTCAATGGGTGGAGTATTTACGGTAAACTGCCC
ACTTGGCAGTACATCAAGTGTATCATATGCCAAGTACGCCCCCTATTGACGTCAATGACGGTAAATGGCCCGCCT
GGCATTATGCCCAGTACATGACCTTATGGGACTTTCCTACTTGGCAGTACATCTACGTATTAGTCATCGCTATTAC
CATGGTGATGCGGTTTTGGCAGTACATCAATGGGCGTGGATAGCGGTTTGACTCACGGGGATTTCCAAGTCTCC
ACCCCATTGACGTCAATGGGAGTTTGTTTTGGCACCAAAATCAACGGGACTTTCCAAAATGTCGTAACAACTCCG
CCCCATTGACGCAAATGGGCGGTAGGCGTGTACGGTGGGAGGTCTATATAAGCAGAGCTCGTTTAGTGAACCG
TCAGATCGCCTGGAGACGCCATCCACGCTGTTTTGACCTCCATAGAAGACACCGGGACCGATCCAGCCTCCGG
AATGGATTCTCGTCCTCAGAAAGTCTGGATGACGCCGAGTCTCACTGAATCTGACATGGATTACCACAAGATCTT
GACAGCAGGTCTGTCCGTTCAACAGGGGATTGTTCGGCAAAGAGTCATCCCAGTGTATCAAGTAAACAATCTTG
AGGAGATTTGCCAACTTATCATACAGGCCTTTGAAGCAGGTGTTGATTTTCAAGAGAGTGCGGACAGTTTCCTTC
TCATGCTTTGTCTTCATCATGCGTACCAGGGAGATTACAAACTTTTCTTGGAAAGTGGCGCAGTCAAGTATTTGG
AAGGGCACGGGTTCCGTTTTGAAGTCAAGAAGCGTGATGGAGTGAAGCGCCTTGAGGAATTGCTGCCAGCAGT
ATCTAGTGGAAAAAACATTAAGAGAACACTTGCTGCCATGCCGGAAGAGGAGACAACTGAAGCTAATGCCGGTC
AGTTTCTCTCCTTTGCAAGTCTATTCCTTCCGAAATTGGTAGTAGGAGAAAAGGCTTGTCTTGAGAAGGTTCAAAG
GCAAATTCAAGTACATGCAGAGCAAGGACTGATACAATATCCAACAGCTTGGCAATCAGTAGGACACATGATGGT
GATTTTCCGTTTGATGCGAACAAATTTTTTGATCAAATTTCTCCTAATACACCAAGGGATGCACATGGTTGCCGGG
CATGATGCCAACGACGCTGTGATTTCAAATTCAGTGGCTCAAGCTCGTTTTTCAGGTTTATTGATTGTCAAAACAG
TACTTGATCATATCCTACAAAAGACAGAACGCGGAGTTCGTCTCCATCCTCTTGCAAGGACCGCCAAGGTAAAAA
ATGAGGTGAACTCCTTTAAGGCTGCACTCAGCTCCCTGGCCAAGCATGGAGAGTATGCTCCTTTCGCCCGACTT
TTGAACCTTTCTGGAGTAAATAATCTTGAGCATGGTCTTTTCCCTCAACTATCAGCAATTGCACTCGGAGTCGCCA
CAGCACACGGGAGTACCCTCGCAGGAGTAAATGTTGGAGAACAGTATCAACAACTCAGAGAGGCTGCCACTGA
GGCTGAGAAGCAACTCCAACAATACGCAGAGTCTCGCGAACTTGACCATCTTGGACTTGATGATCAGGAAAAGA
AAATTCTTATGAACTTCCATCAGAAAAAGAACGAAATCAGCTTCCAGCAAACAAACGCTATGGTAACTCTAAGAAA
AGAGCGCCTGGCCAAGCTGACGGAAGCTATCACTGCTGCGTCACTGCCCAAAACAAGTGGACATTACGATGATG
ATGACGACATTCCCTTTCCAGGACCCATCAATGATGACGACAATCCTGGCCATCAAGATGATGATCCAACTGACT
CACAAGATACGACCATTCCTGATGTGGTGGTTGATCCCGATGATGGAAGCTACGGCGAATACCAGAGTTACTCG
GAAAACGGCATGAATGCACCAGATGACTTGGTCCTATTCGATCTAGACGAGGACGATGAGGACACTAAGCCAGT
GCCTAATAGATCAACCAAGGGTGGACAACAGAAAAACAGTCAAAAGGGCCAGCATACAGAGGGCAGACAGACA
CAATCCAGGCCAACTCAAAATGTCCCAGGCCCTCACAGAACAATCCACCACGCCAGTGCTCCACTCACGGATAA
TGACAGAAGAAATGAACCCTCCGGCTCAACCAGCCCTCGCATGCTGACACCAATCAACGAAGAGGCAGACCCA
CTGGACGATGCCGACGACGAGACGTCTAGCCTTCCGCCCCTGGAGTCAGACGATGAAGAACAGGACAGGGAC
GGAACTTCCAACCGCACACCCACTGTCGCCCCACCGGCTCCCGTATACAGAGATCACTCTGAAAAGAGAGAACT
CCCGCAAGATGAGCAACAAGATCAGGACCACACTCAAGAGGCCAGGAACCAGGACAGTGACAACACCCAGCCA
GAACACTCTTTTGAGGAGATGTATCGCCACATTCTAAGATCACAGGGGCCATTTGATGCTGTTTTGTATTATCATA
TGATGAAGGATGAGCCTGTAGTTTTCAGTACCAGTGATGGCAAAGAGTACACGTATCCAGACTCCCTTGAAGAG
GAATATCCACCATGGCTCACTGAAAAAGAGGCTATGAATGAAGAGAATAGATTTGTTACATTGGATGGTCAACAA
TTTTATTGGCCGGTAATGAATCACAAGAATAAATTCATGGCAATCCTGCAACATCATCAGGGATCCGGCGCTACT
AACTTCAGCCTGCTGAAGCAGGCTGGAGACGTGGAGGAGAACCCTGGACCTATGACAACCAGAACAAAGGGCA
GGGGCCACACTGCGGCCACGACTCAAAACGACAGAATGCCAGGCCCTGAGCTTTCGGGCTGGATCTCTGAGCA
GCTAATGACCGGAAGAATTCCTGTAAGCGACATCTTCTGTGATATTGAGAACAATCCAGGATTATGCTACGCATC
CCAAATGCAACAAACCAAGCCAAACCCGAAGACGCGCAACAGTCAAACCCAAACGGACCCAATTTGCAATCATA
GTTTTGAGGAGGTAGTACAAACATTAGCTTCATTGGCTACTGTTGTGCAACAACAAACCATTGCATCAGAATCATT
AGAACAACGCATTACGAGTCTTGAGAATGGTCTAAAGCCAGTTTATGATATGGCTAAAACAATCTCCTCATTGAAC
AGGGTTTGTGCTGAGATGGTTGCAAAATATGATCTTCTGGTGATGACAACCGGTCGGGCAACAGCAACCGCTGC
GGCAACTGAGGCTTATTGGGCCGAACATGGTCAACCACCACCTGGACCATCACTTTATGAAGAAAGTGCAATTC
GGGGTAAGATTGAATCTAGAGATGAGACCGTCCCTCAAAGTGTTAGGGAGGCATTCAACAATCTAGACAGTACC
ACTTCACTAACTGAGGAAAATTTTGGGAAACCTGACATTTCGGCAAAGGATTTGAGAAACATTATGTATGATCACT
TGCCTGGTTTTGGAACTGCTTTCCACCAATTAGTACAAGTGATTTGTAAATTGGGAAAAGATAGCAACTCATTGGA
CATCATTCATGCTGAGTTCCAGGCCAGCCTGGCTGAAGGAGACTCTCCTCAATGTGCCCTAATTCAAATTACAAA
AAGAGTTCCAATCTTCCAAGATGCTGCTCCACCTGTCATCCACATCCGCTCTCGAGGTGACATTCCCCGAGCTTG
CCAGAAAAGCTTGCGTCCAGTCCCACCATCGCCCAAGATTGATCGAGGTTGGGTATGTGTTTTTCAGCTTCAAGA

TGGTAAAACACTTGGACTCAAAATTTGATCTAGAGGTAAGCCTATCCCTAACCCTCTCCTCGGTCTCGATTCTAC
GTAAGATCTAGAACTAGTGTCGACGCAAGGGTTCGATCCCTACCGGTTAGTAATGAGTTTAATCAACCTCTGGAT
TACAAAATTTGTGAAAGATTGACTGGTATTCTTAACTATGTTGCTCCTTTTACGCTATGTGGATACGCTGCTTTAAT
GCCTTTGTATCATGCTATTGCTTCCCGTATGGCTTTCATTTTCTCCTCCTTGTATAAATCCTGGTTGCTGTCTCTTT
ATGAGGAGTTGTGGCCCGTTGTCAGGCAACGTGGCGTGGTGTGCACTGTGTTTGCTGACGCAACCCCCACTGG
TTGGGGCATTGCCACCACCTGTCAGCTCCTTTCCGGGACTTTCGCTTTCCCCCTCCCTATTGCCACGGCGGAAC
TCATCGCCGCCTGCCTTGCCCGCTGCTGGACAGGGGCTCGGCTGTTGGGCACTGACAATTCCGTGGTGTTGTC
GGGGAAGCTGACGTCCTTTCCATGGCTGCTCGCCTGTGTTGCCACCTGGATTCTGCGCGGGACGTCCTTCTGCT
ACGTCCCTTCGGCCCTCAATCCAGCGGACCTTCCTTCCCGCGGCCTGCTGCCGGCTCTGCGGCCTCTTCCGCG
TCTTCGCCTTCGCCCTCAGACGAGTCGGATCTCCCTTTGGGCCGCCTCCCCGCAAACGGGGGAGGCTAACTGA
AACACGGAAGGAGACAATACCGGAAGGAACCCGCGCTATGACGGCAATAAAAAGACAGAATAAAACGCACGGG
TGTTGGGTCGTTTGTTCATAAACGCGGGGTTCGGTCCCAGGGCTGGCACTCTGTCGATACCCCACCGAGACCC
CATTGGGGCCAATACGCCCGCGTTTCTTCCTTTTCCCCACCCCACCCCCCAAGTTCGGGTGAAGGCCCAGGGCT
CGCAGCCAACGTCGGGGCGGCAGGCCCTGCCATAGCAGATCTGCGCAGCTGTTTGCAAAAGCCTAGGCCTCCA
AAAAAGCCTCCTCACTACTTCTGGAATAGCTCAGAGGCAGAGGCGGCCTCGGCCTCTGCATAAATAAAAAAAATT
AGTCAGCCATGGGGCGGAGAATGGGCGGAACTGGGCGGAGTTAGGGGCGGGATGGGCGGAGTTAGGGGCGG
GACTATGGTTGCTGACTAATTGAGATGCATGCTTTGCATACTTCTGCCTGCTGGGGAGCCTGGGGACTTTCCACA
CCTGGTTGCTGACTAATTGAGATGCATGCTTTGCATACTTCTGCCTGCTGGGGAGCCTGGGGACTTTCCACACC
CTAACTGACACACCAGCTGCATTAATGAATCGGCCAACGCGCGGGGAGAGGCGGTTTGCGTATTGGGCGCTCT
TCCGCTTCCTCGCTCACTGACTCGCTGCGCTCGGTCGTTCGGCTGCGGCGAGCGGTATCAGCTCACTCAAAGG
CGGTAATACGGTTATCCACAGAATCAGGGGATAACGCAGGAAAGAACATGTGAGCAAAAGGCCAGCAAAAGGC
CAGGAACCGTAAAAAGGCCGCGTTGCTGGCGTTTTTCCATAGGCTCCGCCCCCCTGACGAGCATCACAAAAATC
GACGCTCAAGTCAGAGGTGGCGAAACCCGACAGGACTATAAAGATACCAGGCGTTTCCCCCTGGAAGCTCCCT
CGTGCGCTCTCCTGTTCCGACCCTGCCGCTTACCGGATACCTGTCCGCCTTTCTCCCTTCGGGAAGCGTGGCG
CTTTCTCATAGCTCACGCTGTAGGTATCTCAGTTCGGTGTAGGTCGTTCGCTCCAAGCTGGGCTGTGTGCACGA
ACCCCCCGTTCAGCCCGACCGCTGCGCCTTATCCGGTAACTATCGTCTTGAGTCCAACCCGGTAAGACACGACT
TATCGCCACTGGCAGCAGCCACTGGTAACAGGATTAGCAGAGCGAGGTATGTAGGCGGTGCTACAGAGTTCTT
GAAGTGGTGGCCTAACTACGGCTACACTAGAAGAACAGTATTTGGTATCTGCGCTCTGCTGAAGCCAGTTACCTT
CGGAAAAAGAGTTGGTAGCTCTTGATCCGGCAAACAAACCACCGCTGGTAGCGGTGGTTTTTTTGTTTGCAAGC
AGCAGATTACGCGCAGAAAAAAAGGATCTCAAGAAGATCCTTTGATCTTTTCTACGGGGTCTGACGCTCAGTGGA
ACGAAAACTCACGTTAAGGGATTTTGGTCATGAGATTATCAAAAAGGATCTTCACCTAGATCCTTTTAAATTAAAA
ATGAAGTTTTAAATCAATCTAAAGTATATATGAGTAAACTTGGTCTGACAGTTACCAATGCTTAATCAGTGAGGCA
CCTATCTCAGCGATCTGTCTATTTCGTTCATCCATAGTTGCCTGACTCCCCGTCGTGTAGATAACTACGATACGG
GAGGGCTTACCATCTGGCCCCAGTGCTGCAATGATACCGCGAGACCCACGCTCACCGGCTCCAGATTTATCAG
CAATAAACCAGCCAGCCGGAAGGGCCGAGCGCAGAAGTGGTCCTGCAACTTTATCCGCCTCCATCCAGTCTATT
AATTGTTGCCGGGAAGCTAGAGTAAGTAGTTCGCCAGTTAATAGTTTGCGCAACGTTGTTGCCATTGCTACAGGC
ATCGTGGTGTCACGCTCGTCGTTTGGTATGGCTTCATTCAGCTCCGGTTCCCAACGATCAAGGCGAGTTACATG
ATCCCCCATGTTGTGCAAAAAAGCGGTTAGCTCCTTCGGTCCTCCGATCGTTGTCAGAAGTAAGTTGGCCGCAG
TGTTATCACTCATGGTTATGGCAGCACTGCATAATTCTCTTACTGTCATGCCATCCGTAAGATGCTTTTCTGTGAC
TGGTGAGTACTCAACCAAGTCATTCTGAGAATAGTGTATGCGGCGACCGAGTTGCTCTTGCCCGGCGTCAATAC
GGGATAATACCGCGCCACATAGCAGAACTTTAAAAGTGCTCATCATTGGAAAACGTTCTTCGGGGCGAAAACTCT
CAAGGATCTTACCGCTGTTGAGATCCAGTTCGATGTAACCCACTCGTGCACCCAACTGATCTTCAGCATCTTTTA
CTTTCACCAGCGTTTCTGGGTGAGCAAAAACAGGAAGGCAAAATGCCGCAAAAAAGGGAATAAGGGCGACACG
GAAATGTTGAATACTCATACTCTTCCTTTTTCAATATTATTGAAGCATTTATCAGGGTTATTGTCTCATGAGCGGAT
ACATATTTGAATGTATTTAGAAAAATAAACAAATAGGGGTTCCGCGCACATTTCCCCGAAAAGTGCCACCTGACG
TCGACGGATCGGGAGATCTCCCGATCCCCTATGGTGCACTCTCAGTACAATCTGCTCTGATGCCGCATAGTTAA
GCCAGTATCTGCTCCCTGCTTGTGTGTTGGAGGTCGCTGAGTAGTGCGCGAGCAAAATTTAAGCTACAACAAGG
CAAGGCTTGACCGACAATTGCATGAAGAATCTGCTTAGGGTTAGGCGTTTTGCGCTGCTTCGCGATGTACGGGC
CAGATATACGCGTT

3. **EBOV/Kikwit L**

GACATTGATTATTGACTAGTTATTAATAGTAATCAATTACGGGGTCATTAGTTCATAGCCCATATATGGAGTTCCG
CGTTACATAACTTACGGTAAATGGCCCGCCTGGCTGACCGCCCAACGACCCCCGCCCATTGACGTCAATAATGA
CGTATGTTCCCATAGTAACGCCAATAGGGACTTTCCATTGACGTCAATGGGTGGAGTATTTACGGTAAACTGCCC
ACTTGGCAGTACATCAAGTGTATCATATGCCAAGTACGCCCCCTATTGACGTCAATGACGGTAAATGGCCCGCCT
GGCATTATGCCCAGTACATGACCTTATGGGACTTTCCTACTTGGCAGTACATCTACGTATTAGTCATCGCTATTAC
CATGGTGATGCGGTTTTGGCAGTACATCAATGGGCGTGGATAGCGGTTTGACTCACGGGGATTTCCAAGTCTCC
ACCCCATTGACGTCAATGGGAGTTTGTTTTGGCACCAAAATCAACGGGACTTTCCAAAATGTCGTAACAACTCCG
CCCCATTGACGCAAATGGGCGGTAGGCGTGTACGGTGGGAGGTCTATATAAGCAGAGCTCGTTTAGTGAACCG
TCAGATCGCCTGGAGACGCCATCCACGCTGTTTTGACCTCCATAGAAGACACCGGGACCGATCCAGCCTCCGG
AATGGCTACACAACATACCCAATACCCGGACGCTAGGTTATCATCACCAATTGTATTGGACCAATGTGACCTAGT
CACTAGAGCTTGCGGGTTATATTCATCATACTCCCTTAATCCGCAACTACGCAACTGTAAACTCCCGAAACATATC
TACCGTTTGAAATACGATGTAACTGTTACCAAGTTCTTGAGTGATGTACCAGTGGCGACATTGCCCATAGATTTCA
TAGTCCCAATTCTTCTCAAGGCACTGTCAGGCAATGGGTTCTGTCCTGTTGAGCCGCGGTGCCAACAGTTCTTAG
ATGAAATCATTAAGTACACAATGCAAGATGCTCTCTTCTTGAAATATTATCTCAAAAATGTGGGTGCTCAAGAAGA
CTGTGTTGATGACCACTTTCAAGAGAAAATCTTATCTTCAATTCAGGGCAATGAATTTTTACATCAAATGTTTTTCT
GGTATGATCTGGCTATTTTAACTCGAAGGGGTAGATTAAATCGAGGAAACTCTAGATCAACATGGTTTGTTCATG
ATGATTTAATAGACATCTTAGGTTATGGGGACTATGTTTTTTGGAAGATCCCAATTTCAATGTTACCACTGAACAC
ACAAGGAATCCCCCATGCTGCTATGGACTGGTATCAGGCATCAGTATTCAAAGAAGCGGTTCAAGGGCATACAC
ACATTGTTTCTGTTTCTACTGCCGACGTCTTGATAATGTGCAAAGATTTAATTACATGTCGATTCAACACAACTCTA
ATCTCAAAAATAGCAGAGATTGAGGATCCAGTTTGTTCTGATTATCCCAATTTTAAGATTGTGTCTATGCTTTACCA
GAGCGGAGATTACTTACTCTCCATATTAGGGTCTGATGGGTATAAAATTATTAAGTTCCTCGAACCATTGTGCTTG
GCCAAAATTCAATTATGCTCAAAGTACACCGAGAGGAAGGGCCGATTCTTAACACAAATGCATTTAGCTGTAAAT
CACACCCTAGAAGAAATTACAGAAATGCGTGCACTAAAGCCTTCACAGGCTCAAAAGATCCGTGAATTCCATAGA
ACATTGATAAGGCTGGAGATGACGCCACAACAGCTTTGTGAGCTATTTTCCATTCAAAAACACTGGGGGCATCCT
GTGCTACATAGTGAAACAGCAATCCAAAAAGTTAAAAAACATGCTACGGTGCTAAAAGCATTACGCCCTATAGTG
ATTTTCGAGACATACTGTGTTTTTAAATATAGTATTGCCAAACATTATTTTGATAGTCAAGGGTCTTGGTACAGTGT
TACTTCAGATAGGAATCTAACACCAGGTCTTAATTCTTATATCAAAAGAAATCAATTCCCTCCGTTGCCAATGATTA
AAGAACTACTATGGGAATTTTACCACCTTGACCATCCTCCACTTTTCTCAACCAAAATTATTAGTGACTTAAGTATT
TTTATAAAAGACAGAGCTACTGCAGTAGAAAGGACATGCTGGGATGCAGTATTCGAGCCTAATGTTCTAGGATAT
AATCCACCTCACAAATTTAGTACTAAACGTGTACCGGAACAATTTTTAGAGCAAGAAAACTTTTCTATTGAGAATG
TTCTTTCCTACGCGCAAAAACTCGAGTATCTACTACCACAATATCGGAACTTTTCTTTCTCATTGAAAGAGAAAGA
GTTGAATGTAGGTAGAACCTTCGGAAAATTGCCTTATCCGACTCGCAATGTTCAAACACTTTGTGAAGCTCTGTTA
GCTGATGGTCTTGCTAAAGCATTTCCTAGCAATATGATGGTAGTTACGGAACGTGAGCAAAAAGAAAGCTTATTG
CATCAAGCATCATGGCACCACACAAGTGATGATTTTGGTGAACATGCCACAGTTAGAGGGAGTAGCTTTGTAACT
GATTTAGAGAAATACAATCTTGCATTTAGATATGAGTTTACAGCACCTTTTATAGAATATTGCAACCGTTGCTATG
GTGTTAAGAATGTTTTTAATTGGATGCATTATACAATCCCACAGTGTTATATGCATGTCAGTGATTATTATAATCCA
CCACATAACCTCACACTGGAAAATCGAGACAACCCCCCCGAAGGGCCTAGTTCATACAGGGGTCATATGGGAGG
GATTGAAGGACTGCAACAAAAACTCTGGACAAGTATTTCATGTGCTCAAATTTCTTTAGTTGAAATTAAGACTGGT
TTTAAGTTACGCTCAGCTGTGATGGGTGACAATCAGTGCATTACCGTTTTATCAGTCTTCCCCTTAGAGACTGAC
GCAGACGAGCAGGAACAGAGCGCCGAAGACAATGCAGCGAGGGTGGCCGCTAGCCTAGCAAAAGTTACAAGT
GCCTGTGGAATCTTTTTAAAACCTGATGAAACATTTGTACATTCAGGTTTTATCTATTTTGGAAAAAAACAATATTT
GAATGGGGTCCAATTGCCTCAGTCCCTTAAAACGGCTACAAGAATGGCACCATTGTCTGATGCAATTTTTGATGA
TCTTCAAGGGACCCTAGCTAGTATAGGCACTGCTTTTGAGCGATCCATCTCTGAGACACGACATATCTTTCCTTG
CAGGATAACCGCAGCTTTCCATACGTTTTTTTCGGTGAGAATCTTGCAATATCATCATCTCGGGTTCAATAAAGGT
TTTGACCTTGGACAGTTAACACTCGGTAAACCTCTGGATTTCGGAACAATATCATTGGCACTAGCGGTACCGCAG
GTGCTTGGAGGATTATCCTTCTTGAATCCTGAGAAATGTTTCTACCGGAATCTAGGAGATCCAGTTACCTCAGGT
TTATTCCAGTTAAAAACTTATCTCCGAATGATTGAGATGGATGATTTATTCTTACCTTTAATTGCAAAGAACCCTGG
GAACTGCACTGCCATTGACTTTGTGCTAAATCCTAGCGGATTAAATGTCCCTGGGTCGCAAGACTTAACTTCATTT
CTGCGCCAGATTGTACGCAGGACCATCACCCTAAGTGCGAAAAACAAACTTATTAATACCTTATTTCATGCGTCA
GCTGACTTCGAAGACGAAATGGTTTGTAAATGGCTATTATCATCAACTCCTGTTATGAGTCGTTTTGCGGCCGAT
ATCTTTTCACGCACGCCGAGCGGGAAGCGATTGCAAATTCTAGGATACCTGGAAGGAACACGCACATTATTAGC
CTCTAGGATCATCAACAATAATACAGAGACACCGGTTTTGGACAGACTGAGGAAAATAACATTGCAAAGGTGGAG
TCTGTGGTTTAGTTATCTTGATCATTGTGATAATATCCTGGCGGAGGCTTTAACCCAAATAACTTGCACAGTTGAT

TTAGCACAGATCCTGAGGGAATATTCATGGGCTCATATTTTAGAGGGAAGACCTCTTATTGGAGCCACACTCCCA
TGTATGATTGAGCAATTCAAAGTGTTTTGGCTGAAACCCTACGAACAATGTCCGCAGTGTTCAAATGCAAAGCAA
CCAGGTGGGAAACCATTCGTGTCAGTGGCAGTCAAGAAACATATTGTTAGTGCATGGCCTAACGCATCCCGACT
AAGCTGGACTATCGGGGATGGAATCCCTTACATTGGATCAAGGACAGAAGATAAGATAGGACAACCTGCTATTAA
ACCAAAATGTCCTTCCGCAGCCTTAAGAGAGGCCATTGAATTGGCGTCCCGTTTAACATGGGTAACTCAAGGCA
GTTCGAACAGTGACTTGCTAATAAAGCCATTTTTGGAAGCACGAGTAAATTTAAGTGTTCAAGAAATACTTCAAAT
GACCCCTTCACATTACTCAGGAAATATTGTTCACAGGTACAACGATCAATACAGTCCTCATTCTTTCATGGCCAAT
CGTATGAGTAATTCAGCAACGCGATTGATTGTTTCTACAAACACTTTAGGTGAGTTTTCAGGAGGTGGCCAGTCT
GCACGCGACAGCAATATTATTTTCCAGAATGTTATAAATTATGCAGTTGCACTGTTCGATATTAAATTTAGAAACA
CTGAGGCTACAGATATCCAATATAATCGTGCTCACCTTCATCTAACTAAGTGTTGCACCCGGGAAGTACCAGCTC
AGTATTTAACATACACATCTACATTGGATTTAGATTTAACAAGATACCGAGAAAACGAATTGATTTATGACAATAAT
CCTCTAAAAGGAGGACTCAATTGCAATATCTCATTCGATAACCCATTTTTCCAAGGTAAACAGCTGAACATTATAG
AAGATGATCTTATTCGACTGCCTCACTTATCTGGATGGGAGCTAGCCAAGACCATCATGCAATCAATTATTTCAGA
TAGCAACAATTCATCTACAGACCCAATTAGCAGTGGAGAAACAAGATCATTCACTACCCATTTCTTAACTTATCCC
AAGATAGGACTTCTGTACAGTTTTGGGGCCTTTGTAAGTTATTATCTTGGCAATACAATTCTTCGGACTAAGAAAT
TGACACTTGACAATTTTTTATATTACTTAACTACCCAAATTCATAATCTACCACATCGCTCATTGCGAATACTTAAG
CCAACATTCAAACATGCAAGCGTTATGTCACGGTTAATGAGTATTGATCCTCATTTTTCTATTTACATAGGCGGTG
CTGCAGGTGACAGAGGACTCTCAGATGCGGCCAGGTTATTTTTGAGAACGTCTATTTCATCTTTTCTTACATTTGT
AAAAGAATGGATAATTAATCGCGGAACAATTGTCCCTTTATGGATAGTATATCCGCTAGAGGGTCAAAACCCAAC
ACCTGTTAATAATTTTCTCTATCAGATCGTAGAACTGCTGGTGCATGATTCATCAAGACAACAGGCTTTAAAAACT
ACCATAAGTGATCATGTACATCCTCACGACAATCTTGTTTACACATGTAAGAGTACAGCCAGCAATTTCTTCCATG
CATCATTGGCGTACTGGAGGAGCAGGCACAGAAACAGCAACCGAAAATACTTGGCAAGAGACTCTTCAACTAGA
TCAAGCACAAACAACAGTGATGGTCATATTGAGAGAAGTCAAGAACAAACCACCAGAGATCCACATGATGGCACT
GAACGGAATCTAGTTCTACAAATGAGCCATGAAATAAAAAGAACGACAATTCCACAAGAAAACACGCACCAGGGT
CCGTCGTTCCAGTCCTTTCTAAGTTACTCTGCTTGTGGTACAGCAAATCCAAAACTAAATTTCGATCGATCGAGAC
ACAATGTGAAATCTCAGGATCATAACTCGGCATCCAAGAGGGAAGGTCATCAAATAATCTCACACCGTCTAGTCC
TACCTTTCTTTACATTATCTCAAGGGACACGCCAATTAACGTCATCTAATGAGTCACAGACCCAAGACGAGATATC
AAAGTACTTACGGCAATTGAGATCCGTCATTGATACCACAGTTTATTGTAGGTTTACCGGTATAGTCTCGTCCATG
CATTACAAACTTGATGAGGTCCTTTGGGAAATAGAGAGTTTTAAGTCGGCTGTGACGCTAGCAGAGGGAGAAGG
TGCTGGTGCCTTACTATTGATTCAGAAATACCAAGTTAAGACCTTATTTTTCAACACGCTAGCTACTGAGTCCAGT
ATAGAGTCAGAAATAGTATCAGGAATGACTACTCCTAGGATGCTTCTACCTGTTATGTCAAAATTCCATAATGACC
AAATTGAGATTATTCTTAACAACTCAGCAAGCCAAATAACAGACATAACAAATCCTACTTGGTTTAAAGACCAAAG
AGCAAGGCTACCTAAGCAAGTCGAGGTTATAACCATGGATGCAGAGACGACAGAGAATATAAACAGATCGAAAT
TGTACGAAGCTGTATATAAACTGATCTTACACCATATTGATCCCAGCGTATTGAAAGCAGTGGTCCTTAAAGTCTT
CCTAAGTGATACTGAGGGTATGTTATGGCTAAATGATAATTTAGCCCCGTTTTTTGCCACTGGTTATTTAATTAAG
CCAATAACGTCAAGTGCTAGATCTAGTGAGTGGTATCTTTGTCTGACGAACTTCTTATCAACTACACGAAAGATG
CCACACCAAAACCATCTCAGTTGTAAACAGGTAATACTTACGGCATTGCAACTGCAAATTCAACGGAGCCCATAC
TGGCTAAGTCATTTAACTCAGTATGCTGACTGTGATTTACATTTAAGTTATATCCGCCTTGGTTTTCCATCATTAGA
GAAAGTACTATACCACAGGTATAACCTCGTCGATTCAAAAAGAGGTCCACTAGTCTCTATCACTCAGCACTTAGC
ACATCTTAGAGCAGAGATTCGAGAATTAACTAATGATTATAATCAACAGCGACAAAGTCGAACTCAAACATATCAC
TTTATTCGTACTGCAAAAGGACGAATCACAAAACTAGTCAATGATTATTTAAAATTCTTTCTTATTGTGCAAGCATT
AAAACATAATGGGACATGGCAAGCTGAGTTTAAGAAATTACCAGAGTTGATTAGTGTGTGCAATAGGTTCTACCA
TATTAGAGATTGCAATTGTGAAGAACGTTTCTTAGTTCAAACCTTATATTTACATAGAATGCAGGATTCTGAAGTTA
AGCTTATCGAAAGGCTGACAGGGCTTCTGAGTTTATTTCCGGATGGTCTCTACAGGTTTGATTGATCTAGAGGTA
AGCCTATCCCTAACCCTCTCCTCGGTCTCGATTCTACGTAAGATCTAGAACTAGTGTCGACGCAAGGGTTCGATC
CCTACCGGTTAGTAATGAGTTTAATCAACCTCTGGATTACAAAATTTGTGAAAGATTGACTGGTATTCTTAACTAT
GTTGCTCCTTTTACGCTATGTGGATACGCTGCTTTAATGCCTTTGTATCATGCTATTGCTTCCCGTATGGCTTTCA
TTTTCTCCTCCTTGTATAAATCCTGGTTGCTGTCTCTTTATGAGGAGTTGTGGCCCGTTGTCAGGCAACGTGGCG
TGGTGTGCACTGTGTTTGCTGACGCAACCCCCACTGGTTGGGGCATTGCCACCACCTGTCAGCTCCTTTCCGGG
ACTTTCGCTTTCCCCCTCCCTATTGCCACGGCGGAACTCATCGCCGCCTGCCTTGCCCGCTGCTGGACAGGGG
CTCGGCTGTTGGGCACTGACAATTCCGTGGTGTTGTCGGGGAAGCTGACGTCCTTTCCATGGCTGCTCGCCTGT
GTTGCCACCTGGATTCTGCGCGGGACGTCCTTCTGCTACGTCCCTTCGGCCCTCAATCCAGCGGACCTTCCTTC
CCGCGGCCTGCTGCCGGCTCTGCGGCCTCTTCCGCGTCTTCGCCTTCGCCCTCAGACGAGTCGGATCTCCCTT
TGGGCCGCCTCCCCGCAAACGGGGGAGGCTAACTGAAACACGGAAGGAGACAATACCGGAAGGAACCCGCGC

TATGACGGCAATAAAAAGACAGAATAAAACGCACGGGTGTTGGGTCGTTTGTTCATAAACGCGGGGTTCGGTCC
CAGGGCTGGCACTCTGTCGATACCCCACCGAGACCCCATTGGGGCCAATACGCCCGCGTTTCTTCCTTTTCCCC
ACCCCACCCCCCAAGTTCGGGTGAAGGCCCAGGGCTCGCAGCCAACGTCGGGGCGGCAGGCCCTGCCATAGC
AGATCTGCGCAGCTGTTTGCAAAAGCCTAGGCCTCCAAAAAAGCCTCCTCACTACTTCTGGAATAGCTCAGAGG
CAGAGGCGGCCTCGGCCTCTGCATAAATAAAAAAAATTAGTCAGCCATGGGGCGGAGAATGGGCGGAACTGGG
CGGAGTTAGGGGCGGGATGGGCGGAGTTAGGGGCGGGACTATGGTTGCTGACTAATTGAGATGCATGCTTTGC
ATACTTCTGCCTGCTGGGGAGCCTGGGGACTTTCCACACCTGGTTGCTGACTAATTGAGATGCATGCTTTGCATA
CTTCTGCCTGCTGGGGAGCCTGGGGACTTTCCACACCCTAACTGACACACCAGCTGCATTAATGAATCGGCCAA
CGCGCGGGGAGAGGCGGTTTGCGTATTGGGCGCTCTTCCGCTTCCTCGCTCACTGACTCGCTGCGCTCGGTCG
TTCGGCTGCGGCGAGCGGTATCAGCTCACTCAAAGGCGGTAATACGGTTATCCACAGAATCAGGGGATAACGC
AGGAAAGAACATGTGAGCAAAAGGCCAGCAAAAGGCCAGGAACCGTAAAAAGGCCGCGTTGCTGGCGTTTTTC
CATAGGCTCCGCCCCCCTGACGAGCATCACAAAAATCGACGCTCAAGTCAGAGGTGGCGAAACCCGACAGGAC
TATAAAGATACCAGGCGTTTCCCCCTGGAAGCTCCCTCGTGCGCTCTCCTGTTCCGACCCTGCCGCTTACCGGA
TACCTGTCCGCCTTTCTCCCTTCGGGAAGCGTGGCGCTTTCTCATAGCTCACGCTGTAGGTATCTCAGTTCGGT
GTAGGTCGTTCGCTCCAAGCTGGGCTGTGTGCACGAACCCCCCGTTCAGCCCGACCGCTGCGCCTTATCCGGT
AACTATCGTCTTGAGTCCAACCCGGTAAGACACGACTTATCGCCACTGGCAGCAGCCACTGGTAACAGGATTAG
CAGAGCGAGGTATGTAGGCGGTGCTACAGAGTTCTTGAAGTGGTGGCCTAACTACGGCTACACTAGAAGAACAG
TATTTGGTATCTGCGCTCTGCTGAAGCCAGTTACCTTCGGAAAAAGAGTTGGTAGCTCTTGATCCGGCAAACAAA
CCACCGCTGGTAGCGGTGGTTTTTTTGTTTGCAAGCAGCAGATTACGCGCAGAAAAAAAGGATCTCAAGAAGAT
CCTTTGATCTTTTCTACGGGGTCTGACGCTCAGTGGAACGAAAACTCACGTTAAGGGATTTTGGTCATGAGATTA
TCAAAAAGGATCTTCACCTAGATCCTTTTAAATTAAAAATGAAGTTTTAAATCAATCTAAAGTATATATGAGTAAAC
TTGGTCTGACAGTTACCAATGCTTAATCAGTGAGGCACCTATCTCAGCGATCTGTCTATTTCGTTCATCCATAGTT
GCCTGACTCCCCGTCGTGTAGATAACTACGATACGGGAGGGCTTACCATCTGGCCCCAGTGCTGCAATGATACC
GCGAGACCCACGCTCACCGGCTCCAGATTTATCAGCAATAAACCAGCCAGCCGGAAGGGCCGAGCGCAGAAGT
GGTCCTGCAACTTTATCCGCCTCCATCCAGTCTATTAATTGTTGCCGGGAAGCTAGAGTAAGTAGTTCGCCAGTT
AATAGTTTGCGCAACGTTGTTGCCATTGCTACAGGCATCGTGGTGTCACGCTCGTCGTTTGGTATGGCTTCATTC
AGCTCCGGTTCCCAACGATCAAGGCGAGTTACATGATCCCCCATGTTGTGCAAAAAAGCGGTTAGCTCCTTCGG
TCCTCCGATCGTTGTCAGAAGTAAGTTGGCCGCAGTGTTATCACTCATGGTTATGGCAGCACTGCATAATTCTCT
TACTGTCATGCCATCCGTAAGATGCTTTTCTGTGACTGGTGAGTACTCAACCAAGTCATTCTGAGAATAGTGTATG
CGGCGACCGAGTTGCTCTTGCCCGGCGTCAATACGGGATAATACCGCGCCACATAGCAGAACTTTAAAAGTGCT
CATCATTGGAAAACGTTCTTCGGGGCGAAAACTCTCAAGGATCTTACCGCTGTTGAGATCCAGTTCGATGTAACC
CACTCGTGCACCCAACTGATCTTCAGCATCTTTTACTTTCACCAGCGTTTCTGGGTGAGCAAAAACAGGAAGGCA
AAATGCCGCAAAAAAGGGAATAAGGGCGACACGGAAATGTTGAATACTCATACTCTTCCTTTTTCAATATTATTGA
AGCATTTATCAGGGTTATTGTCTCATGAGCGGATACATATTTGAATGTATTTAGAAAAATAAACAAATAGGGGTTC
CGCGCACATTTCCCCGAAAAGTGCCACCTGACGTCGACGGATCGGGAGATCTCCCGATCCCCTATGGTGCACT
CTCAGTACAATCTGCTCTGATGCCGCATAGTTAAGCCAGTATCTGCTCCCTGCTTGTGTGTTGGAGGTCGCTGAG
TAGTGCGCGAGCAAAATTTAAGCTACAACAAGGCAAGGCTTGACCGACAATTGCATGAAGAATCTGCTTAGGGTT
AGGCGTTTTGCGCTGCTTCGCGATGTACGGGCCAGATATACGCGTT

4. EBOV/Kikwit VP30

GACATTGATTATTGACTAGTTATTAATAGTAATCAATTACGGGGTCATTAGTTCATAGCCCATATATGGAGTTCCG
CGTTACATAACTTACGGTAAATGGCCCGCCTGGCTGACCGCCCAACGACCCCCGCCCATTGACGTCAATAATGA
CGTATGTTCCCATAGTAACGCCAATAGGGACTTTCCATTGACGTCAATGGGTGGAGTATTTACGGTAAACTGCCC
ACTTGGCAGTACATCAAGTGTATCATATGCCAAGTACGCCCCCTATTGACGTCAATGACGGTAAATGGCCCGCCT
GGCATTATGCCCAGTACATGACCTTATGGGACTTTCCTACTTGGCAGTACATCTACGTATTAGTCATCGCTATTAC
CATGGTGATGCGGTTTTGGCAGTACATCAATGGGCGTGGATAGCGGTTTGACTCACGGGGATTTCCAAGTCTCC
ACCCCATTGACGTCAATGGGAGTTTGTTTTGGCACCAAAATCAACGGGACTTTCCAAAATGTCGTAACAACTCCG
CCCCATTGACGCAAATGGGCGGTAGGCGTGTACGGTGGGAGGTCTATATAAGCAGAGCTCGTTTAGTGAACCG
TCAGATCGCCTGGAGACGCCATCCACGCTGTTTTGACCTCCATAGAAGACACCGGGACCGATCCAGCCTCCGG
AATGGAAGCTTCATATGAGAGAGGACGCCCACGAGCTGCCAGACAGCATTCAAGGGATGGACACGACCACCAT
GTTCGAGCACGATCATCATCCAGAGAGAATTATCGAGGTGAGTACCGTCAATCAAGGAGCGCCTCACAAGTGCG
CGTTCCTACTGTATTTCATAAGAAGAGAGTTGAACCATTAACAGTTCCTCCAGCACCTAAAGACATATGTCCGAC
CTTGAAAAAAGGATTTTTGTGTGACAGTAGTTTTTGCAAAAAAGATCACCAGTTGGAGAGTTTAACTGATAGGGAA
TTACTCCTACTAATCGCCCGTAAGACTTGTGGATCAGTAGAACAACAATTAAATATAACTGCACCCAAGGACTCG
CGCTTAGCAAATCCAACGGCTGATGATTTCCAGCAAGAGGAAGGTCCAAAAATTACCTTGTTGACTCTGATCAAG
ACGGCAGAACACTGGGCGAGACAAGACATCAGAACCATAGAGGATTCAAAATTAAGAGCATTGTTGACTCTATGT
GCTGTGATGACGAGGAAATTCTCAAAATCCCAGCTGAGTCTTTTATGTGAGACACACCTAAGGCGTGAGGGGCT
TGGGCAAGATCAGGCAGAACCTGTTCTCGAAGTATATCAACGATTACACAGTGATAAAGGAGGCAGTTTTGAAG
CTGCACTATGGCAACAATGGGACCGACAATCCCTAATTATGTTTATCACTGCATTCTTGAATATCGCTCTCCAGTT
ACCGTGTGAAAGTTCTGCTGTCGTTGTTTCAGGGTTAAGAACATTGGTTCCTCAATCAGATAATGAGGAAGCTTC
AACCAACCCGGGGACATGCTCATGGTCTGATGAGGGTACCCCTTAATCTAGAGGTAAGCCTATCCCTAACCCTC
TCCTCGGTCTCGATTCTACGTAAGATCTAGAACTAGTGTCGACGCAAGGGTTCGATCCCTACCGGTTAGTAATGA
GTTTAATCAACCTCTGGATTACAAAATTTGTGAAAGATTGACTGGTATTCTTAACTATGTTGCTCCTTTTACGCTAT
GTGGATACGCTGCTTTAATGCCTTTGTATCATGCTATTGCTTCCCGTATGGCTTTCATTTTCTCCTCCTTGTATAAA
TCCTGGTTGCTGTCTCTTTATGAGGAGTTGTGGCCCGTTGTCAGGCAACGTGGCGTGGTGTGCACTGTGTTTGC
TGACGCAACCCCCACTGGTTGGGGCATTGCCACCACCTGTCAGCTCCTTTCCGGGACTTTCGCTTTCCCCCTCC
CTATTGCCACGGCGGAACTCATCGCCGCCTGCCTTGCCCGCTGCTGGACAGGGGCTCGGCTGTTGGGCACTGA
CAATTCCGTGGTGTTGTCGGGGAAGCTGACGTCCTTTCCATGGCTGCTCGCCTGTGTTGCCACCTGGATTCTGC
GCGGGACGTCCTTCTGCTACGTCCCTTCGGCCCTCAATCCAGCGGACCTTCCTTCCCGCGGCCTGCTGCCGGC
TCTGCGGCCTCTTCCGCGTCTTCGCCTTCGCCCTCAGACGAGTCGGATCTCCCTTTGGGCCGCCTCCCCGCAAA
CGGGGGAGGCTAACTGAAACACGGAAGGAGACAATACCGGAAGGAACCCGCGCTATGACGGCAATAAAAAGAC
AGAATAAAACGCACGGGTGTTGGGTCGTTTGTTCATAAACGCGGGGTTCGGTCCCAGGGCTGGCACTCTGTCG
ATACCCCACCGAGACCCCATTGGGGCCAATACGCCCGCGTTTCTTCCTTTTCCCCACCCCACCCCCCAAGTTCG
GGTGAAGGCCCAGGGCTCGCAGCCAACGTCGGGGCGGCAGGCCCTGCCATAGCAGATCTGCGCAGCTGTTTG
CAAAAGCCTAGGCCTCCAAAAAAGCCTCCTCACTACTTCTGGAATAGCTCAGAGGCAGAGGCGGCCTCGGCCTC
TGCATAAATAAAAAAAATTAGTCAGCCATGGGGCGGAGAATGGGCGGAACTGGGCGGAGTTAGGGGCGGGATG
GGCGGAGTTAGGGGCGGGACTATGGTTGCTGACTAATTGAGATGCATGCTTTGCATACTTCTGCCTGCTGGGGA
GCCTGGGGACTTTCCACACCTGGTTGCTGACTAATTGAGATGCATGCTTTGCATACTTCTGCCTGCTGGGGAGC
CTGGGGACTTTCCACACCCTAACTGACACACCAGCTGCATTAATGAATCGGCCAACGCGCGGGGAGAGGCGGT
TTGCGTATTGGGCGCTCTTCCGCTTCCTCGCTCACTGACTCGCTGCGCTCGGTCGTTCGGCTGCGGCGAGCGG
TATCAGCTCACTCAAAGGCGGTAATACGGTTATCCACAGAATCAGGGGATAACGCAGGAAAGAACATGTGAGCA
AAAGGCCAGCAAAAGGCCAGGAACCGTAAAAAGGCCGCGTTGCTGGCGTTTTTCCATAGGCTCCGCCCCCCTG
ACGAGCATCACAAAAATCGACGCTCAAGTCAGAGGTGGCGAAACCCGACAGGACTATAAAGATACCAGGCGTTT
CCCCCTGGAAGCTCCCTCGTGCGCTCTCCTGTTCCGACCCTGCCGCTTACCGGATACCTGTCCGCCTTTCTCCC
TTCGGGAAGCGTGGCGCTTTCTCATAGCTCACGCTGTAGGTATCTCAGTTCGGTGTAGGTCGTTCGCTCCAAGC
TGGGCTGTGTGCACGAACCCCCCGTTCAGCCCGACCGCTGCGCCTTATCCGGTAACTATCGTCTTGAGTCCAAC
CCGGTAAGACACGACTTATCGCCACTGGCAGCAGCCACTGGTAACAGGATTAGCAGAGCGAGGTATGTAGGCG
GTGCTACAGAGTTCTTGAAGTGGTGGCCTAACTACGGCTACACTAGAAGAACAGTATTTGGTATCTGCGCTCTGC
TGAAGCCAGTTACCTTCGGAAAAAGAGTTGGTAGCTCTTGATCCGGCAAACAAACCACCGCTGGTAGCGGTGGT
TTTTTTGTTTGCAAGCAGCAGATTACGCGCAGAAAAAAAGGATCTCAAGAAGATCCTTTGATCTTTTCTACGGGGT
CTGACGCTCAGTGGAACGAAAACTCACGTTAAGGGATTTTGGTCATGAGATTATCAAAAAGGATCTTCACCTAGA
TCCTTTTAAATTAAAAATGAAGTTTTAAATCAATCTAAAGTATATATGAGTAAACTTGGTCTGACAGTTACCAATGC
TTAATCAGTGAGGCACCTATCTCAGCGATCTGTCTATTTCGTTCATCCATAGTTGCCTGACTCCCCGTCGTGTAG

ATAACTACGATACGGGAGGGCTTACCATCTGGCCCCAGTGCTGCAATGATACCGCGAGACCCACGCTCACCGG
CTCCAGATTTATCAGCAATAAACCAGCCAGCCGGAAGGGCCGAGCGCAGAAGTGGTCCTGCAACTTTATCCGCC
TCCATCCAGTCTATTAATTGTTGCCGGGAAGCTAGAGTAAGTAGTTCGCCAGTTAATAGTTTGCGCAACGTTGTT
GCCATTGCTACAGGCATCGTGGTGTCACGCTCGTCGTTTGGTATGGCTTCATTCAGCTCCGGTTCCCAACGATC
AAGGCGAGTTACATGATCCCCCATGTTGTGCAAAAAAGCGGTTAGCTCCTTCGGTCCTCCGATCGTTGTCAGAA
GTAAGTTGGCCGCAGTGTTATCACTCATGGTTATGGCAGCACTGCATAATTCTCTTACTGTCATGCCATCCGTAA
GATGCTTTTCTGTGACTGGTGAGTACTCAACCAAGTCATTCTGAGAATAGTGTATGCGGCGACCGAGTTGCTCTT
GCCCGGCGTCAATACGGGATAATACCGCGCCACATAGCAGAACTTTAAAAGTGCTCATCATTGGAAAACGTTCTT
CGGGGCGAAAACTCTCAAGGATCTTACCGCTGTTGAGATCCAGTTCGATGTAACCCACTCGTGCACCCAACTGA
TCTTCAGCATCTTTTACTTTCACCAGCGTTTCTGGGTGAGCAAAAACAGGAAGGCAAAATGCCGCAAAAAAGGGA
ATAAGGGCGACACGGAAATGTTGAATACTCATACTCTTCCTTTTTCAATATTATTGAAGCATTTATCAGGGTTATTG
TCTCATGAGCGGATACATATTTGAATGTATTTAGAAAAATAAACAAATAGGGGTTCCGCGCACATTTCCCCGAAAA
GTGCCACCTGACGTCGACGGATCGGGAGATCTCCCGATCCCCTATGGTGCACTCTCAGTACAATCTGCTCTGAT
GCCGCATAGTTAAGCCAGTATCTGCTCCCTGCTTGTGTGTTGGAGGTCGCTGAGTAGTGCGCGAGCAAAATTTA
AGCTACAACAAGGCAAGGCTTGACCGACAATTGCATGAAGAATCTGCTTAGGGTTAGGCGTTTTGCGCTGCTTC
GCGATGTACGGGCCAGATATACGCGTT
