# Supplementary material for: Natural history of Ebola virus disease in rhesus monkeys shows viral variant emergence dynamics and tissue-specific host responses
Source: Cell Genom. 2023 Nov 21;3(12):100440. doi: 10.1016/j.xgen.2023.100440 (PMC10759212; doi:10.1016/j.xgen.2023.100440)
Supplement: Document S2. Article plus supplemental information [file mmc7.pdf]

Cell Genomics

Resource

Natural history of Ebola virus disease in rhesus monkeys shows viral variant emergence dynamics and tissue-specific host responses

Graphical abstract

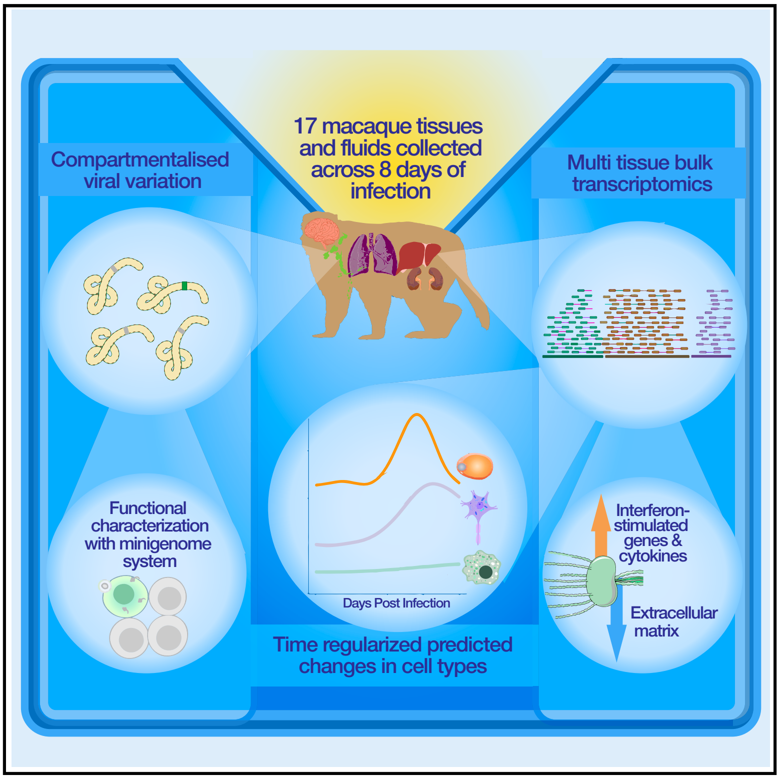

Highlights

- Ebola virus disseminates quickly and widely, likely mediated by infected monocytes
- Emerging viral variation impacts fitness and identifies compartmentalized infections
- Host-transcriptional signatures identify changes in vasculature-related genes
- Temporal deconvolution of bulk transcriptomes improves cell abundance estimates

Authors

Erica Normandin, Sergio Triana, Siddharth S. Raju, ..., Lisa E. Hensley, Pardis C. Sabeti, Katherine J. Siddle

Correspondence

strianas@broadinstitute.org (S.T.),
lisa.hensley@usda.gov (L.E.H.),
pardis@broadinstitute.org (P.C.S.),
katherine_siddle@brown.edu (K.J.S.)

In brief

Normandin et al. explored temporal and spatial host-pathogen dynamics in Ebola virus disease through a natural history study in rhesus monkeys. Bulk transcriptomics of over 400 samples highlighted clinically relevant changes in host gene expression and the emergence of viral mutations that were specific to individual tissues.

Normandin et al., 2023, Cell Genomics *3*, 100440
December 13, 2023 © 2023 The Authors.
https://doi.org/10.1016/j.xgen.2023.100440

CellPress

Cell Genomics

CellPress OPEN ACCESS

Resource

Natural history of Ebola virus disease in rhesus monkeys shows viral variant emergence dynamics and tissue-specific host responses

Erica Normandin,[1,2,29] Sergio Triana,[1,3,4,5,29,*] Siddharth S. Raju,[1,2,29] Tammy C.T. Lan,[1,6,29] Kim Lagerborg,[1,7] Melissa Rudy,[1] Gordon C. Adams,[1,8] Katherine C. DeRuff,[1] James Logue,[9] David Liu,[9] Daniel Strebinger,[1,10,11,12,13] Arya Rao,[14,15] Katelyn S. Messer,[1] Molly Sacks,[1] Ricky D. Adams,[9] Krisztina Janosko,[9] Dylan Kotliar,[1,2,3] Rickey Shah,[1,27]

(Author list continued on next page)

[1]Broad Institute of Harvard and MIT, Cambridge, MA 02142, USA
[2]Department of Systems Biology, Harvard Medical School, Boston, MA 02115, USA
[3]Harvard-MIT Division of Health Sciences and Technology, Massachusetts Institute of Technology, Cambridge, MA 02142, USA
[4]Department of Chemistry, Institute for Medical Engineering and Sciences (IMES), and Koch Institute for Integrative Cancer Research, MIT, Cambridge, MA 02142, USA
[5]Ragon Institute of MGH, Harvard, and MIT, Cambridge, MA 02139, USA
[6]Department of Molecular and Cellular Biology, Harvard University, Boston, MA, USA
[7]Harvard Program in Biological and Biomedical Sciences, Harvard Medical School, Boston, MA, USA
[8]Division of Infectious Diseases, Massachusetts General Hospital, Boston, MA 02114, USA
[9]Integrated Research Facility, Division of Clinical Research, National Institute of Allergy and Infectious Diseases, National Institutes of Health, Frederick, MD 21702, USA
[10]Howard Hughes Medical Institute, Chevy Chase, MD 20815-6789, USA
[11]McGovern Institute for Brain Research, Massachusetts Institute of Technology, Cambridge, MA 02139, USA
[12]Department of Brain and Cognitive Sciences, Massachusetts Institute of Technology, Cambridge, MA 02139, USA
[13]Department of Biological Engineering, Massachusetts Institute of Technology, Cambridge, MA 02139, USA
[14]Columbia University, New York, NY, USA
[15]Harvard/MIT MD-PhD Program, Harvard Medical School, Boston, MA 02115, USA
[16]Clinical Monitoring Research Program Directorate, Frederick National Laboratory for Cancer Research, Frederick, MD 21702, USA
[17]Department of Biochemistry, University of Colorado Boulder, Boulder, CO 80303, USA
[18]Life Sciences Department, Barcelona Supercomputing Center, 08034 Barcelona, Catalonia, Spain
[19]National Emerging Infectious Diseases Laboratories, Boston University, Boston, MA 02118, USA

(Affiliations continued on next page)

SUMMARY

Ebola virus (EBOV) causes Ebola virus disease (EVD), marked by severe hemorrhagic fever; however, the mechanisms underlying the disease remain unclear. To assess the molecular basis of EVD across time, we performed RNA sequencing on 17 tissues from a natural history study of 21 rhesus monkeys, developing new methods to characterize host-pathogen dynamics. We identified alterations in host gene expression with previously unknown tissue-specific changes, including downregulation of genes related to tissue connectivity. EBOV was widely disseminated throughout the body; using a new, broadly applicable deconvolution method, we found that viral load correlated with increased monocyte presence. Patterns of viral variation between tissues differentiated primary infections from compartmentalized infections, and several variants impacted viral fitness in a EBOV/Kikwit minigenome system, suggesting that functionally significant variants can emerge during early infection. This comprehensive portrait of host-pathogen dynamics in EVD illuminates new features of pathogenesis and establishes resources to study other emerging pathogens.

INTRODUCTION

Ebola virus disease (EVD), caused by infection with Ebola virus (EBOV), is among the most severe infectious diseases, with case fatality rates (CFRs) ranging from 40% to 50% in patients.[1] Since 1976, over 30 outbreaks of EVD have been recorded, claiming tens of thousands of lives.[2,3] While new vaccines[4] and treatments[5] are available, CFRs remain high, especially among patients who present late in the disease course.[6] Recent outbreaks of EVD in the Democratic Republic of the Congo and

This is an open access article under the CC BY license (http://creativecommons.org/licenses/by/4.0/).

Ian Crozier,[16] John L. Rinn,[17] Marta Melé,[18] Anna N. Honko,[9,19,28] Feng Zhang,[1,10,11,12,13] Mehrtash Babadi,[1] Jeremy Luban,[1,5,20] Richard S. Bennett,[9] Alex K. Shalek,[1,3,4,5] Nikolaos Barkas,[1,30] Aaron E. Lin,[1,21,22,23,30] Lisa E. Hensley,[9,30,*] Pardis C. Sabeti,[1,10,24,25,30,*] and Katherine J. Siddle[1,26,30,31,*]
[20]Program in Molecular Medicine, University of Massachusetts Chan Medical School, Worcester, MA 01655, USA
[21]Harvard Program in Virology, Harvard Medical School, Boston, MA 02115, USA
[22]Department of Molecular Biology, Princeton University, Princeton, NJ 08544, USA
[23]Lewis-Sigler Institute for Integrative Genomics, Princeton University, Princeton, NJ 08544, USA
[24]Department of Organismic and Evolutionary Biology, Harvard University, Cambridge, MA 02138, USA
[25]Department of Immunology and Infectious Diseases, Harvard T.H. Chan School of Public Health, Boston, MA 02115, USA
[26]Department of Molecular Microbiology and Immunology, Brown University, Providence, RI 02912, USA
[27]Present address: Noorda College of Osteopathic Medicine, Provo, UT, USA
[28]Present address: Apriori Bio, Inc., Cambridge, MA, USA
[29]These authors contributed equally
[30]These authors contributed equally
[31]Lead contact
*Correspondence: strianas@broadinstitute.org (S.T.), lisa.hensley@usda.gov (L.E.H.), pardis@broadinstitute.org (P.C.S.), katherine_siddle@brown.edu (K.J.S.)
https://doi.org/10.1016/j.xgen.2023.100440

Uganda and of other filovirus diseases, such as Marburg virus disease, underscore the importance of addressing filovirus threats. EVD is a prototypical viral hemorrhagic fever (VHF) with clinical manifestations including fever, severe gastrointestinal involvement, hemodynamic dysfunction, and multiorgan failure leading to death.[7] Notably, the host-pathogen determinants of this severity remain relatively obscure, and we lack comprehensive insight into the molecular pathobiology underlying severe EVD.

Genomic technologies let us better understand the molecular basis of infection, but their application has been centered on a few well-studied pathogens. Transcriptomic approaches in particular enable quantification of host transcripts and pathogen sequences, shedding light on relevant host factors, tissue pathologies, cellular targets of infection, and emerging genetic variation.[8–11] Comparative analyses of these signals between pathogens and populations can identify pathogen-agnostic and pathogen-specific responses, thereby indicating pathways of potential evolutionary and therapeutic significance.[12] Despite the important roles genomics and transcriptomics have played in our understanding of diseases, including coronavirus disease 2019 (COVID-19),[8–11] many severe viral threats have not been studied as extensively, in particular high-containment pathogens. Thus, there is a need for improved datasets and analytical methods integrating transcriptomics data to build a comprehensive understanding of molecular factors involved in diverse pathologies.

Previous studies of EBOV infection in non-human primate (NHP) models have largely focused on immune-related organs, with limited temporal or spatial resolution and overlooking pathogen dynamics. These studies have found that EVD is characterized by lymphocyte depletion and reduction in platelet counts,[7] while interferon-stimulated genes (ISGs), pro-inflammatory cytokines, and apoptosis-related genes have been identified as blood biomarkers that predict EVD severity and fatality.[13–15] An extended time course further identified early and conserved blood transcriptional responses,[16] with tissue-specific and temporal-specific gene expression changes observed in some solid tissues.[17] Single-cell RNA sequencing (scRNA-seq) and protein quantification by mass cytometry (CyTOF) of peripheral immune cells revealed emergency myelopoiesis and suppression of antiviral responses in infected cells.[18] RNA viruses, including EBOV, have a high mutation rate, allowing better resolution of inter-tissue viral spread and evolution. Emerging variations may allow the virus to better infect and replicate in a host;[19] biologically meaningful EBOV variants have emerged during animal studies[20] and recent outbreaks,[21,22] and varying levels of evolutionary constraint and adaptive potential have been described across the viral genome.[23] In patients, these variants are generally identified from blood, which likely reflects only a subset of viral diversity as tissues present different selective pressures.[24–27] Determining the shared and specific host dynamics across tissues and associating them with the corresponding viral dynamics promises to yield a more holistic view of disease progression.

Here, we present the first comprehensive spatiotemporal characterization of host and viral dynamics in a key NHP model of severe EVD. This dataset—the largest of its kind for any maximum-containment pathogen—provides novel insights into the establishment and progression of EVD and a rich resource for understanding host-pathogen interactions. To explore this dataset, we developed and applied ternaDecov, a computational tool to infer cell type proportions from bulk RNA-seq datasets with continuous covariates, and demonstrated its broader applicability. This study elucidates global and tissue-specific changes that may contribute to pathogenesis and illuminates potential routes of viral adaptation, circulation, and compartmentalization in peripheral tissues.

RESULTS

Multiorgan RNA-seq of rhesus monkeys with EVD shows widespread viral distribution and transcriptional changes

We established an extensive viral genomic and host transcriptomic dataset from a natural history study in 21 NHPs exposed to a lethal dose of EBOV. In this study, described in depth previously,[18,28] rhesus monkeys were sacrificed at baseline or 3–8 days post infection (DPI). Over 400 bulk RNA samples were collected at necropsy from 14 solid tissues and 3 tissue fluids (Figure 1A). Additionally, blood draws on alternate days were collected for a subset of animals. We quantified viral load

CellPress
OPEN ACCESS

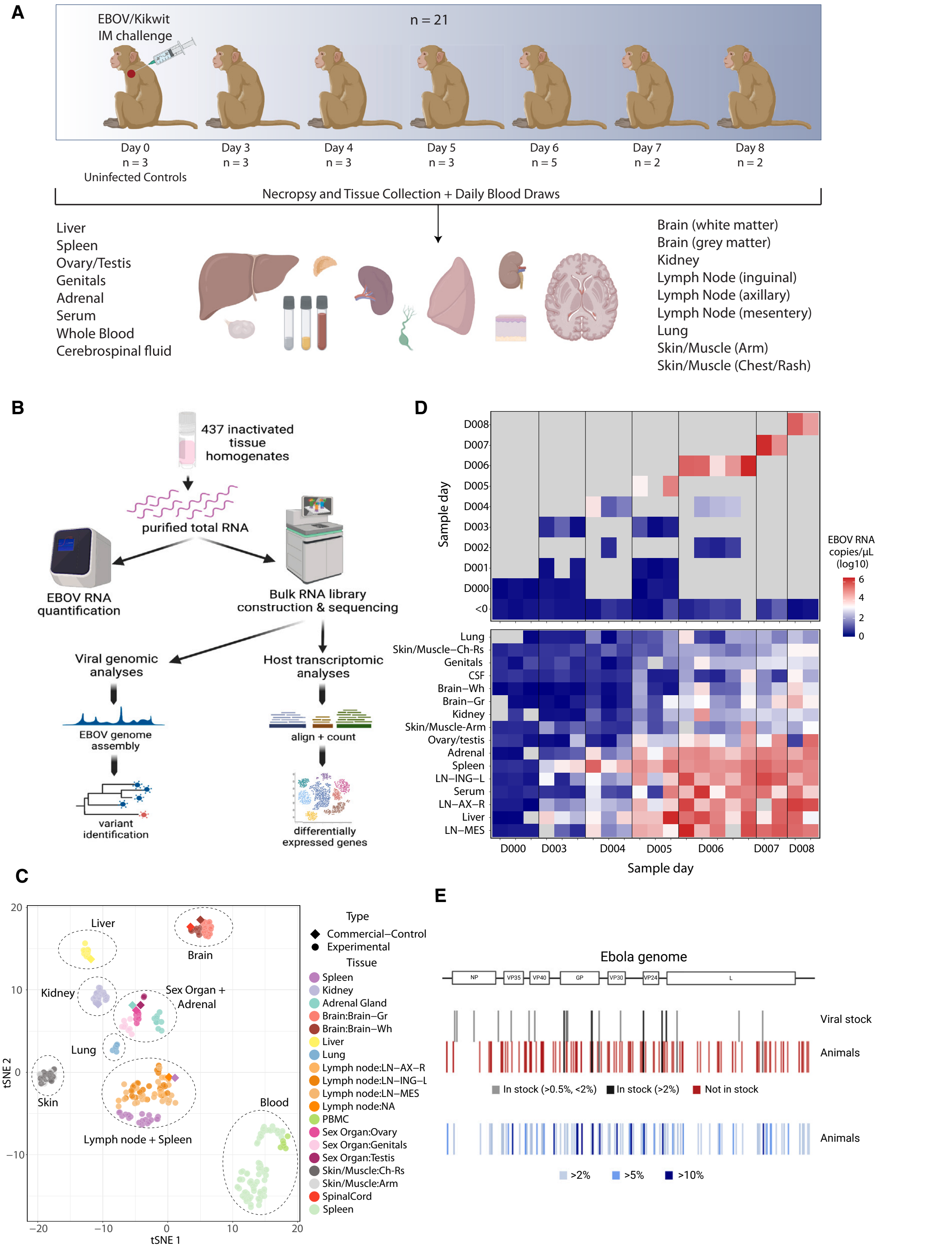

(legend on next page)

by qRT-PCR and attempted bulk RNA-seq on all samples (Figures 1B and 1C).

We observed high EBOV viral loads across fluids and tissues, indicating widespread viral dissemination (Figure 1D and Table S1). Viral loads were under a detectable threshold across tissues in uninfected animals but ranged from undetected to greater than 10^6 copies/μL in EBOV-exposed animals and were detectable in all tissues by 6 DPI. Viral loads were generally highest in the blood, serum, liver, lymph nodes, spleen, and adrenal gland. Viral loads in some tissues, such as kidney, skin, ovary/testis, and brain, were high in select animals after 6 DPI by qRT-PCR and sequencing-based viral read counts, which were highly correlated (Figure S1).

We obtained high-quality sequencing data from over 300 samples despite variable RNA quality, likely arising from challenges intrinsic to biosafety level 4 (BSL-4) containment conditions. We employed rigorous filtering and quality control methods to ensure the accuracy of this large dataset (Table S2). Briefly, we removed 13 samples that had insufficient total reads (<0.5 million reads), and eight additional samples that did not match the expected animal or tissue from NHP genotype fingerprinting, chromosome X:Y read ratios, or dimensionality reduction clustering (Figure S2). Host gene expression patterns across the sample set were driven primarily by the tissue identity (Figure 1C), and within each tissue group, host expression clustering patterns were driven by DPI (Figures S3 and S4). We assembled complete EBOV genomes from many tissues and identified variants in samples with high coverage depth (Figure 1E).

Host-virus analysis, using time-regularized deconvolution, reveals the contribution of direct infection and monocyte infiltration to tissue-specific viral loads and host responses

The host and virus data from this study provide a spatiotemporal picture of how EBOV establishes infection and spreads to multiple organ systems. Viral loads increased over time across all tissues, but the rate of increase differed (Figure 2A). Spleen and liver had the sharpest rise in viral load; these tissues were likely the primary sites of infection and replication after intramuscular exposure, putatively seeding infections throughout the body.[14,29,30] Lymph nodes, whole blood, and serum had high terminal viral loads (~10^5 copies/μL) but peaked later in infection (Figure 2A); these tissues likely accumulated infected cells. Other tissues (including brain, ovary/testis, skin, lung, kidney, and adrenal) had generally lower peak viral loads (<10^3 copies/μL) and slower rates of increase in viral RNA burden. In most tissues, we found that several host genes were correlated with viral RNA load. The top genes that correlated with viral load were interferon gamma and alpha ISGs (such as *CXCL10/11*, *IF16*, and *IFI27*) and those thought to be involved in viral defense (*KCNH*, *OASL*, and *OAS2*) (Figure 2B). The top genes anticorrelated with viral load included epigenetic and cell division-related genes, such as a H3K27 methyltransferase (*EZH1*) and a Yippee-like protein (*YPEL*) as well as a cell adhesion protein (*NCAM1*) involved in cell-matrix interactions and expansion of lymphocytes.[31]

We sought to further determine the factors driving differences in viral load across tissues. The viral load of a given tissue is determined by the efficiency with which EBOV infects and spreads within that tissue, the propensity of infected monocytes—the main infected immune cell population *in vivo*[18]—to infiltrate the tissue during infection, and/or the virus load present in circulating blood. We noted that the expression of canonical monocyte genes demonstrated a trend toward positive correlation with viral load in most tissues (Figure 2C) but not in tissues in which monocytes/monocyte-derived-macrophages are either normally abundant (blood and spleen) or a low viral load is detected (brain). We observed no consistent correlation (correlation < 0.45) between non-monocyte blood cell marker genes and viral load (Figure S5), suggesting that recruitment of infected monocytes is a significant driver of the viral load. This finding led us to investigate the role that intra-tissue changes in cell type proportion may play during pathogenesis.

Despite the availability of several deconvolution methods, which allow inference of cell type composition in bulk RNA-seq samples based on an scRNA-seq reference set,[32–35] most approaches are computationally inefficient. Furthermore, existing approaches provide only single-point estimates and do not use continuous covariates (such as time, age, developmental stage, or location) that are common features of large sequencing datasets. To address these limitations, we developed and applied a novel computational method to characterize tissue-specific changes in cell type proportions over the course of disease. We reasoned that continuous processes result in smooth trajectories that can simultaneously improve deconvolution (by sharing information between samples in close temporal proximity) and provide more information about the underlying biological process by inferring a specific parametric form of the cellular change trajectory. In our generalizable model for trajectory-based deconvolution, ternaDecov (temporal RNA deconvolution), the cellular proportions at each data point for every sample are drawn from a continuous function (Figure 2D). The form of the continuous function is not fixed and can be derived from alternative parametric and non-parametric trajectory models (STAR Methods).

Figure 1. Study overview

(A) Description of the animal study and dataset, including the number of animals, time points, and samples collected.

(B) Schematization of study design and experimental and analytical workflow.

(C) t-distributed stochastic neighbor embedding (tSNE) plot of transcriptional signatures, demonstrating that unique tissues cluster together and with commercial controls of the same type.

(D) Viral load across time in whole blood (top) and across tissues and other fluids at necropsy (bottom) for each animal, ordered by time between infection and necropsy. Colors represent viral RNA as log10(copies/μL), as assessed by qRT-PCR; gray represents no data.

(E) Viral variants across the EBOV genome identified in infecting viral stock and infected animals. Variants, designated by lines, are colored by their presence in stock (top) and frequency in infected animals (bottom).

Images were created with BioRender.

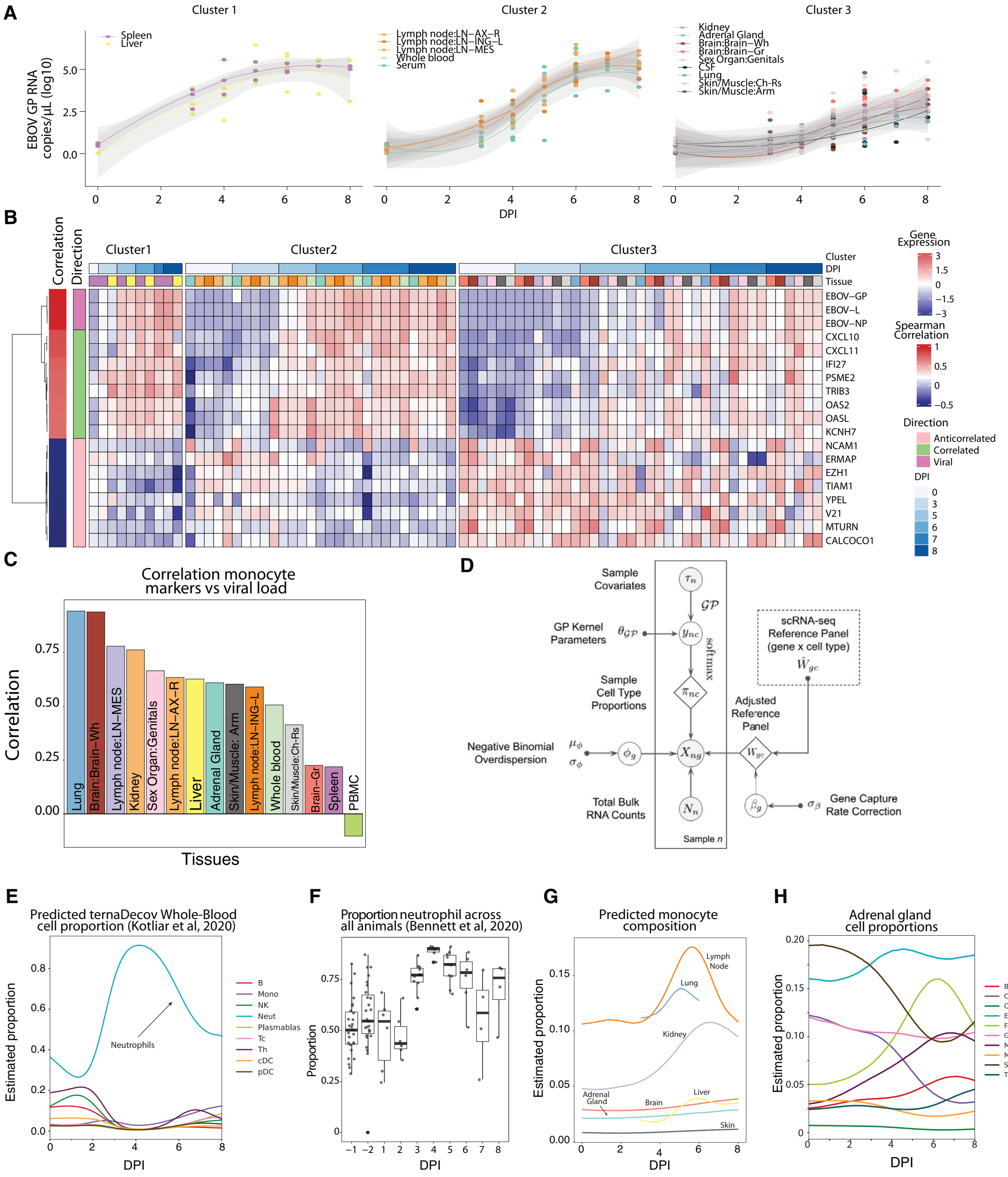

Figure 2. Correlating viral dynamics and host response to infection

(A) Viral loads, as determined by qRT-PCR, plotted versus time. The trajectories for different tissues were separated into three distinct patterns using K-means longitudinal data clustering, yielding groups of tissues with similar viral load dynamics.

(B) Gene expression across tissues (separated by the clusters in A) for the top 8 correlated and anti-correlated DEGs and 3 representative viral genes. Samples are ordered along the x axis by tissue and DPI. On the y axis, DEGs are clustered and labeled by direction.

(C) Correlation between viral load and canonical monocyte marker expression across each tissue.

(D) Overview of modular deconvolution framework used in ternaDecov. The output proportions from the models are then used to draw observed sample counts from a negative binomial distribution based on the provided single-cell profiles.

(legend continued on next page)

We confirmed the accuracy and biological relevance of ternaDecov's cellular proportion estimates and showed that trajectory models have advantages over individual point estimates made by existing methods. We benchmarked ternaDecov using a published bulk RNA-seq dataset from human pancreatic islets[36] and an scRNA-seq reference dataset.[37] We used expression of HbA1C as the covariate for trajectory regularization because levels of this gene are known to be related to changes in cell proportions.[32] Estimated cell proportions from ternaDecov showed a high correlation with results from an established deconvolution method, MuSiC,[32] including a negative correlation of β cell abundance with HbA1C levels (Figure S6). To further assess the biological relevance of ternaDecov's outputs, we used the whole-blood samples in our study. Deconvolution of bulk whole-blood RNA sequencing with ternaDecov identified an increase in the proportion of neutrophils that peaked at 4 DPI (Figure 2E). This peak mirrored the observed increase in neutrophils as measured by fluorescence flow cytometry[28] (Figure 2F), scRNA-seq (0.2%–65.1% of cells between baseline and late EVD),[18] and CyTOF (9.3%–49.8%).[18] Results were again consistent between ternaDecov and MuSiC (Figure S6), but ternaDecov showed faster runtimes. In addition, the trajectory models used by ternaDecov allow inference of unmeasured time points and reduce L1 error of estimates for measured time points (STAR Methods).

We next applied ternaDecov to estimate monocyte infiltration across tissues. For each tissue, we created a joint atlas of tissue-specific cell types and blood cell types (STAR Methods), and deconvolved their blood monocyte, blood non-monocyte, and tissue-specific cell type fractions. The proportion of monocytes/monocyte-derived macrophages varied across tissues, with the highest peak occurring in the lymph nodes following infection. Several tissues—most notably the lymph node, lung, kidney and liver—showed a sharp increase in the proportion of monocytes beginning around 4 DPI (Figure 2G). In contrast, the proportions of other blood cell types remained stable, and this change was not observed in tissues that are large reservoirs of monocytes at baseline (Figures 2E and S6), indicating a specific increase in monocytes in certain tissues and not an increase in circulating blood. This finding suggests that infiltrating monocytes influence the transcriptional signatures observed at this stage of infection. Deconvolution further illuminated changes in tissue-specific cell types during infection (Figure S6), such as the decrease of chromaffin cells in the adrenal gland (Figure 2H), a cell type that is infected during EVD.[38] Chromaffin cells produce epinephrine, an essential hormone for the host response to infection, whose depletion could be associated with severe disease.

A tissue atlas illuminates the spatiotemporal dynamics of interferon and cytokines during EVD

To further discover molecular signatures of infection, we identified genes whose expression changed upon infection in at least one tissue or fluid. We identified differentially expressed genes (DEGs) between infected and non-infected samples (DPI $\leq$ 0) independently for every tissue (false discovery rate [FDR] < 0.05 and log2 fold change (FC) > 2), resulting in the identification of between 35 and 974 DEGs per tissue (Figure 3A; Table S3). To avoid tissue sampling effects, we excluded tissue marker genes when interpreting genes across tissues (Figure S7; STAR Methods). Principal component analysis (PCA) using the log2 FCs of DEGs showed separation of tissues, indicating tissue-specific differences in response to infection (Figure 3B). Interestingly, the primary axis of variation (PC1; 12.3% variance explained) across tissues is driven by several genes related to the interferon response (Figure 3B).

We confirmed the key role of interferons and cytokines in the host response during EVD across tissues. Past studies have shown that expression of genes associated with the type I interferon response generally increases in blood and several tissues during EVD.[14,17,39–41] Similarly, we found that interferon and related genes were upregulated in EVD and demonstrate that this trend is recapitulated in our extensive set of 15 distinct tissues (Figures 3C and S4). We observe a similar increase in some cytokine genes, especially in the whole blood, spleen, and skin (Figure 3C). These responses are common to viral infections in general, and their increased expression across multiple tissues is present in the well-established clinical manifestation of "cytokine storm/cytokine release syndrome," which occurs during EVD.[42,43]

While these genes were upregulated across distinct tissues, the degree and temporal dynamics of this upregulation differed. Indeed, although many of these genes were globally upregulated across tissues, they were also represented as the top genes driving the separation of tissues, underscoring the distinct dynamic profiles (Figure 3B). To further explore differences in the interferon and cytokine response across tissues, we examined DEGs changing over time in each tissue. Among these genes globally upregulated in response to infection, ISGs and cytokines had different dynamics between tissues across time, with an early increase in spleen, lymph nodes, liver, and whole blood and a delayed increase in secondary organs such as the brain (Figures 3D and S8). This indicates a broadly conserved interferon and cytokine response across tissues, albeit with distinct dynamics likely associated with the circulation of the virus and recruited immune cells during pathogenesis.

Tissue-specific transcription profiles reveal novel genes and pathways dysregulated in EVD

We uncovered novel transcriptional signatures of disease, identifying differences in the host responses across tissues and inter-tissue heterogeneity (Figures 3D, 3E, and S8). Among the DEGs with the greatest fold change in each tissue, several genes were differentially expressed in only a subset of tissues. For example, we observed changes in apoptosis- and inflammation-related genes particularly in the whole blood and kidneys. We also

(E) Deconvolution of whole blood using scRNA-seq data[18] confirms the detected increase in neutrophils at 4 DPI.
(F) Proportion of neutrophils across samples using Sysmex XT-2000iV automated hematology by flow cytometry[28].
(G) Deconvolution of monocyte composition across time for each tissue based on an scRNA-seq reference of *Macaca fascicularis*.
(H) Deconvolution of predicted cell type proportion across time for adrenal glands.

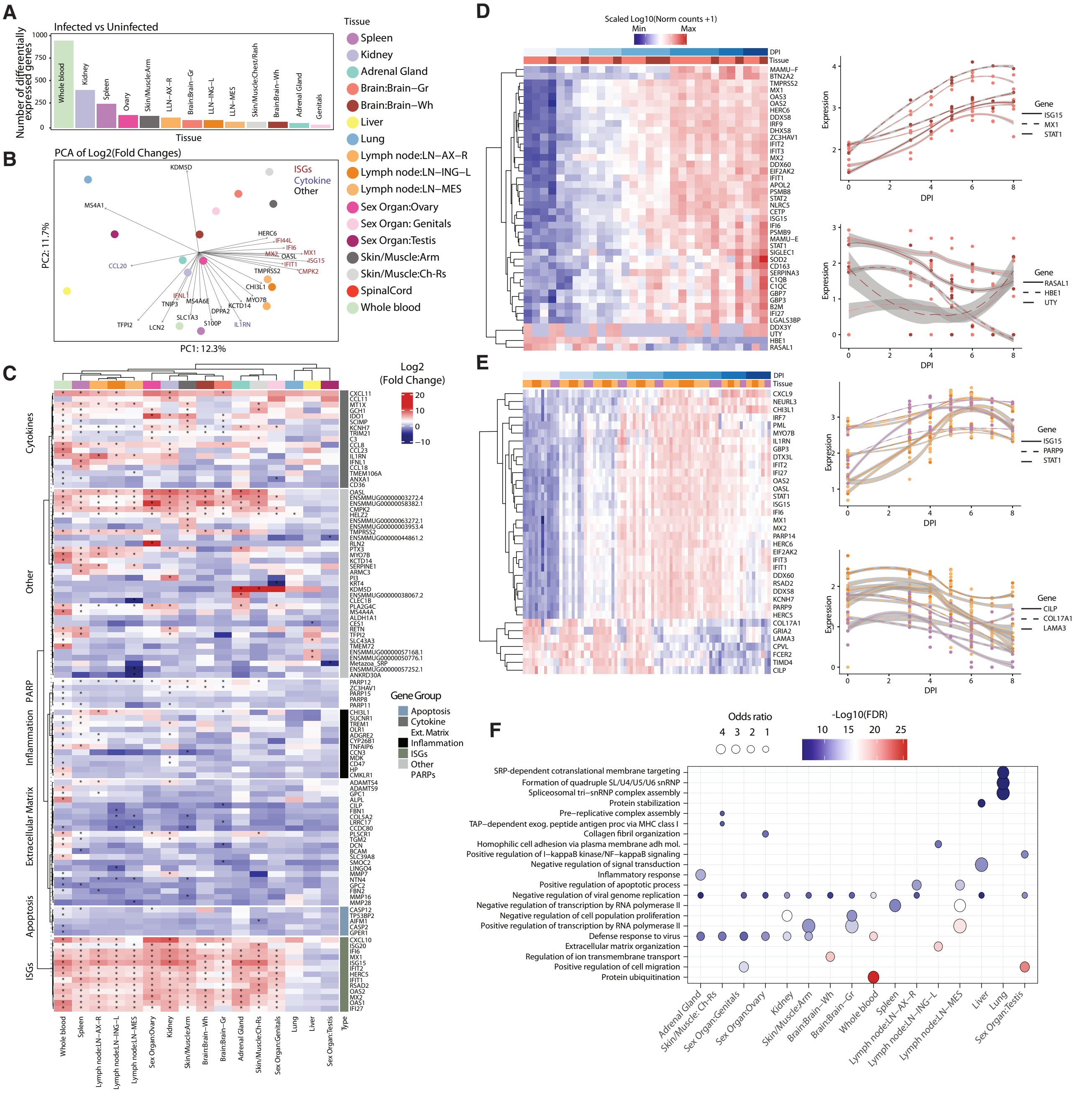

Figure 3. Host transcriptomics across tissues and time

(A) Number of DEGs between non-infected and infected samples; tissues with more than 5 DEGs are shown in the plot.

(B) PCA of log2 fold changes of significantly DEGs between infected and uninfected samples. Top contributing genes for PC1 and PC2 are highlighted.

(C) Heatmap of fold-changes of top DEGs across tissues, stratified by meaningful gene categories; stars marks significant differential expression (FDR < 0.05).

(D) Left: heatmap of genes changing significantly across time for brain. Right: gene expression changes across time for selected genes. Colors atop plots designate gray (light red) and white matter (dark red).

(E) Same as (D) but for lymph nodes (shades of orange) and spleen (purple); colors atop plots designate tissues.

(F) Gene Ontology (GO) term analysis of genes differentially expressed (top 100 FDR < 0.01) across time as determined by ImpulseDE2. Enriched terms were determined per tissue, and the top 3 GO terms, as determined by Kolmogorov-Smirnov (KS) test, per tissue were selected for display. Colors of circles correspond to −log10(KS pval) of the enriched term within tissue, and sizes of circles correspond to odds ratio.

noted increased expression of PARP-family genes (*PARP12*, *ZC3HAV1*, *PARP15*, *PARP6*, and *PARP11*) in kidney and skin (Figure 3C). Members of the PARP family are responsible for functions including DNA repair and chaperoning[44,45] and can have pro-viral effects. For instance, PARP11 acts as a pro-viral factor in vesicular stomatitis virus infection by inhibiting the strength of interferon (IFN)-I-activated signaling.[46] It is possible, therefore, that the PARP family may contribute to pathogenesis during EVD.

To nominate underlying pathogenic processes of EVD that might be indicated by DEGs, we used Gene Ontology enrichment analysis to interpret tissue-conserved and tissue-specific signals. We identified common pathways enriched across tissues during infection, including "negative regulation of viral genome replication" and "defense response to virus" (Figure 3F). These pathways likely represent an enrichment of general antiviral defense genes common to all tissues, including genes related to the conserved IFN and cytokine responses we identified previously. Additionally, we identified enriched tissue-specific pathways, including cell migration, matrix formation, and organization (Figure 3F). These pathways suggest differential remodeling of tissues as a driver or consequence of EVD progression.

We observed significant changes in expression of genes encoding tissue connectivity- and extracellular matrix (ECM)-related proteins. Specifically, we saw a significant decrease in expression over time for tissue connectivity-related genes such as laminin, cartilage, and collagen (*CILP*, *LAMA3*, and *COL17A1*) in lymph nodes and spleen (Figures 3E and S9). These genes have not been reported as molecular signatures of disease but are consistent with the histological changes in vascular structure and function observed during EVD.[42] We observed similar changes in ECM-related genes in other organs, specifically in skin/muscle samples, as well as an increase in the expression of genes encoding metallopeptidases proteins (*MMP2*, *MMP3*, and *MMP8*) in the skin, brain, and whole blood (Figure S9). These results suggest that onset of multiorgan failure, increase in vascular permeability, and internal bleeding associated with EVD may be related to weakening of tissue connectivity associated with a downregulation of ECM genes, in addition to the known increase of tissue factor (F3) in the blood[30] (Figure S9).

Viral variants reveal patterns of compartmentalization and circulation among tissues

Given the high viral loads in several tissues in this study and the promiscuous tropism of EBOV,[47] we sought to elucidate how the virus spreads *in vivo* using viral variants that emerge over infection. We attempted viral genome assembly on all sequenced samples and obtained complete (>95% unambiguous nucleotides) viral genomes from 95 samples for further comparisons. Among all complete genomes, there was a single consensus-level (>50% variant frequency) mutation. The variant, which fell at position 10,343 (in the viral protein 24 [VP24] 5′ UTR), was detected in the sex organ of an animal sacrificed 6 DPI. The lack of consensus-level variants was expected, given the short duration of infection and absence of specific selective pressure. We also profiled minor variants in 45 samples that had sufficient viral coverage (>400x mean depth) (Figure S10; Table S4). Across the sample set, minor variants ranged from 2%–22% frequency and fell at a total of 111 unique nucleotide positions. Of these 111 variants, 5 variants were present in the infecting stock at more than 2% frequency, and an additional 3 variants were present at a more conservative threshold of 0.5% frequency (Figure 1E). To focus our analysis only on variants that arose within animals, we filtered out these 8 variants, leaving variants at 103 nucleotide positions for further study.

We first assessed global patterns in the number and frequency of variants in different tissues. We analyzed all samples available but specifically focused on whole blood, spleen, and the three distinct lymph nodes because high-coverage viral genomes were available for many animals in each of these tissues. The lymph nodes had a large number of variants that emerged within animals with high frequency; 37% of variants in the inguinal lymph node and 43% of variants in the axial lymph node had more than 5% frequency (Figure 4A). The number of variants was also consistently high in the lymph node samples across animals but with variable DPI (Figure 4B). Conversely, spleen and whole blood consistently had the fewest variants detected across animals (Figure 4B). We observe that, compared with spleen and whole blood, lymph nodes harbor more variants, and these variants also tend to be observed at higher frequencies. We find an apparent skew in the ratio of nonsynonymous to synonymous mutations in high-frequency (>5%) vs. low-frequency (<5%) variants in the inguinal lymph nodes by permutation test (5 vs. 0.11 in inguinal, $p = 0.006$; 1 vs. 1.36 in mesenteric, $p = 0.58$; 1.3 vs. 1.7 in axial, $p = 0.43$), suggesting that selective pressure may contribute to differences in variant frequencies between tissues.

We probed further to investigate the cause of the higher viral population diversity observed in the lymph nodes compared with that of the whole blood and spleen. For the 6 animals (2 animals from each of the 6-, 7-, and 8-DPI cohorts), we assessed the overlap of all variants observed across tissues. Globally, we found that samples from each of the three lymph nodes had several variants that were unique to that tissue, while spleen and whole blood variants were almost always shared with at least one other tissue (Figure 4C). In fact, many of the variants identified in the whole blood and spleen samples were identified in every other tissue profiled (Figure 4D). Generally, we observed a high degree of similarity between variant profiles in the whole blood and spleen and more similarity between these two tissues and each lymph node than among the lymph nodes (Figure 4D).

To investigate the source of viral diversity in the lymph nodes, we considered all tissues, noting that the sex organ samples have variant profiles that are most distinct from other tissues. For example, in the animal with a consensus-level (>50% frequency) variant, we found that there were multiple high-frequency variants in the sex organ and ovary samples, which were at an elevated frequency in the mesenteric lymph node sample, but were not detected or at low frequency (<5%) in any other sample from that individual. Previous studies have suggested that infection can be compartmentalized to the sex organs and ovaries.[48,49] Our data more directly confirm the occurrence of compartmentalized infections in these tissues. The variants rising to high frequency in these sites were likely spread to the more proximal mesenteric lymph node (Figure 4E). This hypothesis may be generalized to explain why lymph nodes harbor

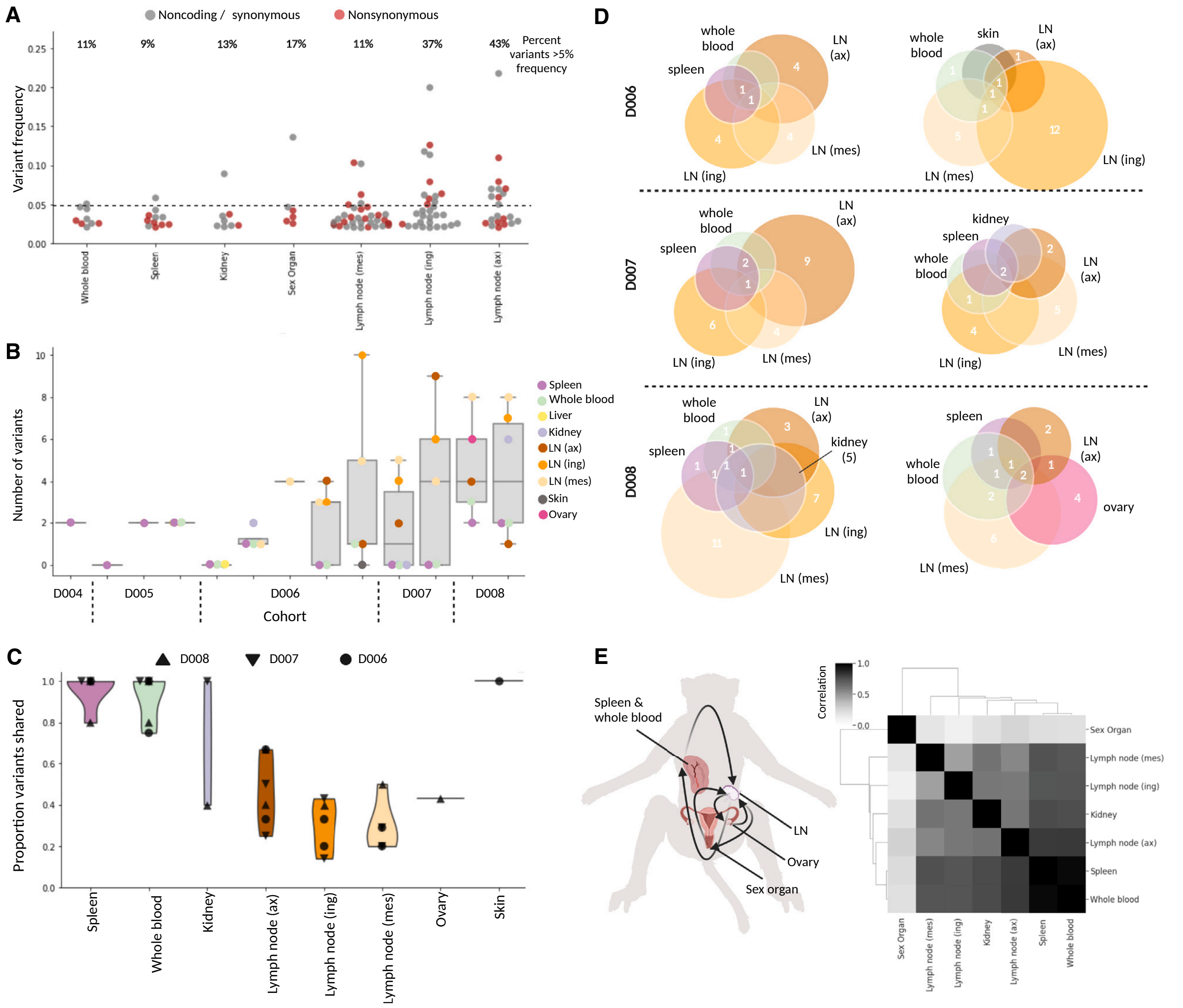

Figure 4. Minor viral variants show compartmentalization and circulation

(A) Frequencies of all nonsynonymous (red) and synonymous/noncoding (gray) variants that emerged during infection, plotted and separated by tissue; the percentage of variants above 5% frequency (dotted line) is given above each tissue.

(B) For each animal (ordered by DPI), the number of variants that emerged in every tissue (samples with >400× mean viral coverage).

(C) Violin plot showing the proportion of shared viral variants, separated by tissue; each point represents a unique animal, and symbols demonstrate DPI.

(D) Schematic representing variants that are shared (numbers displayed in overlapping circles) and not shared (numbers displayed in non-overlapping circles) in all tissues available for 6 animals (2 of each the D6, D7, and D8 cohorts).

(E) Left: schematic of viral circulation among tissues, based on the variant profiles (image created with BioRender). Right: a Spearman correlation of different tissues' variant profiles, concatenated across animals.

many high-frequency, unshared variants; they likely traffic between a subset of peripheral tissues with high-frequency variants that have emerged in compartmentalized infections.

Viral variants and functional analysis suggest adaptation during EBOV infection

The viral variants that emerged over the course of infection can also help us understand viral evolution and dynamics. Emergent variants may positively or negatively impact virus biology, including altering tropism, infectivity, and escape potential.[20,50]

We examined the distribution and types of emerging mutations across the viral genome. UTRs showed a higher number of variants per 1,000 bp than coding regions (8.1 versus 5.9), consistent with findings of intra-host diversity in human cases.[23] Among genes, we observed the highest number of mutations per 1,000 bp in VP40 (14.3), which is involved in virion assembly and immune evasion,[51] and glycoprotein (GP) (6.9), which is immunogenic and critical for infectivity[52] (Figure 5A). VP40 and GP also had the highest proportions of nonsynonymous variants. We observed narrower regions of other genes that, with high

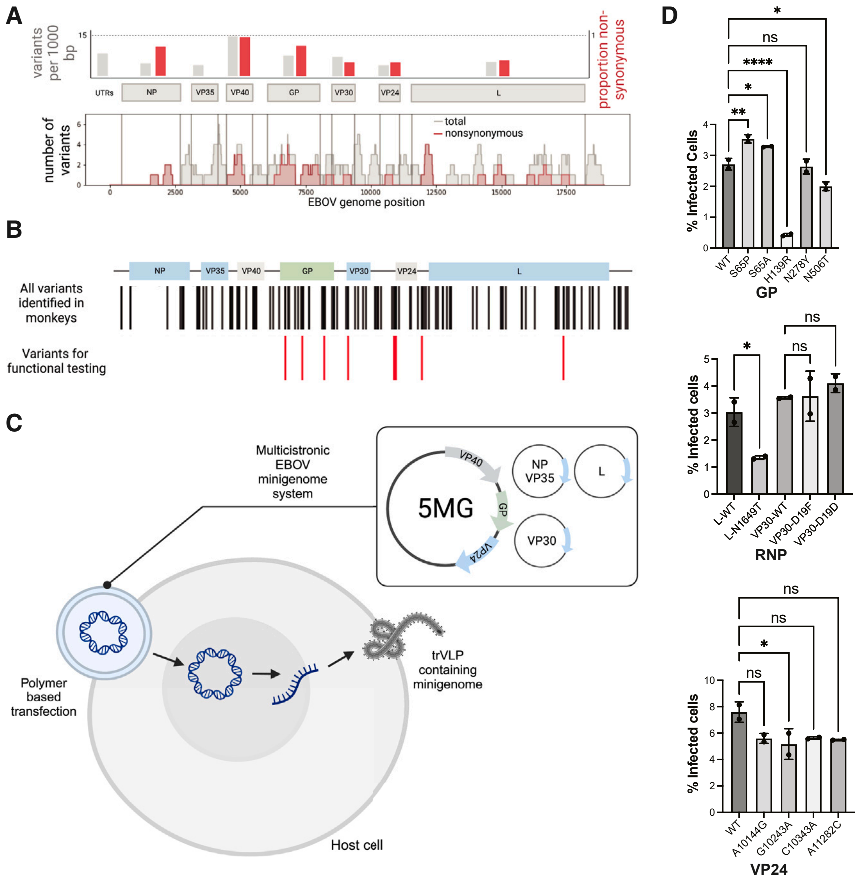

Figure 5. Viral adaptation and fitness effects

(A) Top: number of emergent variants per 1,000 bp (gray) were quantified for each gene-coding region as well as proportion of nonsynonymous variants (red). Bottom: accumulation of total (gray) and nonsynonymous (red) variants in specific gene regions was quantified using a sliding window of 200 bp.

(B) Genomic locations of variants selected for further functional testing (red) among all variants identified across the EBOV genome (black).

(C) Schematic of the EBOV/Kikwit transcription- and replication-competent virus-like particle (trVLP) minigenome system that recapitulates the wild-type and variant viral life cycle in a host cell (image created with BioRender).

(D) Flow cytometry analysis of the percentage of GFP+ cells 48 h post minigenome transfection as a percentage of infected host cells by seed stock (wild type [WT]) or viral variants in GP, RNP, and VP24. Error bars represent standard deviation.

proportions of nonsynonymous variants, including the C-terminal end of the nucleoprotein (NP) and N-terminal end of the viral polymerase (L), which are each part of the ribonucleoprotein (RNP) complex that performs viral replication and transcription (Figure 5A). We find evidence of negative selection in the L gene by binomial test ($p = 2.6 \times 10^{-5}$) but no evidence of ratio skew in VP40, GP, or NP (respective p values of 0.24, 0.53, and 0.13). Across the genome, A-to-G and T-to-C mutations were more frequent than G-to-A or C-to-T mutations, with a particularly high proportion of these mutations in two specific animals (Figure S11). We did not observe clear tissue-specific trends in variant location or type (Figure S12).

We adapted a well-established transcription- and replication-competent virus-like particle (trVLP) minigenome system[53] to assess the functional effects of eight coding mutations (in GP, L, and VP30) and four non coding mutation (in the UTR of VP24) across the complete viral life cycle (Figure 5B). This system allows the study of EBOV genes outside of BSL-4 laboratories by separating the RNP complex into three separate plasmids (L, NP-P2A-VP35, and VP30) that drive replication of a T7-driven minigenome composed of reporter genes and the remaining three EBOV genes (VP40, GP, and VP24) (Figure 5C). We recloned the entire system to encode the EBOV/Kikwit backbone, as established previously trVLP systems encoded EBOV genes from variants that diverge in sequence from Kikwit by hundreds of nucleotides. Co-transfection of all four plasmids into mammalian host cells results in transcription and replication of the multicistronic minigenome, including a fluorescent marker, which we detected by flow cytometry. These cells also produce GP-coated trVLPs, which can infect any target cell that expresses the viral RNP complex. For testing, we prioritized variants that emerged in multiple animals or rose to high frequency or changed in frequency relative to the infecting stock and were in genes or regions likely to be important for viral fitness (Figure 5B).

Because mutations in viral glycoproteins are often under selection, we prioritized these variants for functional effects. Of the five GP variants we tested, four had a significant effect on viral fitness (Figures 5D and S13). Consistent with the role GP plays during viral entry, additional testing with a GP-pseudotyping assay that specifically models this step suggests that this fitness difference is likely due to a difference in productive host-receptor interactions (Figure S13). The convergent mutations at amino acid position 65 (S65A and S65P) resulted in an increase in infectivity. Notably, a mutation at this position was present in viral sequences from a human case (GenBank: MH121168.1) and has been shown previously to be important for establishing mouse-adapted variants of EBOV/Mayinga and EBOV/Makona,[54–56] further supporting a key role played by this position. On the other hand, the variants H139R and N506T resulted in a significant loss of infectivity. Interestingly, a published crystal structure of GP bound to the human receptor NPC1 showed that H139R is proximal to this interaction,[57] and the region surrounding N506T is the binding site of the neutralizing antibody KZ52, derived from a human survivor of the 1995 Kikwit outbreak.[58]

Next, we leveraged our ability to simulate the full viral life cycle with the trVLP minigenome system to study mutations in genes that impact transcription and replication. Functionally relevant

mutations have emerged during human outbreaks of EBOV in genes involved in viral replication and transcription as well as in regulatory regions.[22,23,59,60] Of the four VP24 UTR variants we tested, only G10243A showed a slight impact on viral fitness, potentially because of the more subtle ways in which UTR variants could affect viral fitness, which are outside the limit of detection for this system. Among the three variants we tested in the RNP complex, we found that mutations in VP30 showed no significant effect on viral fitness; however, a mutation (N1649T) on the viral polymerase (L) has a significant effect on viral fitness (Figures 5D and S13). N1649T is located in the predicted MTase domain of the viral RNA dependent RNA polymerase (RdRp)[61] and decreased viral fitness. Despite recent elucidation of the complete RdRp structure,[61] the MTase domain has yet to be experimentally resolved. Our results suggest that it might play a role in maintaining viral fitness, warranting further studies of its structure and function.

DISCUSSION

Here, we apply high-depth, unbiased sequencing, complemented by newly established experimental and computational approaches, to a large natural history study in rhesus monkeys to provide insights into the molecular basis of disease. We describe detectable levels of EBOV RNA in most tissues, with the earliest infection in the liver and spleen and particularly high viral loads in the blood, lymph nodes, and adrenals, consistent with previous reports of tropism and pathology.[47,49,62–66] By following these dynamics over time, we can further observe how infection drives disease progression and virus adaptation. Together, these perspectives show widespread, systemic changes during acute disease.

Emerging variants at over 100 positions across the viral genome illuminated potential sites of adaptation and compartmentalization during acute infection. Shared patterns of minor variants suggest a model where the spleen and blood spread virus systemically, likely mediated by recruitment of infected monocytes, while the lymph nodes traffic virus among locally compartmentalized infections. Compartmentalized infections in EVD, particularly in immune-privileged sites like the reproductive tract, could promote persistent infection and sustained evolution and pose a risk for reactivation and onward transmission.[67] Using genomic data, we show that, after viral dissemination in EBOV-exposed NHPs,[48,68,69] viral populations are actively maintained and compartmentalized in these tissues, distinct from infection in other organs. Several features of this emerging viral variation, including a higher frequency of T-to-C mutations, have been observed in human outbreaks[23,70,71] and in response to therapeutic agents.[20] The higher frequency of T-to-C and A-to-G mutations relative to G-to-A mutations may suggest host RNA editing activity, and past studies indicate that T-to-C mutations are clustered in specific regions.[70] In contrast, VP40, which here had the highest frequency of nonsynonymous mutations (Figure 5A), has been suggested previously to be strongly conserved in human outbreaks.[23] The differences in the distribution of mutations across some viral genes may reflect rapid initial adaptation of the virus, similar to that seen immediately after zoonotic spillover. The number of unique viral variants we detect in tissues highlights the importance of animal models for providing insights into selective pressures in different compartments.

Of the 12 variants we tested in our minigenome system, six were found to significantly alter viral fitness, with the majority of these (4 of 6) falling in the GP gene, indicating viral entry as a mechanism. Half of the variants we tested did not have any observed impact on viral fitness. This is unsurprising because variants could have increased in frequency by chance because of genetic drift, further highlighting the importance of experimental assays that can rapidly and easily screen for functional effects of mutations. The filovirus GP, RdRp, and RNP complexes have long been considered promising targets for broad antiviral therapy.[72–76] Although further mechanistic and structural studies are needed to determine the impact the emerging mutations detected in this study have on viral fitness, our results support the potential of trVLPs to uncover novel mutations that affect viral entry, replication, and infection, which could guide future rational design approaches in drug discovery.

Our analysis of host transcriptional responses across tissues adds further dynamic and tissue-specific context to known features of pathogenesis and identifies intriguing novel responses related to tissue connectivity. Beyond expected changes in ISG and cytokine expression,[14] the comprehensive nature of our dataset enabled us to identify differential dynamics across tissues. This study also revealed previously unknown features of disease. We observed changes in ECM genes in most tissues, with widespread dysregulation of collagen-, laminin-, and cartilage-related gene families in several tissues as well as an increase in collagen cleaving enzymes such as metallopeptidase (*MMP8*, *MMP3*, and *MMP2*) in the blood, skin, and brain. These findings provide new molecular insight into the etiology of vascular endothelial and connective tissue disruption (i.e., vascular leak syndromes, characteristic of severe EVD) and may suggest molecular pathobiology common to other hemorrhagic fevers; for example, similar dysregulations in ECM have been reported in other hemorrhagic fevers, such as dengue virus infection,[77] and ECM cleaving enzymes play a key role in venom-induced hemorrhage.[78] Interestingly, these enzymes have also been reported to play a role in cell-to-cell viral transmission in West Nile virus[79] and influenza virus,[80] warranting further investigation into the roles of these genes in EVD.

Characterizing host and pathogen dynamics in this large serial sacrifice study required establishing new computational and experimental tools that we believe will be of broad use in future studies. ternaDecov fills a key gap among available deconvolution tools[32–35] when time-series bulk RNA-seq data are available. By incorporating time as a variable in its deconvolution model of bulk data from a single-cell reference, ternaDecov better models gene expression dynamics. While studying changes over the course of infection was our primary motivation in developing ternaDecov, any continuous covariates can be used, demonstrating the broader applicability of this method. Similarly, existing trVLP minigenome systems were not adapted to the EBOV variant used in this and many other animal studies of EVD. TrVLP minigenomes are powerful systems because they allow the full viral life cycle to be modeled at lower levels of biosafety containment and have been used previously to functionally

characterize mutations in other EBOV variants.[22,53] Because the EBOV Kikwit variant is recognized as the standard challenge virus for testing clinical countermeasures in animal studies, we believe that the EBOV/Kikwit trVLP system we adapted will be a valuable community resource for future assessment of emerging mutations.

Through this study, we add further spatial and temporal granularity to known signatures of EVD while also suggesting new molecular drivers of pathogenesis. We illustrate relationships between host and viral signatures during EVD and propose potential mechanisms that may generate these signatures. Finally, we provide computational and experimental tools to not only facilitate further investigations of EBOV infections but also provide a model for future studies seeking to nominate and validate molecular bases of disease progression.

Limitations of the study

The major limitations of this study arise from the constraints inherent to working in maximum containment, and there are several areas where the study could be expanded to increase the breadth and depth of characterization. In particular, many liver samples had low RNA quality, restricting the insights we could obtain for this tissue. The liver harbors many enzymes that degrade RNA, and degradation was likely exacerbated by the constraints of working in maximum containment. Improved preservation methods as well as even broader sampling of clinically relevant tissues, such as the gastrointestinal tract,[81,82] would be of interest for future investigations. Additionally, the timing of host transcriptional changes suggests that the recruitment of infected circulating monocytes is a major contributing factor to the spread of the virus to secondary organs. Future studies using scRNA-seq on tissue samples would allow changes in cell type proportions and the impact of infection on specific cell types to be measured more directly, as shown previously in peripheral blood mononuclear cells from this study.[18] Finally, uniformly lethal animal models like the one used here restrict the study of persistence, acute recovery, and long-term effects of the infection. New experimental challenge models with different routes of inoculation and heterogeneity in outcomes could enable a better understanding of these features in surviving NHPs.

STAR★METHODS

Detailed methods are provided in the online version of this paper and include the following:

- KEY RESOURCES TABLE
- RESOURCE AVAILABILITY
  - Lead contact
  - Materials availability
  - Data and code availability
- EXPERIMENTAL MODEL AND SUBJECT DETAILS
- METHOD DETAILS
  - Natural history study
  - Sample extraction and RNA purification
  - Quantification of viral RNA
  - Library construction and sequencing
  - Pentacistronic minigenome assay
  - GP-pseudotyped lentivirus and infectivity assays
  - Sequencing data preprocessing and quality control
  - Viral genomic analyses
  - Viral mutation statistics
  - Differential expression analysis
  - GO term enrichment analysis and correlation analysis
  - Genes expression changes across time
  - Time-regularized deconvolution of bulk RNA sequencing (ternaDecov)
  - ternaDecov: Trajectory models
  - ternaDecov: Implementation
  - ternaDecov: Technical benchmarking
  - ternaDecov: Biological benchmarking and application to EBOV RNAseq data

SUPPLEMENTAL INFORMATION

Supplemental information can be found online at https://doi.org/10.1016/j.xgen.2023.100440.

ACKNOWLEDGMENTS

This work is supported by US Food and Drug Administration (FDA) contracts HHSF223201810172C and HHSF223201610018C, National Institute of Allergy and Infectious Diseases (NIAID) U19AI110818, and HHMI (to P.C.S.). This work was partially supported by NIAID Interagency agreement NOR15003-001-0000. The non-human primate work completed at the NIAID Integrated Research Facility was supported in part by the NIAID Division of Intramural Research and NIAID Division of Clinical Research and was performed under Battelle Memorial Institute contract HHSN272200700016I, and manuscript drafting was performed under Laulima Government Solutions, LLC contract HHSN272201800013C. S.T.. was supported in part by a Pew Latin American Fellowship Program in Biomedical Sciences. J. Logue performed this work as an employee of Battelle. R.D.A., and R.S.B. are current employees of Laulima Government Solutions. D.K. was supported by award T32GM007753 from the National Institute of General Medical Sciences (NIGMS). A.E.L. was supported by the National Science Foundation (NSF) under grant DGE 1144152 and Damon Runyon Fellowship Award DRG-2432-21. M.M. was a Gilead Fellow of the Life Sciences Research Foundation. A.K.S. was supported by the Searle Scholars Program, the Beckman Young Investigator Program, a Sloan Fellowship in Chemistry, NIH 5U24AI118672, and the Bill and Melinda Gates Foundation. J. Luban was supported by NIH R01AI148784. D.S. was supported by fellowships from the Swiss National Science Foundation (P400PB_199261 and P2ELP3_187926). This project has been funded in whole or in part with federal funds from the National Cancer Institute, National Institutes of Health, under contracts HHSN261201500003I and 75N91019D00024 (to I.C.). The content of this publication does not necessarily reflect the views or policies of the Department of Health and Human Services, nor does mention of trade names, commercial products, or organizations imply endorsement by the US Government. The authors are solely responsible for the content of this paper, which does not necessarily represent the official views of the US Department of Health and Human Services (HHS), the NIH, the NIGMS, the FDA, or the institutions and companies affiliated with the authors. We thank Brittany Petros, Gage Moreno, and other members of the Sabeti lab for helpful discussions and Parvathy Nair for illustration of the graphical abstract.

AUTHOR CONTRIBUTIONS

Conceptualization, E.N., S.T., S.S.R., K.L., D.K., R.S.B., A.K.S., A.E.L., N.B., L.E.H., P.C.S., and K.J.S.; methodology, E.N., S.T., S.S.R., T.C.T.L., K.L., D.L., D.S., D.K., R.S., A.N.H., R.S.B., A.K.S., N.B., A.E.L., L.E.H., P.C.S., and K.J.S.; software, M.S. and N.B.; formal analysis, E.N., S.T., S.S.R., T.C.T.L.,

K.L., A.R., K.S.M., M.S., and N.B.; investigation, E.N., T.C.T.L., K.L., M.R., G.C.A., K.C.D., J. Logue, D.L., D.S., R.D.A., K.J., R.S.B., and A.E.L.; resources, F.Z., M.B., J. Luban, A.K.S., L.E.H., and P.C.S.; data curation, E.N., S.H.T.S., S.S.R., and N.B.; writing – original draft; E.N., S.T., S.S.R., T.C.T.L., K.L., N.B., A.E.L., and K.J.S.; writing – review & editing; all authors; supervision, I.C., F.Z., M.B., J. Luban, R.S.B., A.K.S., N.B., A.E.L., L.E.H., P.C.S., and K.J.S.; funding acquisition, D.K., J.L.R., M.M., A.K.S., A.E.L., L.E.H., and P.C.S.

DECLARATION OF INTERESTS

P.C.S. is a co-founder and shareholder of Sherlock Biosciences and Delve Bio, a board member and shareholder of Danaher Corporation, and has filed IP related to genomic sequencing and diagnostic technologies. A.K.S. reports compensation for consulting and/or scientific advisory board (SAB) membership from Merck, Honeycomb Biotechnologies, Cellarity, Repertoire Immune Medicines, Ochre Bio, Third Rock Ventures, Hovione, Relation Therapeutics, FL82, FL86, Empress Therapeutics, IntrECate Biotherapeutics, Senda Biosciences, and Dahlia Biosciences unrelated to this work. F.Z. is a scientific advisor and cofounder of Editas Medicine, Beam Therapeutics, Pairwise Plants, Arbor Biotechnologies, and Aera Therapeutics. F.Z. is a scientific advisor for Octant.

INCLUSION AND DIVERSITY

One or more of the authors of this paper self-identifies as an underrepresented ethnic minority in their field of research or within their geographical location. One or more of the authors of this paper self-identifies as a gender minority in their field of research. One or more of the authors of this paper self-identifies as a member of the LGBTQIA+ community. One or more of the authors of this paper self-identifies as living with a disability. One or more of the authors of this paper received support from a program designed to increase minority representation in their field of research.

Received: August 8, 2022
Revised: February 27, 2023
Accepted: October 15, 2023
Published: November 21, 2023

REFERENCES

1. Jacob, S.T., Crozier, I., Fischer, W.A., 2nd, Hewlett, A., Kraft, C.S., Vega, M.A.d.L., Soka, M.J., Wahl, V., Griffiths, A., Bollinger, L., and Kuhn, J.H. (2020). Ebola virus disease. Nat. Rev. Dis. Prim. *6*, 13.
2. Hartman, A.L., Towner, J.S., and Nichol, S.T. (2010). Ebola and marburg hemorrhagic fever. Clin. Lab. Med. *30*, 161–177.
3. Misasi, J., and Sullivan, N.J. (2014). Camouflage and misdirection: the full-on assault of ebola virus disease. Cell *159*, 477–486.
4. Center for Biologics Evaluation, and Research (2020). ERVEBO. U.S. Food and Drug Administration. https://www.fda.gov/vaccines-blood-biologics/ervebo.
5. Office of the Commissioner (2020). FDA Approves First Treatment for Ebola Virus (U.S. Food and Drug Administration). https://www.fda.gov/news-events/press-announcements/fda-approves-first-treatment-ebola-virus.
6. Mulangu, S., Dodd, L.E., Davey, R.T., Jr., Tshiani Mbaya, O., Proschan, M., Mukadi, D., Lusakibanza Manzo, M., Nzolo, D., Tshomba Oloma, A., Ibanda, A., et al. (2019). A Randomized, Controlled Trial of Ebola Virus Disease Therapeutics. N. Engl. J. Med. *381*, 2293–2303.
7. Basler, C.F. (2017). Molecular pathogenesis of viral hemorrhagic fever. Semin. Immunopathol. *39*, 551–561.
8. Woolsey, C., Borisevich, V., Prasad, A.N., Agans, K.N., Deer, D.J., Dobias, N.S., Heymann, J.C., Foster, S.L., Levine, C.B., Medina, L., et al. (2021). Establishment of an African green monkey model for COVID-19 and protection against re-infection. Nat. Immunol. *22*, 86–98.
9. Delorey, T.M., Ziegler, C.G.K., Heimberg, G., Normand, R., Yang, Y., Segerstolpe, Å., Abbondanza, D., Fleming, S.J., Subramanian, A., Montoro, D.T., et al. (2021). COVID-19 tissue atlases reveal SARS-CoV-2 pathology and cellular targets. Nature *595*, 107–113.
10. Stephenson, E., Reynolds, G., Botting, R.A., Calero-Nieto, F.J., Morgan, M.D., Tuong, Z.K., Bach, K., Sungnak, W., Worlock, K.B., Yoshida, M., et al. (2021). Single-cell multi-omics analysis of the immune response in COVID-19. Nat. Med. *27*, 904–916.
11. Normandin, E., Rudy, M., Barkas, N., Schaffner, S.F., Levine, Z., Padera, R.F., Jr., Babadi, M., Mukerji, S.S., Park, D.J., MacInnis, B.L., et al. (2023). High-depth sequencing characterization of viral dynamics across tissues in fatal COVID-19 reveals compartmentalized infection. Nat. Commun. *14*, 574.
12. Quach, H., Rotival, M., Pothlichet, J., Loh, Y.-H.E., Dannemann, M., Zidane, N., Laval, G., Patin, E., Harmant, C., Lopez, M., et al. (2016). Genetic Adaptation and Neandertal Admixture Shaped the Immune System of Human Populations. Cell *167*, 643–656.e17.
13. Garamszegi, S., Yen, J.Y., Honko, A.N., Geisbert, J.B., Rubins, K.H., Geisbert, T.W., Xia, Y., Hensley, L.E., and Connor, J.H. (2014). Transcriptional correlates of disease outcome in anticoagulant-treated non-human primates infected with ebolavirus. PLoS Neglected Trop. Dis. *8*, e3061.
14. Caballero, I.S., Honko, A.N., Gire, S.K., Winnicki, S.M., Melé, M., Gerhardinger, C., Lin, A.E., Rinn, J.L., Sabeti, P.C., Hensley, L.E., and Connor, J.H. (2016). In vivo Ebola virus infection leads to a strong innate response in circulating immune cells. BMC Genom. *17*, 707.
15. Liu, X., Speranza, E., Muñoz-Fontela, C., Haldenby, S., Rickett, N.Y., Garcia-Dorival, I., Fang, Y., Hall, Y., Zekeng, E.-G., Lüdtke, A., et al. (2017). Transcriptomic signatures differentiate survival from fatal outcomes in humans infected with Ebola virus. Genome Biol. *18*, 4.
16. Speranza, E., Bixler, S.L., Altamura, L.A., Arnold, C.E., Pratt, W.D., Taylor-Howell, C., Burrows, C., Aguilar, W., Rossi, F., Shamblin, J.D., et al. (2018). A conserved transcriptional response to intranasal Ebola virus exposure in nonhuman primates prior to onset of fever. Sci. Transl. Med. *10*, eaaq1016.
17. Jankeel, A., Menicucci, A.R., Woolsey, C., Fenton, K.A., Mendoza, N., Versteeg, K., Cross, R.W., Geisbert, T.W., and Messaoudi, I. (2020). Early Transcriptional Changes within Liver, Adrenal Gland, and Lymphoid Tissues Significantly Contribute to Ebola Virus Pathogenesis in Cynomolgus Macaques. J. Virol. *94*, e00250-20.
18. Kotliar, D., Lin, A.E., Logue, J., Hughes, T.K., Khoury, N.M., Raju, S.S., Wadsworth, M.H., 2nd, Chen, H., Kurtz, J.R., Dighero-Kemp, B., et al. (2020). Single-Cell Profiling of Ebola Virus Disease In Vivo Reveals Viral and Host Dynamics. Cell *183*, 1383–1401.e19.
19. Whitfield, Z.J., Prasad, A.N., Ronk, A.J., Kuzmin, I.V., Ilinykh, P.A., Andino, R., and Bukreyev, A. (2020). Species-Specific Evolution of Ebola Virus during Replication in Human and Bat Cells. Cell Rep. *32*, 108028.
20. Kugelman, J.R., Kugelman-Tonos, J., Ladner, J.T., Pettit, J., Keeton, C.M., Nagle, E.R., Garcia, K.Y., Froude, J.W., Kuehne, A.I., Kuhn, J.H., et al. (2015). Emergence of Ebola Virus Escape Variants in Infected Nonhuman Primates Treated with the MB-003 Antibody Cocktail. Cell Rep. *12*, 2111–2120.
21. Diehl, W.E., Lin, A.E., Grubaugh, N.D., Carvalho, L.M., Kim, K., Kyawe, P.P., McCauley, S.M., Donnard, E., Kucukural, A., McDonel, P., et al. (2016). Ebola Virus Glycoprotein with Increased Infectivity Dominated the 2013-2016 Epidemic. Cell *167*, 1088–1098.e6.
22. Lin, A.E., Diehl, W.E., Cai, Y., Finch, C.L., Akusobi, C., Kirchdoerfer, R.N., Bollinger, L., Schaffner, S.F., Brown, E.A., Saphire, E.O., et al. (2020). Reporter Assays for Ebola Virus Nucleoprotein Oligomerization, Virion-Like Particle Budding, and Minigenome Activity Reveal the Importance of Nucleoprotein Amino Acid Position 111. Viruses *12*.
23. Ni, M., Chen, C., Qian, J., Xiao, H.-X., Shi, W.-F., Luo, Y., Wang, H.-Y., Li, Z., Wu, J., Xu, P.-S., et al. (2016). Intra-host dynamics of Ebola virus during 2014. Nat. Microbiol. *1*, 16151.

24. Jacobs, M., Rodger, A., Bell, D.J., Bhagani, S., Cropley, I., Filipe, A., Gifford, R.J., Hopkins, S., Hughes, J., Jabeen, F., et al. (2016). Late Ebola virus relapse causing meningoencephalitis: a case report. Lancet *388*, 498–503.

25. Varkey, J.B., Shantha, J.G., Crozier, I., Kraft, C.S., Lyon, G.M., Mehta, A.K., Kumar, G., Smith, J.R., Kainulainen, M.H., Whitmer, S., et al. (2015). Persistence of Ebola Virus in Ocular Fluid during Convalescence. N. Engl. J. Med. *372*, 2423–2427.

26. Barnes, K.G., Kindrachuk, J., Lin, A.E., Wohl, S., Qu, J., Tostenson, S.D., Dorman, W.R., Busby, M., Siddle, K.J., Luo, C.Y., et al. (2017). Evidence of Ebola Virus Replication and High Concentration in Semen of a Patient During Recovery. Clin. Infect. Dis. *65*, 1400–1403.

27. Whitmer, S.L.M., Ladner, J.T., Wiley, M.R., Patel, K., Dudas, G., Rambaut, A., Sahr, F., Prieto, K., Shepard, S.S., Carmody, E., et al. (2018). Active Ebola Virus Replication and Heterogeneous Evolutionary Rates in EVD Survivors. Cell Rep. *22*, 1159–1168.

28. Bennett, R.S., Logue, J., Liu, D.X., Reeder, R.J., Janosko, K.B., Perry, D.L., Cooper, T.K., Byrum, R., Ragland, D., St Claire, M., et al. (2020). Kikwit Ebola Virus Disease Progression in the Rhesus Monkey Animal Model. Viruses *12*, 753.

29. Speranza, E., and Connor, J.H. (2017). Host Transcriptional Response to Ebola Virus Infection. Vaccines (Basel) *5*, 30.

30. Geisbert, T.W., Hensley, L.E., Larsen, T., Young, H.A., Reed, D.S., Geisbert, J.B., Scott, D.P., Kagan, E., Jahrling, P.B., and Davis, K.J. (2003). Pathogenesis of Ebola hemorrhagic fever in cynomolgus macaques: evidence that dendritic cells are early and sustained targets of infection. Am. J. Pathol. *163*, 2347–2370.

31. Van Acker, H.H., Capsomidis, A., Smits, E.L., and Van Tendeloo, V.F. (2017). CD56 in the Immune System: More Than a Marker for Cytotoxicity? Front. Immunol. *8*, 892.

32. Wang, X., Park, J., Susztak, K., Zhang, N.R., and Li, M. (2019). Bulk tissue cell type deconvolution with multi-subject single-cell expression reference. Nat. Commun. *10*, 380.

33. Avila Cobos, F., Alquicira-Hernandez, J., Powell, J.E., Mestdagh, P., and De Preter, K. (2020). Benchmarking of cell type deconvolution pipelines for transcriptomics data. Nat. Commun. *11*, 5650.

34. Chu, T., Wang, Z., Pe'er, D., and Danko, C.G. (2022). Cell type and gene expression deconvolution with BayesPrism enables Bayesian integrative analysis across bulk and single-cell RNA sequencing in oncology. Nat. Can. (Ott.) *3*, 505–517.

35. Dong, M., Thennavan, A., Urrutia, E., Li, Y., Perou, C.M., Zou, F., and Jiang, Y. (2021). SCDC: bulk gene expression deconvolution by multiple single-cell RNA sequencing references. Briefings Bioinf. *22*, 416–427.

36. Fadista, J., Vikman, P., Laakso, E.O., Mollet, I.G., Esguerra, J.L., Taneera, J., Storm, P., Osmark, P., Ladenvall, C., Prasad, R.B., et al. (2014). Global genomic and transcriptomic analysis of human pancreatic islets reveals novel genes influencing glucose metabolism. Proc. Natl. Acad. Sci. USA *111*, 13924–13929.

37. Segerstolpe, Å., Palasantza, A., Eliasson, P., Andersson, E.-M., Andréasson, A.C., Sun, X., Picelli, S., Sabirsh, A., Clausen, M., Bjursell, M.K., et al. (2016). Single-Cell Transcriptome Profiling of Human Pancreatic Islets in Health and Type 2 Diabetes. Cell Metabol. *24*, 593–607.

38. Cooper, T.K., Huzella, L., Johnson, J.C., Rojas, O., Yellayi, S., Sun, M.G., Bavari, S., Bonilla, A., Hart, R., Jahrling, P.B., et al. (2018). Histology, immunohistochemistry, and in situ hybridization reveal overlooked Ebola virus target tissues in the Ebola virus disease guinea pig model. Sci. Rep. *8*, 1250.

39. Pinski, A.N., Maroney, K.J., Marzi, A., and Messaoudi, I. (2021). Distinct transcriptional responses to fatal Ebola virus infection in cynomolgus and rhesus macaques suggest species-specific immune responses. Emerg. Microb. Infect. *10*, 1320–1330.

40. Kuroda, M., Halfmann, P.J., Hill-Batorski, L., Ozawa, M., Lopes, T.J.S., Neumann, G., Schoggins, J.W., Rice, C.M., and Kawaoka, Y. (2020). Identification of interferon-stimulated genes that attenuate Ebola virus infection. Nat. Commun. *11*, 2953.

41. Greenberg, A., Huber, B.R., Liu, D.X., Logue, J.P., Hischak, A.M.W., Hart, R.J., Abbott, M., Isic, N., Hisada, Y.M., Mackman, N., et al. (2020). Quantification of Viral and Host Biomarkers in the Liver of Rhesus Macaques: A Longitudinal Study of Zaire Ebolavirus Strain Kikwit (EBOV/Kik). Am. J. Pathol. *190*, 1449–1460.

42. Basler, C.F. (2017). West African Ebola Virus Strains: Unstable and Ready to Invade? Cell Host Microbe *21*, 316–318.

43. Younan, P., Iampietro, M., and Bukreyev, A. (2018). Disabling of lymphocyte immune response by Ebola virus. PLoS Pathog. *14*, e1006932.

44. Malgras, M., Garcia, M., Jousselin, C., Bodet, C., and Lévêque, N. (2021). The Antiviral Activities of Poly-ADP-Ribose Polymerases. Viruses *13*.

45. Jubin, T., Kadam, A., Jariwala, M., Bhatt, S., Sutariya, S., Gani, A.R., Gautam, S., and Begum, R. (2016). The PARP family: insights into functional aspects of poly (ADP-ribose) polymerase-1 in cell growth and survival. Cell Prolif. *49*, 421–437.

46. Guo, T., Zuo, Y., Qian, L., Liu, J., Yuan, Y., Xu, K., Miao, Y., Feng, Q., Chen, X., Jin, L., et al. (2019). ADP-ribosyltransferase PARP11 modulates the interferon antiviral response by mono-ADP-ribosylating the ubiquitin E3 ligase β-TrCP. Nat. Microbiol. *4*, 1872–1884.

47. Martines, R.B., Ng, D.L., Greer, P.W., Rollin, P.E., and Zaki, S.R. (2015). Tissue and cellular tropism, pathology and pathogenesis of Ebola and Marburg viruses. J. Pathol. *235*, 153–174.

48. Perry, D.L., Huzella, L.M., Bernbaum, J.G., Holbrook, M.R., Jahrling, P.B., Hagen, K.R., Schnell, M.J., and Johnson, R.F. (2018). Ebola Virus Localization in the Macaque Reproductive Tract during Acute Ebola Virus Disease. Am. J. Pathol. *188*, 550–558.

49. Liu, D.X., Cooper, T.K., Perry, D.L., Huzella, L.M., Hischak, A.M.W., Hart, R.J., Isic, N., Byrum, R., Ragland, D., St Claire, M., et al. (2022). Expanded Histopathology and Tropism of Ebola Virus in the Rhesus Macaque Model: Potential for Sexual Transmission, Altered Adrenomedullary Hormone Production, and Early Viral Replication in Liver. Am. J. Pathol. *192*, 121–129.

50. Audet, J., and Kobinger, G.P. (2015). Immune evasion in ebolavirus infections. Viral Immunol. *28*, 10–18.

51. Pleet, M.L., DeMarino, C., Lepene, B., Aman, M.J., and Kashanchi, F. (2017). The Role of Exosomal VP40 in Ebola Virus Disease. DNA Cell Biol. *36*, 243–248.

52. Jain, S., Martynova, E., Rizvanov, A., Khaiboullina, S., and Baranwal, M. (2021). Structural and Functional Aspects of Ebola Virus Proteins. Pathogens *10*, 1330.

53. Watt, A., Moukambi, F., Banadyga, L., Groseth, A., Callison, J., Herwig, A., Ebihara, H., Feldmann, H., and Hoenen, T. (2014). A novel life cycle modeling system for Ebola virus shows a genome length-dependent role of VP24 in virus infectivity. J. Virol. *88*, 10511–10524.

54. Chan, M., Leung, A., Griffin, B.D., Vendramelli, R., Tailor, N., Tierney, K., Audet, J., and Kobasa, D. (2019). Generation and Characterization of a Mouse-Adapted Makona Variant of Ebola Virus. Viruses *11*.

55. Ebihara, H., Takada, A., Kobasa, D., Jones, S., Neumann, G., Theriault, S., Bray, M., Feldmann, H., and Kawaoka, Y. (2006). Molecular determinants of Ebola virus virulence in mice. PLoS Pathog. *2*, e73.

56. Bray, M., Davis, K., Geisbert, T., Schmaljohn, C., and Huggins, J. (1998). A mouse model for evaluation of prophylaxis and therapy of Ebola hemorrhagic fever. J. Infect. Dis. *178*, 651–661.

57. Wang, H., Shi, Y., Song, J., Qi, J., Lu, G., Yan, J., and Gao, G.F. (2016). Ebola Viral Glycoprotein Bound to Its Endosomal Receptor Niemann-Pick C1. Cell *164*, 258–268.

58. Lee, J.E., Fusco, M.L., Hessell, A.J., Oswald, W.B., Burton, D.R., and Saphire, E.O. (2008). Structure of the Ebola virus glycoprotein bound to an antibody from a human survivor. Nature *454*, 177–182.

59. Dietzel, E., Schudt, G., Krähling, V., Matrosovich, M., and Becker, S. (2017). Functional Characterization of Adaptive Mutations during the West African Ebola Virus Outbreak. J. Virol. *91*, e01913-16.

60. Wong, G., He, S., Leung, A., Cao, W., Bi, Y., Zhang, Z., Zhu, W., Wang, L., Zhao, Y., Cheng, K., et al. (2019). Naturally Occurring Single Mutations in Ebola Virus Observably Impact Infectivity. J. Virol. *93*, e01098-18.

61. Yuan, B., Peng, Q., Cheng, J., Wang, M., Zhong, J., Qi, J., Gao, G.F., and Shi, Y. (2022). Structure of the Ebola virus polymerase complex. Nature *610*, 394–401.

62. Peters, C.J., and Zaki, S.R. (2011). Overview of viral hemorrhagic fevers. In Tropical Infectious Diseases: Principles, Pathogens and Practice, 3rd ed., R.L. Guerrant, D.H. Walker, and P.F. Weller, eds. (Philadelphia: Elsevier), pp. 441–448.

63. Zaki, S.R., and Goldsmith, C.S. (1999). Pathologic features of filovirus infections in humans. Curr. Top. Microbiol. Immunol. *235*, 97–116.

64. Ellis, D.S., Simpson, I.H., Francis, D.P., Knobloch, J., Bowen, E.T., Lolik, P., and Deng, I.M. (1978). Ultrastructure of Ebola virus particles in human liver. J. Clin. Pathol. *31*, 201–208.

65. Schnittler, H.J., and Feldmann, H. (1998). Marburg and Ebola hemorrhagic fevers: does the primary course of infection depend on the accessibility of organ-specific macrophages? Clin. Infect. Dis. *27*, 404–406.

66. Geisbert, T.W., Young, H.A., Jahrling, P.B., Davis, K.J., Larsen, T., Kagan, E., and Hensley, L.E. (2003). Pathogenesis of Ebola hemorrhagic fever in primate models: evidence that hemorrhage is not a direct effect of virus-induced cytolysis of endothelial cells. Am. J. Pathol. *163*, 2371–2382.

67. Keita, A.K., Koundouno, F.R., Faye, M., Düx, A., Hinzmann, J., Diallo, H., Ayouba, A., Le Marcis, F., Soropogui, B., Ifono, K., et al. (2021). Resurgence of Ebola virus in 2021 in Guinea suggests a new paradigm for outbreaks. Nature *597*, 539–543.

68. Liu, J., Trefry, J.C., Babka, A.M., Schellhase, C.W., Coffin, K.M., Williams, J.A., Raymond, J.L.W., Facemire, P.R., Chance, T.B., Davis, N.M., et al. (2022). Ebola virus persistence and disease recrudescence in the brains of antibody-treated nonhuman primate survivors. Sci. Transl. Med. *14*, eabi5229.

69. Zeng, X., Blancett, C.D., Koistinen, K.A., Schellhase, C.W., Bearss, J.J., Radoshitzky, S.R., Honnold, S.P., Chance, T.B., Warren, T.K., Froude, J.W., et al. (2017). Identification and pathological characterization of persistent asymptomatic Ebola virus infection in rhesus monkeys. Nat. Microbiol. *2*, 17113.

70. Park, D.J., Dudas, G., Wohl, S., Goba, A., Whitmer, S.L.M., Andersen, K.G., Sealfon, R.S., Ladner, J.T., Kugelman, J.R., Matranga, C.B., et al. (2015). Ebola Virus Epidemiology, Transmission, and Evolution during Seven Months in Sierra Leone. Cell *161*, 1516–1526.

71. Tong, Y.-G., Shi, W.-F., Liu, D., Qian, J., Liang, L., Bo, X.-C., Liu, J., Ren, H.-G., Fan, H., Ni, M., et al. (2015). Genetic diversity and evolutionary dynamics of Ebola virus in Sierra Leone. Nature *524*, 93–96.

72. Polack, F. (2019). Faculty Opinions recommendation of Safety, Tolerability, and Pharmacokinetics of MEDI8897, the Respiratory Syncytial Virus Prefusion F-Targeting Monoclonal Antibody with an Extended Half-Life. In Healthy Adults (Faculty Opinions – Post-Publication Peer Review of the Biomedical Literature).

73. Basu, A., Li, B., Mills, D.M., Panchal, R.G., Cardinale, S.C., Butler, M.M., Peet, N.P., Majgier-Baranowska, H., Williams, J.D., Patel, I., et al. (2011). Identification of a small-molecule entry inhibitor for filoviruses. J. Virol. *85*, 3106–3119.

74. Warren, T.K., Wells, J., Panchal, R.G., Stuthman, K.S., Garza, N.L., Van Tongeren, S.A., Dong, L., Retterer, C.J., Eaton, B.P., Pegoraro, G., et al. (2014). Protection against filovirus diseases by a novel broad-spectrum nucleoside analogue BCX4430. Nature *508*, 402–405.

75. Oestereich, L., Lüdtke, A., Wurr, S., Rieger, T., Muñoz-Fontela, C., and Günther, S. (2014). Successful treatment of advanced Ebola virus infection with T-705 (favipiravir) in a small animal model. Antivir. Res. *105*, 17–21.

76. Warren, T.K., Jordan, R., Lo, M.K., Ray, A.S., Mackman, R.L., Soloveva, V., Siegel, D., Perron, M., Bannister, R., Hui, H.C., et al. (2016). Therapeutic efficacy of the small molecule GS-5734 against Ebola virus in rhesus monkeys. Nature *531*, 381–385.

77. Afroz, S., Giddaluru, J., Abbas, M.M., and Khan, N. (2016). Transcriptome meta-analysis reveals a dysregulation in extra cellular matrix and cell junction associated gene signatures during Dengue virus infection. Sci. Rep. *6*, 33752.

78. Herrera, C., Escalante, T., Voisin, M.-B., Rucavado, A., Morazán, D., Macêdo, J.K.A., Calvete, J.J., Sanz, L., Nourshargh, S., Gutiérrez, J.M., and Fox, J.W. (2015). Tissue localization and extracellular matrix degradation by PI, PII and PIII snake venom metalloproteinases: clues on the mechanisms of venom-induced hemorrhage. PLoS Neglected Trop. Dis. *9*, e0003731.

79. Wang, P., Dai, J., Bai, F., Kong, K.-F., Wong, S.J., Montgomery, R.R., Madri, J.A., and Fikrig, E. (2008). Matrix metalloproteinase 9 facilitates West Nile virus entry into the brain. J. Virol. *82*, 8978–8985.

80. Talmi-Frank, D., Altboum, Z., Solomonov, I., Udi, Y., Jaitin, D.A., Klepfish, M., David, E., Zhuravlev, A., Keren-Shaul, H., Winter, D.R., et al. (2016). Extracellular Matrix Proteolysis by MT1-MMP Contributes to Influenza-Related Tissue Damage and Mortality. Cell Host Microbe *20*, 458–470.

81. Carroll, M.W., Haldenby, S., Rickett, N.Y., Pályi, B., Garcia-Dorival, I., Liu, X., Barker, G., Bore, J.A., Koundouno, F.R., Williamson, E.D., et al. (2017). Deep Sequencing of RNA from Blood and Oral Swab Samples Reveals the Presence of Nucleic Acid from a Number of Pathogens in Patients with Acute Ebola Virus Disease and Is Consistent with Bacterial Translocation across the Gut. mSphere *2*, e00325-17.

82. Reisler, R.B., Zeng, X., Schellhase, C.W., Bearss, J.J., Warren, T.K., Trefry, J.C., Christopher, G.W., Kortepeter, M.G., Bavari, S., and Cardile, A.P. (2018). Ebola Virus Causes Intestinal Tract Architectural Disruption and Bacterial Invasion in Non-Human Primates. Viruses *10*.

83. Trombley, A.R., Wachter, L., Garrison, J., Buckley-Beason, V.A., Jahrling, J., Hensley, L.E., Schoepp, R.J., Norwood, D.A., Goba, A., Fair, J.N., and Kulesh, D.A. (2010). Comprehensive panel of real-time TaqMan polymerase chain reaction assays for detection and absolute quantification of filoviruses, arenaviruses, and New World hantaviruses. Am. J. Trop. Med. Hyg. *82*, 954–960.

84. Matranga, C.B., Andersen, K.G., Winnicki, S., Busby, M., Gladden, A.D., Tewhey, R., Stremlau, M., Berlin, A., Gire, S.K., England, E., et al. (2014). Enhanced methods for unbiased deep sequencing of Lassa and Ebola RNA viruses from clinical and biological samples. Genome Biol. *15*, 519.

85. Levin, J.Z., Yassour, M., Adiconis, X., Nusbaum, C., Thompson, D.A., Friedman, N., Gnirke, A., and Regev, A. (2010). Comprehensive comparative analysis of strand-specific RNA sequencing methods. Nat. Methods *7*, 709–715.

86. MacConaill, L.E., Burns, R.T., Nag, A., Coleman, H.A., Slevin, M.K., Giorda, K., Light, M., Lai, K., Jarosz, M., McNeill, M.S., et al. (2018). Unique, dual-indexed sequencing adapters with UMIs effectively eliminate index cross-talk and significantly improve sensitivity of massively parallel sequencing. BMC Genom. *19*, 30.

87. Chandran, K., Sullivan, N.J., Felbor, U., Whelan, S.P., and Cunningham, J.M. (2005). Endosomal proteolysis of the Ebola virus glycoprotein is necessary for infection. Science *308*, 1643–1645.

88. Sanchez, A., Trappier, S.G., Mahy, B.W., Peters, C.J., and Nichol, S.T. (1996). The virion glycoproteins of Ebola viruses are encoded in two reading frames and are expressed through transcriptional editing. Proc. Natl. Acad. Sci. USA *93*, 3602–3607.

89. Dobin, A., Davis, C.A., Schlesinger, F., Drenkow, J., Zaleski, C., Jha, S., Batut, P., Chaisson, M., and Gingeras, T.R. (2013). STAR: ultrafast universal RNA-seq aligner. Bioinformatics *29*, 15–21.

90. Smith, T., Heger, A., and Sudbery, I. (2017). UMI-tools: modeling sequencing errors in Unique Molecular Identifiers to improve quantification accuracy. Genome Res. *27*, 491–499.

91. Durinck, S., Moreau, Y., Kasprzyk, A., Davis, S., De Moor, B., Brazma, A., and Huber, W. (2005). BioMart and Bioconductor: a powerful link between biological databases and microarray data analysis. Bioinformatics *21*, 3439–3440.

92. Wilm, A., Aw, P.P.K., Bertrand, D., Yeo, G.H.T., Ong, S.H., Wong, C.H., Khor, C.C., Petric, R., Hibberd, M.L., and Nagarajan, N. (2012). LoFreq: a sequence-quality aware, ultra-sensitive variant caller for uncovering cell-population heterogeneity from high-throughput sequencing datasets. Nucleic Acids Res. *40*, 11189–11201.

93. Love, M.I., Huber, W., and Anders, S. (2014). Moderated estimation of fold change and dispersion for RNA-seq data with DESeq2. Genome Biol. *15*, 550.

94. Alexa, A., Rahnenführer, J., and Lengauer, T. (2006). Improved scoring of functional groups from gene expression data by decorrelating GO graph structure. Bioinformatics *22*, 1600–1607. https://doi.org/10.1093/bioinformatics/btl140.

95. Fischer, D.S., Theis, F.J., and Yosef, N. (2018). Impulse model-based differential expression analysis of time course sequencing data. Nucleic Acids Res. *46*, e119.

96. Kolde, R. Pheatmap: pretty heatmaps. R package, version 1.0.12. Published January 4, 2019.

97. Bingham, E., Chen, J.P., Jankowiak, M., Obermeyer, F., Pradhan, N., Karaletsos, T., Singh, R., Szerlip, P., Horsfall, P., and Goodman, N.D. (2018). Pyro: Deep Universal Probabilistic Programming. Preprint at arXiv.

98. Fan, J., Lyu, Y., Zhang, Q., Wang, X., Li, M., and Xiao, R. (2022). MuSiC2: cell-type deconvolution for multi-condition bulk RNA-seq data. Briefings Bioinf. *23*, bbac430.

99. Han, L., Wei, X., Liu, C., Volpe, G., Zhuang, Z., Zou, X., Wang, Z., Pan, T., Yuan, Y., Zhang, X., et al. (2022). Cell transcriptomic atlas of the non-human primate Macaca fascicularis. Nature *604*, 723–731.

STAR★METHODS

KEY RESOURCES TABLE

REAGENT or RESOURCE	SOURCE	IDENTIFIER
**Bacterial and virus strains**		
Ebola virus/H. sapiens-tc/COD/1995/Kikwit-9510621 (EBOV/Kikwit; GenBank accession MG572235.1; *Filoviridae*: *Zaire ebolavirus*)	BEI Resources	Cat#NR-50306
**Biological samples**		
Monkey Adrenal Total RNA, Rhesus	Zyagen	UR-501
Monkey Brain Total RNA, Rhesus	Zyagen	UR-201
Monkey Kidney Total RNA, Rhesus	Zyagen	UR-901
Monkey Liver Total RNA, Rhesus	Zyagen	UR-314
Monkey Lymph nodes Total RNA, Rhesus	Zyagen	UR-703
Monkey Skin Total RNA Total RNA, Rhesus	Zyagen	UR-101
Monkey Spinal cord Total RNA, Rhesus	Zyagen	UR-230
Monkey Spleen Total RNA, Rhesus	Zyagen	UR-701
**Chemicals, peptides, and recombinant proteins**		
X-tremeGENE 9 DNA Transfection Reagent	Sigma-Aldrich	6365787001
Actinomycin D	Millipore Sigma	A1410-2MG
2′-Deoxyuridine 5′-triphosphate sodium salt (dUTP)	Millipore Sigma	D0184-25UMO
NEBNext Ultra II End Repair/dA-Tailing Module	NEB	E7546L
Instant Sticky-end Ligase Master Mix	NEB	M0370L
Thermolabile USER II Enzyme	NEB	M5508L
**Critical commercial assays**		
Q5 Site-Directed Mutagenesis Kit	New England Biolabs	E0554S
**Deposited data**		
EBOV NHP infection RNA-Seq reads	This study	GSE226106
Macaca fascicularis single-cell reference data	[83]	https://db.cngb.org/nhpca/download
RNA-seq data for healthy and diseased pancreatic islet samples	[36]	GSE50244
pancreatic islets scRNA-seq RNA-seq data	[37]	E-MTAB-5061
Peripheral blood data from the same EBOV-infected rhesus monkeys	[18]	GSE158390
**Experimental models: Cell lines**		
HEK293T	ATCC	CRL-3216
U2OS	ATCC	HTB-96
**Oligonucleotides**		
See Table S5		N/A
**Recombinant DNA**		
See Table S5		N/A
**Software and algorithms**		
Bulk RNA-seq Processing	This study	https://github.com/broadinstitute/EbolaNaturalHistory/
ternaDecov	This study	https://doi.org/10.5281/zenodo.8411808
STAR	[84]	https://github.com/alexdobin/STAR
python	Python core team	https://www.python.org/
R	R Core Team	https://www.r-project.org/
UMI-tools	[85]	https://github.com/CGATOxford/UMI-tools

(*Continued on next page*)

Continued

REAGENT or RESOURCE	SOURCE	IDENTIFIER
BioMart	[86]	https://github.com/grimbough/biomaRt
viral-ngs	https://viral-ngs.readthedocs.io/en/latest/index.html	https://github.com/broadinstitute/viral-ngs
DESeq2	[87]	https://bioconductor.org/packages/release/bioc/html/DESeq2.html
go.db.df	[88]	https://bioconductor.org/packages/release/data/annotation/html/GO.db.html
topGO	[89]	https://bioconductor.org/packages/release/bioc/html/topGO.html
ImpulseDE2	[90]	https://github.com/YosefLab/ImpulseDE2
MuSiC	[32]	https://xuranw.github.io/MuSiC/articles/MuSiC.html

RESOURCE AVAILABILITY

Lead contact

Further information and requests for resources and reagents should be directed to Katherine Siddle (katherine_siddle@brown.edu).

Materials availability

Plasmids generated in this study are available upon request.

Data and code availability

The RNA-Seq datasets reported in this paper are available in GEO under accession GSE226106. The scripts used in this study are available at https://github.com/broadinstitute/temporal-rna-seq-deconvolution/and https://github.com/broadinstitute/EbolaNaturalHistory/. The version of ternaDecov used in this study is available at https://doi.org/10.5281/zenodo.8411808.

EXPERIMENTAL MODEL AND SUBJECT DETAILS

This study included a subset (21 of 27) outbred rhesus monkeys (*Macaca mulatta*) of Chinese origin described recently,[18,28] balancing age, weight, and sex (8 males and 13 females). All work was approved and performed in accordance with the Guide for the Care and Use of Laboratory Animals of the National Institute of Health, the Office of Animal Welfare, and the US Department of Agriculture.

HEK293 (human [Homo sapiens] fetal kidney) and U2OS (human [Homo sapiens] osteosarcoma) were obtained from the ATCC (https://www.atcc.org/). Cells were maintained in DMEM containing 10% fetal bovine serum, 1% non-essential amino acids, 1% sodium pyruvate, and 1% penicillin-streptomycin at 37°C with 5% CO2 and seeded onto coated plates for transfection experiments described in details below.

METHOD DETAILS

Natural history study

The details regarding the infecting viral stock and animals used have been published previously.[28] Briefly, 18 rhesus monkeys were inoculated intramuscularly with 1 mL of 1000 plaque-forming units/mL EBOV/Kikwit (Ebola virus/Homo sapiens-terminal control-COD/1995/Kikwit-9510621 from BEI Resources, Manassas, VA) in the left lateral triceps muscle at study day 0. Animals were humanely euthanized at either a predetermined time point (3 animals on each of days 3, 4, 5 and 6 post-infection) or at terminal endpoint (N = 6). Sequential blood draws under general anesthetic were collected for the 6 animals in the terminal endpoint group. Three uninfected control monkeys (2 female, 1 male) were sham-exposed with 1 mL phosphate-buffered saline at the same anatomic location before sacrifice on day 0. Baseline blood draws at approximately 30 and 14 days prior to infection were collected for all 21 animals. Tissue samples were collected from each animal at necropsy in bead beater tubes and homogenized in TRIzol and inactivated in TRIzol LS.

All monkeys used in this research project were cared for and used humanely according to the following policies: the U.S. Public Health Service Policy on Humane Care and Use of Animals (2000); NIH's Guide for the Care and Use of Laboratory Animals; and the U.S. Government Principles for Utilization and Care of Vertebrate Animals Used in Testing, Research, and Training (1985). All National Institute of Allergy and Infectious Diseases Integrated Research Facility animal facilities and programs are accredited by the Association for Assessment and Accreditation of Laboratory Animal Care International. This study was performed in the Biosafety Level 4 Laboratory at the NIH/National Institute of Allergy and Infectious Diseases, Integrated Research Facility at Fort Detrick (Fredrick, MD).

Sample extraction and RNA purification

Tissue homogenates inactivated in TRIzol were phase-separated with chloroform at the Broad Institute, and total RNA was extracted from the aqueous phase using the MagMAX MirVana total RNA kit (ThermoFisher) on a KingFisher FLEX instrument. DNA was removed by TURBO DNase treatment following RNA extraction. A TRIzol-inactivated aliquot of the viral seed stock injected into animals from this study was also obtained and extracted with the Direct-zol-96 MagBead RNA (Zymo Research).

Quantification of viral RNA

Ebola viral load in all extracted RNA samples was measured by qRT-PCR using an SYBR Green assay with previously published primers targeting the EBOV NP gene.[83] A standard curve of a DNA gBlock (IDT) encoding the target region was used to calculate viral copy numbers. Curves of temporal change in viral load in each tissue were clustered using iterative K-means longitudinal data clustering with the R package KLM with maximum number of NA tolerates per trajectory of 1.

Library construction and sequencing

We depleted ribosomal RNA from purified RNA using an RNase H-based approach,[84] then performed strand-specific ligation-based library construction.[85] Briefly, we heat-fragmented RNA, performed reverse transcription, labeled second-strand cDNA with dUTP, then ligated xGen UDI-UMI adapters[86] at a concentration of 0.04 μM for fluid samples and viral seed stock, and 0.2 μM for tissue samples. We then USER-digested the dUTP-labeled strand, and PCR amplified libraries. Libraries were quantified with TapeStation high-sensitivity DNA assay (Agilent). Samples were pooled at equimolar ratios and sequenced on a NovaSeq SP (Illumina) with 2x146bp cycles for the cDNA and 17 cycles of Index Read 1 to sequence the 9-base UMI.

Pentacistronic minigenome assay

We constructed a EBOV/Kikwit pentacistronic (5MG) minigenome system based on a previously published EBOV/Mak-C15 tetracistronic (4MG) minigenome system[22] but cloned in EBOV/Kikwit sequences either amplified by RT-PCR from viral seed stock or ordered as dsDNA gBlocks (IDT) to replace EBOV/Mak-C15 genes. The EBOV/Kikwit 5MG plasmid includes eGFP and nano luciferase as reporter genes and VP40, GP, and VP24 CDS and UTRs. EBOV/Kikwit L and VP30 were cloned into pcDNA3.4 vectors to facilitate site directed mutagenesis (SDM) experiments as pCAGGs vectors from the published system have GC-rich regions that are difficult to amplify under standard PCR conditions. SDM was performed to create single nucleotide variants following manufacturer's protocol (NEB) with custom designed primers (Table S5). Full plasmid sequences are in Data S1.

We followed an existing protocol for the multicistronic minigenome assay[53] with some modifications. We seeded HEK 293T cells into collagen-coated 24-well plates, grew to 60% confluency, and transfected cells following the xtremegene9 transfection protocol with the previously described plasmid ratio (31.25 ng of NP-P2A-VP35, 18.75 ng of VP30, 250 ng of L, 62.5 ng of 5MG plasmid encoding eGFP, 62.5ng of T7pol). We harvested cells 48 h post-transfection with trypsin, washed once with PBS and stained with DAPI for cell viability. We then measured the percentage of eGFP positive live cells for each condition which we considered as infected host cells.

GP-pseudotyped lentivirus and infectivity assays

The following mutants were selected for a GP-pseudotyping assay: S65A, S65P, H139R, N278Y, and N506T. A gBlock for the EBOV GP seed stock (GenBank: KU182908.1) was designed and synthesized (IDT) with a deleted mucin like domain from amino acid positions 309–489 and an additional adenosine at nucleotide position 890 to produce the full length glycoprotein.[21,87,88] This gBlock was cloned into the pGL4.23 backbone expression plasmid described in Diehl et al. using restriction enzymes with the GP sequence placed under the control of a cytomegalovirus immediate-early (CMV IE) promoter/enhancer.[21] Q5 Site directed mutagenesis (NEB) was used to introduce the mutations in the backbone.

GP-pseudotyped lentiviral virions carrying an EFS driven H2B-mCherry reporter gene were produced in triplicate by transfecting HEK293FT cells (Takara, Cat# 632180) using polyethylenimine (PEI, Polysciences Cat# 24765–1) with 800 ng GP envelope, 866 ng psPAX2, and 1,333 ng H2B-mCherry reporter plasmid. Media was exchanged 4 h after transfection and viral supernatants were collected 2 days later. The viral supernatant was filtered through a 0.4μm filter (Pall, Cat# 8129), treated with Benzonase-nuclease (Sigma-Aldrich, Cat# E1014-25KU) for 1 h at 37°C after which viral RNA was extracted using a Zymo RNA extraction kit according to manufacturers protocols (Zymo, Cat# R1041). An qRT-PCR was run to determine the titer of each sample using the Takara Lenti-X Quant RT-qPCR kit (Takara Bio, Cat#: 631235). Viral supernatants were normalized to the same multiplicity of infection for infectivity assays.

U2OS cells were maintained in DMEM containing 10% fetal bovine serum, 1% non-essential amino acids, 1% sodium pyruvate, and 1% penicillin-streptomycin at 37°C with 5% CO_2. U2OS cells were plated in 96-well plates at 7,500 cells per well and the normalized viral supernatant was added to the plate in duplicate. Media was exchanged 24 h later and then cells were analyzed by flow cytometry after 4 days.

Sequencing data preprocessing and quality control

Host transcriptomics data was processed using the umiRNAseq custom pipeline for Bulk RNA-seq Processing with UMI correction on Terra (https://github.com/broadinstitute/EbolaNaturalHistory/blob/main/00-bulk-rna-seq/umiRNASeq.wdl). Briefly, we merged

and tagged raw Fastq files with their corresponding UMI barcode, and mapped, using the STAR aligner,[89] to the rhesus monkey (*Macaca mulatta)* reference genome and annotation (Mmul_10). Resulting BAM files were filtered for multiple mapped reads, sorted and indexed using samtools. Then, PCR duplicates were removed by UMI-tools[90] using the UMI barcodes of each transcript, and featureCounts were used to quantify expression from the aligned and processed RNA-Seq BAM files. We used the BioMart R package[91] to annotate the gene type, gene name, and gene function using the ensembl *M. mulatta* database "mmulatta_gene_ensembl". Quality control over the sample was performed removing samples with low sequencing quality and mismatched sex assignment.

Viral genomic analyses

Viral genomic analyses were performed using viral-ngs pipelines (https://github.com/broadinstitute/viral-ngs) implemented on the Terra platform (app.terra.bio). We assembled EBOV genomes using the assemble_refbased workflow (viral-ngs version 2.0.21), with the EBOV/Kikwit reference GenBank: KU182908.1. Genomes with >95% unambiguous bases were considered complete. On all genomes with >400x mean depth of coverage, we used LoFreq with -q 20 and -Q 20 to identify minor variants, relative to the EBOV reference GenBank: KU182908.1.[92] We filtered out variants that were present in <2% or >98% of reads mapping to a given position (relative to reference), as well as those at sites with depth of coverage <100 and variant reads <5.

Viral mutation statistics

A one-tailed exact binomial test with $p = 0.75$ was used to determine whether the ratio of nonsynonymous to synonymous mutations in a given analysis differed from the expected 3:1 ratio for neutral selection.These analyses were done within a tissue across all genes, and also with respect to a particular gene across all tissues. A one-tailed permutation test (with 10,000 trials) was used to determine whether the ratio of nonsynonymous to synonymous mutations differed between high-frequency and low-frequency variants.

Differential expression analysis

The raw read counts of all samples were normalized using the DESeq2 R package.[93] In order to identify tissue markers, we compared counts from samples at time zero and 3 days post infection (DPI) using a model matrix to compare each tissue against all others. Genes with an adjusted p-Value and a log2 fold change higher than one in each comparison were selected as tissue markers for that specific tissue.

To identify differentially expressed genes between not infected (samples at 0 DPI) and infected conditions, samples were further analyzed with the DESeq2 package.[93] For tissues lacking samples at 0 DPI (lung, liver and testis) samples at 3 DPI were used instead. For each tissue, genes previously identified as tissue markers were excluded from downstream interpretation. We considered differentially expressed genes (DEGs) to be those genes with a p-adj <0.05 and a log2 fold change higher than 2. Genes meeting these criteria were stratified into ISGs, Cytokines, Inflammatory response, PARPs, apoptosis, and extracellular matrix related genes using the go.db.df R package and custom lists.

GO term enrichment analysis and correlation analysis

Enrichment analysis was performed on DEGs using the R package topGO[94] with the "Biological Process" ontology. For each tissue, we selected the top 100 DEGs across time (FDR <0.01) for this analysis. We selected the top 3 enriched terms for each tissue as defined by the p values of the Kolmogorov-Smirnov test. Correlation between host genes and viral counts was performed using the normalized DESeq2 counts and the total viral read counts using Spearman rank correlation analysis as implemented in the stats R package. A similar approach was performed for the correlation between viral load and monocyte markers (mean of *CTSS*, *VCAN*, *FCN1*, *CD14*, *S100A9*, *MS4A1* normalized counts) and whole-blood non-monocyte markers (mean of *CD3D*, *HBA*, *SELL*, *PPBP*, *HBA*, *CD8A*, *GNLY* normalized counts).

Genes expression changes across time

To identify genes changing across time, we used the ImpulseDE2 package[95] to perform a time-series differential expressed gene analysis of each tissue across the 8 days of infection. ImpulseDE2 includes a DEseq2 normalization step, thus, the raw gene read counts from FeatureCounts were used as input data. The function "runImpulseDE2" was applied to each tissue independently, significant genes were selected as those with a p-adj <0.05. Furthermore tissue marker genes corresponding to each tissue were excluded from downstream analysis.

The data analysis mentioned before were performed in R version 4.1.2, using the aforementioned R packages. Visualization was performed using the Packages ggplot2, Pheatmap[96] and ComplexHeatmap.

Time-regularized deconvolution of bulk RNA sequencing (ternaDecov)

We developed ternaDecov as a time-regularized method for deconvolution of bulk sequencing data using scRNA-seq reference data. Briefly, ternaDecov uses stochastic variational inference to simultaneously identify an underlying trajectory of cellular composition change in terms of user-specified covariates (e.g., days post infection) and deconvolve individual sample compositions using annotated single-cell profiles. The code for the ternaDecov software is available from github at https://github.com/broadinstitute/temporal-rna-seq-deconvolution as an installable python package and several introductory tutorials are provided.

TernaDecov offers a modular model structure in which the cell type proportions of each sample are obtained from one of several alternative trajectory modules. The trajectory modules take as input the sampling time covariate and return a draw of sample-specific cell proportions (π_{nc}) as a result in different ways depending on their internal structure. Trajectory modules currently implemented in ternaDeCov include: (1) simple polynomial trajectories, (2) Legendre polynomial trajectories, (3) Gaussian process with different kernel functions, and (4) a "trivial" trajectory model that does not take into account sample collection time, effectively producing independent deconvolution of samples similar to traditional deconvolution algorithms.

The cell-type proportions (π_{nc}) are multiplied with the summarized single-cell reference (…) after scaling by learnable gene specific capture rate coefficients (β_g) to produce location parameter for a Negative Binomial distribution from which the observed count matrix is sampled from using gene specific dispersion parameters (φ_g).

The full model is specified as follows:

$$n : sample\ index$$

$$c : celltype\ index$$

$$g : gene\ index$$

$$N_n : total\ library\ size$$

$$\tau_n : sampling\ times$$

$$\widehat{W}_{gc} : summarized\ single - cell\ reference$$

$$\mu_g : gene - specific\ dispersion\ mean$$

$$\sigma_g : gene - specific\ dispersion\ variance$$

$$\sigma_\beta : gene - specific\ capture\ rate\ variance$$

$$W_{gc} = \beta_g \widehat{W}_{gc}$$

$$\varphi_g = \mathcal{N}(\mu_g, \sigma_g)$$

$$\beta_g = \mathcal{N}(0, \sigma_\beta)$$

$$\pi_{nc} = Trajectory\ Module(\tau_n)$$

$$X_{ng} = NB(N_n \pi_{nc} \widehat{W}_{cg}, \varphi_g)$$

ternaDecov: Trajectory models

TernaDecov offers two trajectory models, described below.

Polynomial trajectory model

The polynomial trajectory model is shown in Figure S14A (left). To obtain the prior cell proportions for a given sample n at time τ_n, we evaluate a specified polynomial function basis $\varphi_k(.)$ for $k = 1, \ldots, K$ on τ_n to obtain a polynomial feature matrix $\varphi_k(\tau_n)$. At the same time, we (globally) sample a set of weights $z_{kc} \sim \mathcal{N}(0, {\alpha_k}^{-1})$, where α_k is the precision of prior Gaussian and controls the usage of basis function φ_k. We matrix multiply the global weights with the sample polynomial feature matrix to obtain the unnormalized cell population $y_{nc} = \sum_{k=1}^{K} z_{kc}\varphi_k(\tau_n)$. We normalize the latter by applying the softmax function along the last dimension to obtain $\widehat{\pi}_{nc} = softmax(y_{nc})$. To allow sample-specific deviations from this prior trajectory, we finally sample π_{nc} from a Dirichlet distribution $\pi_{nc} \sim Dirichlet(\alpha_{dir}\widehat{\pi}_{nc})$. Here, α_{dir} is the global Dirichlet concentration parameter which controls how sample trajectories can deviate from the prior trajectory.

Gaussian process (GP) trajectory model

In contrast to the polynomial model, the GP model (Figure S14A, right) allows for more flexible trajectories. The function space of trajectories is specified by the kernel function, and the parameters of the kernel function are optimized to obtain the maximum likelihood trajectory fit. To obtain the prior cell proportions for a given sample n at time τ_n, we draw unnormalized cell proportions y_{nc} independently for each cell type using a cell-type-specific GP and sample collection time τ_n as the covariate. We specifically used radial basis function (RBF) kernel function with added white noise $k(\tau, \tau') = \sigma_0 \, exp(-|\tau - \tau'|^2 / 2T^2) + \sigma_1 \, \delta(\tau, \tau')$, where $\theta_{GP} = \{\sigma_0, \sigma_1, T\}$ constitute the set of GP kernel parameters to be optimized. Intuitively, a larger choice of σ_1 allows for more sample-to-sample trajectory deviation, a larger choice of σ_0 couples adjacent times more strongly together (i.e., stronger time regularization), and T sets the trajectory correlation timescale. Like before, we normalize the unnormalized cell population y_{nc} by applying the softmax function along the last dimension to obtain $\pi_{nc} = softmax(y_{nc})$. In contrast to the polynomial model, y_{nc} is already a latent variable which accommodates for sample-to-sample deviation from the trajectory. Therefore, sampling from the Dirichlet distribution is no longer needed in this approach.

ternaDecov: Implementation

TernaDecov is implemented in python as a hierarchical model using the pyro[97] probabilistic programming framework. When available, ternaDecov can utilize underlying CUDA graphics processors for acceleration. Parameter estimation is performed using the Adam with a learning rate of 1e-3 optimizer and an ELBO loss; 20,000 learning iterations are utilized unless noted otherwise. TernaDecov can be run using a CLI interface or via API calls using a jupyter notebook. Inputs for ternaDecov execution encompass two scanpy AnnData objects: one for the single-cell reference (that requires a cell type annotation column) and one for the bulk data that requires a column annotating the time of collection of each sample. The results can be exported in tabular format as well as plotter in raster and vector formats.

The package provides facilities for simulating random sample proportion trajectories using different basis functions that are different in functional form from the bases used to estimate trajectories and include softmax normalized sigmoid, sinusoidal and linear (first degree polynomial) trajectories, using the Simulator module. Furthermore, the package allows for automated scanning of prior parameters and configuration options for assessing stability of results with respect to these values, using the SensitivityAnalyzer module.

ternaDecov: Technical benchmarking

Run time

We benchmarked runtime performance using simulated samples from a fixed random trajectory (Figure S14B). Furthermore, deconvolution of 10 adrenal samples with ternaDecov required 4.7 min, MuSic accomplished the same task in 57.9 min. Although scaling with the number of samples is exponential, running time for 1000 samples is sufficiently short to be run interactively. Scaling of the polynomial trajectory module is more linear that the full GP shown here. We anticipate that memory limitations will be more important than execution time when utilizing the GP model. We found that executing the model using a GPU processor accelerated execution (data not shown).

Accuracy

We assessed the value of i) increasing sample number and ii) trajectory estimation on improving sample composition estimates with ternaDecov. Using the built-in simulator we assessed the ability of ternaDecov to recover underlying trajectories from which bulk samples are derived as function of the number of equidistant temporal samples obtained. We generated a single random fixed periodic type of trajectory (Figure S14C) and increasingly sampled N equidistant samples from it. After learning the underlying trajectory we evaluated composition values as 1000 points and scored trajectory reconstruction quality by means of normalized L1 error. L1 error declined with increasing sample numbers, indicating that larger sample sizes improve trajectory estimation (Figure S14D).

Sample proportion and simultaneous trajectory estimation is expected to reduce the error of individual sample proportion estimation as information between samples is shared. In order to confirm that, we deconvolved fixed trajectory using the 'gp' and the 'nontrajectory' deconvolution models. The 'nontrajectory' model does not impose any trajectory structure between samples and therefore does not share any information between samples. It is therefore expected to reflect the performance of all general methods for deconvolution that do not make use of covariate information. The normalized L1 error for 10 independent deconvolution runs on the same dataset was markedly higher without trajectory estimation (Figure S14E), supporting the value of this approach.

Robustness

We extensively evaluated the robustness of ternaDecov to perturbations of the prior parameters and gene selection algorithm. For example, using an increasingly stringent parameter for the overall abundance of genes in the single-cell dataset the results remain stable well beyond the values used for the analysis (Figure S14F).

ternaDecov: Biological benchmarking and application to EBOV RNAseq data

To benchmark the method on independent biological datasets, we first used the bulk RNA-seq data from Fadista et al.[36] which contain RNA-seq data for healthy and diseased pancreatic islet samples simulated based on pancreatic islets scRNA-seq RNA-seq data from Segerstolpe et al.[37] We ran ternaDecov with HbA1C as the covariate to use for trajectory regularization. We compared cell proportions estimated by ternaDecov to those reported for MuSiC[32] and established quantitative agreement between the two methods. Moreover, ternaDecov inferred cell type composition trajectories were concordant with the results reported earlier.[98]

In order to assess blood infiltration in peripheral tissues during EBOV infection we applied ternaDecov to bulk RNAseq data with two alternative datasets as a single-cell reference; *Macaca fascicularis* single-cell atlas data,[99] and peripheral blood data from the same EBOV-infected rhesus monkeys.[18] We performed summarization of the deconvolved cell type proportions to 'Monocytes', 'non-Monocyte blood' and tissue-specific cell types. In all cases, we ran ternaDecov for 20,000 iterations for each analysis in the 'GP trajectory' mode with default settings for gene selection. Stability analysis with respect to the most salient input parameters was performed using 5,000 iterations. We validated the finding of a decrease in Chromaffin cells in adrenal tissue with MuSiC[32] run using the default parameters and identical single-cell reference.

Cell Genomics, Volume *3*

Supplemental information

Natural history of Ebola virus disease in rhesus monkeys shows viral variant emergence dynamics and tissue-specific host responses

Erica Normandin, Sergio Triana, Siddharth S. Raju, Tammy C.T. Lan, Kim Lagerborg, Melissa Rudy, Gordon C. Adams, Katherine C. DeRuff, James Logue, David Liu, Daniel Strebinger, Arya Rao, Katelyn S. Messer, Molly Sacks, Ricky D. Adams, Krisztina Janosko, Dylan Kotliar, Rickey Shah, Ian Crozier, John L. Rinn, Marta Melé, Anna N. Honko, Feng Zhang, Mehrtash Babadi, Jeremy Luban, Richard S. Bennett, Alex K. Shalek, Nikolaos Barkas, Aaron E. Lin, Lisa E. Hensley, Pardis C. Sabeti, and Katherine J. Siddle

Supplemental Figures

Supplemental figures for “Natural history of Ebola virus disease in rhesus monkeys shows viral variant emergence dynamics and tissue specific host responses” by Normandin et al.

Figures Contents

Figure S1. qPCR quantification compared to viral read counts.
Figure S2. Overview of samples profiled.
Figure S3. Host transcriptome dimensionality reduction.
Figure S4. Expression profiles during infections across tissues
Figure S5. Viral load correlates with monocyte markers.
Figure S6. Cell type deconvolution of Bulk RNA-seq.
Figure S7. Tissue specific marker genes.
Figure S8. Genes change across time.
Figure S9. ECM and Coagulation related genes change across time and tissues.
Figure S10. Reliability of minor variant calling methodology.
Figure S11. Mutation types across the sample set.
Figure S12. Mutations across animals and tissues.
Figure S13. Functional characterization of viral variants
Figure S14. Utilizing Time-series Covariate Information for Enhanced RNA-seq Deconvolution
Data S1: Pentacistronic Minigenome Assay plasmid sequences, related to STAR methods.

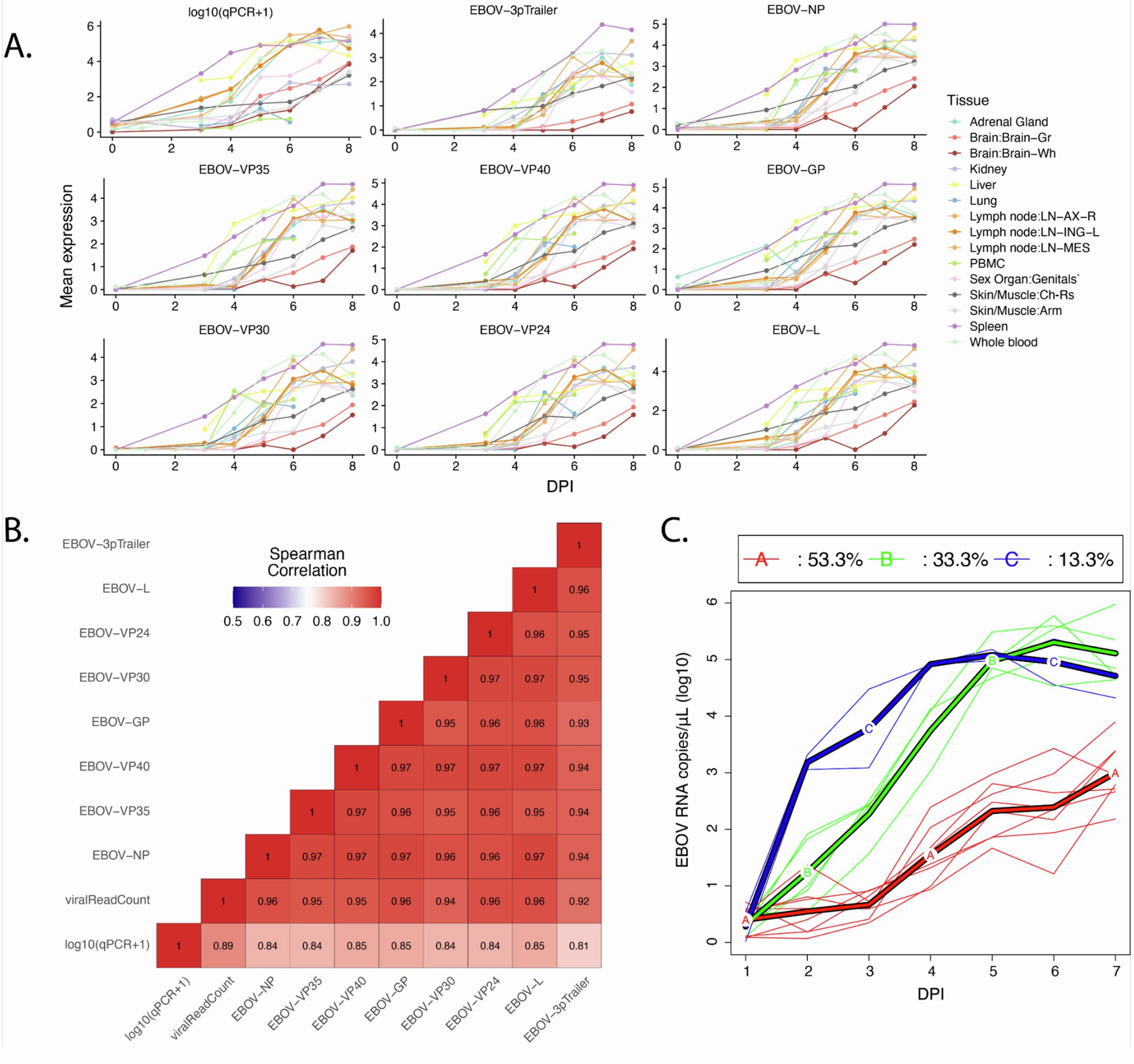

Figure S1. qPCR quantification compared to viral read counts, related to figure 1. (A) Mean expression of viral genes across tissues and DPI (B) Heatmap showing spearman correlations between each viral gene, the total viral read count and the qPCR quantification of GP RNA. (C) Longitudinal K-mean clustering of qPCR data.

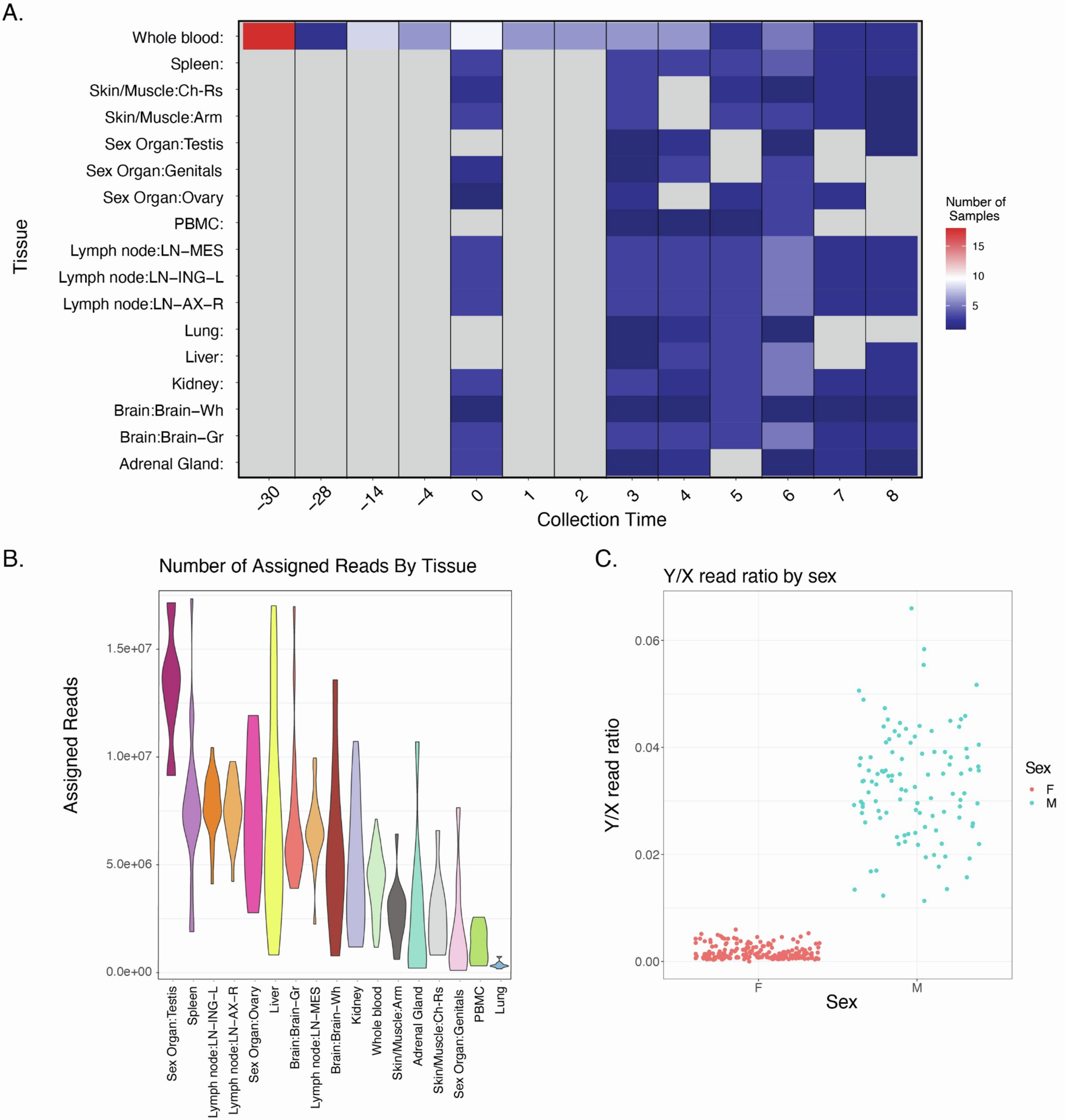

Figure S2. Overview of samples profiled, related to figure 1. (A) Sequenced sample count per tissue and collection time (B) Violin plots of total assigned reads per tissue. (C) Chromosome X and Y ratio per sample.

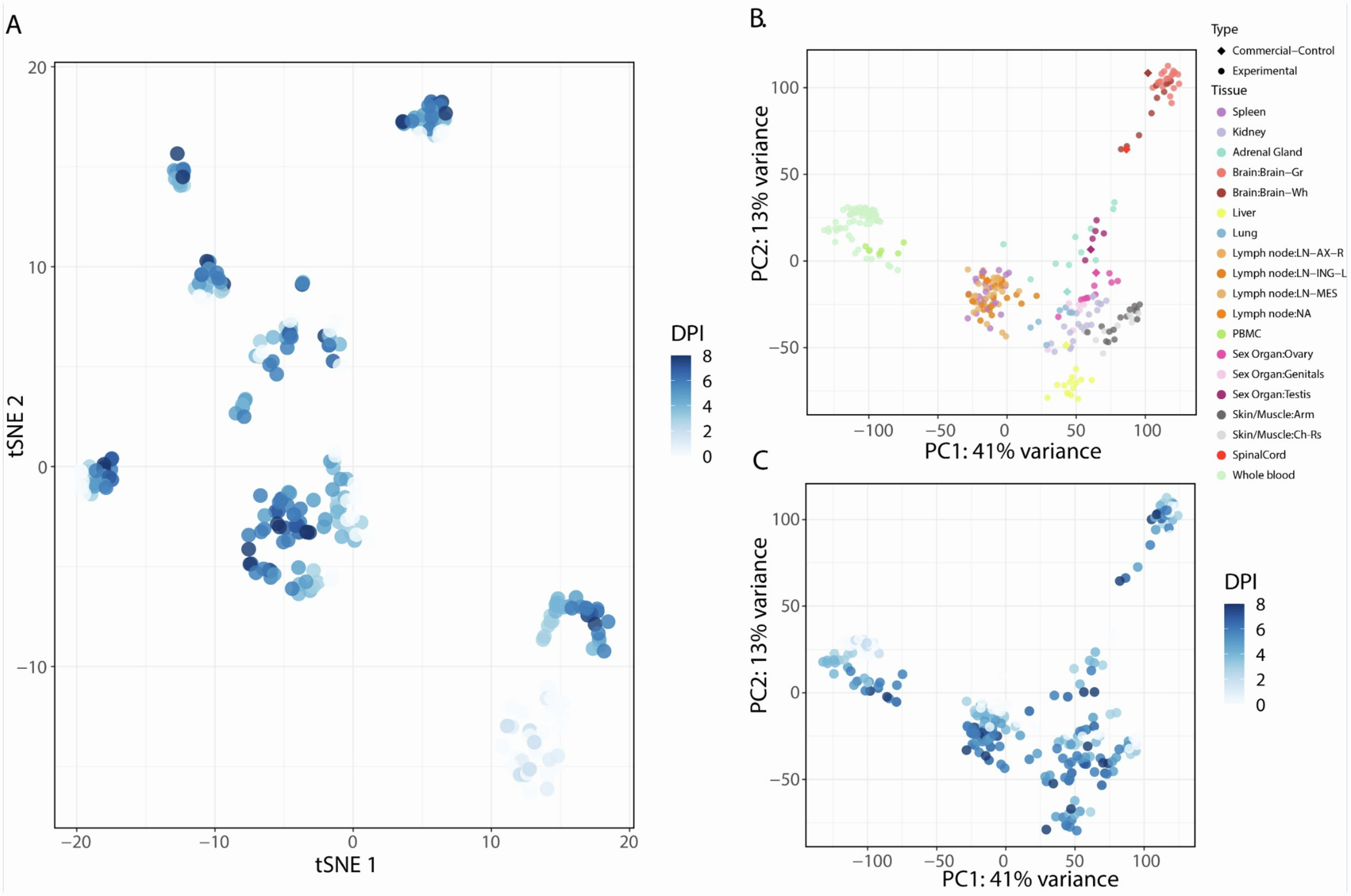

Figure S3. Host transcriptome dimensionality reduction, related to figure 1. (A) tSNE plot of transcriptional signatures colored by DPI. PCA of transcriptional profiles colored by (B) sample type and (C) DPI.

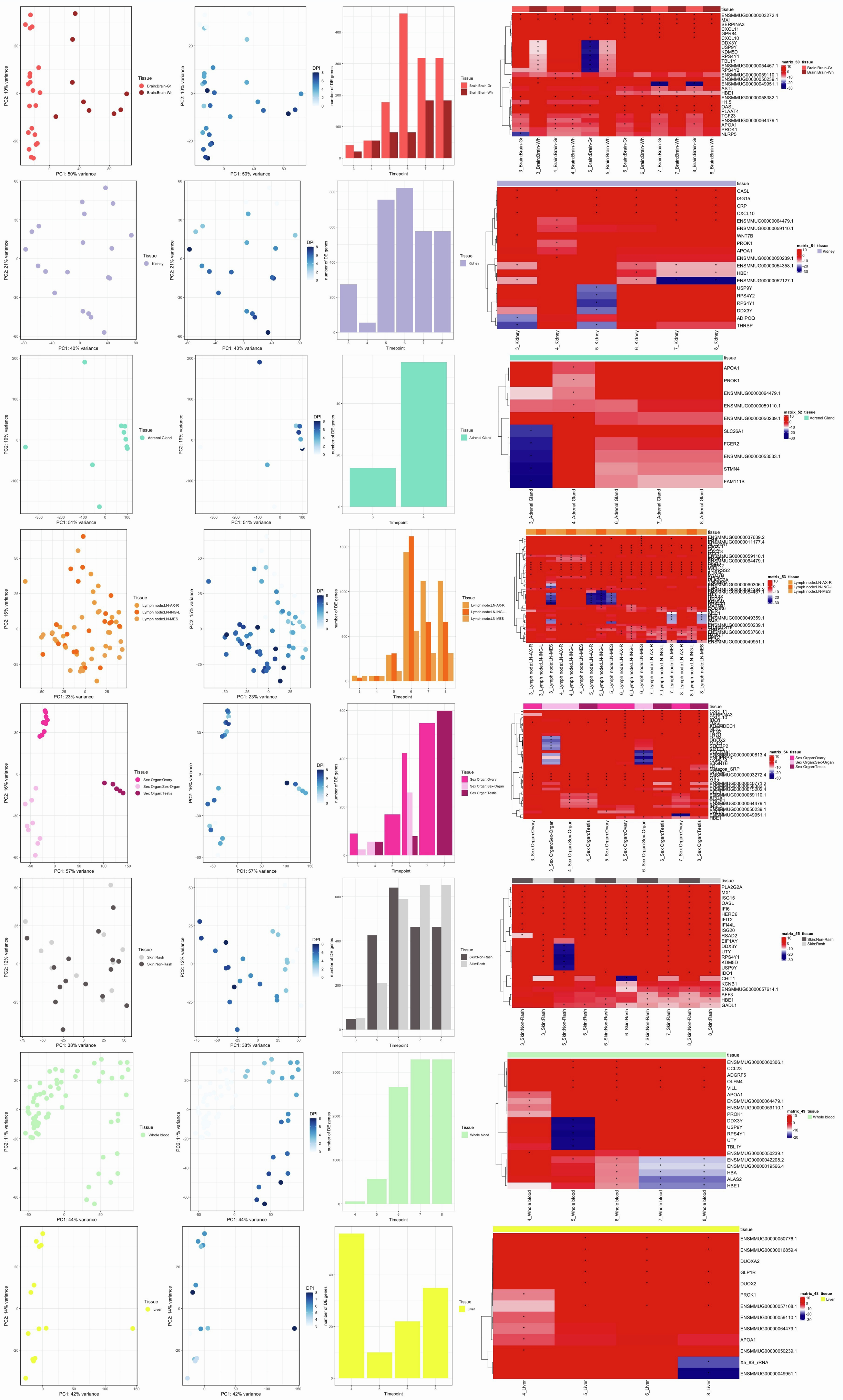

PC1: 50% variance
PC2: 10% variance
Tissue
Brain:Brain-Gr
Brain:Brain-Wh
DPI
number of DE genes
Timepoint
tissue
matrix_50
ENSMMUG00000003272.4
MX1
SERPINA3
CXCL11
GPR84
CXCL10
DDX3Y
USP9Y
KDM5D
RPS4Y1
TBL1Y
ENSMMUG00000054467.1
RPS4Y2
ENSMMUG00000059110.1
ENSMMUG00000050239.1
ENSMMUG00000049951.1
ASTL
HBE1
ENSMMUG00000058382.1
H1.5
OASL
PLAAT4
TCF23
ENSMMUG00000064479.1
APOA1
PROK1
NLRP5
PC1: 40% variance
PC2: 21% variance
Kidney
matrix_51
OASL
ISG15
CRP
CXCL10
ENSMMUG00000064479.1
ENSMMUG00000059110.1
WNT7B
PROK1
APOA1
ENSMMUG00000050239.1
ENSMMUG00000054358.1
HBE1
ENSMMUG00000052127.1
USP9Y
RPS4Y2
RPS4Y1
DDX3Y
ADIPOQ
THRSP
PC1: 51% variance
PC2: 19% variance
Adrenal Gland
matrix_52
APOA1
PROK1
ENSMMUG00000064479.1
ENSMMUG00000059110.1
ENSMMUG00000050239.1
SLC26A1
FCER2
ENSMMUG00000053533.1
STMN4
FAM111B
PC1: 23% variance
PC2: 15% variance
Lymph node:LN-AX-R
Lymph node:LN-ING-L
Lymph node:LN-MES
matrix_53
PC1: 57% variance
PC2: 16% variance
Sex Organ:Ovary
Sex Organ:Sex-Organ
Sex Organ:Testis
matrix_54
PC1: 38% variance
PC2: 12% variance
Skin:Rash
Skin:Non-Rash
matrix_55
PLA2G2A
MX1
ISG15
OASL
IFI6
HERC6
IFIT2
IFI44L
ISG20
RSAD2
EIF1AY
DDX3Y
UTY
RPS4Y1
KDM5D
USP9Y
IDO1
CHIT1
KCNB1
ENSMMUG00000057614.1
AFF3
HBE1
GADL1
PC1: 44% variance
PC2: 11% variance
Whole blood
matrix_49
ENSMMUG00000060306.1
CCL23
ADGRF5
OLFM4
VILL
APOA1
ENSMMUG00000064479.1
ENSMMUG00000059110.1
PROK1
DDX3Y
USP9Y
RPS4Y1
UTY
TBL1Y
ENSMMUG00000050239.1
ENSMMUG00000042208.2
ENSMMUG00000019566.4
HBA
ALAS2
HBE1
PC1: 42% variance
PC2: 14% variance
Liver
matrix_48
ENSMMUG00000050776.1
ENSMMUG00000016859.4
DUOXA2
GLP1R
DUOX2
PROK1
ENSMMUG00000057168.1
ENSMMUG00000059110.1
ENSMMUG00000064479.1
APOA1
ENSMMUG00000050239.1
X5_8S_rRNA
ENSMMUG00000049951.1

Figure S4. Expression profiles during infections across tissues related to figure 3. Right, PCA of transcriptional profiles of each tissue colored by sample type and DPI. Center, Number of differentially expressed genes between non-infected and each time point. Heatmap of Fold-changes of Top DE genes in each time point and tissue

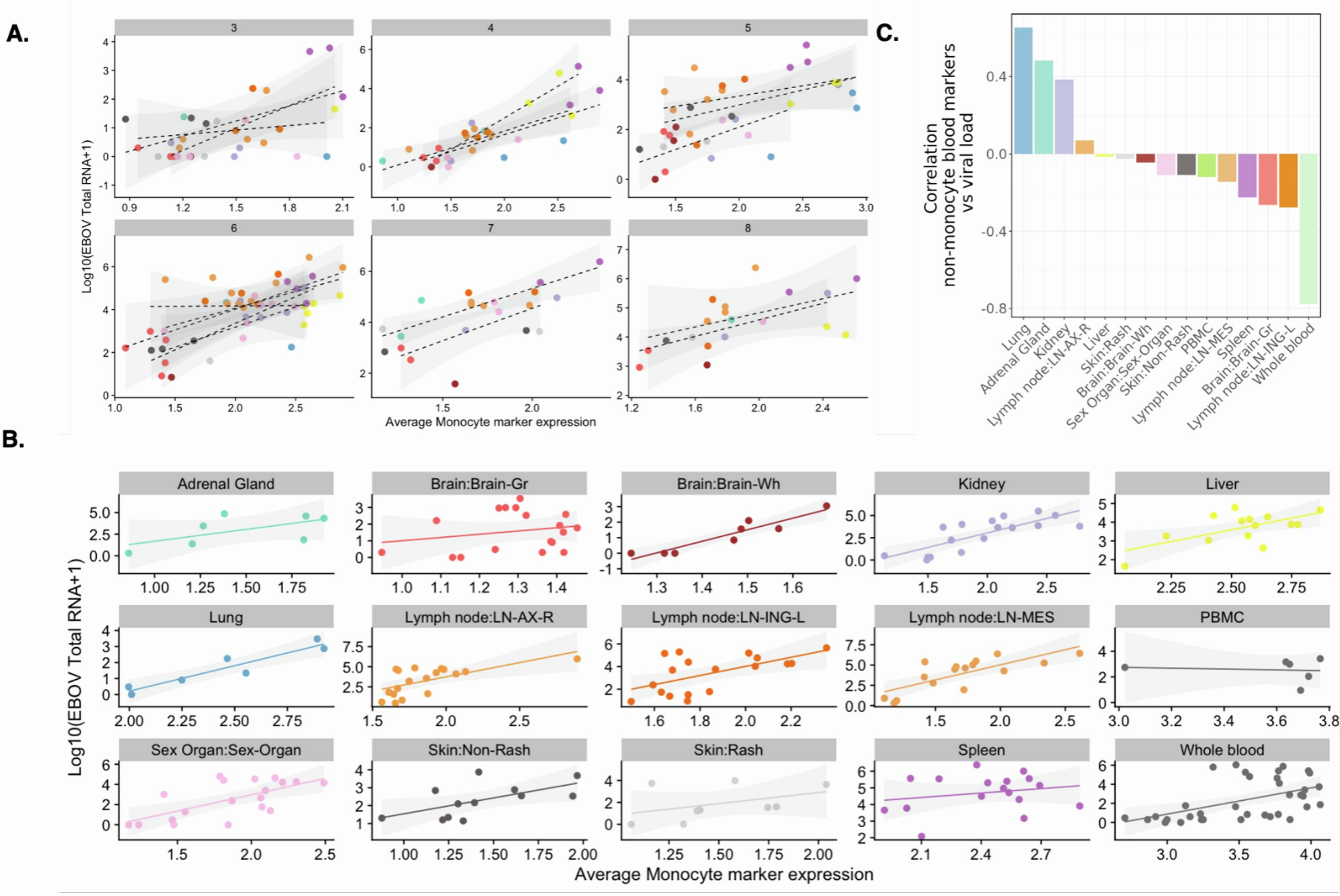

Figure S5. Viral load correlates with monocyte markers, related to figure 2. (A) Total viral counts compare to the average expression of canonical monocyte markers (*CTSS, VCAN, FCN1, CD14, S100A9*) across each time by individual (A) and by tissue (B). (C) Correlation between viral load and non-monocyte blood markers (*CD3D,HBA, SELL, PPBP, HBA,CD8A, GNLY, CD4*) expression across each tissue.

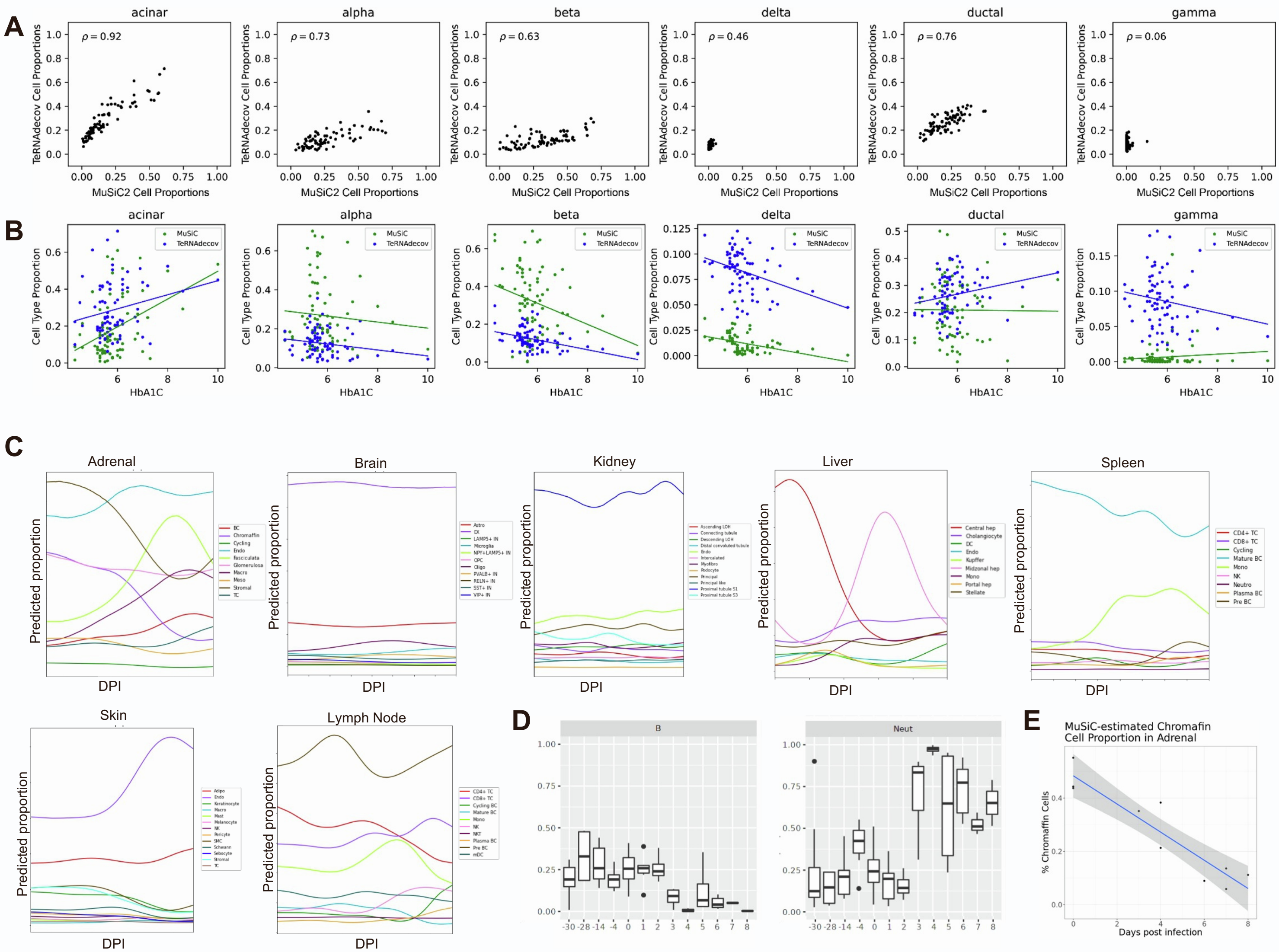

Figure S6. Cell type deconvolution of Bulk RNA-seq, related to figure 2. A) Predicted cell type proportion for pancreatic islet bulk RNA-seq data from Fadista et al using ternaDecov and MuSiC2. B) Correlation of HbA1c level and cell-type composition C) Deconvolution of predicted cell types changes across time for each tissue based on a single-cell RNAseq reference of *Macaca fascicularis*. (D) Deconvolution of Whole Blood with MuSiC confirms Neutrophil peak at 4 DPI. (E) Deconvolution of adrenal tissues using MuSiC confirms the reduction of the relative proportion of Chromaffin cells during infection.

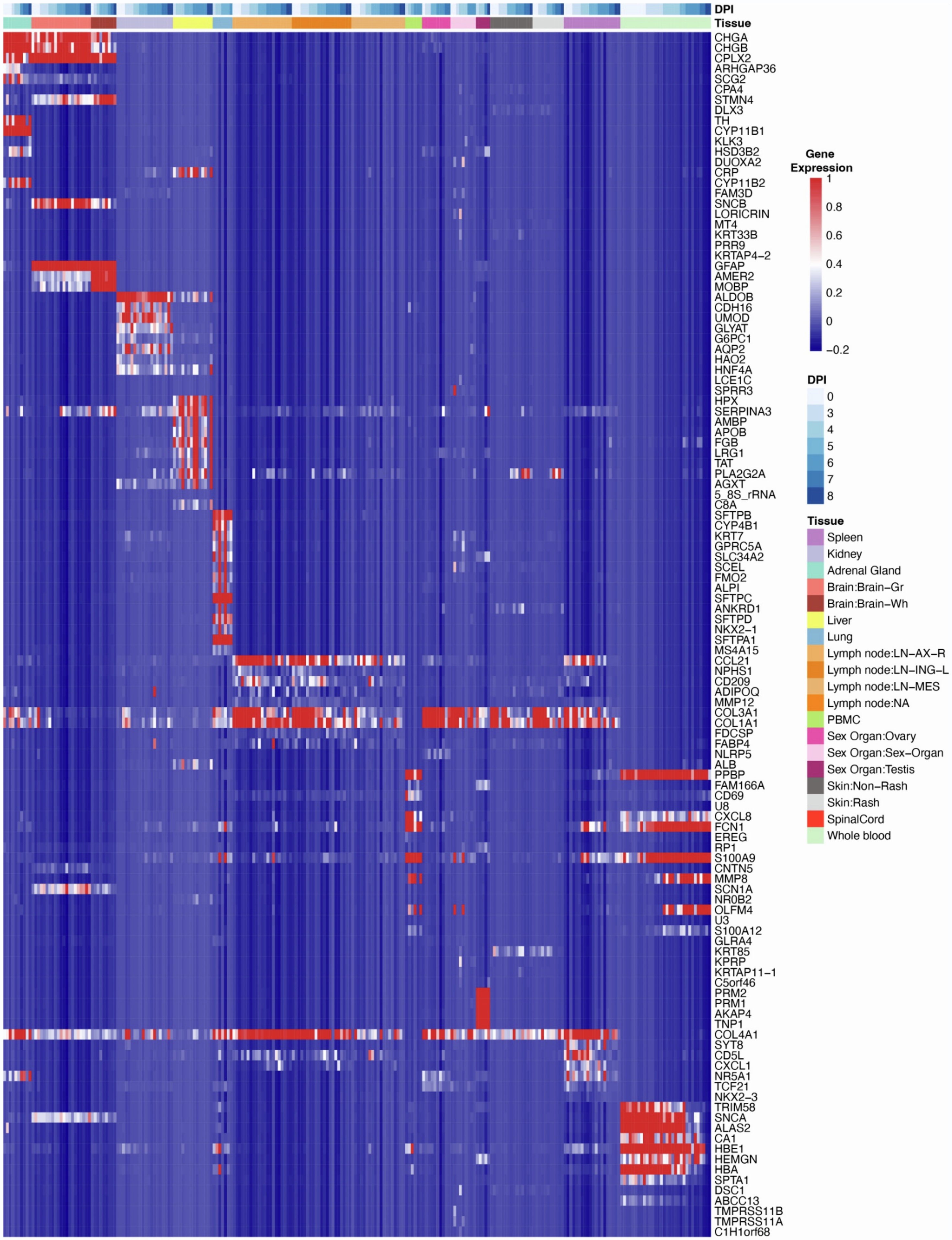

Figure S7. Tissue specific marker genes, related to figure 3. Heatmap of top specific tissue marker genes across time points.

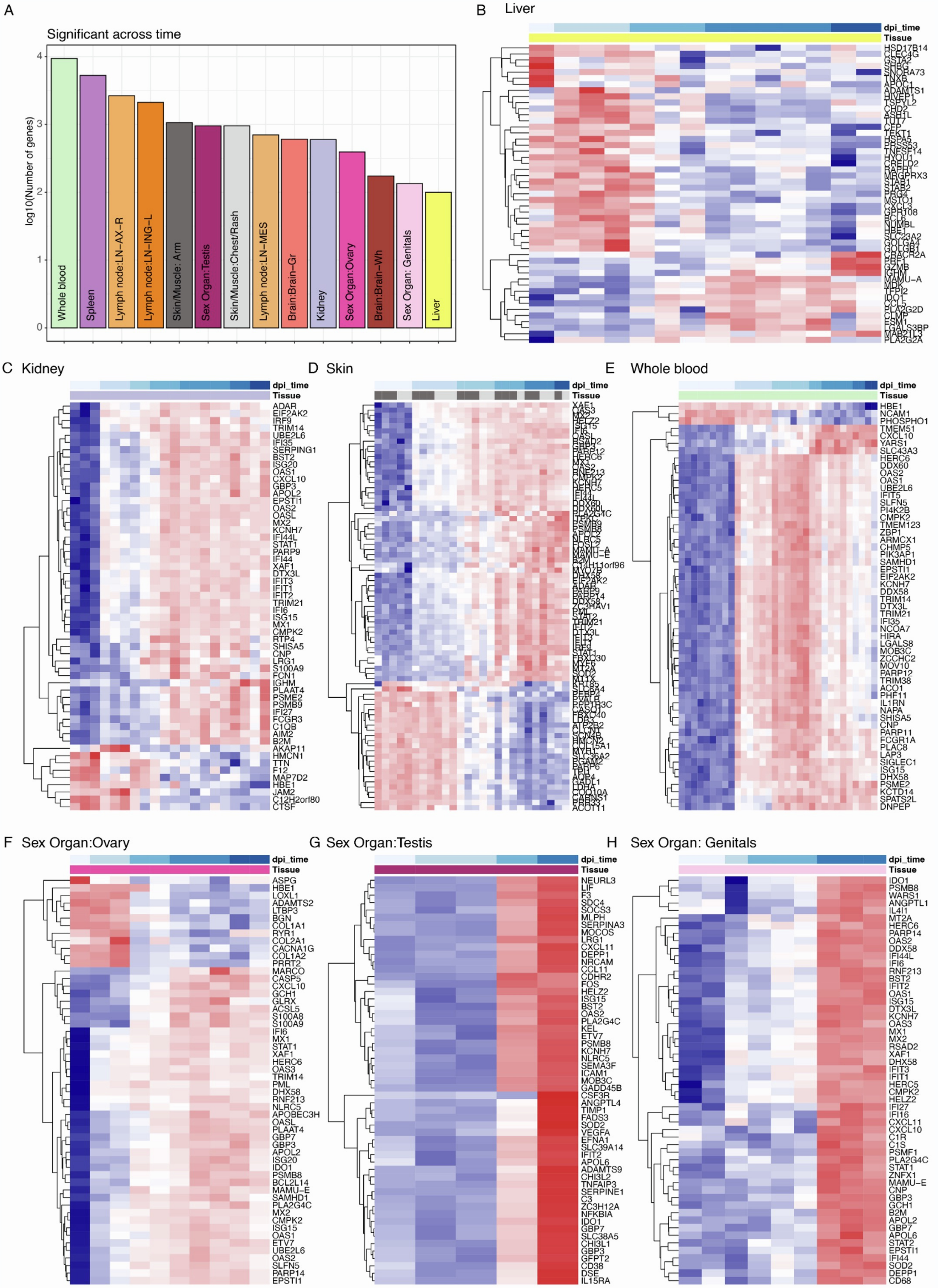

Figure S8. Genes change across time, related to figure 3. (A) Number of differentially expressed genes across time, tissues with more than 5 DE genes are shown in the plot. Heatmap of genes changing significantly across time for (B) Liver (C) Kidney (D) Skin (E) Whole Blood (F) Ovary (G) Testis and (H) Genitals

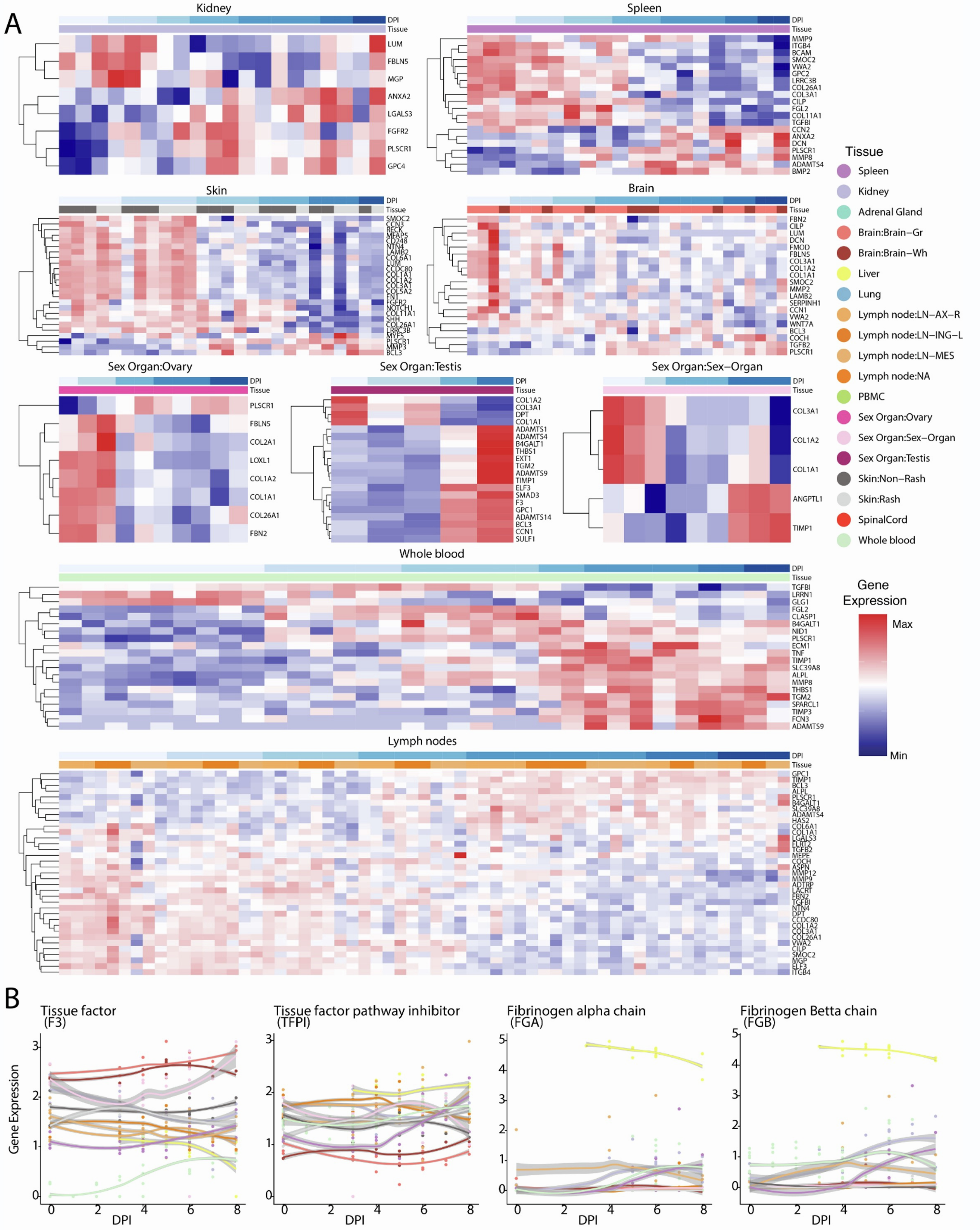

Figure S9. ECM and Coagulation related genes change across time and tissues, related to figure 3. (A) Heatmap of ECM genes that significantly change across time for each tissue (ImpulseDE2 Adjusted P-Value < 0.05). (B) Selected coagulation related genes expression.

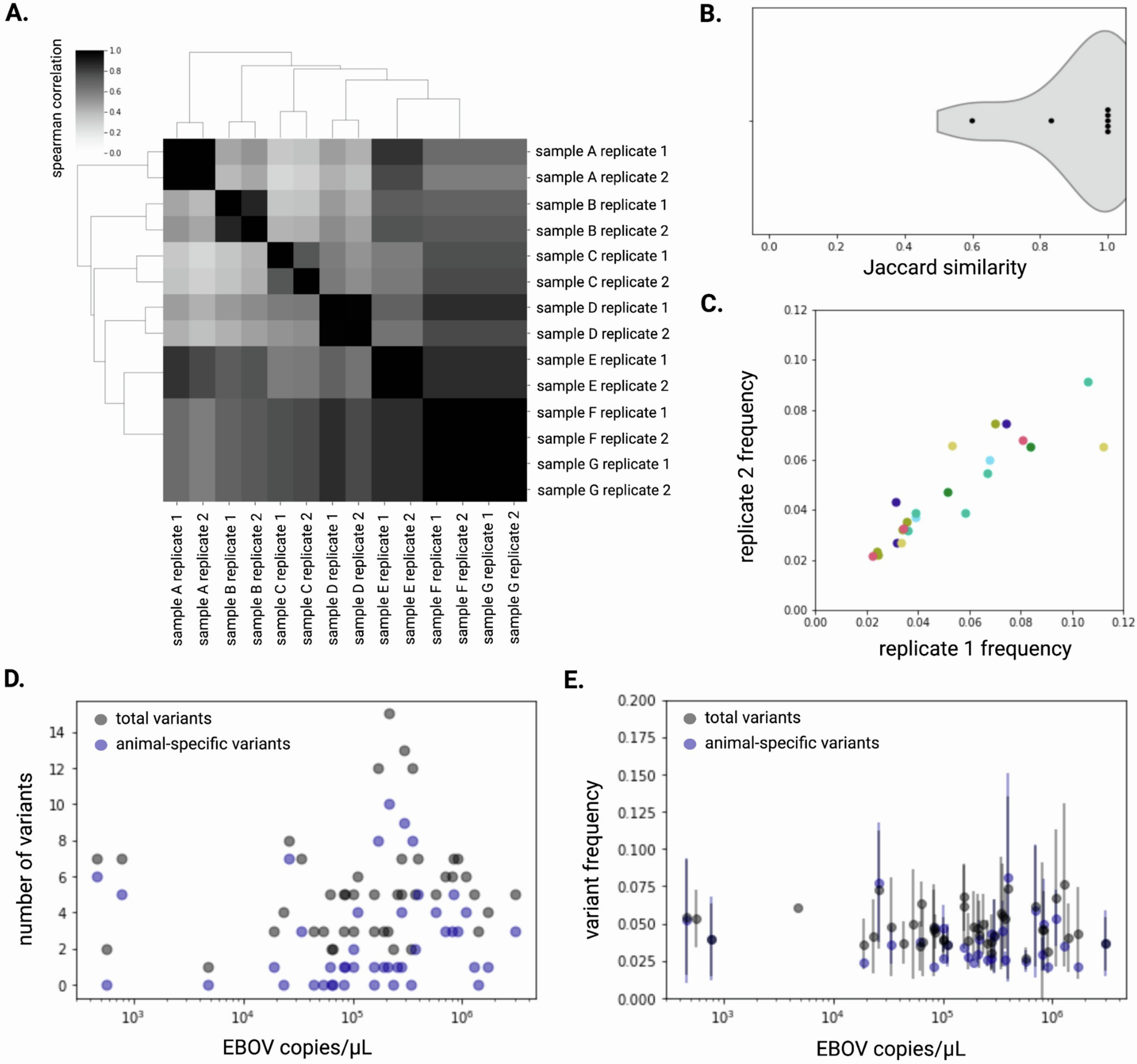

Figure S10. Reliability of minor variant calling methodology, related to figure 4. (A) Spearman correlation of variant profiles of 7 select samples with duplicate sequencing. (B) Violin plot of Jaccard similarities of variants identified in duplicate libraries from 7 samples. (C) For variants identified in two replicates, comparison of the frequency at which the variant was identified in each replicate. Each of the 7 samples is represented by a different color. (D) Total (black) and animal-specific (blue) variants identified versus viral load in each sample profiled. (E) Mean variant frequency versus viral load in each sample profiled. Error bars represent standard deviation of variant frequency.

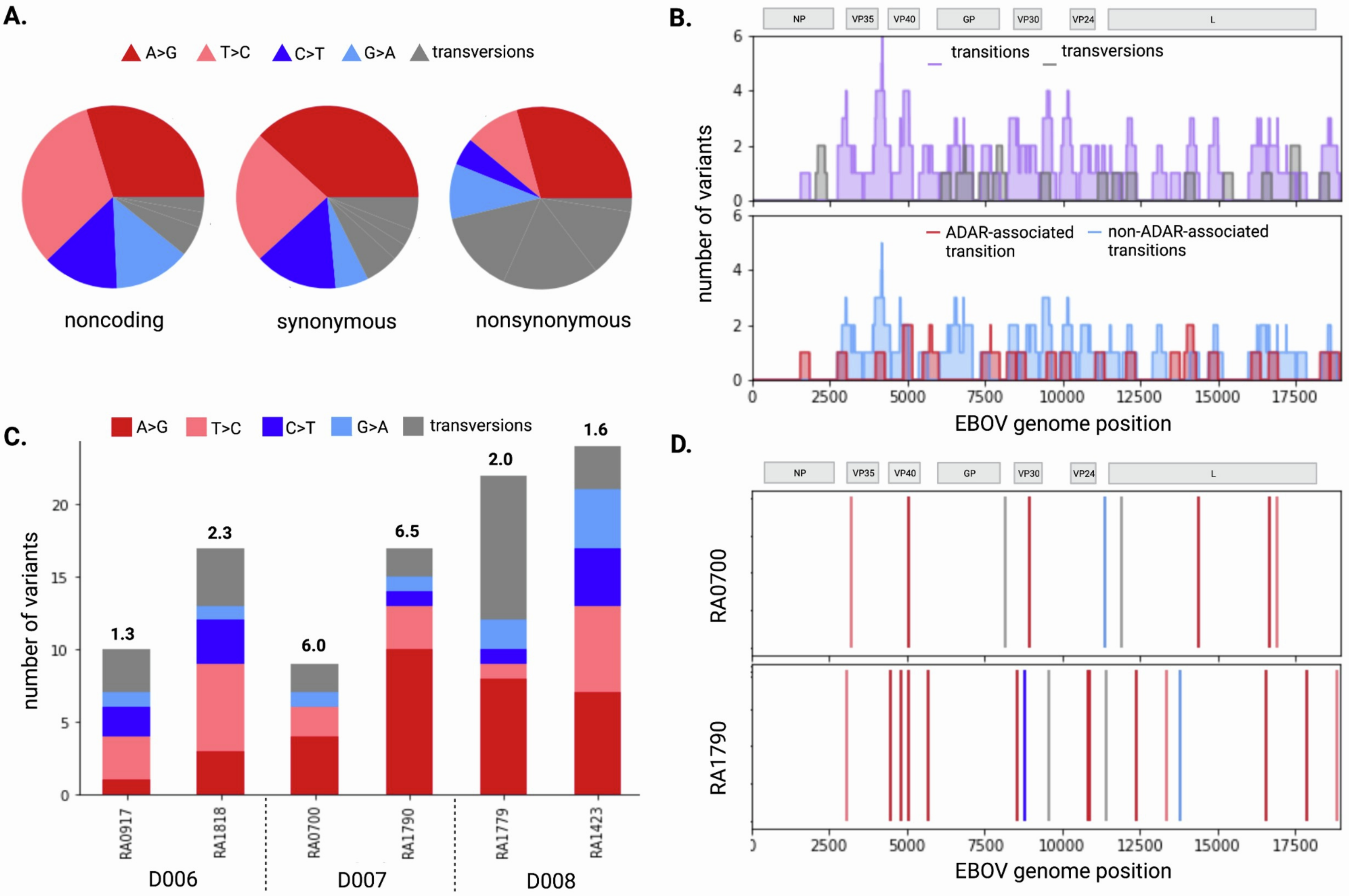

Figure S11. Mutation types across the sample set, related to figure 4. (A) Pie charts show relative amounts of each type of transition (labeled by color) and transversions (gray), separated by the mutation type. (B) Number of mutations that are transitions (purple) or transversions (gray) quantified by a 300 base pair sliding window across the EBOV genome. An EBOV gene map is above. (C) Stacked bar plots show the number of mutations that were each type of transition (labeled by color) and transversions (gray), separated by animal, ordered by DPI cohort. The bold number above shows the ratio of A-to-G and T-to-C mutations to C-to-T and G-to-A mutations, a marker of host RNA editing enzyme activity. (D) For two animals with the highest ratio of host RNA editing-associated mutations (RA0700 and RA1790), each mutation is represented by a vertical line along the EBOV genome on the x-axis. An EBOV gene map is above.

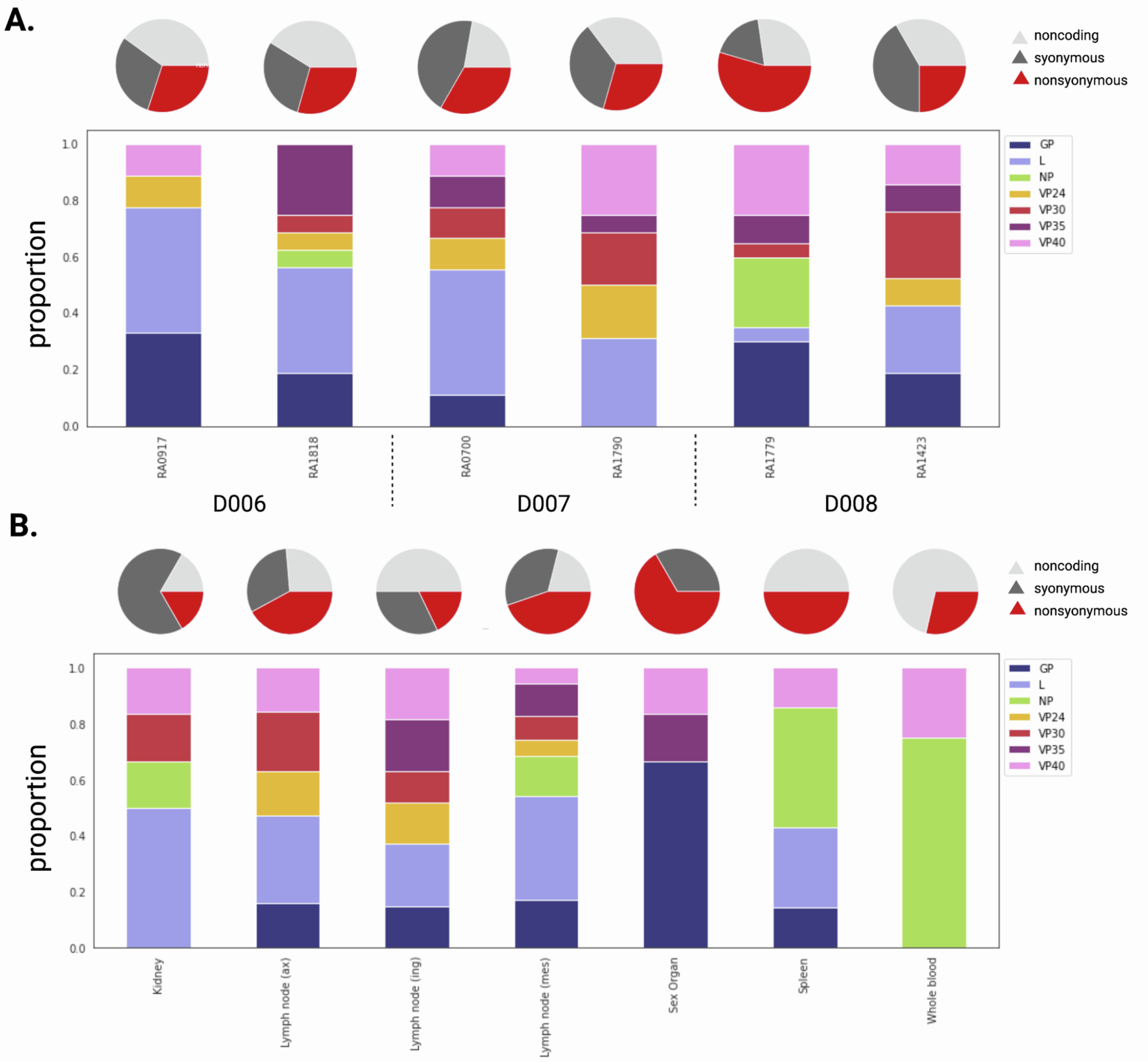

Figure S12. Mutations across animals and tissues, related to figure 4. (A) Proportion of nonsynonymous (red), synonymous (dark gray), and noncoding (light gray) variants across animals, ordered by cohort (top). Proportion of variants falling into each EBOV gene in each animal, ordered by cohort (bottom). (B) Proportion of nonsynonymous (red), synonymous (dark gray), and noncoding (light gray) variants within each tissue (top). Proportion of variants falling into each EBOV gene within each tissue (bottom).

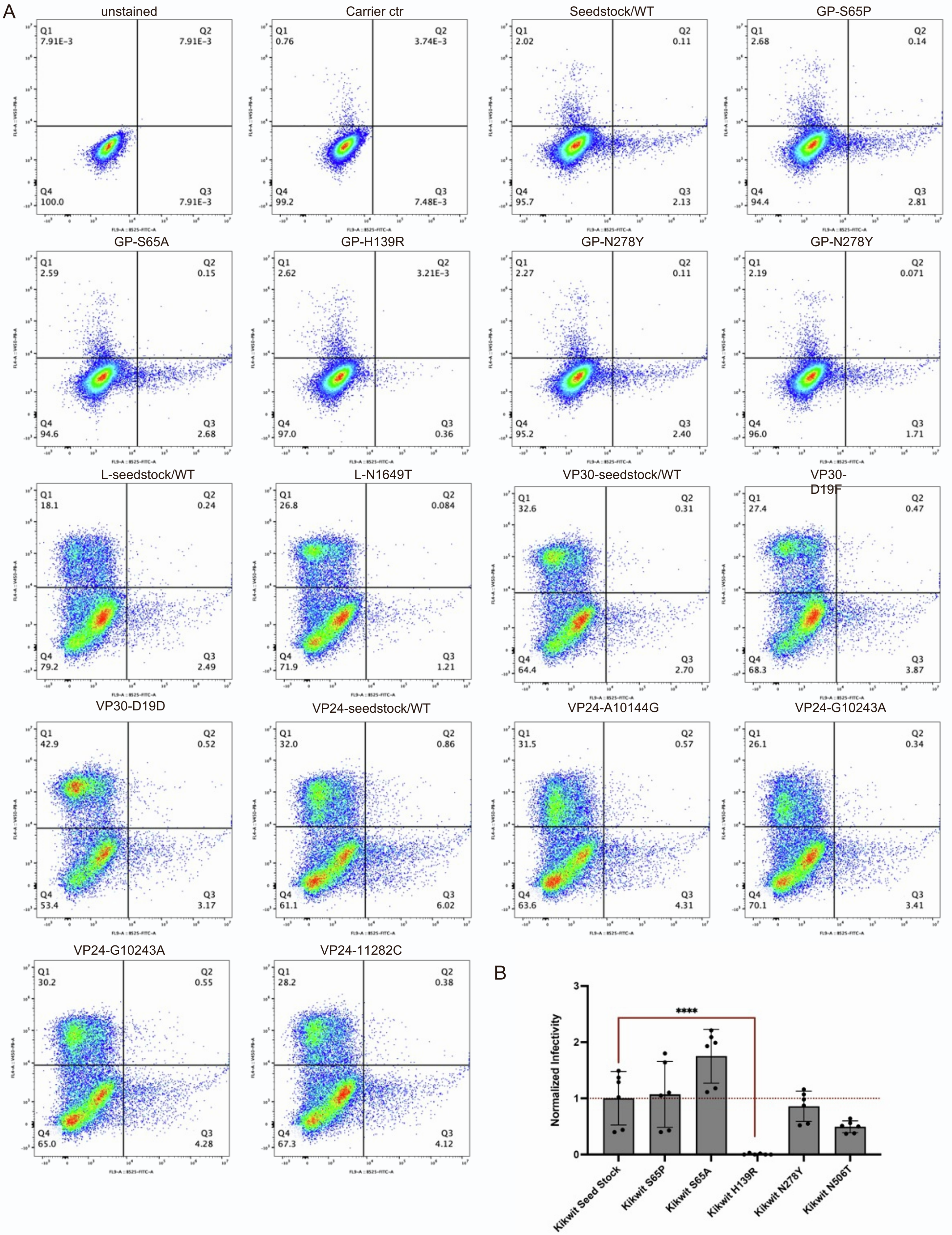

Figure S13. Functional characterization of viral variants, related to figure 5 (A) Gating strategy for GFP+ cells EBOV minigenome expression in HEK293T cells, representative data for n=2 independent biological replicates. (B) Quantification of fold difference in mCherry mRNA expression in HEK293T cells transduced with lentiviral virions pseudotyped with EBOV GP bearing the viral seed stock sequence or variants. Error bars represents standard deviation.

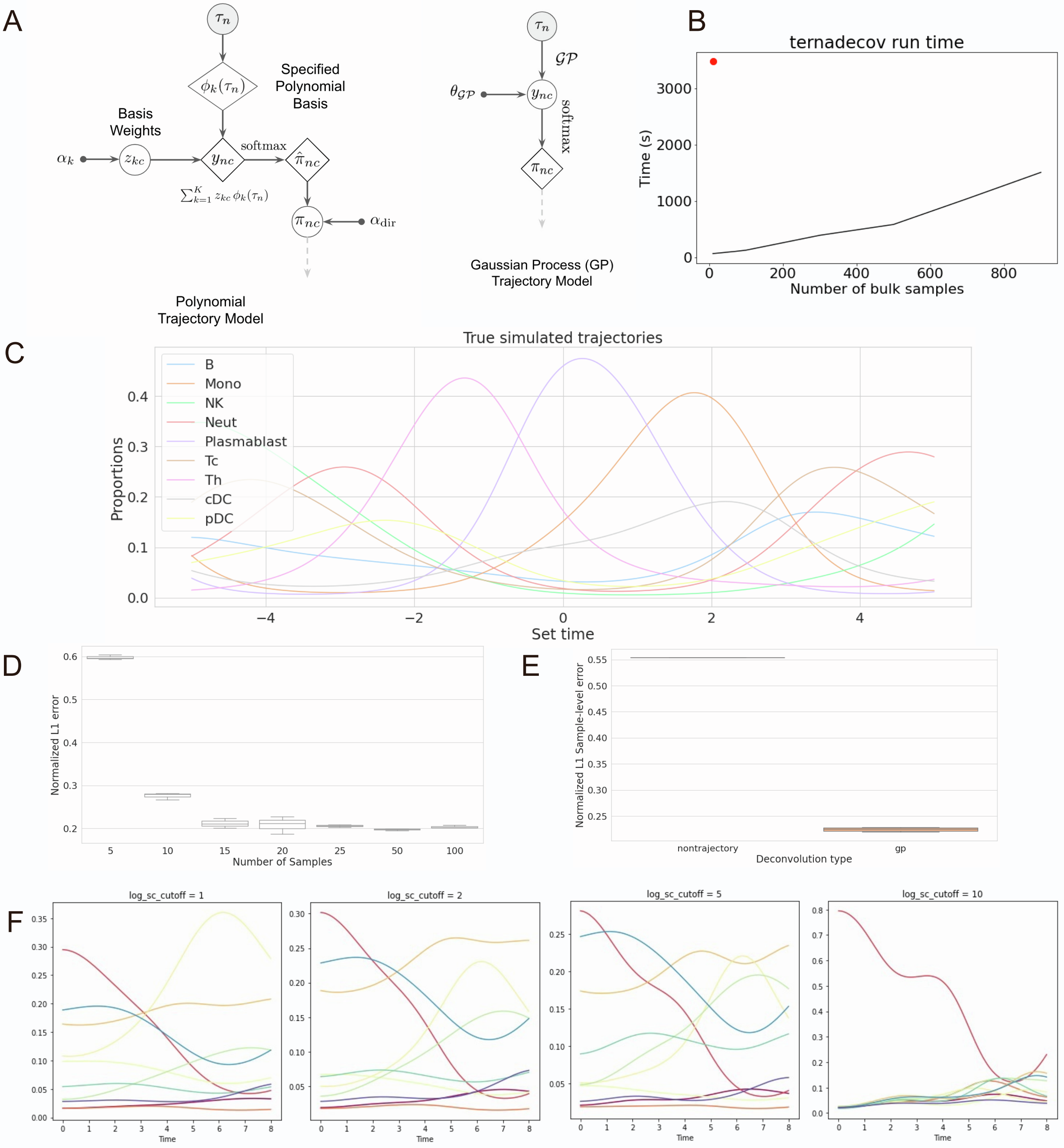

Figure S14. Utilizing Time-series Covariate Information for Enhanced RNA-seq Deconvolution, related to STAR methods (A) The parametric polynomial basis trajectory model (left), and the non-parametric Gaussian process trajectory model (right). The models are drop-in replacements as sub-models inside ternaDecov (see Figure 2D) (B) Execution time of ternaDecov (5,000 iterations, no GPU acceleration) for varying number of simulated samples using the GP trajectory module. The red point indicates the time required for deconvolution of the 10 samples from adrenal glands using MuSiC, shown for validation in the main text (C) Random simulated trajectory used for assessment of improvement in trajectory estimation as the number of sampled points increases (D) Normalized L1 trajectory error for trajectory estimation (at 1000 fixed points) using GP method from variable number of samples (3 replicates per point). Trajectory estimation improves as the number of samples increases (E) Normalized L1 sample-level error for the GP and non-trajectory (naive) model indicates that imposition of trajectory improves our ability to deconvolve the sample composition (F) Deconvolution results for adrenal tissues with increasing values of the log_sc_cutoff parameter (abundance cutoff for gene selection of the single-cell data) indicates robustness to parameter values.

Data S1: Pentacistronic Minigenome Assay plasmid sequences, related to STAR methods.

1. EBOV/Kikwit 5MG

CGGACACACAAAAAGAAAGAAGAATTTTTAGGATCTTTTGTGTGCGAATAACTATGAGGAAGATTAATAATTTTCC
TCTCATTGAAATTTATATCGGAATTTAAATTGAAATTGTTACTGTAATCACACCTGGTTTGTTTCAGAGCCACATCA
CAAAGATAGAGAACAACCTAGGTCTCTGAAGGGAGCAAGGGCATCAGTGTGCTCAGTTGAAAATCCCTTGTCAA
CATCTAGGTCTTATCACATCACAAGTCCCACCTCAGACTCTGCAGGGTGATCCAACAACCTTAATAGAAACATTAT
TGTTAAAGGACAGCATTAGTTCACAGTCAAACAAGCAAGATTGAGAATTAACCTTGGTTTTGAACTTGAATACTTA
GAGGATTGGAGATTCAACAACCCTAAAGCTTGGGGTAAAACATTGGAAATAGTTAAAAGACAAATTGCTCGGAAT
CACAACATTCCGAGTATGGTGAGCAAGGGCGAGGAGCTGTTCACCGGGGTGGTGCCCATCCTGGTCGAGCTGG
ACGGCGACGTAAACGGCCACAAGTTCAGCGTGTCCGGCGAGGGCGAGGGCGATGCCACCTACGGCAAGCTGA
CCCTGAAGTTCATCTGCACCACCGGCAAGCTGCCCGTGCCCTGGCCCACCCTCGTGACCACCTTGACCTACGG
CGTGCAGTGCTTCGCCCGCTACCCCGACCACATGAAGCAGCACGACTTCTTCAAGTCCGCCATGCCCGAAGGC
TACGTCCAGGAGCGCACCATCTTCTTCAAGGACGACGGCAACTACAAGACCCGCGCCGAGGTGAAGTTCGAGG
GCGACACCCTGGTGAACCGCATCGAGCTGAAGGGCATCGACTTCAAGGAGGACGGCAACATCCTGGGGCACAA
GCTGGAGTACAACTACAACAGCCACAAGGTCTATATCACCGCCGACAAGCAGAAGAACGGCATCAAGGTGAACT
TCAAGACCCGCCACAACATCGAGGACGGCAGCGTGCAGCTCGCCGACCACTACCAGCAGAACACCCCCATCGG
CGACGGCCCCGTGCTGCTGCCCGACAACCACTACCTGAGCACCCAGTCCGCCCTGAGCAAAGACCCCAACGAG
AAGCGCGATCACATGGTCCTGCTGGAGTTCGTGACCGCCGCCGGGATCACTCTCGGCATGGACGAGCTGTACA
AGTAAATGAGCATGGAACAATGGGATGATTCAACCGACAAATAGCTAACATTAGGTAGTCAAGGAACGAAAACAG
GAAGAATTTTTGATGTCTAAGGTGTGAATTATTATCACAATAAAAGTGATTCTTATTTTTGAATTTAAAGCTAGCTT
ATTATTACTAGCCGTTTTTCAAAGTCCAATTTGAGTCTTAATGCAAATAGGCGTTAAGCCACAGTTATAGCCATAA
TTGTAACTCAATATCCTAACTAGCGATTTATCTAAATTAAATTACATTATGCTTTTATAACTTACCTACTAGCCTACC
CAACATTTACACGATCATTTTATAATTAAGAAAAAACTAATGATGAAGATTAAAACCTTCATCATCCTTACGTCAAT
TGAATTCTCTAGCACTCGAAGCTTATTGTCCTCAATGTAAAAGAAAAGCTGGTCTAACAAGATGGTCTTCACACTC
GAAGATTTCGTTGGGGACTGGCGACAGACAGCCGGCTACAACCTGGACCAAGTCCTTGAACAGGGAGGTGTGT
CCAGTTTGTTTCAGAATCTCGGGGTGTCCGTAACTCCGATCCAAAGGATTGTCCTGAGCGGTGAAAATGGGCTG
AAGATCGACATCCATGTCATCATCCCGTATGAAGGTCTGAGCGGCGACCAAATGGGCCAGATCGAAAAAATTTTT
AAGGTGGTGTACCCTGTGGATGATCATCACTTTAAGGTGATCCTGCACTATGGCACACTGGTAATCGACGGGGT
TACGCCGAACATGATCGACTATTTCGGACGGCCGTATGAAGGCATCGCCGTGTTCGACGGCAAAAAGATCACTG
TAACAGGGACCCTGTGGAACGGCAACAAAATTATCGACGAGCGCCTGATCAACCCCGACGGCTCCCTGCTGTTC
CGAGTAACCATCAACGGAGTGACCGGCTGGCGGCTGTGCGAACGCATTCTGGCGTAAGCCCATCTTCCTTCCCT
CCGAAAGAGGCGACTAATAGCAGAGGCTTCAACTGCTGAACTATAGGGTACGTTACATTAATGATACACTTGTGA
GTATCAGCCCTGGATAATATAAGTCAATTAAACGACCAAGATAAAATTGTTCTTATCTCGCTAGCAGCTTAAAATA
TGAATGTAATAGGAGCTATATCTCTGACAGTATTATAATCAATCGTTATTAAGTAACCCAAACCAAAAGTGATGAA
GATTAAGAAAAACCTACCTCGACTGAGAGAGTGTTTTTTCATTAACCTTCATCTTGTAAACGTTGAGCAAAATTGT
TAAAAATATGAGGCGGGTTATATTACCTACTGCTCCTCCTGAATATATGGAGGCCATATACCCTGTCAGGTCAAA
TTCAACAATTGCTAGAGGTGGCAACAGCAATACAGGCTTCCTGACACCGGAGTCAGTCAATGGGGACACTCCAT
CGAATCCACTCAGGCCAATTGCCGATGACACCATCGACCATGCCAGCCACACACCAGGCAGTGTGTCATCAGCA
TTCATCCTTGAAGCTATGGTGAATGTCATATCGGGCCCCAAAGTGCTAATGAAGCAAATTCCAATTTGGCTTCCT
CTAGGTGTCGCTGATCAAAAGACCTACAGCTTTGACTCAACAACGGCCGCCATCATGCTTGCTTCATATACTATC
ACCCATTTCGGCAAGGCAACCAATCCACTTGTCAGAGTCAATCGGCTGGGTCCTGGAATCCCGGATCACCCCCT
CAGGCTCCTGCGAATTGGAAACCAGGCCTTCCTCCAGGAGTTCGTTCTTCCGCCAGTCCAACTACCCCAGTATT
TCACCTTTGATTTGACAGCACTCAAACTGATCACCCAACCACTGCCTGCTGCAACATGGACCGATGACACTCCAA
CAGGATCAAATGGAGCGTTGCGCCCAGGGATTTCATTTCATCCAAAACTTCGCCCCATTCTTTTACCCAACAAGA
GTGGGAAGAAGGGGAATAGTGCCGATCTAACATCTCCGGAGAAAATCCAAGCAATAATGACTTCACTCCAGGAC
TTTAAGATCGTTCCAATTGATCCAACCAAAAATATCATGGGAATCGAAGTGCCAGAAACTCTGGTCCACAAGCTG
ACCGGTAAGAAGGTGACTTCTAAAAATGGACAACCAATCATCCCTGTTCTTTTGCCAAAGTACATTGGTTTGGAC
CCGGTGGCTCCAGGAGACCTCACCATGGTAATCACACAGGATTGTGACACGTGTCATTCTCCTGCGAGTCTTCC
AGCTGTGATTGAGAAGTAATTGCAATAATTGACTCAGATCCAGTTTTACAGAATCTTCTCAGGGATAGTGATAACA
TCTATTTAGTAATCCGTCCATTAGAGGAGATACTTTTAATTGATCAATATACTAAAGGTGCTTTACACCATTGTCTT
TTTCCTCTCCTAAATGTAGAACTTAACAAAAGACTCATAATATACTTGTTTTTAAAAGATTGATTGATGAAAGATCA

TAACTAATAACATTACAAATAATCCTACTATAATCAATACGGTGATCCAAATGTTAATCTTTCTCATTGCAAATACT
CTTTGCCCTTATCCTCAAATTGCCTACATGCTTACATCTGAGGATAGCCAGTGTGACTTGGATTGGAGATGTGGA
GAAAAAATCGGGACCCATTTCTAGGTTGTTCACCATCCAAGTACAGACATTGCCCTTCTAATTAAGAAAAAATCG
GCGATGAAGATTAAGCCGACAGTGAGCGTAATCTTCATCTCTCTTAGATTATTTGTCCTCCAGAGTAGGGATCGT
CAGGTCCTTTTCAATCGTATAACCAAAATAAACTTCACTAGAAGGATATTGTGGGGCAACAACACAATGGGTGTT
ACAGGAATATTGCAGTTACCTCGTGATCGATTCAAGAGGACATCATTCTTTCTTTGGGTAATTATCCTTTTCCAAA
GAACATTTTCCATCCCACTTGGAGTCATCCACAATAGCACATTACAGGTTAGTGATGTCGACAAACTGGTTTGCC
GTGACAAACTGTCATCCACAAATCAATTGAGATCAGTTGGACTGAATCTCGAAGGGAATGGAGTGGCAACTGAC
GTGCCATCTGCAACTAAAAGATGGGGCTTCAGGTCCGGTGTCCCACCAAAGGTGGTCAATTATGAAGCTGGTGA
ATGGGCTGAAAACTGCTACAATCTTGAAATCAAAAAACCTGACGGGAGTGAGTGTCTACCAGCAGCGCCAGACG
GGATTCGGGGCTTCCCCCGGTGCCGGTATGTGCACAAAGTATCAGGAACGGGACCGTGTGCCGGAGACTTTGC
CTTCCACAAAGAGGGTGCTTTCTTCCTGTATGACCGACTTGCTTCCACAGTTATCTACCGAGGAACGACTTTCGC
TGAAGGTGTCGTTGCATTTCTGATACTGCCCCAAGCTAAGAAGGACTTCTTCAGCTCACACCCCTTGAGAGAGC
CGGTCAATGCAACGGAGGACCCGTCTAGTGGCTACTATTCTACCACAATTAGATATCAAGCTACCGGTTTTGGAA
CCAATGAGACAGAGTATTTGTTCGAGGTTGACAATTTGACCTACGTCCAACTTGAATCAAGATTCACACCACAGT
TTCTGCTCCAGCTGAATGAGACAATATATACAAGTGGGAAAAGGAGCAATACCACGGGAAAACTAATTTGGAAGG
TCAACCCCGAAATTGATACAACAATCGGGGAGTGGGCCTTCTGGGAAACTAAAAAAAACCTCACTAGAAAAATTC
GCAGTGAAGAGTTGTCTTTCACAGCTGTATCAAACAGAGCCAAAAACATCAGTGGTCAGAGTCCGGCGCGAACT
TCTTCCGACCCAGGGACCAACACAACAACTGAAGACCACAAAATCATGGCTTCAGAAAATTCCTCTGCAATGGTT
CAAGTGCACAGTCAAGGAAGGGAAGCTGCAGTGTCGCATCTGACAACCCTTGCCACAATCTCCACGAGTCCTCA
ACCCCCCACAACCAAACCAGGTCCGGACAACAGCACCCACAATACACCCGTGTATAAACTTGACATCTCTGAGG
CAACTCAAGTTGAACAACATCACCGCAGAACAGACAACGACAGCACAGCCTCCGACACTCCCCCCGCCACGAC
CGCAGCCGGACCCCTAAAAGCAGAGAACACCAACACGAGCAAGGGTACCGACCTCCTGGACCCCGCCACCACA
ACAAGTCCCCAAAACCACAGCGAGACCGCTGGCAACAACAACACTCATCACCAAGATACCGGAGAAGAGAGTG
CCAGCAGCGGGAAGCTAGGCTTAATTACCAATACTATTGCTGGAGTCGCAGGACTGATCACAGGCGGGAGGAG
AGCTCGAAGAGAAGCAATTGTCAATGCTCAACCCAAATGCAACCCTAATTTACATTACTGGACTACTCAGGATGA
AGGTGCTGCAATCGGACTGGCCTGGATACCATATTTCGGGCCAGCAGCCGAGGGAATTTACATAGAGGGGCTG
ATGCACAATCAAGATGGTTTAATCTGTGGGTTGAGACAGCTGGCCAACGAGACGACTCAAGCTCTTCAACTGTTC
CTGAGAGCCACAACCGAGCTACGCACCTTTTCAATCCTCAACCGTAAGGCAATTGATTTCTTGCTGCAGCGATG
GGGCGGCACATGCCACATTTTGGGACCGGACTGCTGTATCGAACCACATGATTGGACCAAGAACATAACAGACA
AAATTGATCAGATTATTCATGATTTTGTTGATAAAACCCTTCCGGACCAGGGGGACAATGACAATTGGTGGACAG
GATGGAGACAATGGATACCGGCAGGTATTGGAGTTACAGGCGTTATAATTGCAGTTATCGCTTTATTCTGTATAT
GCAAATTTGTCTTTTAGTAAGGCTGACTAAAACACTATATAACCTTCTACTTGATCACAATACTCCGTATACCTATT
ATCATATATTTAATCAAGACGATATCCTTTAAGACTTATTCAGTACTATAATCACTCTCGTTTCAAATTAATAAGATG
TGCATGATTGCCCTAATATATGAAGAGGTATGATACAACCCTAACAGTGACCAAAGAAAATCATAATCTCGTATCG
CTCGTAATATAACCTGCCAAGCATACCTCTTGCACAAAGTGATTCTTGTACACAAATAATGTTTTACCCTACAGGA
GGTAGCAACGATCCATCCCATCAAAAAATAAGTATTTTATGACTTACTAATGACCTCTTAAAATATTAAGAAAAACT
GACGGAACATAAATTCTTTCTGCTTCAAGTTGTGGAGGAGGTGTTTGGTATTGGCTATTGTTATATTACAATCAAT
AACAAGCTTGTAAAAATATTGTTCTTGTTTCAAGAGGTAGATTGTGGCTGGAAATGCTAAACTAATGATGAAGATT
AATGCGGAGGTCTGATAAGAATAAACCTTATTATTCAGATTAGGCCCCAAGAGGCATTCTTCATCTCCTTTTAGCA
AAGTACTATTTCAGGGTAGTCCAATTAGTGACACGTCTCTTAGCTGTATATCAGTCGCCCCTGAGATACGCCACA
AAAGTGTCTCTAAGCTAAATTGGTCTGTACACATCTCATACATTGTATTAGGGACAATAATATCTAATTGAACTTAG
CCGTTTAAAATTTAGTGCATAAATCTGGGCTAACTCCACCAGGTCAACTCCATTGGCTGAAAAGAAGCCTACCTA
CAACGAACATCACTTTGAGCGCCCTCACATTTAAAAAATAGGAACGTCGTTCCAACAATCGAGCGCAAGGTTTCA
AGGTTGAACTGAGAGTGTCTAGACAACAAAGTATCGATCCTCCAGACACCAAGCAAGACCTGAGAAAAAACCAT
GGCTAAAGCTACGGGACGATACAATCTAATATCGCCCAAAAAGGACCTGGAGAAAGGGGTTGTCTTAAGCGACC
TCTGTAACTTCTTAGTTAGCCAAACTATTCAGGGGTGGAAGGTTTATTGGGCTGGTATTGAGTTTGATGTGACTCA
CAAAGGAATGGCCCTATTGCAAAGACTGAAAACTAATGACTTTGCCCCTGCATGGTCAATGACAAGGAATCTCTT
TCCTCATTTATTTCAAAATCCGAATTCCACAATTGAATCACCGCTGTGGGCATTGAGAGTCATCCTTGCAGCAGG
GATACAAGACCAGCTGATTGACCAGTCTTTGATTGAACCCTTAGCAGGAGCCCTTGGTCTGATCTCTGATTGGCT
GCTAACAACCAACACTAACCATTTCAACATGCGAACACAACGTGTCAAGGAACAATTGAGCCTAAAAATGCTGTC
GTTGATTCGATCCAATATTCTCAAGTTTATTAACAAATTGGATGCTCTACATGTCGTGAACTACAACGGATTGTTG
AGCAGTATTGAAATTGGAACTCAAAATCATACAATCATCATAACTCGAACTAACATGGGTTTTCTGGTGGAGCTCC
AAGAACCCGACAAATCGGCAATGAACCGCAAGAAGCCTGGGCCGGCGAAATTTTCCCTCCTTCATGAGTCCACA

CTGAAAGCATTTACACAAGGATCCTCAACACGAATGCAAAGTTTGATTCTTGAATTTAATAGCTCTCTTGCTATCT
AACTAAGATGGAATACTTCATATTGAGCTAACTCATATATGCTGACTCAATAGTTATCTTGACATCTCTGCTTTCAT
AATCAGATATATAAGCATAATAAATAAATACGCATATTTCTTGATAATTTGTTTAACCACAGATAAATCCTCACTGT
AAGCCAGCTTCCAAGTTGACACCCTTACAAAAACCAGGACTCAGAATCCCTCAAATAAGAGATTCCAAGACAACA
TCATAGAATTGCTTTATCATATGAATAAGCATTTTATCACCAGAAATCTTATATACTAAATGGTTAATTGTAACTGAA
CCCGCAGGTCACATGTGTTAGGTTTCACAGATTTTATATATTACTAACTCTATACTCGTAATTAACATTAGATAAGT
AGATTAAGAAAAAAATTACCGTGCATAGTATCCTGATACTTGCAAAGGTTGGTTATCAACATACAGATTATAAAAA
ACTCATAAATTGCTCTCATACATCATATTGATCTGATTTCAATAAACAACTATTTAAATAACGAAAGGAGTCCCTAT
ATTATACACTATATTTAGCCTCTCTCCCTGCGTGATAATCAAAAAATTCACAATGCAGCATGTGTGACATATTAATG
CCGCAATGAATTTAACGCAACATAATAAACTCTGCACTCTTTATAATTAAGCTTTAACGAAAGGTCTGGGCTCATA
TTGTTATTGATATAATAATGTTATATCAATGTCCTGTCAGATGGAATAGTGTTTTGGTTGATAACACGACTTCTTAA
AACAAAATTGATCTTTAAGATTAAGTTTTTTATAATTATCATTACTTTAATTTATCGATTTGAAAATGGTAATAGCCTT
AATCTTTGTGTAAAATAAGAGATTAGGTGTAATAACTTTAACATTTTTGTCTAGTAAGTTACTATTTCATACAGAAT
GATAAAATTAAAAGAAAAGGCAGGACTGTAAAATCATAAATACCTTCTTTACAATATAGCAGACTAGATAATAATCT
TTGTGTTAATGATAATTAAGACATTGACCACGCTCATCAGAAGGCTCGCCAAAATAAACGTTGCAAAAAGGATTC
CTGGAAAAATGGTCGCACACAAAAATTTAAAAATAAATCTATTTCTTCTTTTTTGTGTGTCCTATAGTGAGTCGTAT
TAACCCGGGATCGATATCCCCCTGCATTAATGAATCGGCCAACGCGCGGGGAGAGGCGGTTTGCGTATTGGGC
GCTCTTCCGCTTCCTCGCTCACTGACTCGCTGCGCTCGGTCGTTCGGCTGCGGCGAGCGGTATCAGCTCACTC
AAAGGCGGTAATACGGTTATCCACAGAATCAGGGGATAACGCAGGAAAGAACATGTGAGCAAAAGGCCAGCAAA
AGGCCAGGAACCGTAAAAAGGCCGCGTTGCTGGCGTTTTTCCATAGGCTCCGCCCCCCTGACGAGCATCACAA
AAATCGACGCTCAAGTCAGAGGTGGCGAAACCCGACAGGACTATAAAGATACCAGGCGTTTCCCCCTGGAAGCT
CCCTCGTGCGCTCTCCTGTTCCGACCCTGCCGCTTACCGGATACCTGTCCGCCTTTCTCCCTTCGGGAAGCGTG
GCGCTTTCTCATAGCTCACGCTGTAGGTATCTCAGTTCGGTGTAGGTCGTTCGCTCCAAGCTGGGCTGTGTGCA
CGAACCCCCCGTTCAGCCCGACCGCTGCGCCTTATCCGGTAACTATCGTCTTGAGTCCAACCCGGTAAGACACG
ACTTATCGCCACTGGCAGCAGCCACTGGTAACAGGATTAGCAGAGCGAGGTATGTAGGCGGTGCTACAGAGTT
CTTGAAGTGGTGGCCTAACTACGGCTACACTAGAAGAACAGTATTTGGTATCTGCGCTCTGCTGAAGCCAGTTAC
CTTCGGAAAAAGAGTTGGTAGCTCTTGATCCGGCAAACAAACCACCGCTGGTAGCGGTGGTTTTTTTGTTTGCAA
GCAGCAGATTACGCGCAGAAAAAAAGGATCTCAAGAAGATCCTTTGATCTTTTCTACGGGGTCTGACGCTCAGT
GGAACGAAAACTCACGTTAAGGGATTTTGGTCATGAGATTATCAAAAAGGATCTTCACCTAGATCCTTTTAAATTA
AAAATGAAGTTTTAAATCAATCTAAAGTATATATGAGTAAACTTGGTCTGACAGTTACCAATGCTTAATCAGTGAG
GCACCTATCTCAGCGATCTGTCTATTTCGTTCATCCATAGTTGCCTGACTCCCCGTCGTGTAGATAACTACGATA
CGGGAGGGCTTACCATCTGGCCCCAGTGCTGCAATGATACCGCGAGACCCACGCTCACCGGCTCCAGATTTAT
CAGCAATAAACCAGCCAGCCGGAAGGGCCGAGCGCAGAAGTGGTCCTGCAACTTTATCCGCCTCCATCCAGTC
TATTAATTGTTGCCGGGAAGCTAGAGTAAGTAGTTCGCCAGTTAATAGTTTGCGCAACGTTGTTGCCATTGCTAC
AGGCATCGTGGTGTCACGCTCGTCGTTTGGTATGGCTTCATTCAGCTCCGGTTCCCAACGATCAAGGCGAGTTA
CATGATCCCCCATGTTGTGCAAAAAAGCGGTTAGCTCCTTCGGTCCTCCGATCGTTGTCAGAAGTAAGTTGGCC
GCAGTGTTATCACTCATGGTTATGGCAGCACTGCATAATTCTCTTACTGTCATGCCATCCGTAAGATGCTTTTCTG
TGACTGGTGAGTACTCAACCAAGTCATTCTGAGAATAGTGTATGCGGCGACCGAGTTGCTCTTGCCCGGCGTCA
ATACGGGATAATACCGCGCCACATAGCAGAACTTTAAAAGTGCTCATCATTGGAAAACGTTCTTCGGGGCGAAAA
CTCTCAAGGATCTTACCGCTGTTGAGATCCAGTTCGATGTAACCCACTCGTGCACCCAACTGATCTTCAGCATCT
TTTACTTTCACCAGCGTTTCTGGGTGAGCAAAAACAGGAAGGCAAAATGCCGCAAAAAAGGGAATAAGGGCGAC
ACGGAAATGTTGAATACTCATACTCTTCCTTTTTCAATATTATTGAAGCATTTATCAGGGTTATTGTCTCATGAGCG
GATACATATTTGAATGTATTTAGAAAAATAAACAAATAGGGGTTCCGCGCACATTTCCCCGAAAAGTGCCACCTG
ACGTCTAAGAAACCATTATTATCATGACATTAACCTATAAAAATAGGCGTATCACGAGGCCCTTTCGTCTCGCGC
GTTTCGGTGATGACGGTGAAAACCTCTGACACATGCAGCTCCCGGAGACGGTCACAGCTTGTCTGTAAGCGGAT
GCCGGGAGCAGACAAGCCCGTCAGGGCGCGTCAGCGGGTGTTGGCGGGTGTCGGGGCTGGCTTAACTATGCG
GCATCAGAGCAGATTGTACTGAGAGTGCACCATTCGACGCTCTCCCTTATGCGACTCCTGCATTAAGAAGCAGC
CCAGTAGTAGGTTGAGGCCGTTGAGCACCGCCGCCGCAAGGAATGGTGCAAGGAGATGGCGCCCAACAGTCC
CCCGGCCACGGGGCCTGCCACCATACCCACGCCGAAACAAGCGCTCATGAGCCCGAAGTGGCGAGCCCGATC
TTCCCCATCGGTGATGTCGGCGATATAGGCGCCAGCAACCGCACCTGTGGCGCCGGTGATGCCGGCCACGATG
CGTCCGGCGTAGAGGATCTGGCTAGCGATGACCCTGCTGATTGGTTCGCTGACCATTTCCGGGTGCGGGACGG
CGTTACCAGAAACTCAGAAGGTTCGTCCAACCAAACCGACTCTGACGGCAGTTTACGAGAGAGATGATAGGGTC
TGCTTCAGTAAGCCAGATGCTACACAATTAGGCTTGTACATATTGTCGTTAGAACGCGGCTACAATTAATACATAA
CCTTATGTATCATACACATACGATTTAGGTGACACTATAGAATACAAGCTTGCATGCCTGCAGGTCGACTCTAGA

GGATCTCGATCCGGATATAGTTCCTCCTTTCAGCAAAAAACCCCTCAAGACCCGTTTAGAGGCCCCAAGGGGTT
ATGCTAGTTATTGCTCAGCGGTGGCAGCAGCCAACTCAGCTTCCTTTCGGGCTTTGTTAGCAGCCGGATCCTTTT
TTTTGAGCTCTCCCTTAGCCATCCGAGTGGACGACGTCCTCCTTCGGATGCCCAGGTCGGACCGCGAGGAGGT
GGAGATGCCATGCCGACCC

2. EBOV/Kikwit NP-P2A-VP35

GACATTGATTATTGACTAGTTATTAATAGTAATCAATTACGGGGTCATTAGTTCATAGCCCATATATGGAGTTCCG
CGTTACATAACTTACGGTAAATGGCCCGCCTGGCTGACCGCCCAACGACCCCCGCCCATTGACGTCAATAATGA
CGTATGTTCCCATAGTAACGCCAATAGGGACTTTCCATTGACGTCAATGGGTGGAGTATTTACGGTAAACTGCCC
ACTTGGCAGTACATCAAGTGTATCATATGCCAAGTACGCCCCCTATTGACGTCAATGACGGTAAATGGCCCGCCT
GGCATTATGCCCAGTACATGACCTTATGGGACTTTCCTACTTGGCAGTACATCTACGTATTAGTCATCGCTATTAC
CATGGTGATGCGGTTTTGGCAGTACATCAATGGGCGTGGATAGCGGTTTGACTCACGGGGATTTCCAAGTCTCC
ACCCCATTGACGTCAATGGGAGTTTGTTTTGGCACCAAAATCAACGGGACTTTCCAAAATGTCGTAACAACTCCG
CCCCATTGACGCAAATGGGCGGTAGGCGTGTACGGTGGGAGGTCTATATAAGCAGAGCTCGTTTAGTGAACCG
TCAGATCGCCTGGAGACGCCATCCACGCTGTTTTGACCTCCATAGAAGACACCGGGACCGATCCAGCCTCCGG
AATGGATTCTCGTCCTCAGAAAGTCTGGATGACGCCGAGTCTCACTGAATCTGACATGGATTACCACAAGATCTT
GACAGCAGGTCTGTCCGTTCAACAGGGGATTGTTCGGCAAAGAGTCATCCCAGTGTATCAAGTAAACAATCTTG
AGGAGATTTGCCAACTTATCATACAGGCCTTTGAAGCAGGTGTTGATTTTCAAGAGAGTGCGGACAGTTTCCTTC
TCATGCTTTGTCTTCATCATGCGTACCAGGGAGATTACAAACTTTTCTTGGAAAGTGGCGCAGTCAAGTATTTGG
AAGGGCACGGGTTCCGTTTTGAAGTCAAGAAGCGTGATGGAGTGAAGCGCCTTGAGGAATTGCTGCCAGCAGT
ATCTAGTGGAAAAAACATTAAGAGAACACTTGCTGCCATGCCGGAAGAGGAGACAACTGAAGCTAATGCCGGTC
AGTTTCTCTCCTTTGCAAGTCTATTCCTTCCGAAATTGGTAGTAGGAGAAAAGGCTTGTCTTGAGAAGGTTCAAAG
GCAAATTCAAGTACATGCAGAGCAAGGACTGATACAATATCCAACAGCTTGGCAATCAGTAGGACACATGATGGT
GATTTTCCGTTTGATGCGAACAAATTTTTTGATCAAATTTCTCCTAATACACCAAGGGATGCACATGGTTGCCGGG
CATGATGCCAACGACGCTGTGATTTCAAATTCAGTGGCTCAAGCTCGTTTTTCAGGTTTATTGATTGTCAAAACAG
TACTTGATCATATCCTACAAAAGACAGAACGCGGAGTTCGTCTCCATCCTCTTGCAAGGACCGCCAAGGTAAAAA
ATGAGGTGAACTCCTTTAAGGCTGCACTCAGCTCCCTGGCCAAGCATGGAGAGTATGCTCCTTTCGCCCGACTT
TTGAACCTTTCTGGAGTAAATAATCTTGAGCATGGTCTTTTCCCTCAACTATCAGCAATTGCACTCGGAGTCGCCA
CAGCACACGGGAGTACCCTCGCAGGAGTAAATGTTGGAGAACAGTATCAACAACTCAGAGAGGCTGCCACTGA
GGCTGAGAAGCAACTCCAACAATACGCAGAGTCTCGCGAACTTGACCATCTTGGACTTGATGATCAGGAAAAGA
AAATTCTTATGAACTTCCATCAGAAAAAGAACGAAATCAGCTTCCAGCAAACAAACGCTATGGTAACTCTAAGAAA
AGAGCGCCTGGCCAAGCTGACGGAAGCTATCACTGCTGCGTCACTGCCCAAAACAAGTGGACATTACGATGATG
ATGACGACATTCCCTTTCCAGGACCCATCAATGATGACGACAATCCTGGCCATCAAGATGATGATCCAACTGACT
CACAAGATACGACCATTCCTGATGTGGTGGTTGATCCCGATGATGGAAGCTACGGCGAATACCAGAGTTACTCG
GAAAACGGCATGAATGCACCAGATGACTTGGTCCTATTCGATCTAGACGAGGACGATGAGGACACTAAGCCAGT
GCCTAATAGATCAACCAAGGGTGGACAACAGAAAAACAGTCAAAAGGGCCAGCATACAGAGGGCAGACAGACA
CAATCCAGGCCAACTCAAAATGTCCCAGGCCCTCACAGAACAATCCACCACGCCAGTGCTCCACTCACGGATAA
TGACAGAAGAAATGAACCCTCCGGCTCAACCAGCCCTCGCATGCTGACACCAATCAACGAAGAGGCAGACCCA
CTGGACGATGCCGACGACGAGACGTCTAGCCTTCCGCCCCTGGAGTCAGACGATGAAGAACAGGACAGGGAC
GGAACTTCCAACCGCACACCCACTGTCGCCCCACCGGCTCCCGTATACAGAGATCACTCTGAAAAGAGAGAACT
CCCGCAAGATGAGCAACAAGATCAGGACCACACTCAAGAGGCCAGGAACCAGGACAGTGACAACACCCAGCCA
GAACACTCTTTTGAGGAGATGTATCGCCACATTCTAAGATCACAGGGGCCATTTGATGCTGTTTTGTATTATCATA
TGATGAAGGATGAGCCTGTAGTTTTCAGTACCAGTGATGGCAAAGAGTACACGTATCCAGACTCCCTTGAAGAG
GAATATCCACCATGGCTCACTGAAAAAGAGGCTATGAATGAAGAGAATAGATTTGTTACATTGGATGGTCAACAA
TTTTATTGGCCGGTAATGAATCACAAGAATAAATTCATGGCAATCCTGCAACATCATCAGGGATCCGGCGCTACT
AACTTCAGCCTGCTGAAGCAGGCTGGAGACGTGGAGGAGAACCCTGGACCTATGACAACCAGAACAAAGGGCA
GGGGCCACACTGCGGCCACGACTCAAAACGACAGAATGCCAGGCCCTGAGCTTTCGGGCTGGATCTCTGAGCA
GCTAATGACCGGAAGAATTCCTGTAAGCGACATCTTCTGTGATATTGAGAACAATCCAGGATTATGCTACGCATC
CCAAATGCAACAAACCAAGCCAAACCCGAAGACGCGCAACAGTCAAACCCAAACGGACCCAATTTGCAATCATA
GTTTTGAGGAGGTAGTACAAACATTAGCTTCATTGGCTACTGTTGTGCAACAACAAACCATTGCATCAGAATCATT
AGAACAACGCATTACGAGTCTTGAGAATGGTCTAAAGCCAGTTTATGATATGGCTAAAACAATCTCCTCATTGAAC
AGGGTTTGTGCTGAGATGGTTGCAAAATATGATCTTCTGGTGATGACAACCGGTCGGGCAACAGCAACCGCTGC
GGCAACTGAGGCTTATTGGGCCGAACATGGTCAACCACCACCTGGACCATCACTTTATGAAGAAAGTGCAATTC
GGGGTAAGATTGAATCTAGAGATGAGACCGTCCCTCAAAGTGTTAGGGAGGCATTCAACAATCTAGACAGTACC
ACTTCACTAACTGAGGAAAATTTTGGGAAACCTGACATTTCGGCAAAGGATTTGAGAAACATTATGTATGATCACT
TGCCTGGTTTTGGAACTGCTTTCCACCAATTAGTACAAGTGATTTGTAAATTGGGAAAAGATAGCAACTCATTGGA
CATCATTCATGCTGAGTTCCAGGCCAGCCTGGCTGAAGGAGACTCTCCTCAATGTGCCCTAATTCAAATTACAAA
AAGAGTTCCAATCTTCCAAGATGCTGCTCCACCTGTCATCCACATCCGCTCTCGAGGTGACATTCCCCGAGCTTG
CCAGAAAAGCTTGCGTCCAGTCCCACCATCGCCCAAGATTGATCGAGGTTGGGTATGTGTTTTTCAGCTTCAAGA

TGGTAAAACACTTGGACTCAAAATTTGATCTAGAGGTAAGCCTATCCCTAACCCTCTCCTCGGTCTCGATTCTAC
GTAAGATCTAGAACTAGTGTCGACGCAAGGGTTCGATCCCTACCGGTTAGTAATGAGTTTAATCAACCTCTGGAT
TACAAAATTTGTGAAAGATTGACTGGTATTCTTAACTATGTTGCTCCTTTTACGCTATGTGGATACGCTGCTTTAAT
GCCTTTGTATCATGCTATTGCTTCCCGTATGGCTTTCATTTTCTCCTCCTTGTATAAATCCTGGTTGCTGTCTCTTT
ATGAGGAGTTGTGGCCCGTTGTCAGGCAACGTGGCGTGGTGTGCACTGTGTTTGCTGACGCAACCCCCACTGG
TTGGGGCATTGCCACCACCTGTCAGCTCCTTTCCGGGACTTTCGCTTTCCCCCTCCCTATTGCCACGGCGGAAC
TCATCGCCGCCTGCCTTGCCCGCTGCTGGACAGGGGCTCGGCTGTTGGGCACTGACAATTCCGTGGTGTTGTC
GGGGAAGCTGACGTCCTTTCCATGGCTGCTCGCCTGTGTTGCCACCTGGATTCTGCGCGGGACGTCCTTCTGCT
ACGTCCCTTCGGCCCTCAATCCAGCGGACCTTCCTTCCCGCGGCCTGCTGCCGGCTCTGCGGCCTCTTCCGCG
TCTTCGCCTTCGCCCTCAGACGAGTCGGATCTCCCTTTGGGCCGCCTCCCCGCAAACGGGGGAGGCTAACTGA
AACACGGAAGGAGACAATACCGGAAGGAACCCGCGCTATGACGGCAATAAAAAGACAGAATAAAACGCACGGG
TGTTGGGTCGTTTGTTCATAAACGCGGGGTTCGGTCCCAGGGCTGGCACTCTGTCGATACCCCACCGAGACCC
CATTGGGGCCAATACGCCCGCGTTTCTTCCTTTTCCCCACCCCACCCCCCAAGTTCGGGTGAAGGCCCAGGGCT
CGCAGCCAACGTCGGGGCGGCAGGCCCTGCCATAGCAGATCTGCGCAGCTGTTTGCAAAAGCCTAGGCCTCCA
AAAAAGCCTCCTCACTACTTCTGGAATAGCTCAGAGGCAGAGGCGGCCTCGGCCTCTGCATAAATAAAAAAAATT
AGTCAGCCATGGGGCGGAGAATGGGCGGAACTGGGCGGAGTTAGGGGCGGGATGGGCGGAGTTAGGGGCGG
GACTATGGTTGCTGACTAATTGAGATGCATGCTTTGCATACTTCTGCCTGCTGGGGAGCCTGGGGACTTTCCACA
CCTGGTTGCTGACTAATTGAGATGCATGCTTTGCATACTTCTGCCTGCTGGGGAGCCTGGGGACTTTCCACACC
CTAACTGACACACCAGCTGCATTAATGAATCGGCCAACGCGCGGGGAGAGGCGGTTTGCGTATTGGGCGCTCT
TCCGCTTCCTCGCTCACTGACTCGCTGCGCTCGGTCGTTCGGCTGCGGCGAGCGGTATCAGCTCACTCAAAGG
CGGTAATACGGTTATCCACAGAATCAGGGGATAACGCAGGAAAGAACATGTGAGCAAAAGGCCAGCAAAAGGC
CAGGAACCGTAAAAAGGCCGCGTTGCTGGCGTTTTTCCATAGGCTCCGCCCCCCTGACGAGCATCACAAAAATC
GACGCTCAAGTCAGAGGTGGCGAAACCCGACAGGACTATAAAGATACCAGGCGTTTCCCCCTGGAAGCTCCCT
CGTGCGCTCTCCTGTTCCGACCCTGCCGCTTACCGGATACCTGTCCGCCTTTCTCCCTTCGGGAAGCGTGGCG
CTTTCTCATAGCTCACGCTGTAGGTATCTCAGTTCGGTGTAGGTCGTTCGCTCCAAGCTGGGCTGTGTGCACGA
ACCCCCCGTTCAGCCCGACCGCTGCGCCTTATCCGGTAACTATCGTCTTGAGTCCAACCCGGTAAGACACGACT
TATCGCCACTGGCAGCAGCCACTGGTAACAGGATTAGCAGAGCGAGGTATGTAGGCGGTGCTACAGAGTTCTT
GAAGTGGTGGCCTAACTACGGCTACACTAGAAGAACAGTATTTGGTATCTGCGCTCTGCTGAAGCCAGTTACCTT
CGGAAAAAGAGTTGGTAGCTCTTGATCCGGCAAACAAACCACCGCTGGTAGCGGTGGTTTTTTTGTTTGCAAGC
AGCAGATTACGCGCAGAAAAAAAGGATCTCAAGAAGATCCTTTGATCTTTTCTACGGGGTCTGACGCTCAGTGGA
ACGAAAACTCACGTTAAGGGATTTTGGTCATGAGATTATCAAAAAGGATCTTCACCTAGATCCTTTTAAATTAAAA
ATGAAGTTTTAAATCAATCTAAAGTATATATGAGTAAACTTGGTCTGACAGTTACCAATGCTTAATCAGTGAGGCA
CCTATCTCAGCGATCTGTCTATTTCGTTCATCCATAGTTGCCTGACTCCCCGTCGTGTAGATAACTACGATACGG
GAGGGCTTACCATCTGGCCCCAGTGCTGCAATGATACCGCGAGACCCACGCTCACCGGCTCCAGATTTATCAG
CAATAAACCAGCCAGCCGGAAGGGCCGAGCGCAGAAGTGGTCCTGCAACTTTATCCGCCTCCATCCAGTCTATT
AATTGTTGCCGGGAAGCTAGAGTAAGTAGTTCGCCAGTTAATAGTTTGCGCAACGTTGTTGCCATTGCTACAGGC
ATCGTGGTGTCACGCTCGTCGTTTGGTATGGCTTCATTCAGCTCCGGTTCCCAACGATCAAGGCGAGTTACATG
ATCCCCCATGTTGTGCAAAAAAGCGGTTAGCTCCTTCGGTCCTCCGATCGTTGTCAGAAGTAAGTTGGCCGCAG
TGTTATCACTCATGGTTATGGCAGCACTGCATAATTCTCTTACTGTCATGCCATCCGTAAGATGCTTTTCTGTGAC
TGGTGAGTACTCAACCAAGTCATTCTGAGAATAGTGTATGCGGCGACCGAGTTGCTCTTGCCCGGCGTCAATAC
GGGATAATACCGCGCCACATAGCAGAACTTTAAAAGTGCTCATCATTGGAAAACGTTCTTCGGGGCGAAAACTCT
CAAGGATCTTACCGCTGTTGAGATCCAGTTCGATGTAACCCACTCGTGCACCCAACTGATCTTCAGCATCTTTTA
CTTTCACCAGCGTTTCTGGGTGAGCAAAAACAGGAAGGCAAAATGCCGCAAAAAAGGGAATAAGGGCGACACG
GAAATGTTGAATACTCATACTCTTCCTTTTTCAATATTATTGAAGCATTTATCAGGGTTATTGTCTCATGAGCGGAT
ACATATTTGAATGTATTTAGAAAAATAAACAAATAGGGGTTCCGCGCACATTTCCCCGAAAAGTGCCACCTGACG
TCGACGGATCGGGAGATCTCCCGATCCCCTATGGTGCACTCTCAGTACAATCTGCTCTGATGCCGCATAGTTAA
GCCAGTATCTGCTCCCTGCTTGTGTGTTGGAGGTCGCTGAGTAGTGCGCGAGCAAAATTTAAGCTACAACAAGG
CAAGGCTTGACCGACAATTGCATGAAGAATCTGCTTAGGGTTAGGCGTTTTGCGCTGCTTCGCGATGTACGGGC
CAGATATACGCGTT

3. EBOV/Kikwit L

GACATTGATTATTGACTAGTTATTAATAGTAATCAATTACGGGGTCATTAGTTCATAGCCCATATATGGAGTTCCG
CGTTACATAACTTACGGTAAATGGCCCGCCTGGCTGACCGCCCAACGACCCCCGCCCATTGACGTCAATAATGA
CGTATGTTCCCATAGTAACGCCAATAGGGACTTTCCATTGACGTCAATGGGTGGAGTATTTACGGTAAACTGCCC
ACTTGGCAGTACATCAAGTGTATCATATGCCAAGTACGCCCCCTATTGACGTCAATGACGGTAAATGGCCCGCCT
GGCATTATGCCCAGTACATGACCTTATGGGACTTTCCTACTTGGCAGTACATCTACGTATTAGTCATCGCTATTAC
CATGGTGATGCGGTTTTGGCAGTACATCAATGGGCGTGGATAGCGGTTTGACTCACGGGGATTTCCAAGTCTCC
ACCCCATTGACGTCAATGGGAGTTTGTTTTGGCACCAAAATCAACGGGACTTTCCAAAATGTCGTAACAACTCCG
CCCCATTGACGCAAATGGGCGGTAGGCGTGTACGGTGGGAGGTCTATATAAGCAGAGCTCGTTTAGTGAACCG
TCAGATCGCCTGGAGACGCCATCCACGCTGTTTTGACCTCCATAGAAGACACCGGGACCGATCCAGCCTCCGG
AATGGCTACACAACATACCCAATACCCGGACGCTAGGTTATCATCACCAATTGTATTGGACCAATGTGACCTAGT
CACTAGAGCTTGCGGGTTATATTCATCATACTCCCTTAATCCGCAACTACGCAACTGTAAACTCCCGAAACATATC
TACCGTTTGAAATACGATGTAACTGTTACCAAGTTCTTGAGTGATGTACCAGTGGCGACATTGCCCATAGATTTCA
TAGTCCCAATTCTTCTCAAGGCACTGTCAGGCAATGGGTTCTGTCCTGTTGAGCCGCGGTGCCAACAGTTCTTAG
ATGAAATCATTAAGTACACAATGCAAGATGCTCTCTTCTTGAAATATTATCTCAAAAATGTGGGTGCTCAAGAAGA
CTGTGTTGATGACCACTTTCAAGAGAAAATCTTATCTTCAATTCAGGGCAATGAATTTTTACATCAAATGTTTTTCT
GGTATGATCTGGCTATTTTAACTCGAAGGGGTAGATTAAATCGAGGAAACTCTAGATCAACATGGTTTGTTCATG
ATGATTTAATAGACATCTTAGGTTATGGGGACTATGTTTTTTGGAAGATCCCAATTTCAATGTTACCACTGAACAC
ACAAGGAATCCCCCATGCTGCTATGGACTGGTATCAGGCATCAGTATTCAAAGAAGCGGTTCAAGGGCATACAC
ACATTGTTTCTGTTTCTACTGCCGACGTCTTGATAATGTGCAAAGATTTAATTACATGTCGATTCAACACAACTCTA
ATCTCAAAAATAGCAGAGATTGAGGATCCAGTTTGTTCTGATTATCCCAATTTTAAGATTGTGTCTATGCTTTACCA
GAGCGGAGATTACTTACTCTCCATATTAGGGTCTGATGGGTATAAAATTATTAAGTTCCTCGAACCATTGTGCTTG
GCCAAAATTCAATTATGCTCAAAGTACACCGAGAGGAAGGGCCGATTCTTAACACAAATGCATTTAGCTGTAAAT
CACACCCTAGAAGAAATTACAGAAATGCGTGCACTAAAGCCTTCACAGGCTCAAAAGATCCGTGAATTCCATAGA
ACATTGATAAGGCTGGAGATGACGCCACAACAGCTTTGTGAGCTATTTTCCATTCAAAAACACTGGGGGCATCCT
GTGCTACATAGTGAAACAGCAATCCAAAAAGTTAAAAAACATGCTACGGTGCTAAAAGCATTACGCCCTATAGTG
ATTTTCGAGACATACTGTGTTTTTAAATATAGTATTGCCAAACATTATTTTGATAGTCAAGGGTCTTGGTACAGTGT
TACTTCAGATAGGAATCTAACACCAGGTCTTAATTCTTATATCAAAAGAAATCAATTCCCTCCGTTGCCAATGATTA
AAGAACTACTATGGGAATTTTACCACCTTGACCATCCTCCACTTTTCTCAACCAAAATTATTAGTGACTTAAGTATT
TTTATAAAAGACAGAGCTACTGCAGTAGAAAGGACATGCTGGGATGCAGTATTCGAGCCTAATGTTCTAGGATAT
AATCCACCTCACAAATTTAGTACTAAACGTGTACCGGAACAATTTTTAGAGCAAGAAAACTTTTCTATTGAGAATG
TTCTTTCCTACGCGCAAAAACTCGAGTATCTACTACCACAATATCGGAACTTTTCTTTCTCATTGAAAGAGAAAGA
GTTGAATGTAGGTAGAACCTTCGGAAAATTGCCTTATCCGACTCGCAATGTTCAAACACTTTGTGAAGCTCTGTTA
GCTGATGGTCTTGCTAAAGCATTTCCTAGCAATATGATGGTAGTTACGGAACGTGAGCAAAAAGAAAGCTTATTG
CATCAAGCATCATGGCACCACACAAGTGATGATTTTGGTGAACATGCCACAGTTAGAGGGAGTAGCTTTGTAACT
GATTTAGAGAAATACAATCTTGCATTTAGATATGAGTTTACAGCACCTTTTATAGAATATTGCAACCGTTGCTATG
GTGTTAAGAATGTTTTTAATTGGATGCATTATACAATCCCACAGTGTTATATGCATGTCAGTGATTATTATAATCCA
CCACATAACCTCACACTGGAAAATCGAGACAACCCCCCCGAAGGGCCTAGTTCATACAGGGGTCATATGGGAGG
GATTGAAGGACTGCAACAAAAACTCTGGACAAGTATTTCATGTGCTCAAATTTCTTTAGTTGAAATTAAGACTGGT
TTTAAGTTACGCTCAGCTGTGATGGGTGACAATCAGTGCATTACCGTTTTATCAGTCTTCCCCTTAGAGACTGAC
GCAGACGAGCAGGAACAGAGCGCCGAAGACAATGCAGCGAGGGTGGCCGCTAGCCTAGCAAAAGTTACAAGT
GCCTGTGGAATCTTTTTAAAACCTGATGAAACATTTGTACATTCAGGTTTTATCTATTTTGGAAAAAAACAATATTT
GAATGGGGTCCAATTGCCTCAGTCCCTTAAAACGGCTACAAGAATGGCACCATTGTCTGATGCAATTTTTGATGA
TCTTCAAGGGACCCTAGCTAGTATAGGCACTGCTTTTGAGCGATCCATCTCTGAGACACGACATATCTTTCCTTG
CAGGATAACCGCAGCTTTCCATACGTTTTTTTCGGTGAGAATCTTGCAATATCATCATCTCGGGTTCAATAAAGGT
TTTGACCTTGGACAGTTAACACTCGGTAAACCTCTGGATTTCGGAACAATATCATTGGCACTAGCGGTACCGCAG
GTGCTTGGAGGATTATCCTTCTTGAATCCTGAGAAATGTTTCTACCGGAATCTAGGAGATCCAGTTACCTCAGGT
TTATTCCAGTTAAAAACTTATCTCCGAATGATTGAGATGGATGATTTATTCTTACCTTTAATTGCAAAGAACCCTGG
GAACTGCACTGCCATTGACTTTGTGCTAAATCCTAGCGGATTAAATGTCCCTGGGTCGCAAGACTTAACTTCATTT
CTGCGCCAGATTGTACGCAGGACCATCACCCTAAGTGCGAAAAACAAACTTATTAATACCTTATTTCATGCGTCA
GCTGACTTCGAAGACGAAATGGTTTGTAAATGGCTATTATCATCAACTCCTGTTATGAGTCGTTTTGCGGCCGAT
ATCTTTTCACGCACGCCGAGCGGGAAGCGATTGCAAATTCTAGGATACCTGGAAGGAACACGCACATTATTAGC
CTCTAGGATCATCAACAATAATACAGAGACACCGGTTTTGGACAGACTGAGGAAAATAACATTGCAAAGGTGGAG
TCTGTGGTTTAGTTATCTTGATCATTGTGATAATATCCTGGCGGAGGCTTTAACCCAAATAACTTGCACAGTTGAT

TTAGCACAGATCCTGAGGGAATATTCATGGGCTCATATTTAGAGGGAAGACCTCTTATTGGAGCCACACTCCCA
TGTATGATTGAGCAATTCAAAGTGTTTTGGCTGAAACCCTACGAACAATGTCCGCAGTGTTCAAATGCAAAGCAA
CCAGGTGGGAAACCATTCGTGTCAGTGGCAGTCAAGAAACATATTGTTAGTGCATGGCCTAACGCATCCCGACT
AAGCTGGACTATCGGGGATGGAATCCCTTACATTGGATCAAGGACAGAAGATAAGATAGGACAACCTGCTATTAA
ACCAAAATGTCCTTCCGCAGCCTTAAGAGAGGCCATTGAATTGGCGTCCCGTTTAACATGGGTAACTCAAGGCA
GTTCGAACAGTGACTTGCTAATAAAGCCATTTTTGGAAGCACGAGTAAATTTAAGTGTTCAAGAAATACTTCAAAT
GACCCCTTCACATTACTCAGGAAATATTGTTCACAGGTACAACGATCAATACAGTCCTCATTCTTTCATGGCCAAT
CGTATGAGTAATTCAGCAACGCGATTGATTGTTTCTACAAACACTTTAGGTGAGTTTTCAGGAGGTGGCCAGTCT
GCACGCGACAGCAATATTATTTTCCAGAATGTTATAAATTATGCAGTTGCACTGTTCGATATTAAATTTAGAAACA
CTGAGGCTACAGATATCCAATATAATCGTGCTCACCTTCATCTAACTAAGTGTTGCACCCGGGAAGTACCAGCTC
AGTATTTAACATACACATCTACATTGGATTTAGATTTAACAAGATACCGAGAAAACGAATTGATTTATGACAATAAT
CCTCTAAAAGGAGGACTCAATTGCAATATCTCATTCGATAACCCATTTTTCCAAGGTAAACAGCTGAACATTATAG
AAGATGATCTTATTCGACTGCCTCACTTATCTGGATGGGAGCTAGCCAAGACCATCATGCAATCAATTATTTCAGA
TAGCAACAATTCATCTACAGACCCAATTAGCAGTGGAGAAACAAGATCATTCACTACCCATTTCTTAACTTATCCC
AAGATAGGACTTCTGTACAGTTTTGGGGCCTTTGTAAGTTATTATCTTGGCAATACAATTCTTCGGACTAAGAAAT
TGACACTTGACAATTTTTTATATTACTTAACTACCCAAATTCATAATCTACCACATCGCTCATTGCGAATACTTAAG
CCAACATTCAAACATGCAAGCGTTATGTCACGGTTAATGAGTATTGATCCTCATTTTTCTATTTACATAGGCGGTG
CTGCAGGTGACAGAGGACTCTCAGATGCGGCCAGGTTATTTTTGAGAACGTCTATTTCATCTTTTCTTACATTTGT
AAAAGAATGGATAATTAATCGCGGAACAATTGTCCCTTTATGGATAGTATATCCGCTAGAGGGTCAAAACCCAAC
ACCTGTTAATAATTTTCTCTATCAGATCGTAGAACTGCTGGTGCATGATTCATCAAGACAACAGGCTTTAAAAACT
ACCATAAGTGATCATGTACATCCTCACGACAATCTTGTTTACACATGTAAGAGTACAGCCAGCAATTTCTTCCATG
CATCATTGGCGTACTGGAGGAGCAGGCACAGAAACAGCAACCGAAAATACTTGGCAAGAGACTCTTCAACTAGA
TCAAGCACAAACAACAGTGATGGTCATATTGAGAGAAGTCAAGAACAAACCACCAGAGATCCACATGATGGCACT
GAACGGAATCTAGTTCTACAAATGAGCCATGAAATAAAAAGAACGACAATTCCACAAGAAAACACGCACCAGGGT
CCGTCGTTCCAGTCCTTTCTAAGTTACTCTGCTTGTGGTACAGCAAATCCAAAACTAAATTTCGATCGATCGAGAC
ACAATGTGAAATCTCAGGATCATAACTCGGCATCCAAGAGGGAAGGTCATCAAATAATCTCACACCGTCTAGTCC
TACCTTTCTTTACATTATCTCAAGGGACACGCCAATTAACGTCATCTAATGAGTCACAGACCCAAGACGAGATATC
AAAGTACTTACGGCAATTGAGATCCGTCATTGATACCACAGTTTATTGTAGGTTTACCGGTATAGTCTCGTCCATG
CATTACAAACTTGATGAGGTCCTTTGGGAAATAGAGAGTTTTAAGTCGGCTGTGACGCTAGCAGAGGGAGAAGG
TGCTGGTGCCTTACTATTGATTCAGAAATACCAAGTTAAGACCTTATTTTTCAACACGCTAGCTACTGAGTCCAGT
ATAGAGTCAGAAATAGTATCAGGAATGACTACTCCTAGGATGCTTCTACCTGTTATGTCAAAATTCCATAATGACC
AAATTGAGATTATTCTTAACAACTCAGCAAGCCAAATAACAGACATAACAAATCCTACTTGGTTTAAAGACCAAAG
AGCAAGGCTACCTAAGCAAGTCGAGGTTATAACCATGGATGCAGAGACGACAGAGAATATAAACAGATCGAAAT
TGTACGAAGCTGTATATAAACTGATCTTACACCATATTGATCCCAGCGTATTGAAAGCAGTGGTCCTTAAAGTCTT
CCTAAGTGATACTGAGGGTATGTTATGGCTAAATGATAATTTAGCCCCGTTTTTTGCCACTGGTTATTTAATTAAG
CCAATAACGTCAAGTGCTAGATCTAGTGAGTGGTATCTTTGTCTGACGAACTTCTTATCAACTACACGAAAGATG
CCACACCAAAACCATCTCAGTTGTAAACAGGTAATACTTACGGCATTGCAACTGCAAATTCAACGGAGCCCATAC
TGGCTAAGTCATTTAACTCAGTATGCTGACTGTGATTTACATTTAAGTTATATCCGCCTTGGTTTTCCATCATTAGA
GAAAGTACTATACCACAGGTATAACCTCGTCGATTCAAAAAGAGGTCCACTAGTCTCTATCACTCAGCACTTAGC
ACATCTTAGAGCAGAGATTCGAGAATTAACTAATGATTATAATCAACAGCGACAAAGTCGAACTCAAACATATCAC
TTTATTCGTACTGCAAAAGGACGAATCACAAAACTAGTCAATGATTATTTAAAATTCTTTCTTATTGTGCAAGCATT
AAAACATAATGGGACATGGCAAGCTGAGTTTAAGAAATTACCAGAGTTGATTAGTGTGTGCAATAGGTTCTACCA
TATTAGAGATTGCAATTGTGAAGAACGTTTCTTAGTTCAAACCTTATATTTACATAGAATGCAGGATTCTGAAGTTA
AGCTTATCGAAAGGCTGACAGGGCTTCTGAGTTTATTTCCGGATGGTCTCTACAGGTTTGATTGATCTAGAGGTA
AGCCTATCCCTAACCCTCTCCTCGGTCTCGATTCTACGTAAGATCTAGAACTAGTGTCGACGCAAGGGTTCGATC
CCTACCGGTTAGTAATGAGTTTAATCAACCTCTGGATTACAAAATTTGTGAAAGATTGACTGGTATTCTTAACTAT
GTTGCTCCTTTTACGCTATGTGGATACGCTGCTTTAATGCCTTTGTATCATGCTATTGCTTCCCGTATGGCTTTCA
TTTTCTCCTCCTTGTATAAATCCTGGTTGCTGTCTCTTTATGAGGAGTTGTGGCCCGTTGTCAGGCAACGTGGCG
TGGTGTGCACTGTGTTTGCTGACGCAACCCCCACTGGTTGGGGCATTGCCACCACCTGTCAGCTCCTTTCCGGG
ACTTTCGCTTTCCCCCTCCCTATTGCCACGGCGGAACTCATCGCCGCCTGCCTTGCCCGCTGCTGGACAGGGG
CTCGGCTGTTGGGCACTGACAATTCCGTGGTGTTGTCGGGGAAGCTGACGTCCTTTCCATGGCTGCTCGCCTGT
GTTGCCACCTGGATTCTGCGCGGGACGTCCTTCTGCTACGTCCCTTCGGCCCTCAATCCAGCGGACCTTCCTTC
CCGCGGCCTGCTGCCGGCTCTGCGGCCTCTTCCGCGTCTTCGCCTTCGCCCTCAGACGAGTCGGATCTCCCTT
TGGGCCGCCTCCCCGCAAACGGGGGAGGCTAACTGAAACACGGAAGGAGACAATACCGGAAGGAACCCGCGC

TATGACGGCAATAAAAAGACAGAATAAAACGCACGGGTGTTGGGTCGTTTGTTCATAAACGCGGGGTTCGGTCC
CAGGGCTGGCACTCTGTCGATACCCCACCGAGACCCCATTGGGGCCAATACGCCCGCGTTTCTTCCTTTTCCCC
ACCCCACCCCCCAAGTTCGGGTGAAGGCCCAGGGCTCGCAGCCAACGTCGGGGCGGCAGGCCCTGCCATAGC
AGATCTGCGCAGCTGTTTGCAAAAGCCTAGGCCTCCAAAAAAGCCTCCTCACTACTTCTGGAATAGCTCAGAGG
CAGAGGCGGCCTCGGCCTCTGCATAAATAAAAAAAATTAGTCAGCCATGGGGCGGAGAATGGGCGGAACTGGG
CGGAGTTAGGGGCGGGATGGGCGGAGTTAGGGGCGGGACTATGGTTGCTGACTAATTGAGATGCATGCTTTGC
ATACTTCTGCCTGCTGGGGAGCCTGGGGACTTTCCACACCTGGTTGCTGACTAATTGAGATGCATGCTTTGCATA
CTTCTGCCTGCTGGGGAGCCTGGGGACTTTCCACACCCTAACTGACACACCAGCTGCATTAATGAATCGGCCAA
CGCGCGGGGAGAGGCGGTTTGCGTATTGGGCGCTCTTCCGCTTCCTCGCTCACTGACTCGCTGCGCTCGGTCG
TTCGGCTGCGGCGAGCGGTATCAGCTCACTCAAAGGCGGTAATACGGTTATCCACAGAATCAGGGGATAACGC
AGGAAAGAACATGTGAGCAAAAGGCCAGCAAAAGGCCAGGAACCGTAAAAAGGCCGCGTTGCTGGCGTTTTTC
CATAGGCTCCGCCCCCCTGACGAGCATCACAAAAATCGACGCTCAAGTCAGAGGTGGCGAAACCCGACAGGAC
TATAAAGATACCAGGCGTTTCCCCCTGGAAGCTCCCTCGTGCGCTCTCCTGTTCCGACCCTGCCGCTTACCGGA
TACCTGTCCGCCTTTCTCCCTTCGGGAAGCGTGGCGCTTTCTCATAGCTCACGCTGTAGGTATCTCAGTTCGGT
GTAGGTCGTTCGCTCCAAGCTGGGCTGTGTGCACGAACCCCCCGTTCAGCCCGACCGCTGCGCCTTATCCGGT
AACTATCGTCTTGAGTCCAACCCGGTAAGACACGACTTATCGCCACTGGCAGCAGCCACTGGTAACAGGATTAG
CAGAGCGAGGTATGTAGGCGGTGCTACAGAGTTCTTGAAGTGGTGGCCTAACTACGGCTACACTAGAAGAACAG
TATTTGGTATCTGCGCTCTGCTGAAGCCAGTTACCTTCGGAAAAAGAGTTGGTAGCTCTTGATCCGGCAAACAAA
CCACCGCTGGTAGCGGTGGTTTTTTTGTTTGCAAGCAGCAGATTACGCGCAGAAAAAAAGGATCTCAAGAAGAT
CCTTTGATCTTTTCTACGGGGTCTGACGCTCAGTGGAACGAAAACTCACGTTAAGGGATTTTGGTCATGAGATTA
TCAAAAAGGATCTTCACCTAGATCCTTTTAAATTAAAAATGAAGTTTTAAATCAATCTAAAGTATATATGAGTAAAC
TTGGTCTGACAGTTACCAATGCTTAATCAGTGAGGCACCTATCTCAGCGATCTGTCTATTTCGTTCATCCATAGTT
GCCTGACTCCCCGTCGTGTAGATAACTACGATACGGGAGGGCTTACCATCTGGCCCCAGTGCTGCAATGATACC
GCGAGACCCACGCTCACCGGCTCCAGATTTATCAGCAATAAACCAGCCAGCCGGAAGGGCCGAGCGCAGAAGT
GGTCCTGCAACTTTATCCGCCTCCATCCAGTCTATTAATTGTTGCCGGGAAGCTAGAGTAAGTAGTTCGCCAGTT
AATAGTTTGCGCAACGTTGTTGCCATTGCTACAGGCATCGTGGTGTCACGCTCGTCGTTTGGTATGGCTTCATTC
AGCTCCGGTTCCCAACGATCAAGGCGAGTTACATGATCCCCCATGTTGTGCAAAAAAGCGGTTAGCTCCTTCGG
TCCTCCGATCGTTGTCAGAAGTAAGTTGGCCGCAGTGTTATCACTCATGGTTATGGCAGCACTGCATAATTCTCT
TACTGTCATGCCATCCGTAAGATGCTTTTCTGTGACTGGTGAGTACTCAACCAAGTCATTCTGAGAATAGTGTATG
CGGCGACCGAGTTGCTCTTGCCCGGCGTCAATACGGGATAATACCGCGCCACATAGCAGAACTTTAAAAGTGCT
CATCATTGGAAAACGTTCTTCGGGGCGAAAACTCTCAAGGATCTTACCGCTGTTGAGATCCAGTTCGATGTAACC
CACTCGTGCACCCAACTGATCTTCAGCATCTTTTACTTTCACCAGCGTTTCTGGGTGAGCAAAAACAGGAAGGCA
AAATGCCGCAAAAAAGGGAATAAGGGCGACACGGAAATGTTGAATACTCATACTCTTCCTTTTTCAATATTATTGA
AGCATTTATCAGGGTTATTGTCTCATGAGCGGATACATATTTGAATGTATTTAGAAAAATAAACAAATAGGGGTTC
CGCGCACATTTCCCCGAAAAGTGCCACCTGACGTCGACGGATCGGGAGATCTCCCGATCCCCTATGGTGCACT
CTCAGTACAATCTGCTCTGATGCCGCATAGTTAAGCCAGTATCTGCTCCCTGCTTGTGTGTTGGAGGTCGCTGAG
TAGTGCGCGAGCAAAATTTAAGCTACAACAAGGCAAGGCTTGACCGACAATTGCATGAAGAATCTGCTTAGGGTT
AGGCGTTTTGCGCTGCTTCGCGATGTACGGGCCAGATATACGCGTT

4. EBOV/Kikwit VP30

GACATTGATTATTGACTAGTTATTAATAGTAATCAATTACGGGGTCATTAGTTCATAGCCCATATATGGAGTTCCG
CGTTACATAACTTACGGTAAATGGCCCGCCTGGCTGACCGCCCAACGACCCCCGCCCATTGACGTCAATAATGA
CGTATGTTCCCATAGTAACGCCAATAGGGACTTTCCATTGACGTCAATGGGTGGAGTATTTACGGTAAACTGCCC
ACTTGGCAGTACATCAAGTGTATCATATGCCAAGTACGCCCCCTATTGACGTCAATGACGGTAAATGGCCCGCCT
GGCATTATGCCCAGTACATGACCTTATGGGACTTTCCTACTTGGCAGTACATCTACGTATTAGTCATCGCTATTAC
CATGGTGATGCGGTTTTGGCAGTACATCAATGGGCGTGGATAGCGGTTTGACTCACGGGGATTTCCAAGTCTCC
ACCCCATTGACGTCAATGGGAGTTTGTTTTGGCACCAAAATCAACGGGACTTTCCAAAATGTCGTAACAACTCCG
CCCCATTGACGCAAATGGGCGGTAGGCGTGTACGGTGGGAGGTCTATATAAGCAGAGCTCGTTTAGTGAACCG
TCAGATCGCCTGGAGACGCCATCCACGCTGTTTTGACCTCCATAGAAGACACCGGGACCGATCCAGCCTCCGG
AATGGAAGCTTCATATGAGAGAGGACGCCCACGAGCTGCCAGACAGCATTCAAGGGATGGACACGACCACCAT
GTTCGAGCACGATCATCATCCAGAGAGAATTATCGAGGTGAGTACCGTCAATCAAGGAGCGCCTCACAAGTGCG
CGTTCCTACTGTATTTCATAAGAAGAGAGTTGAACCATTAACAGTTCCTCCAGCACCTAAAGACATATGTCCGAC
CTTGAAAAAAGGATTTTTGTGTGACAGTAGTTTTTGCAAAAAAGATCACCAGTTGGAGAGTTTAACTGATAGGGAA
TTACTCCTACTAATCGCCCGTAAGACTTGTGGATCAGTAGAACAACAATTAAATATAACTGCACCCAAGGACTCG
CGCTTAGCAAATCCAACGGCTGATGATTTCCAGCAAGAGGAAGGTCCAAAAATTACCTTGTTGACTCTGATCAAG
ACGGCAGAACACTGGGCGAGACAAGACATCAGAACCATAGAGGATTCAAAATTAAGAGCATTGTTGACTCTATGT
GCTGTGATGACGAGGAAATTCTCAAAATCCCAGCTGAGTCTTTTATGTGAGACACACCTAAGGCGTGAGGGGCT
TGGGCAAGATCAGGCAGAACCTGTTCTCGAAGTATATCAACGATTACACAGTGATAAAGGAGGCAGTTTTGAAG
CTGCACTATGGCAACAATGGGACCGACAATCCCTAATTATGTTTATCACTGCATTCTTGAATATCGCTCTCCAGTT
ACCGTGTGAAAGTTCTGCTGTCGTTGTTTCAGGGTTAAGAACATTGGTTCCTCAATCAGATAATGAGGAAGCTTC
AACCAACCCGGGGACATGCTCATGGTCTGATGAGGGTACCCCTTAATCTAGAGGTAAGCCTATCCCTAACCCTC
TCCTCGGTCTCGATTCTACGTAAGATCTAGAACTAGTGTCGACGCAAGGGTTCGATCCCTACCGGTTAGTAATGA
GTTTAATCAACCTCTGGATTACAAAATTTGTGAAAGATTGACTGGTATTCTTAACTATGTTGCTCCTTTTACGCTAT
GTGGATACGCTGCTTTAATGCCTTTGTATCATGCTATTGCTTCCCGTATGGCTTTCATTTTCTCCTCCTTGTATAAA
TCCTGGTTGCTGTCTCTTTATGAGGAGTTGTGGCCCGTTGTCAGGCAACGTGGCGTGGTGTGCACTGTGTTTGC
TGACGCAACCCCCACTGGTTGGGGCATTGCCACCACCTGTCAGCTCCTTTCCGGGACTTTCGCTTTCCCCCTCC
CTATTGCCACGGCGGAACTCATCGCCGCCTGCCTTGCCCGCTGCTGGACAGGGGCTCGGCTGTTGGGCACTGA
CAATTCCGTGGTGTTGTCGGGGAAGCTGACGTCCTTTCCATGGCTGCTCGCCTGTGTTGCCACCTGGATTCTGC
GCGGGACGTCCTTCTGCTACGTCCCTTCGGCCCTCAATCCAGCGGACCTTCCTTCCCGCGGCCTGCTGCCGGC
TCTGCGGCCTCTTCCGCGTCTTCGCCTTCGCCCTCAGACGAGTCGGATCTCCCTTTGGGCCGCCTCCCCGCAAA
CGGGGGAGGCTAACTGAAACACGGAAGGAGACAATACCGGAAGGAACCCGCGCTATGACGGCAATAAAAAGAC
AGAATAAAACGCACGGGTGTTGGGTCGTTTGTTCATAAACGCGGGGTTCGGTCCCAGGGCTGGCACTCTGTCG
ATACCCCACCGAGACCCCATTGGGGCCAATACGCCCGCGTTTCTTCCTTTTCCCCACCCCACCCCCCAAGTTCG
GGTGAAGGCCCAGGGCTCGCAGCCAACGTCGGGGCGGCAGGCCCTGCCATAGCAGATCTGCGCAGCTGTTTG
CAAAAGCCTAGGCCTCCAAAAAAGCCTCCTCACTACTTCTGGAATAGCTCAGAGGCAGAGGCGGCCTCGGCCTC
TGCATAAATAAAAAAAATTAGTCAGCCATGGGGCGGAGAATGGGCGGAACTGGGCGGAGTTAGGGGCGGGATG
GGCGGAGTTAGGGGCGGGACTATGGTTGCTGACTAATTGAGATGCATGCTTTGCATACTTCTGCCTGCTGGGGA
GCCTGGGGACTTTCCACACCTGGTTGCTGACTAATTGAGATGCATGCTTTGCATACTTCTGCCTGCTGGGGAGC
CTGGGGACTTTCCACACCCTAACTGACACACCAGCTGCATTAATGAATCGGCCAACGCGCGGGGAGAGGCGGT
TTGCGTATTGGGCGCTCTTCCGCTTCCTCGCTCACTGACTCGCTGCGCTCGGTCGTTCGGCTGCGGCGAGCGG
TATCAGCTCACTCAAAGGCGGTAATACGGTTATCCACAGAATCAGGGGATAACGCAGGAAAGAACATGTGAGCA
AAAGGCCAGCAAAAGGCCAGGAACCGTAAAAAGGCCGCGTTGCTGGCGTTTTTCCATAGGCTCCGCCCCCCTG
ACGAGCATCACAAAAATCGACGCTCAAGTCAGAGGTGGCGAAACCCGACAGGACTATAAAGATACCAGGCGTTT
CCCCCTGGAAGCTCCCTCGTGCGCTCTCCTGTTCCGACCCTGCCGCTTACCGGATACCTGTCCGCCTTTCTCCC
TTCGGGAAGCGTGGCGCTTTCTCATAGCTCACGCTGTAGGTATCTCAGTTCGGTGTAGGTCGTTCGCTCCAAGC
TGGGCTGTGTGCACGAACCCCCCGTTCAGCCCGACCGCTGCGCCTTATCCGGTAACTATCGTCTTGAGTCCAAC
CCGGTAAGACACGACTTATCGCCACTGGCAGCAGCCACTGGTAACAGGATTAGCAGAGCGAGGTATGTAGGCG
GTGCTACAGAGTTCTTGAAGTGGTGGCCTAACTACGGCTACACTAGAAGAACAGTATTTGGTATCTGCGCTCTGC
TGAAGCCAGTTACCTTCGGAAAAAGAGTTGGTAGCTCTTGATCCGGCAAACAAACCACCGCTGGTAGCGGTGGT
TTTTTTGTTTGCAAGCAGCAGATTACGCGCAGAAAAAAAGGATCTCAAGAAGATCCTTTGATCTTTTCTACGGGGT
CTGACGCTCAGTGGAACGAAAACTCACGTTAAGGGATTTTGGTCATGAGATTATCAAAAAGGATCTTCACCTAGA
TCCTTTTAAATTAAAAATGAAGTTTTAAATCAATCTAAAGTATATATGAGTAAACTTGGTCTGACAGTTACCAATGC
TTAATCAGTGAGGCACCTATCTCAGCGATCTGTCTATTTCGTTCATCCATAGTTGCCTGACTCCCCGTCGTGTAG

ATAACTACGATACGGGAGGGCTTACCATCTGGCCCCAGTGCTGCAATGATACCGCGAGACCCACGCTCACCGG
CTCCAGATTTATCAGCAATAAACCAGCCAGCCGGAAGGGCCGAGCGCAGAAGTGGTCCTGCAACTTTATCCGCC
TCCATCCAGTCTATTAATTGTTGCCGGGAAGCTAGAGTAAGTAGTTCGCCAGTTAATAGTTTGCGCAACGTTGTT
GCCATTGCTACAGGCATCGTGGTGTCACGCTCGTCGTTTGGTATGGCTTCATTCAGCTCCGGTTCCCAACGATC
AAGGCGAGTTACATGATCCCCCATGTTGTGCAAAAAAGCGGTTAGCTCCTTCGGTCCTCCGATCGTTGTCAGAA
GTAAGTTGGCCGCAGTGTTATCACTCATGGTTATGGCAGCACTGCATAATTCTCTTACTGTCATGCCATCCGTAA
GATGCTTTTCTGTGACTGGTGAGTACTCAACCAAGTCATTCTGAGAATAGTGTATGCGGCGACCGAGTTGCTCTT
GCCCGGCGTCAATACGGGATAATACCGCGCCACATAGCAGAACTTTAAAAGTGCTCATCATTGGAAAACGTTCTT
CGGGGCGAAAACTCTCAAGGATCTTACCGCTGTTGAGATCCAGTTCGATGTAACCCACTCGTGCACCCAACTGA
TCTTCAGCATCTTTTACTTTCACCAGCGTTTCTGGGTGAGCAAAAACAGGAAGGCAAAATGCCGCAAAAAAGGGA
ATAAGGGCGACACGGAAATGTTGAATACTCATACTCTTCCTTTTTCAATATTATTGAAGCATTTATCAGGGTTATTG
TCTCATGAGCGGATACATATTTGAATGTATTTAGAAAAATAAACAAATAGGGGTTCCGCGCACATTTCCCCGAAAA
GTGCCACCTGACGTCGACGGATCGGGAGATCTCCCGATCCCCTATGGTGCACTCTCAGTACAATCTGCTCTGAT
GCCGCATAGTTAAGCCAGTATCTGCTCCCTGCTTGTGTGTTGGAGGTCGCTGAGTAGTGCGCGAGCAAAATTTA
AGCTACAACAAGGCAAGGCTTGACCGACAATTGCATGAAGAATCTGCTTAGGGTTAGGCGTTTTGCGCTGCTTC
GCGATGTACGGGCCAGATATACGCGTT
